# Chemoselective Stabilized Triphenylphosphonium Probes for Capturing Reactive Carbonyl Species and Regenerating Covalent Inhibitors with Acrylamide Warheads in Cellulo

**DOI:** 10.1021/jacs.4c09727

**Published:** 2024-12-27

**Authors:** Ai-Lin Chen, Zih-Jheng Lin, Hsiao-Yu Chang, Tsung-Shing Andrew Wang

**Affiliations:** Department of Chemistry and Center for Emerging Material and Advanced Devices, National Taiwan University, Taipei 106319, Taiwan (R.O.C.)

## Abstract

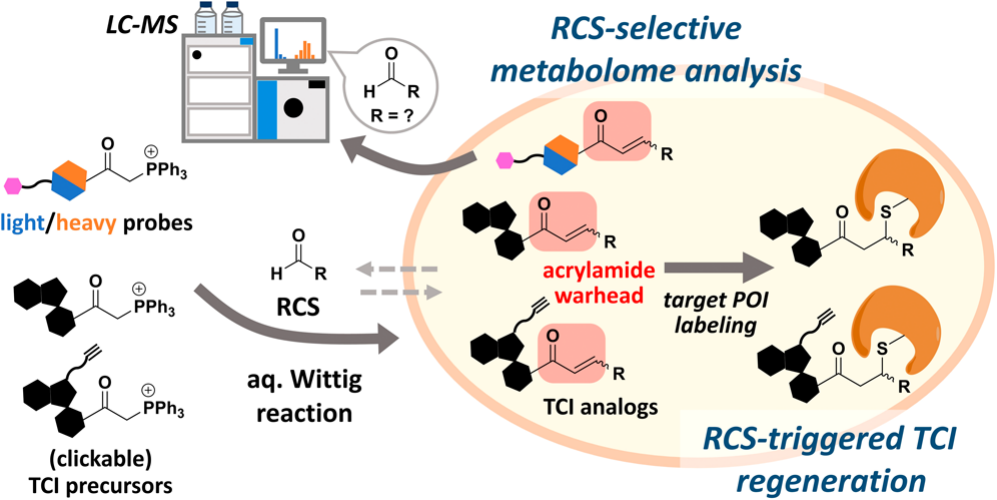

Reactive carbonyl species (RCS) are important biomarkers of oxidative stress-related diseases because of their highly reactive electrophilic nature. Despite their potential as triggers for prodrug activation, selective labeling approaches for RCS remain limited. Here, we utilized triphenylphosphonium groups to chemoselectively capture RCS via an aqueous Wittig reaction, forming α,β-unsaturated carbonyls that enable further functionalization. We first designed native (light) and deuterated (heavy) probes to facilitate RCS metabolomic identification through distinct MS isotope patterns. This approach allowed us to capture and relatively quantify several endogenous RCS related to advanced lipoxidation/glycation end products (ALEs/AGEs). Second, we demonstrated that various endogenous RCS can trigger the in situ generation of acrylamide warheads of targeted covalent inhibitors (TCIs) with different substituents. These structural variations influence their protein binding profiles and consequently alter their cytotoxicity, which is beneficial for the development of inhibitor cocktails.

## Introduction

Reactive carbonyl species (RCS) are metabolites derived from the oxidation of biomolecules. As reactive electrophiles, they readily modify nucleophilic sites on proteins, aminophospholipids, and nucleic acids through Michael addition or Schiff base formation. The accumulation of such biomolecular adducts and cross-links could lead to advanced glycation or lipoxidation end products (AGEs or ALEs), ultimately resulting in cellular and organ dysfunction.^1^ Nevertheless, comprehensive utilization of RCS as disease biomarkers for diagnostics and treatments still requires more tools.^2^

To profile metabolomes and explore their biochemical roles, chemoselective probes are promising tools because they selectively label small molecules based on chemical reactivity.^3^ Not surprisingly, chemoselective probes targeting diverse reactive groups, such as thiols, acids, amines, and carbonyls, have been widely developed.^4^ Often, click handles are appended on probes as flexible tagging strategies. In addition, isotope tags have also been introduced to provide unique isotopic signatures in mass spectrometry (MS), enabling much easier recognition of labeled substances.^5^ Various chemical approaches have been described for selectively targeting bioreactive electrophiles. Amino and homoallylamine groups for formaldehydes^6^ and *o*-phenylenediamine and benzohydrazide groups for dicarbonyls^7^ have been incorporated into the design of turn-on fluorescent probes. Aminooxy, hydroxylamine and hydrazine groups have been widely employed for modifications and conjugations^8,9^ and have been applied in carbonyl metabolome analysis.^10^

Notably, phosphorus ylides stabilized by electron withdrawing groups (EWGs) have been utilized in selective protein and peptide modifications through reactions with *N*-terminal aldehyde handles.^11,12^ Moreover, α,β-unsaturated carbonyl moieties can be assembled from in situ-generated ylides and further functionalized through Michael additions.^12^ These examples imply that Wittig reactions might be expandable to label RCS chemoselectively. Coincidentally, plain and substituted acrylamides, amide-based α,β-unsaturated carbonyl structures, are commonly used as warheads in targeted covalent inhibitors (TCIs), including ibrutinib (Ibr), spebrutinib (Spe), afatinib, dacomitinib, and neratinib.^13^ To increase selectivity and potency, various TCIs with substitutions of their native warheads and/or protein-targeting moieties have been rationally designed and screened.^14^ However, the synthesis process is often nonuniversal and tedious. By reacting with different RCS, we expect that TCI analogs with structural variations adjacent to acrylamide can be efficiently constructed.

Intuitively, in addition to chemoselective labeling, we hypothesized that stabilized ylides are also potential precursors for generating covalent inhibitors with Michael electrophiles. With both stability and availability for derivatization,^15^ amide-stabilized phosphorus ylides seem to be particularly suitable precursors for our design. Here, as a proof of concept, we designed triphenylphosphonium probes for the RCS selective labeling. Additionally, TCI precursors based on two Bruton’s tyrosine kinase (BTK) inhibitors, Ibr and Spe, were also described for the purpose of regenerating the acrylamide warheads of the native inhibitors.

## Results and Discussion

### Evaluating the Aqueous Wittig Reaction Conditions

The stabilized triphenylphosphonium probe **Tz-PPh**_**3**_**(1)** was designed for evaluating chemoselective labeling via the aqueous Wittig reaction (Scheme S1). We first assessed the reaction efficiency under different conditions. The reaction did not occur in phosphate-buffered saline (PBS) (pH 7.4 or 8.0). In contrast, the reaction becomes more efficient with NaHCO_3_,^16^ indicating that alkaline conditions are not a prerequisite for inducing the reaction (Figure S1a). Additionally, **Tz-PPh**_**3**_ disappeared more quickly under NaHCO_3_. Since the base-promoted decomposition of triphenylphosphonium reported previously,^15^ we suspected that the triphenylphosphonium group could be hydrolyzed to the acetamido group as the side product (green triangle). To confirm its structure, we synthesized acetamido Compound **20** (Scheme S1). Using high-performance liquid chromatography (HPLC) and MS, the retention time and observed mass of **20** were consistent with those of the side products (Figure S2).

Hypothetically, NaHCO_3_ somehow activated phosphonium groups and consequently promoted both aqueous Wittig and hydrolysis reactions. Mechanistically, carbon dioxide, which is in equilibrium with NaHCO_3,_ first reacts with phosphonium ylides through α-carboxylation. The resulting carboxylate served as an additional EWG, making its adjacent α-hydrogen more acidic and its deprotonation easier. The highly stabilized carbanion intermediate **X** reacts with substrates (aldehydes), to complete the aqueous Wittig reaction, or with water, to cause hydrolysis. In the aqueous Wittig pathway, single addition (+1 aldehyde) to **X** results in the expected alkene product. At high concentrations, double addition (+2 aldehydes) was also observed (data not shown). In the hydrolysis pathway, the triphenylphosphonium moiety is lost by base-promoted decomposition, and the resulting enol tautomerizes into the acetamido side product, such as **20** (Figure S3). Different concentrations of NaHCO_3_ were also tested, and only minor differences in reaction time and yield were observed (Figure S1b). Ultimately, the concentration of NaHCO_3_ used was 26 mM, which is within the range found in typical culture media and under physiological conditions.^17^

### Stabilized Triphenylphosphonium Probes Selectively Labeled with RCS

In addition to **Tz-PPh**_**3**_**(1)**, the commercial aminooxy probe **Tz-ONH**_**2**_**(2)** was utilized to demonstrate differences in chemoselectivity. Furthermore, deuterated probes, **D-Tz-PPh**_**3**_**(3)** and **D-Tz-ONH**_**2**_**(4)**, were synthesized to introduce unique isotopic signatures for easy identification of labeled peaks via subsequent liquid chromatography–mass spectrometry (LC–MS) analysis.^5^ Notably, when deuterated probes were synthesized, CD_3_CN or deuterated intermediates underwent inevitable H/D exchange during condensation between nitriles and hydrazines. As a result, mixed deuterated (d0 to d3) probes were obtained (Figure S4). Although counterintuitive, these arbitrary but unique mixed isotope patterns can nevertheless facilitate peak identification.^5^

**Figure 1:**
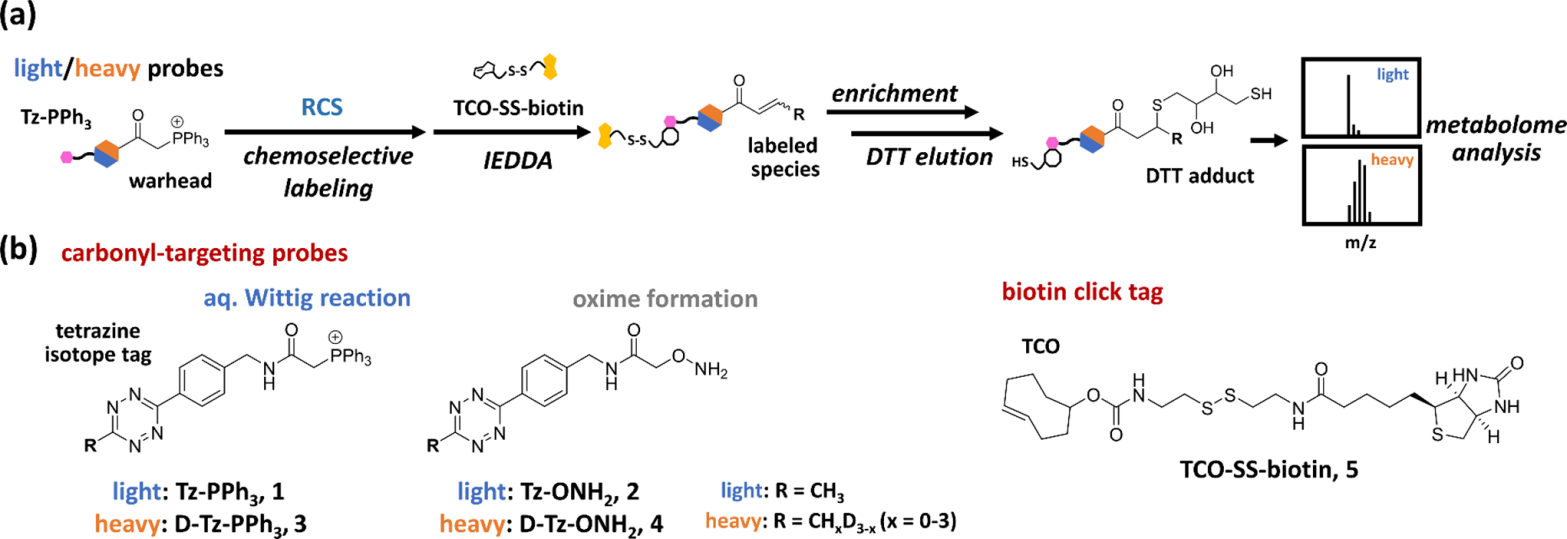
Schematic and chemical structures. (a) Workflow of chemoselective labeling. Detailed reaction scheme is shown in Figure S10. (b) Chemical structures of carbonyl-targeting probes and the biotin click tag.

**Figure 2:**
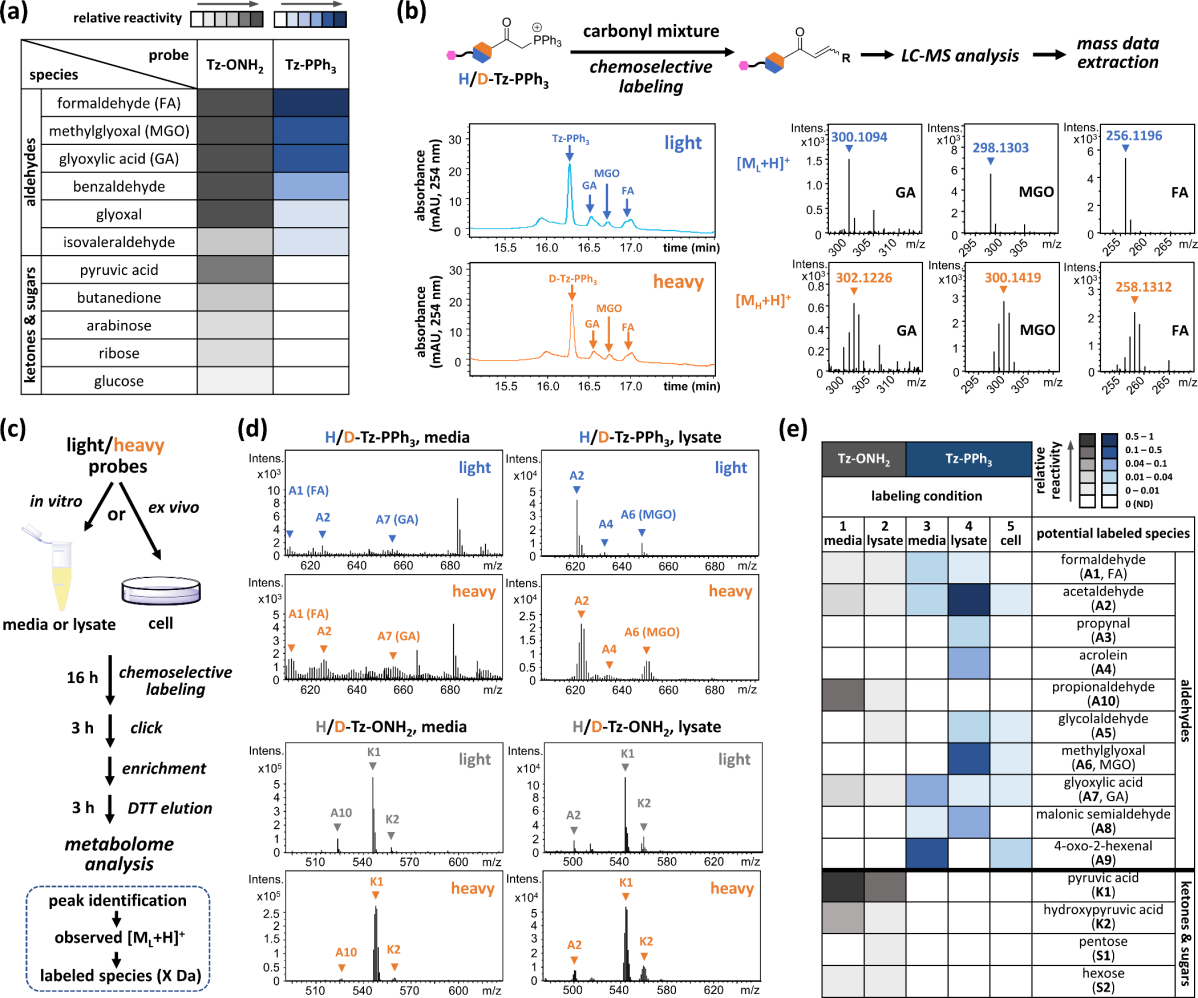
Validating the chemoselective labeling of carbonyls in vitro and in living cells. (a) Heatmap depicting the reactivity of **Tz-PPh**_**3**_**(1)** and **Tz-ONH**_**2**_**(2)** with carbonyls. Quantification is based on the AUC integrated from chromatographs. (b) Chromatographs and MS analysis of **H/D-Tz-PPh**_**3**_-labeled carbonyls in a mixture (FA, MGO, and GA: 50 μM each; pyruvic acid, glucose, ribose: 5 mM each). (c) Workflow of in vitro and ex vivo chemoselective labeling. RPMI medium, Ramos cell lysate, or cells were incubated with probes, followed by IEDDA with **TCO-SS-biotin (5)**, enrichment, elution, and metabolome analysis. (d) MS analysis of labeled species in media and Ramos cell lysates. Labeled species are abbreviated as shown in (e). (e) Heatmap depicting labeled species intensity by various probes and labeling conditions. Color scale is based on the AUC integrated from EICs of labeled species.

Tetrazine groups were appended in all four probes as bioorthogonal handles to click with the cleavable disulfide-containing **TCO-SS-biotin (5)** via inverse electron-demand Diels–Alder reaction (IEDDA) for further enrichment. After the capture of carbonyls, attachment of biotin handles, and streptavidin enrichment, the probe-labeled species were eluted using dithiothreitol (DTT) via disulfide cleavage for further MS analysis (Figure 1). The reactivities of **Tz-PPh**_**3**_ and **Tz-ONH**_**2**_ were assessed via HPLC. Several carbonyl species, including formaldehyde (FA), dicarbonyls (methylglyoxal (MGO), glyoxylic acid (GA), glyoxal, pyruvic acid, and butanedione), aliphatic carbonyls (isovaleraldehyde), aromatic carbonyls (benzaldehyde), and sugars (arabinose, ribose, and glucose), were tested. **Tz-ONH**_**2**_ exhibited high reactivity toward pyruvic acid and most aldehydes, which were consumed within 0.5 h along with the formation of the desired single-labeled products. Single- or double-labeled products were observed with MGO and glyoxal. With less reactive compounds, such as sugars, the desired products could still be observed within 4 h (Figures 2a and S5). In contrast, when reacted with RCS, FA, MGO, and GA, **Tz-PPh**_**3**_ was consumed within 4 h, and the corresponding labeled products and side products **20** formed. With less reactive carbonyls, benzaldehyde, glyoxal, and isovaleraldehyde, **Tz-PPh**_**3**_ was still consumed within 4 h, but **20** was predominantly the major peak. For ketones and sugars, even at a high concentration (5 mM), no labeled products were formed, and only **20** were detected (Figures 2a and S6). In brief, our results suggested that triphenylphosphonium probes showed high chemoselectivity toward various carbonyl species.

### Carbonyl Metabolites Were Captured by Light/Heavy Tetrazine Probes and Monitored via MS

**Tz-PPh**_**3**_**(1)** (light) and **D-Tz-PPh**_**3**_**(3)** (heavy) were applied to a carbonyl mixture containing FA, MGO, GA, pyruvic acid, glucose, and ribose to test the reactivity and selectivity in complex samples. The labeled reactions were analyzed by LC–MS. Our selection criteria required that labeled species appear in both light and heavy channels at the same retention time and that their masses ([M_L_ + H]^+^ and [M_H_ + H]^+^) and corresponding isotope patterns be aligned. The results clearly revealed that only three reactive carbonyls were labeled (Figure 2b), which is consistent with the previous HPLC results (Figures 2a and S6).

To characterize the more complicated RCS metabolome inside the cells, **H/D-Tz-PPh**_**3**_**(1/3)** were applied to the cell culture media RPMI 1640, Ramos cell lysate and Ramos, Burkitt lymphoma cells. **H/D-Tz-ONH**_**2**_**(2/4)** were also applied to compare the labeling selectivity. After labeling, clicking with **TCO-SS-biotin (5)**, enrichment, and DTT elution (Figure 2c), we observed that DTT underwent Michael addition to the α,β-unsaturated carbonyl moieties generated from the Wittig reaction. The labeled species were identified using the above criteria. The labeling efficiency of different labeled species was quantified via their area under the curves (AUCs) of MS intensity integrated from extracted ion chromatographs (EICs) and normalized with the highest AUC to represent relative reactivity. In the **Tz-PPh**_**3**_ labeling groups, the AUCs were normalized to acetaldehyde in the lysate, and in the **Tz-ONH**_**2**_ labeling groups, the AUCs were normalized to pyruvic acid in media. The potential labeled carbonyls were searched for in HMDB (https://hmdb. ca/), and the results are summarized in a heatmap (Figure 2d,e). In addition, we observed both reduced and oxidized adducts, and the dominant species were oxidized adducts.^18^ Notably, even under different elution conditions, the differently labeled products presented similar retention times, which might have resulted from negligible structural differences between them.

Both pyruvic acid and glucose in the medium were labeled when **H/D-Tz-ONH**_**2**_ were applied to media. FA, acetaldehyde, propionaldehyde, GA, and hydroxypyruvic acid were also labeled (Figure 2d,e Column 1, S7 and S8). When **H/D-Tz-PPh**_**3**_ were applied to media, FA, acetaldehyde, and GA were labeled. Other promising labeled species, such as malonic semialdehyde and 4-oxo-2-hexenal, were identified (Figures 2d, 2e Column 3, S10 and S11). Curiously, several RCS were found in both sets of probes. High concentrations of glucose and nutrients can undergo degradation, resulting in aldehydes and reactive glucose degradation products (GDPs).^19^ When **H/D-Tz-ONH**_**2**_ were applied to the cell lysates (from 10^7^ cells), the major labeled species was pyruvic acid. The labeling results for the lysate were similar to those for media, except for the additional labeling of glycolaldehyde and pentose, which were detected only in the lysate (Figures 2d,e Column 2, S7 and S9). In addition to the aldehydes observed in media, propynal, acrolein, glycolaldehyde, and MGO were detected when **H/D-Tz-PPh**_**3**_ was applied to the cell lysates (Figures 2d,e Column 4, S10 and S12).

Our labeling results clearly showed that aminooxy probes could react with various types of carbonyls, including aldehydes, ketones, and sugars. Although aminooxy groups possess high carbonyl reactivity, they can be easily interfered with by other carbonyls present in media or cells, such as pyruvic acid (1 mM in RPMI, 77–201 μM in cells).^20^ In contrast, triphenylphosphonium probes are highly selective toward reactive aldehydes and are therefore less likely to interfere with other carbonyls in complex biological systems. Consequently, **H/D-Tz-PPh**_**3**_ are promising for ex vivo labeling. However, when 10^7^ cells were labeled, the reaction efficiency significantly decreased due to the decreased pH caused by acidification of live cells. When fewer cells (5 × 10^5^) were used for ex vivo labeling, some labeled signals, such as glycolaldehyde and MGO, were also observed in the lysate. These two peaks were not detected in media (Figures 2e Column 5, S10 and S13). The MS signal intensity in cells was lower than the signals of labeled species in the lysate, possibly because fewer cells were used in labeling.

In short, nine carbonyls are labeled by **H/D-Tz-PPh**_**3**_. Among them, FA, acetaldehyde, propynal, and malonic semialdehyde are endogenously generated through metabolic processes.^21^ Acrolein, glycolaldehyde, and 4-oxo-2-hexenal are products of lipid peroxidation.^22,23^ Moreover, glycolaldehyde, MGO, and GA are related to advanced glycation end products (AGEs).^24^ Notably, malonic semialdehyde can undergo decarboxylation to form acetaldehyde,^25^ which could explain the high intensity of labeled acetaldehyde. The residual exogenous ethanol during sanitization may also contribute to some extent.^26^ Interestingly, the mass of the labeled species was generally <150 Da. No significant signals had large exact masses (>150 Da). Presumably, some unstable carbonyls and labeling intermediates may undergo further fragmentation during labeling processes and/or LC–MS analysis. Additionally, several identified masses related to unknown species were observed, which might also result from the degradation of larger carbonyls (Figure S10). Instead of DTT, certain cellular nucleophiles might be responsible for Michael adducts, which can also complicate our mass interpretation.

### Identification and Relative Quantification of Endogenous RCS among Cell Types

**Figure 3:**
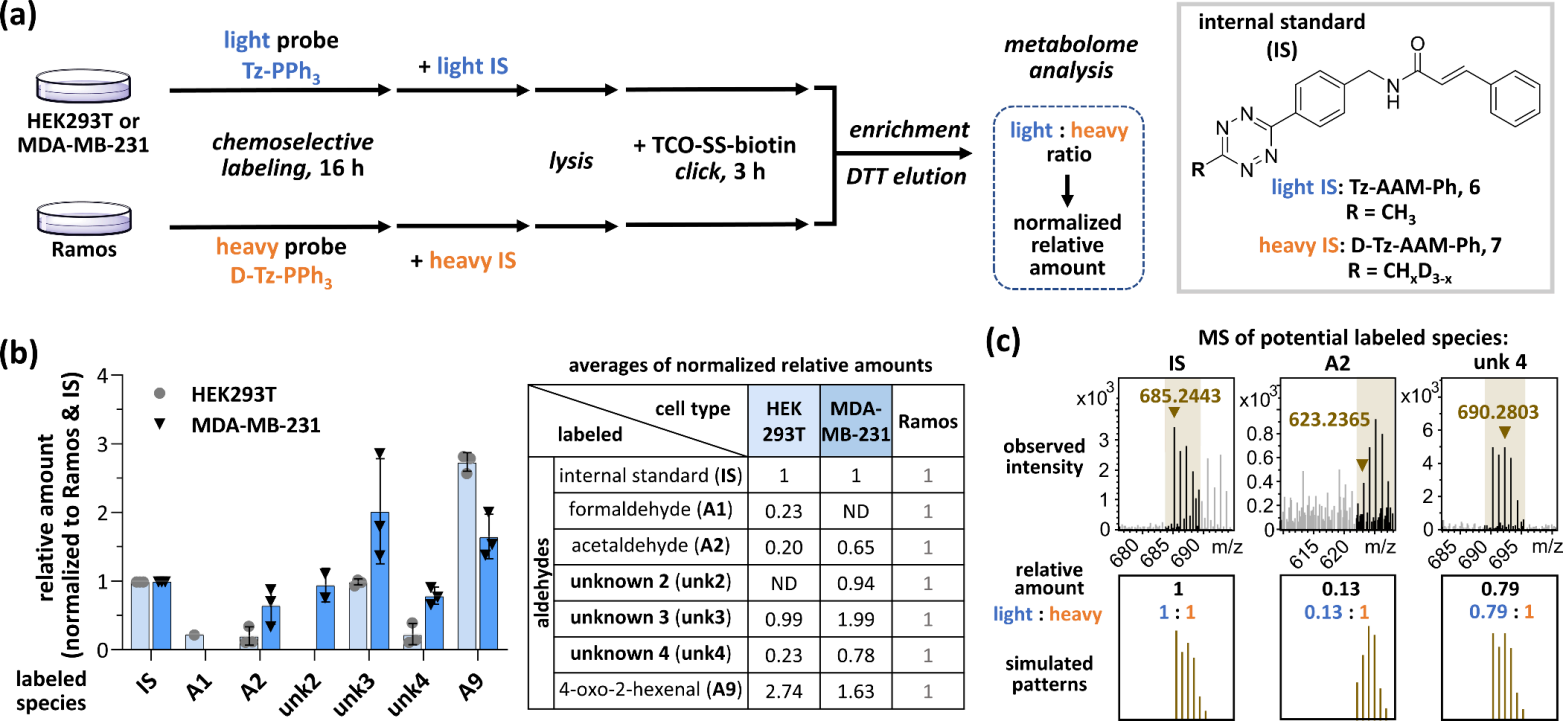
Demonstration of ex vivo RCS relative quantification. (a) Workflow of ex vivo RCS relative quantification. MDA-MB-231 or HEK293T cells and Ramos cells were incubated with **H/D-Tz-PPh**_**3**_**(1/3)** respectively, followed by the addition of the corresponding **IS (6/7)**, cell lysis, installation of **TCO-SS-biotin (5)** through IEDDA, enrichment, elution, and LC–MS metabolome analysis. (b) Quantification results of normalized relative amounts of labeled species in HEK293T/Ramos and MDA-MB-231/Ramos “mixed” triplicates. (c) Observed MS patterns and the simulated patterns of the corresponding ratios of mixed **H/D-Tz-PPh**_**3**_.

In addition to Ramos cells, **H/D-Tz-PPh**_**3**_**(1/3)** were also applied to MDA-MB-231 cells, which are metastatic breast cancer cells, and HEK293T cells, which are human embryonic kidney cells, for metabolome analysis (Figure S14). Interestingly, the RCS profiles qualitatively varied among the cell types. However, accurate comparison of their RCS levels should be more informative. To better quantify and compare cellular RCS levels, in our setup, two cell lines were labeled separately with light (**Tz-PPh**_**3**_) or heavy (**D-Tz-PPh**_**3**_) probes, clicked, and combined before enrichment and LC–MS analysis. To reduce error from sample preparation and detection, **Tz-AAM-Ph (6)** and **D-Tz-AAM-Ph (7)** were synthesized as internal standards (ISs) for LC–MS analysis (Figures 3a and S15).

″Positive” labeling events can be rapidly identified by unique mixed light/heavy MS isotope patterns. In HEK293T/Ramos “mixed” triplicates, we identified IS, FA, acetaldehyde, two unknown species, and 4-oxo-2-hexenal as potential labeled species, whereas FA was observed only once (Figure S16). In MDA-MB-231/Ramos “mixed” triplicates, we identified IS, acetaldehyde, three unknown species, and 4-oxo-2-hexenal, whereas unknown 2 was observed only twice (Figure S17). The light/heavy ratios of the labeled species were subsequently estimated via linear interpolation between each individually extracted light and heavy MS pattern from the eluted acetaldehyde products (Figure S18a). The acetaldehyde products were chosen to calculate ratios because the high intensities observed via LC–MS enabled minimal interference from background noise.

Normalized relative amounts of their corresponding labeled species can be compared across different cell types by setting “Ramos cells” to 1 in all ratios and normalizing to the corresponding IS in each “mixed” sample (Figures 3b, S16 and S17). Notably, the light/heavy ratios of the IS peaks were consistently close to 1 in most of the “mixed” samples, suggesting great reducibility and minimal bias during sample preparation. Once again, in several labeled peaks, the high consistency between the observed and simulated light/heavy MS patterns ensured the reliability of our linear interpolation (Figure 3c). Interestingly, the levels of different RCS appeared to vary among cell lines, suggesting their intrinsic heterogeneity. Most RCS presented relatively lower levels in noncancerous HEK293T cells. Curiously, ALE-related 4-oxo-2-hexenal showed the opposite trend. Nevertheless, our results implied that RCS could serve as disease biomarkers.

The capability of our linear interpolation was evaluated. A wide range of light/heavy ratios (4:1 to 1:4) was used to plot a series of simulated MS patterns. When the ratios that exceeded 3-fold (>3:1 or <1:3) were compared, the isotope patterns were almost indistinguishable, for example, between patterns of 4:1 versus 3:1 or 1:3 versus 1:4 (Figure S18b). Thus, the workable range is estimated to be 3:1–1:3 (relative amounts of 3–0.33). Nevertheless, in addition to characterizing the RCS, we have successfully estimated the relative amounts of RCS in different cells. This approach is promising for evaluating cellular RCS levels under different conditions, offering a novel strategy for simultaneously quantifying various RCS.

### Ibrutinib Analogs Were Generated In Situ from Endogenous RCS and Covalently Bound to BTK

**Figure 4:**
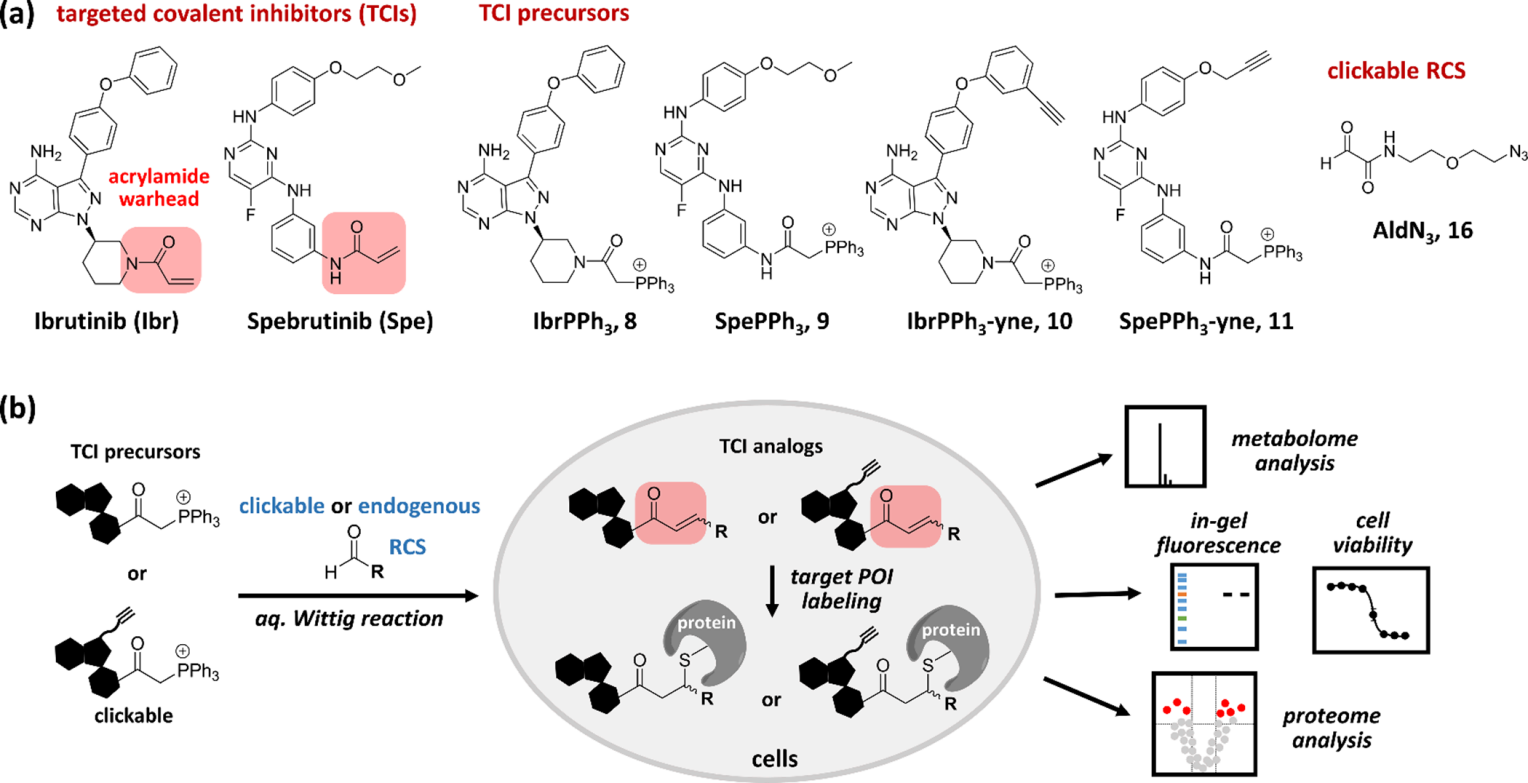
Schematic and chemical structures. (a) Chemical structures of TCIs, TCI precursors, and clickable RCS. (b) Schematic illustration of TCI regeneration and its applications.

The chemoselectivity of the stabilized triphenylphosphonium groups allowed us to selectively label endogenous RCS and subsequently generate new reactive α,β-unsaturated carbonyl moieties in situ, which are potentially suitable for further functionalization. In fact, we attempted to transform the resulting α,β-unsaturated carbonyls into the key acrylamide warheads commonly used in TCI designs. Consequently, as our proof of concept, TCI precursors were designed based on Ibr and Spe, **IbrPPh**_**3**_**(8)** and **SpePPh**_**3**_**(9)**, which are equipped with stabilized triphenylphosphonium groups as the acrylamide precursor (Schemes S4 and S7). TCI acrylamide analogs were generated in situ after reacting with RCS via the aqueous Wittig reaction. The cytotoxicities of the TCI precursors and analogs were then evaluated. Additionally, clickable alkynyl TCI precursors, **IbrPPh**_**3**_**-yne (10)** and **SpePPh**_**3**_**-yne (11)**, were also prepared to assess the efficiency of acrylamide formation (Schemes S5 and S7). Their alkyne handles can be clicked with various commercial and homemade functional probes (**12**-**15**, Scheme S3 and Figure S19), allowing further applications, such as in-gel fluorescence, metabolome and proteome analysis (Figure 4). In the synthesis of TCI precursors, unknown phospho-containing organic impurities were observed after triphenylphosphine substitution and were difficult to remove completely. Nevertheless, the impurities had no effect on the following labeling reactions.

The reactivities of the TCI precursors with aldehydes were again assessed via HPLC. Based on previous results, the three most reactive aldehydes, FA, MGO, and GA, were chosen to evaluate the reactivities of **IbrPPh**_**3**_**-yne** and **SpePPh**_**3**_**-yne**. The corresponding TCI acrylamide products and hydrolysis side products were observed for both precursors. FA showed the highest reactivity, with the reaction completed after 16 h. When MGO and GA were used, the remaining TCI precursors could still be observed after 16 h. In the reactivity test of **SpePPh**_**3**_**-yne**, 10 equiv of MGO was required to obtain detectable product signals (Figure S20).

Because of its relatively high reactivity, **IbrPPh**_**3**_**-yne** was used for further in vitro and ex vivo reactivity evaluations. We synthesized a clickable RCS substrate, **AldN**_**3**_**(16)**, to ensure covalent labeling between BTK and in situ-generated acrylamide warheads (Figure 4a and Scheme S6). The dicarbonyl aldehyde was chosen for derivatization because of its high reactivity. In addition, the azide was included as the second orthogonal click handle to track and distinguish the RCS probe from the alkynyl TCI precursors after labeling. The assembly between **IbrPPh**_**3**_**-yne** and **AldN**_**3**_ was completed in 4 h without the hydrolysis side product detected (Figure S21), confirming their high reactivity.

**Figure 5:**
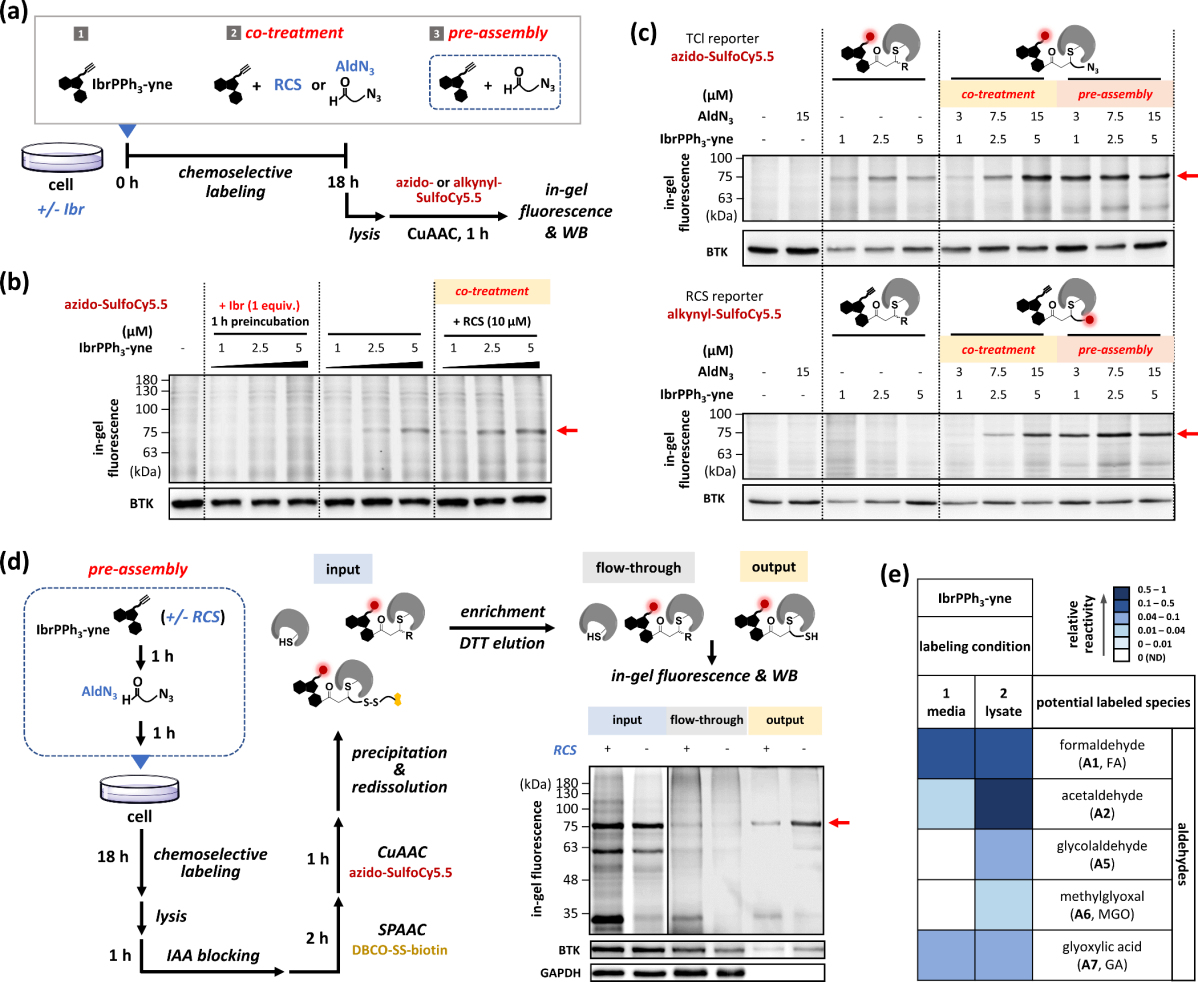
Validating TCI acrylamide analog generation from ibrutinib precursors via endogenous RCS. (a) Labeling workflow. (b) TCI regeneration in living cells. Ramos cells were treated with **IbrPPh**_**3**_**-yne (10)** after preincubation with or without Ibr, followed by lysis, CuAAC with **azido-SulfoCy5.5 (12)**, and detection by in-gel fluorescence. (c) TCI regeneration and acrylamide formation in living cells. For cotreatment, Ramos cells were treated with **IbrPPh**_**3**_**-yne** and **AldN**_**3**_**(16)** simultaneously. For preassembly, **IbrPPh**_**3**_**-yne** (500 μM) and **AldN**_**3**_ (1.5 mM) were preassembled in PBS with 26 mM NaHCO_3_ at 37 °C for 1 h. After treatment, the Ramos cells were lysed, subjected to **12** or **alkynyl-SulfoCy5.5 (13)** for CuAAC, and detected by in-gel fluorescence. (d) Confirmation of the generated double-clickable Ibr derivative. **IbrPPh**_**3**_**-yne** (250 μM) and **AldN**_**3**_ (750 μM) were preassembled for 1 h with or without RCS (FA, MGO, and GA; 125 μM each). Ramos cells were treated with **IbrPPh**_**3**_**-yne** crude solution (2.5 μM), followed by lysis, IAA blocking (40 mM), SPAAC with **DBCO-SS-biotin (14)** (50 μM), and CuAAC with **12**. Proteins were TCA-precipitated, redissolved for streptavidin enrichment, and eluted with DTT (4%, 1 h) and formic acid (1%, 0.5 h). Proteins from different steps of enrichment were detected by in-gel fluorescence and Western blotting. (e) Metabolome analysis of **IbrPPh**_**3**_**-yne** in media and Ramos cell lysates. Media or cell lysate was treated with **IbrPPh**_**3**_**-yne** (100 μM, 37 °C, 16 h) and clicked with **azido-SS-biotin (15)** (100 μM, 37 °C, 3 h). Mixture was enriched and detected via LC–MS. Color scale is based on the normalized AUC integrated from EICs of labeled species.

In in situ labeling, Ramos cells were preincubated with or without Ibr before being treated with **IbrPPh**_**3**_**-yne**. Different approaches have been used to introduce substrates into cells. In “co-treatment,” exogenous RCS/**AldN**_**3**_ and **IbrPPh**_**3**_**-yne** were used to treat cells simultaneously. In “pre-assembly,” **AldN**_**3**_ and **IbrPPh**_**3**_**-yne** were assembled in vitro to obtain stronger signals. Labeling was detected through in-gel fluorescence after treatment, cell lysis, and CuAAC with the fluorescence reporters **azido-** or **alkynyl-SulfoCy5.5** (**12** or **13**) (Figure 5a).

After reacting with RCS, **IbrPPh**_**3**_**-yne** can be converted into Ibr acrylamide analogs, enabling the labeling of target protein BTK in cells. Dose-dependent labeling (∼75 kDa, red arrow) was observed when **IbrPPh**_**3**_**-yne** was added alone. The labeling bands became visible at the lowest concentration (1 μM) when exogenous RCS were added, suggesting that additional RCS promoted Ibr analog generation. Furthermore, the labeling clearly competed when Ibr was added, confirming that the labeled protein was indeed BTK (Figure 5b). Taken together, our results implied that **IbrPPh**_**3**_**-yne** alone might react with endogenous RCS and generate Ibr analogs in situ, resulting in BTK labeling.

To ensure covalent labeling between BTK and Ibr acrylamide analogs, **AldN**_**3**_ was introduced as a clickable RCS substrate. Dose-dependent BTK labeling was detected in the presence of both **IbrPPh**_**3**_**-yne** and **AldN**_**3**_ when the TCl reporter **azido-SulfoCy5.5** was clicked (Figure 5c, upper), suggesting that **AldN**_**3**_ acted as an RCS substrate. When clicking with the RCS reporter **alkynyl-SulfoCy5.5**, dose-dependent BTK labeling was detected “only” when both **IbrPPh**_**3**_**-yne** and **AldN**_**3**_ were applied. BTK was not labeled when **IbrPPh**_**3**_**-yne** was added alone (Figure 5c, lower). Taken together, these results indicated that the Ibr precursors were converted to Ibr analogs, which covalently bound to BTK and contained an azido group. Reversible, nonspecific binding or covalent binding caused by protein cysteine sulfenic acids cannot incorporate **AldN**_**3**_ and thus should not contribute to the observed BTK labeling. Therefore, the in situ-generated Ibr analogs likely had acrylamide warheads via the aqueous Wittig reaction between **IbrPPh**_**3**_**-yne** and **AldN**_**3**_.

We performed an exogenous RCS competition assay to further confirm that the generated Ibr analogs contained both alkynyl and azido groups. After treatment, **DBCO-SS-biotin (14)** and **azido-SulfoCy5.5** were introduced sequentially by SPAAC and CuAAC, prior to enrichment and detection by in-gel fluorescence and Western blotting (Figure 5d). Through our workflow, the “positive” protein bands in the final output should be both biotin- and SulfoCy5.5-labeled. As expected, the “output” samples without competition **(−)** exhibited both stronger fluorescence and anti-BTK signals. Moreover, since the exogenous RCS competes with **AldN**_**3**_, the “output” samples with competition **(+)** presented weaker fluorescence and anti-BTK signals. These results implied that the TCI products bound to BTK likely possessed both biotin and SulfoCy5.5 reporters, derivatized from azido and alkynyl groups, respectively. Curiously, the fluorescence intensity decreased after enrichment, possibly due to solvatochromic effects from residual surfactants, such as Triton X-100 and sodium dodecyl sulfate,^27^ remained after washing.

Since Ibr precursors can capture endogenous RCS, metabolome analysis using **IbrPPh**_**3**_**-yne** combined with **azido-SS-biotin (15)** was performed to characterize the potentially captured RCS. Compared with labeling experiments using **H/D-Tz-PPh**_**3**_**(1/3)**, no labeled products were detected when the same number of cell lysates (10^7^ cells) were used. The generated Ibr analogs likely bind proteins more tightly than **Tz-PPh**_**3**_-labeled products do. When more lysates were utilized, more Ibr analogs were consumed to bind excess proteins, leading to fewer free forms for analysis. Consequently, lysates from 10^6^ cells were used for labeling. Owing to the lack of isotope patterns for identification, the **IbrPPh**_**3**_**-yne**-labeled RCS were searched for in the **H/D-Tz-PPh**_**3**_ labeling results (Figure 2e). The labeling efficiency of the different species was normalized to the AUC of acetaldehyde in the lysate. We observed that many RCS, including FA, acetaldehyde, glycolaldehyde, MGO, and GA, were labeled (Figures 5e and S22), suggesting that these endogenous RCS can be promising building blocks for in situ acrylamide-based TCI generation.

While our results revealed a noticeable increase in BTK labeling upon RCS addition (Figure 5b, Lanes 8–10), we aimed to correlate the endogenous RCS levels with the in situ TCI generation efficiency. We sought to modulate cellular RCS levels to reflect biological conditions by regulating reactive oxygen species (ROS).^22^ Regrettably, it might be challenging to use BTK labeling levels to reflect subtle changes in RCS levels (Figure S23). We suspected that the RCS level might already be sufficient for in situ generation of TCIs to label BTK. Additionally, the added ROS triggers and quenchers could influence the reactivity between the probes and RCS or between the TCI and BTK. Furthermore, the RCS level might not be dramatically altered because of cellular scavenging processes to maintain homeostasis.

### Proteome Analysis and Cytotoxicity of In Situ-Generated Ibrutinib Analogs

**Figure 6:**
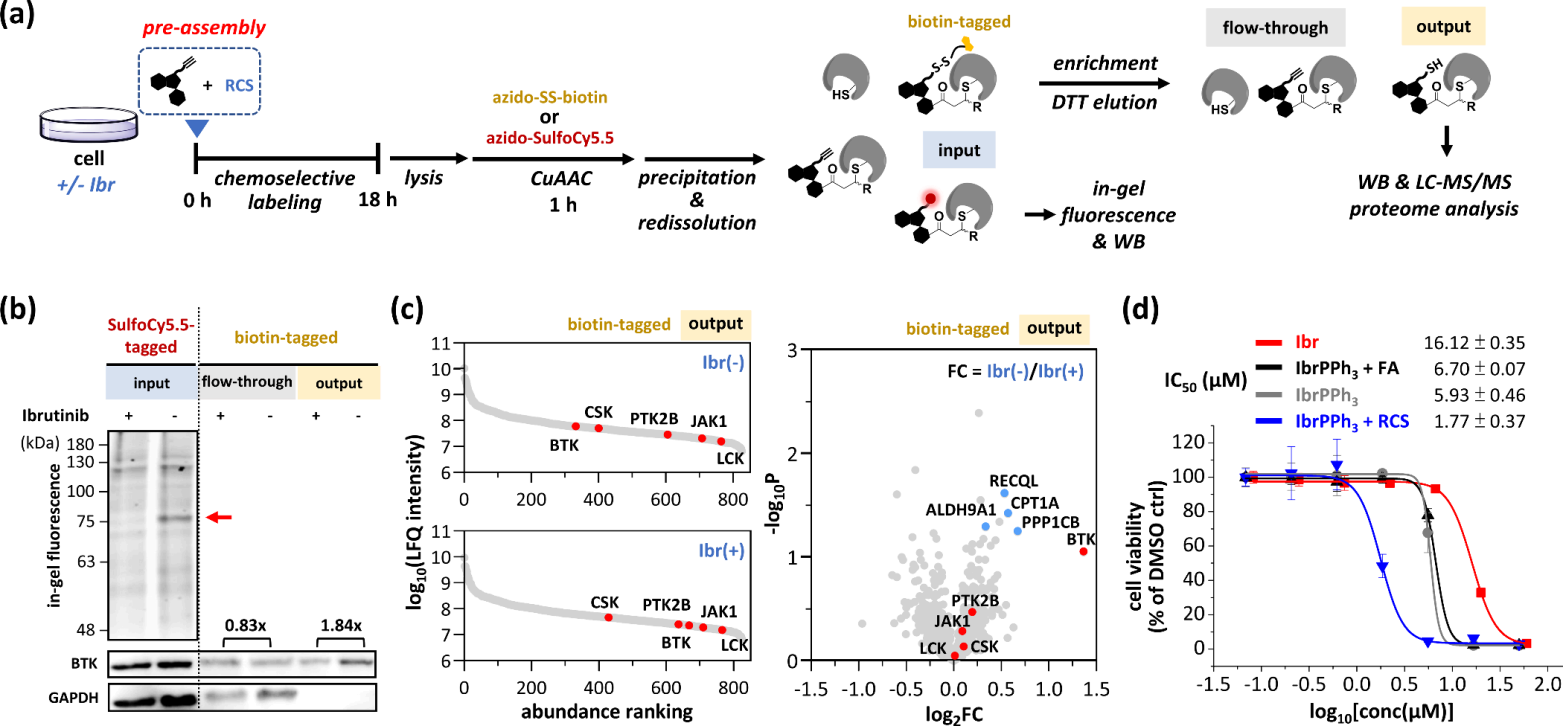
Proteome analysis and cytotoxicity of in situ-generated ibrutinib analogs. (a) Workflow of the proteome experiments. **IbrPPh**_**3**_**-yne (10)** (250 μM) and RCS (FA, MGO, and GA; 125 μM each) were assembled in PBS with 26 mM NaHCO_3_ at 37 °C for 1 h after pretreatment with Ibr (1 μM, 1 h) or not. Ramos cells were treated with **IbrPPh**_**3**_**-yne** crude solution (2.5 μM), followed by lysis, CuAAC with **azido-SS-biotin (15)** (200 μM) or **azido-SulfoCy5.5 (12)** (10 μM). Proteins were TCA-precipitated, redissolved for streptavidin enrichment, and eluted with DTT (4%, 1 h) and formic acid (1%, 0.5 h). Proteins from different steps of enrichment were detected by in-gel fluorescence and Western blotting. (b) In-gel fluorescence and Western blotting of final **Ibr(+)** or **Ibr(−)** enrichment samples prior to proteome analysis. Fold change was quantified via ImageJ. (c) Abundance ranking of **Ibr(+)** or **Ibr(−)** was based on the LFQ intensity and volcano plot of protein abundance changes. (red dots: tyrosine kinase family; blue dots: proteins related to ibrutinib derivatives or cancers). (d) Cytotoxicity of Ibr and its in situ-generated analogs. IC_50_ values are shown.

Proteome analysis was performed via competitive pull-down to identify the proteins labeled by in situ-generated Ibr analogs (Figure 6a). Ramos cells were treated with or without Ibr, followed by incubation with RCS-preassembled **IbrPPh**_**3**_**-yne (10)**. Conceptually, the no-competition groups, **Ibr(−)**, directly reflected the BTK-labeling levels, whereas the competition groups, **Ibr(+)**, served as controls showing nonspecific background labeling. When clicking with **azido-SulfoCy5.5 (12)**, as expected, the fluorescence signal of SulfoCy5.5-tagged BTK (red arrow) was stronger in **Ibr(−)** than in **Ibr(+)**. When clicking with **azido-SS-biotin (15)**, after enrichment and elution, differences in intensity between **Ibr(+)** and **Ibr(−)** were also observed in both “flow-through” and “output” samples (Figure 6b). Compared with the **Ibr(+)** controls, the final elution recovered more biotin-tagged BTK (1.84×) in the **Ibr(−)** “output” group, leaving less nontagged BTK (0.83×) in the **Ibr(−)** “flow-through” group. Moreover, the disappearance of the internal standard, GAPDH, in both **Ibr(−)** and **Ibr(+)** also ensured successful enrichment.

After LC–MS/MS analysis, the proteomics data were normalized by label-free quantification (LFQ) in MaxQuant. The LFQ intensities of the identified proteins were ranked. Many low-abundance proteins, especially those in the tyrosine kinase family (BTK, CSK, PTK2B, JAK1, and LCK), were labeled, enriched, and identified as both **Ibr(+)** and **Ibr(−)**. However, when represented as a volcano plot, with the exception of BTK, other tyrosine kinase proteins remained nearly unchanged between **Ibr(+)** and **Ibr(−)** (Figure 6c, red dots). As expected, BTK had the highest log_2_FC (1.36, ∼2.57-fold), which was statistically significant (*p* < 0.1), indicating that the in situ-generated Ibr analogs were highly selective for BTK. Some proteins that were off-targets of Ibr, such as BLK, TEC, and EGFR,^28^ were not detected. Interestingly, several proteins (*p* < 0.1) were detected as statistically significant (blue dots). For example, ALDH9A1 was selected as its isozymes ALDH1A1 and ALDH1A3 are targeted by alkynyl Ibr analogs.^29^ In addition, RECQL,^30^ CPT1A,^31^ and PPP1CB,^32^ are relevant to cancers, and some of them serve as biomarkers.

Structurally, the generated Ibr analogs differed in the **R** groups adjacent to the acrylamide warheads (Figure 4b). This structural variation should affect the protein binding profile and ultimately alter the cytotoxicity of Ibr analogs. Therefore, their cytotoxicity from various sources was assessed and compared with that of the native Ibr. Impressively, all three generated Ibr analogs, including **IbrPPh**_**3**_**+FA**, **IbrPPh**_**3**_ (+endogenous RCS), and **IbrPPh**_**3**_**+RCS**, showed significantly better cytotoxicity than Ibr did (Figure 6d). Both Ibr and **IbrPPh**_**3**_**+FA** have the same acrylamide warhead (**R** = H) and should exhibit similar IC_50_ values (16.12 and 6.70 μM), where the difference may be due to the freshness of the warheads. More interestingly, **IbrPPh**_**3**_ and **IbrPPh**_**3**_**+RCS** presented much lower IC_50_ values (5.93 and 1.77 μM), strongly suggesting that mixtures of Ibr analogs might have been beneficial as inhibitor cocktails. This finding also suggested that the difference in cytotoxicity primarily resulted from variations in the **R** groups. Nevertheless, when endogenous RCS alone are used, **IbrPPh**_**3**_ should be sufficient to generate Ibr analogs in situ. The clickable **IbrPPh**_**3**_**-yne** (3.40 μM) behaved similarly to **IbrPPh**_**3**_, showing that the alkynyl group had little cytotoxic interference (Figure S24a). This finding strengthens the idea that endogenous RCS are feasible triggers for in situ acrylamide generation. The cytotoxicities of RCS and FA were evaluated to serve as additional controls. RCS (FA, MGO, and GA, 50 μM each) showed no cytotoxicity, and FA exhibited moderate toxicity at 150 μM.^33^ Triphenylphosphine oxide, the side product of the Wittig reaction, also exhibited minimal cytotoxicity (Figure S24d,e). The acetamido side products generated through hydrolysis might contribute to the observed cytotoxicity to a certain degree.^34^

### Demonstration of In Situ TCI Regeneration by Spebrutinib Precursors

The efficiency of in situ generation of Spe analogs was also evaluated to expand the ability of our aqueous Wittig reaction in the TCI assembly. Competition and in situ reactivity studies were performed to ensure that **SpePPh**_**3**_**-yne (11)** reacted with endogenous RCS and formed Spe analogs. BTK was labeled when **SpePPh**_**3**_**-yne** was added alone, and the labeling was competed by Spe pretreatment. Cotreatment with exogenous RCS enhanced BTK labeling (Figure S25a). These results were consistent with those of **IbrPPh**_**3**_**-yne (10)**, confirming that the in situ generation strategy could be extended to other acrylamide-TCIs. Metabolome analysis of **SpePPh**_**3**_**-yne** was performed to characterize the captured endogenous RCS in situ. The labeling efficiency was normalized to the intensity of acetaldehyde in the lysate, and the labeled products were searched across previous labeling results (Figures 2e and 5e). The labeling of FA, acetaldehyde, glycolaldehyde, and GA were detected (Figures S25b and S26). The reactivity of **SpePPh**_**3**_**-yne** seemed moderate compared with that of **IbrPPh**_**3**_**-yne**.

Finally, the cytotoxicity of various Spe analogs, including Spe, **SpePPh**_**3**_**+FA**, **SpePPh**_**3**_, and **SpePPh**_**3**_**+RCS**, was also assessed. All four IC_50_ values ranged from 2.36 to 9.87 μM (Figure S25c). Here, the trend was consistent with that of the generated Ibr analogs. More interestingly, **SpePPh**_**3**_ (+endogenous RCS) and **SpePPh**_**3**_**+RCS** presented lower IC_50_ values (2.96 and 2.36 μM), again suggesting that mixtures of Spe analogs as inhibitor cocktails were beneficial.

Notably, our clickable probe **SpePPh**_**3**_**-yne** also demonstrated better cytotoxicity (0.88 μM), indicating that endogenous RCS was also enough to generate Spe analogs in situ (Figure S24b). The propargyl ether at the aminophenoxy moiety might enhance its cytotoxicity. Additionally, the IC_50_ value of **SpeCH**_**3**_, the acetamido side product obtained through triphenylphosphonium hydrolysis, was as low as 0.81 μM (Figures S24c and S27). Nevertheless, the structure–activity relationship of Spe has not yet been conducted thoroughly. Thus, various modifications, such as substituents around acrylamide and/or aminophenoxy moieties, could improve the cytotoxicity of Spe.

## Conclusions

We utilized stabilized triphenylphosphonium groups as reactive aqueous Wittig reagents for chemoselective probes and demonstrated their selectivity toward RCS. We applied native and deuterated chemoselective probes, **H/D-Tz-PPh**_**3**_**(1/3)**, whose distinct light/heavy isotope MS signatures allowed for easy identification of labeled species. By tagging through the clickable handle, we applied these chemoselective probes for in vitro and ex vivo labeling and successfully captured and characterized several cellular carbonyl species. In Ramos cells, some captured species are related to lipid peroxidation and AGEs.^22−24^ More interestingly, through our relative quantification metabolome analysis of cancerous and noncancerous cells, our results strongly suggest that RCS can potentially serve as therapeutic triggers to activate prodrugs. Therefore, we synthesized TCI precursors, **IbrPPh**_**3**_**(8)** and **SpePPh**_**3**_**(9)**, and their corresponding clickable precursors. By utilizing these precursors and a clickable RCS, **AldN**_**3**_**(16)**, we confirmed that TCI precursors can regenerate acrylamide warheads in situ and covalently bind to the target BTK. We also demonstrated that the endogenous RCS are sufficient to generate both Ibr and Spe analogs for BTK binding.

Finally, we observed differences in cytotoxicity among TCI analogs generated by reacting with different sources of RCS. We attributed these RCS-induced cytotoxicity alterations to the variations in the **R** groups adjacent to the acrylamide warheads (Figure 4b), which are associated with specific aldehydes when reacting with triphenylphosphonium precursors. In particular, when exogenous or endogenous RCS were used, in situ-regenerated TCI analogs showed significantly better cytotoxicity, suggesting that mixtures of analogs might be beneficial as inhibitor cocktails.

Our triphenylphosphonium probes can clearly react with RCS via the aqueous Wittig reaction. Notably, triphenylphosphonium-based probes have been used to target protein sulfenic acids.^15^ Some of our triphenylphosphonium probes might be consumed by protein sulfenic acids. Nevertheless, probes used in experiments should be sufficient for RCS labeling and/or TCI triggering. In addition, during sulfenic acid labeling, triphenylphosphonium moieties remained intact and presumably available for RCS labeling (Figure S28). Conventionally, aminooxy groups provide high and broad reactivity toward carbonyl labeling and therapeutic conjugate assembly.^8^ In contrast, our stabilized triphenylphosphonium probes and precursors exhibited moderate reactivity but great selectivity toward RCS. Since RCS are promising disease biomarkers, these reactivity features allowed us to label and compare RCS levels within various cell types and to specifically trigger in situ TCI analog generation.

Additionally, the hydrolysis of triphenylphosphonium groups seems inevitable, especially at low concentrations of aldehydes. At first glance, the detection limit of RCS metabolites in cells may not be as low as that of aminooxy probes, partly because of hydrolysis. Notably, hydrolysis differed among the molecules (Figures S29 and S30), and highly stable precursors, such as **IbrPPh**_**3**_**(8)** and **IbrPPh**_**3**_**-yne (10)**, were more efficient at labeling both metabolites and proteins. As inhibitor cocktails, the partially hydrolyzed TCI precursors were still inhibitory in a noncovalent fashion. Nevertheless, further investigations of the chemical structures of triphenylphosphonium probes should be able to increase their stability and optimize their labeling efficiency.

Recently, bioorthogonal reactions have been extensively utilized for readily generating bioactive conjugates.^35^ However, most click linkages, such as triazoles from the CuAAC, cannot be easily further functionalized. In contrast, our results showed that the aqueous Wittig reaction enables conjugation between stabilized phosphoniums and aldehydes. Moreover, the in situ formation of α,β-unsaturated carbonyl moieties can serve as TCI warheads or Michael acceptors for further functionalization. In addition, the reaction is compatible with other click reactions. For example, the double-clickable ibrutinib derivative **Ibr-AAM-N**_**3**_**-yne** can be easily synthesized via the aqueous Wittig reaction (Figure S21) and can attach different functional moieties through sequential click reactions. Given its efficiency, biocompatibility and functionalizability, we believe that the aqueous Wittig reaction can serve as a promising linking strategy for biological applications. We also envision that investigation and optimization of various stabilizing EWGs (ketones, nitriles, etc.) and triphenylphosphonium surrogates (phosphonates, phosphine oxides, etc.) will further expand the scope of the aqueous Wittig reaction.

## Supporting Information Available

Reagents, instruments, and methods; full synthetic and characterization details; and additional spectral and in vitro data (PDF)
