# Supplementary material for: Chemoselective Stabilized Triphenylphosphonium Probes for Capturing Reactive Carbonyl Species and Regenerating Covalent Inhibitors with Acrylamide Warheads in Cellulo
Source: J Am Chem Soc. 2024 Dec 27;147(2):1518–28. doi: 10.1021/jacs.4c09727 (PMC11744745; doi:10.1021/jacs.4c09727)
Supplement: Supplementary file 1 — ja4c09727_si_001.pdf [file ja4c09727_si_001.pdf]

Supporting Information

Chemoselective Stabilized Triphenylphosphonium Probes for Capturing Reactive Carbonyl Species and Regenerating Covalent Inhibitors with Acrylamide Warheads *in Cellulo*

Ai-Lin Chen[a], Zih-Jheng Lin[a], Hsiao-Yu Chang[a], Tsung-Shing Andrew Wang[a, *]

[a]*Department of Chemistry and Center for Emerging Material and Advanced Devices, National Taiwan University Taipei, 106319, Taiwan (R.O.C.)*

**Corresponding author: wangts@ntu.edu.tw.*

Table of Contents

1. General Synthetic Methods and Instrumentation S2
- 1.1. Synthetic materials S2
- 1.2. Instrumentation S2

2. Synthesis and Characterization of Compounds S2

3. Biological Materials and Instrumentation S23
- 3.1 Chemicals, critical commercial assays, and antibodies S23
- 3.2 Instrumentation S23

4. Chemoselective Labeling of Model Carbonyls and Metabolites S24
- 4.1 Labeling experiments of model carbonyls using triphenylphosphonium probes S24
- 4.2 Labeling experiments of a carbonyl mixture using triphenylphosphonium probes S24
- 4.3 Labeling experiments of metabolites using triphenylphosphonium probes S24
- 4.4 Labeling experiments of model carbonyls using aminooxy probes S25
- 4.5 Labeling experiments of metabolites using aminooxy probes S25
- 4.6 Experimental methods for the click reaction when labeling metabolites S25
- 4.7 Quantitative methods of relative reactivity in heatmaps S25

5. Sample Preparations, Purifications and Analysis Methods for Metabolome Experiments S26
- 5.1 Cell culture S26
- 5.2 Preparations of samples for metabolite labeling experiments S26
- 5.3 Biotin-streptavidin affinity purification S26
- 5.4 Liquid-liquid extraction for desalting S27
- 5.5 Estimation methods for RCS relative quantification S27

6. TCI Generation Experiments S27
- 6.1 Methods for clickable TCI generation experiments S27
- 6.2 Methods for double-clickable ibrutinib derivative experiments S28
- 6.3 Methods for ROS modulation experiments S28
- 6.4 Western blotting S29

7. Cytotoxicity Experiments with the AlamarBlue Cell Viability Assay S29

8. Protocols and Analysis Methods for Proteome Experiments S29
- 8.1 Cell treatment S29
- 8.2 Biotin-streptavidin affinity purification S30
- 8.3 LC-MS/MS analysis S30
- 8.4 MS data analysis and peptide identification S31

9. Stability Evaluation of Triphenylphosphonium Probes S31

10. Supplementary Schemes, Figures and Tables S32

11. References S68

12. NMR Spectra S70

1. General Synthetic Methods and Instrumentation

1.1. Synthetic materials

The chemicals and solvents were purchased from Echo Chemicals, Merck, Sigma-Aldrich, Alfa Aesar, Acros, NOVA Chemicals, AK Scientific, Showa Chemical Industry, Thermo Fisher Scientific, Combi-Blocks and Tokyo Chemical Industry. All reagents were reagent grade and used without further purification unless mentioned otherwise. The solvents were anhydrous or dried in the solvent purification system (LC Technology Solutions Inc.) using molecular sieves. Thin layer chromatography (TLC) was performed on 0.25 mm silica gel 60 F254 glass plates (Merck), where the compounds were visualized by a UV lamp or visualizing agents. Flash column chromatography purifications were performed using silica gel 60 (0.040-0.063 mm particle sizes) (Merck).

1.2. Instrumentation

NMR spectra were recorded on Agilent Unity Plus-400 MHz, Bruker AVIII-400 MHz (^{1}H: 400 MHz, ^{13}C: 100 MHz), Bruker AVIII-500 MHz (^{1}H: 500 MHz, ^{13}C: 125 MHz) or Bruker AVIII-800 MHz (^{1}H: 800 MHz, ^{13}C: 200 MHz) NMR spectrometers. The chemical shift (δ) was recorded in parts per million (ppm) using the residual non-deuterated solvents as references. The coupling constants (J) are expressed in hertz (Hz). The splitting patterns are reported as s (singlet), d (doublet), t (triplet), q (quartet), m (multiplet) and br (broad). All HPLC experiments were performed on an Agilent 1260 HPLC system, an Agilent 1100 Series HPLC system or a Waters 1525 binary pump machine with a YMC-Triart C18 column (5 µm, 10 i.d. × 250 mm or 5 µm, 4.6 i.d. × 250 mm).

2. Synthesis and Characterization of Compounds

Compound 17

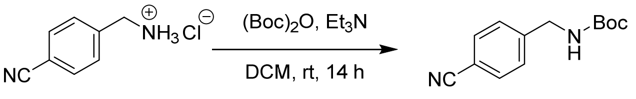

4-(Aminomethyl)benzonitrile hydrochloride (1.0 g, 5.9 mmol, 1 equiv.) was dissolved in DCM (20 mL), and Et_3N (2 mL, 14.8 mmol, 2.5 equiv.) and Boc_2O (1.4 g, 6.5 mmol, 1.1 equiv.) were added. The reaction solution was stirred at rt for 14 h under N_2. After the reaction was complete, the mixture was extracted with DCM and 10% HCl $_{(aq)}$. The organic layer was dried over $MgSO_4$ and evaporated to dryness to afford **17** (1.2 g, 85%) as a white solid. The analytical data of **17** are in agreement with the literature data[1]. **HR-MS (ESI)** calcd. for $C_{13}H_{17}N_2O_2^+$ $[M+H]^+$ 233.1285, found 233.1218. **^{1}H NMR (400 MHz, $CDCl_3$)** δ 7.62 (d, *J*=8.3 Hz, 2H), 7.38 (d, *J*=7.9 Hz, 2H), 4.97 (s, 1H), 4.37 (d, *J*=6.3 Hz, 2H), 1.46 (s, 9H). **^{13}C NMR (100 MHz, $CDCl_3$)** δ 155.84, 144.61, 132.40, 127.77, 118.76, 111.11, 80.06, 44.19, 28.33.

Compound 18

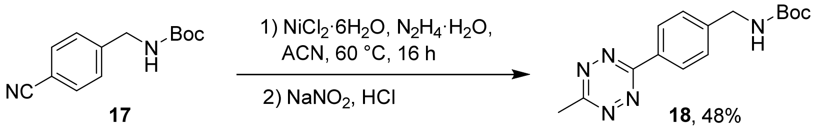

The synthesis of **17** was reported in the previous literature[1]. **17** (1.5 g, 6.5 mmol, 1 equiv.) was dissolved in ACN (3.4 mL, 64.6 mmol, 10 equiv.) and hydrazine (16 mL, 325 mmol, 50 equiv.). After

$NiCl_2 \cdot 6H_2O$ (418 mg, 3.2 mmol, 0.5 equiv.) was added, the reaction mixture was stirred at 60 °C for 16 h under N_2. Then, 5 M $NaNO_{2(aq)}$ (30 mL) was carefully added, and 10% $HCl_{(aq)}$ was added until gas evolution ceased. After the reaction was complete, the solution was extracted with EtOAc, and the organic layer was dried over $MgSO_4$ and concentrated under reduced pressure. The crude product was purified by column chromatography (EtOAc/Hex=1/4, v/v) to afford **18** (934 mg, 48%) as a pink solid. The analytical data of **18** are in agreement with the literature data[2]. **HR-MS (ESI)** calcd. for $C_{15}H_{20}N_5O_2^+$ $[M+H]^+$ 302.1612, found 302.1542. **^{1}H NMR (400 MHz, CDCl$_3$)** δ 8.55 (d, *J*=8.0 Hz, 2H), 7.50 (d, *J*=8.1 Hz, 2H), 4.96 (s, 1H), 4.43 (d, *J*=6.1 Hz, 2H), 3.09 (s, 3H), 1.48 (s, 9H). **^{13}C NMR (100 MHz, CDCl$_3$)** δ 167.21, 163.91, 155.91, 143.93, 130.79, 128.20, 128.05, 79.85, 44.38, 28.39, 21.14.

Compound 19

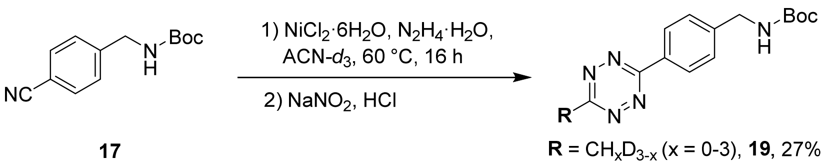

The synthesis of **17** was reported in the previous literature[1]. **17** (1.5 g, 6.5 mmol, 1 equiv.) was dissolved in ACN-*d*$_3$ (3.4 mL, 64.6 mmol, 10 equiv.) and hydrazine (16 mL, 325 mmol, 50 equiv.). After $NiCl_2 \cdot 6H_2O$ (418 mg, 3.2 mmol, 0.5 equiv.) was added, the reaction mixture was stirred at 60 °C for 16 h under N_2. Then, 5 M $NaNO_{2(aq)}$ (30 mL) was carefully added, and 10% $HCl_{(aq)}$ was added until gas evolution ceased. After the reaction was complete, the solution was extracted with EtOAc, and the organic layer was dried over $MgSO_4$ and concentrated under reduced pressure. The crude product was purified by column chromatography (EtOAc/Hex=1/4, v/v) to afford crude compound **19** (525 mg, 27%) as a pink solid. The isotope ratio of **19** was determined by HR-MS. **HR-MS (ESI)** calcd. for $C_{15}H_{20}N_5O_2^+$, $C_{15}H_{19}DN_5O_2^+$, $C_{15}H_{18}D_2N_5O_2^+$, and $C_{15}H_{17}D_3N_5O_2^+$; $[M+H]^+$ 302.1612, 303.1675, 304.1737, and 305.1800; found 302.1565 (8.8%), 303.1624 (19.2%), 304.1682 (32.2%), and 305.1741 (39.8%) **(Figure S4)**. **^{1}H NMR (400 MHz, CDCl$_3$)** δ 8.55 (d, *J*=8.5 Hz, 2H), 7.50 (d, *J*=7.9 Hz, 2H), 4.97 (s, 1H), 4.43 (d, *J*=6.1 Hz, 2H), 3.14 – 3.05 (m, 1H), 1.48 (s, 9H). **^{13}C NMR (100 MHz, CDCl$_3$)** δ 167.17, 163.93, 155.91, 143.94, 130.79, 128.19, 128.04, 79.83, 44.38, 28.39, 22.86 – 18.23 (m).

Compound 1: Tz-PPh$_3$

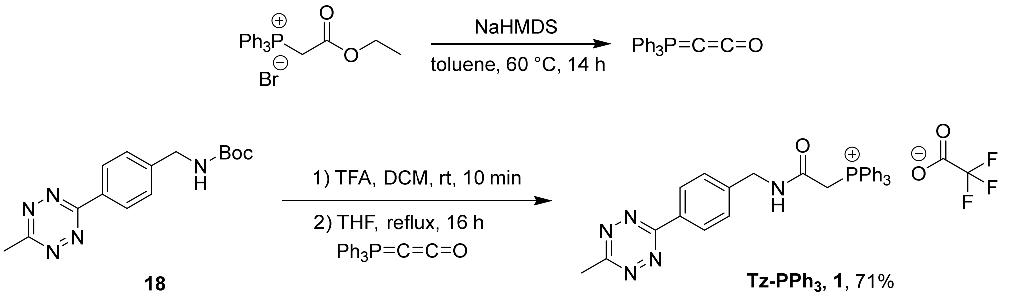

The synthesis of ketenylidenetriphenylphosphorane was reported in previous literature[3]. A solution of (2-ethoxy-2-oxoethyl)triphenylphosphonium bromide (500 mg, 1.4 mmol, 1 equiv.) dissolved in anhydrous toluene (6.5 mL) was added dropwise to a solution of sodium hexamethyl-disilazide (1.7 mL, 1 M solution in THF, 1.7 mmol, 1.2 equiv.) at 0 °C. The reaction mixture was then stirred at 60 °C for 14 h under N_2. After the mixture was cooled to rt, the precipitate was removed by

filtration. The filtrate was concentrated under reduced pressure, and Et_2O (5 mL) was added. The mixture was cooled in an ice bath. The formed precipitate was filtered and washed with Et_2O to afford the desired product (240 mg, 55%) as a colorless solid, and was used without further purification.

The synthesis of **18** was reported in the previous literature[2]. Compound **18** (10.0 mg, 0.033 mmol, 1 equiv.) was dissolved in anhydrous DCM (330 µL), and TFA was added (330 µL). After stirred at rt for 10 min, the solvent was evaporated *in vacuo*. The mixture was then dissolved in anhydrous THF (330 µL), and freshly prepared ketenylidenetriphenylphosphorane (10.9 mg, 0.036 mmol, 1.1 equiv.) was added. The reaction mixture was stirred and refluxed for 16 h under N_2. After the reaction was complete, the crude product was purified by semi-preparative RP-HPLC (H_2O+0.1% TFA/ACN+0.1% TFA=80/20 over 6 min, 80/20 to 0/100 over 12 min, 0/100 over 6 min, flow rate=3 mL/min, retention time: 14 min), affording **1** (14.5 mg, 71%) as a pink solid. **HR-MS (ESI)** calcd. for $C_{30}H_{27}N_5OP^+$ $[M]^+$ 504.1948, found 504.2017. **^{1}H NMR (400 MHz, DMSO-*d*$_6$)** δ 9.11 (t, *J*=5.9 Hz, 1H), 8.37 (d, *J*=8.3 Hz, 2H), 7.91 – 7.85 (m, 3H), 7.84 – 7.79 (m, 4H), 7.78 – 7.72 (m, 8H), 7.33 (d, *J*=8.2 Hz, 2H), 5.04 (d, *J*=14.8 Hz, 2H), 4.35 (d, *J*=5.8 Hz, 2H), 3.01 (s, 3H). **^{13}C NMR (100 MHz, DMSO-*d*$_6$)** δ 167.14, 163.11, 157.68, 142.85, 134.85 (d, J_{PC}=3.0 Hz), 133.75 (d, J_{PC}=10.7 Hz), 130.65, 130.00 (d, J_{PC}=12.8 Hz), 128.11, 127.42, 118.82 (d, J_{PC}=88.6 Hz), 42.54, 31.31 (d, J_{PC}=58.1 Hz), 20.84. **^{31}P NMR (162 MHz, DMSO-*d*$_6$)** δ 22.53.

Compound 3: D-Tz-PPh$_3$

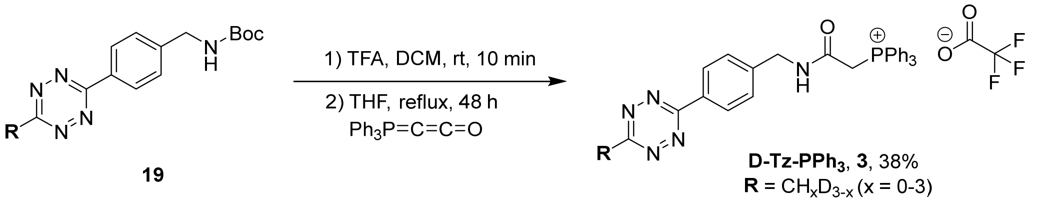

Crude **19** (10.0 mg, 0.033 mmol, 1 equiv.) was dissolved in anhydrous DCM (330 µL), and TFA was added (330 µL). After stirred at rt for 10 min, the solvent was evaporated *in vacuo*. The mixture was then dissolved in anhydrous THF (330 µL), and freshly prepared ketenylidenetriphenylphosphorane (10.9 mg, 0.036 mmol, 1.1 equiv.) was added. The reaction mixture was stirred and refluxed for 48 h under N_2. After the reaction was complete, the crude product was purified by semi-preparative RP-HPLC (H_2O+0.1% TFA/ACN+0.1% TFA=80/20 over 6 min, 80/20 to 0/100 over 12 min, 0/100 over 6 min, flow rate=3 mL/min, retention time: 14 min), affording crude compound **3** (7.8 mg, 38%) as a pink solid. The isotope ratio of **3** was determined by HR-MS. **HR-MS (ESI)** calcd. for $C_{30}H_{27}N_5OP^+$, $C_{30}H_{26}DN_5OP^+$, $C_{30}H_{25}D_2N_5OP^+$, and $C_{30}H_{24}D_3N_5OP^+$; $[M]^+$ 504.1948, 505.2011, 506.2073, and 507.2136; found 504.1940 (8.7%), 505.1998 (23.4%), 506.2059 (33.9%), and 507.2118 (34.0%) **(Figure S4)**. **^{1}H NMR (400 MHz, DMSO-*d*$_6$)** δ 9.09 (t, *J*=5.9 Hz, 1H), 8.36 (d, *J*=8.3 Hz, 2H), 7.90 – 7.84 (m, 3H), 7.83 – 7.78 (m, 4H), 7.77 – 7.72 (m, 8H), 7.33 (d, *J*=8.2 Hz, 2H), 5.03 (d, *J*=14.9 Hz, 2H), 4.34 (d, *J*=5.9 Hz, 2H), 3.03 – 2.95 (m, 1H). **^{13}C NMR (100 MHz, DMSO-*d*$_6$)** δ 167.09, 163.19 – 162.91 (m), 157.89, 142.86, 134.86, 134.08 – 132.72 (m), 130.67, 130.25 – 128.77 (m), 128.17, 128.08, 127.53, 127.33, 118.84 (d, J_{PC}=88.7 Hz), 42.55, 31.32 (d, J_{PC}=58.4 Hz), 21.17 – 20.00 (m). **^{31}P NMR (162 MHz, DMSO-*d*$_6$)** δ 22.41.

Compound 20

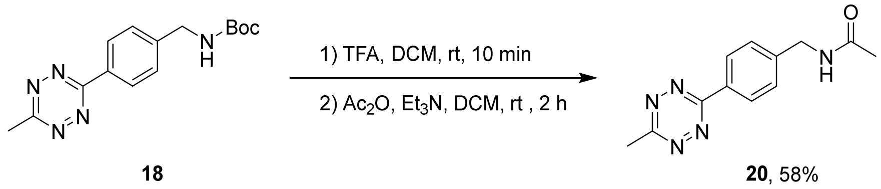

The synthesis of **18** was reported in the previous literature[2]. Compound **18** (25.0 mg, 0.083 mmol, 1 equiv.) was dissolved in anhydrous DCM (825 µL), and TFA was added (825 µL). After stirred at rt for 10 min, the solvent was evaporated *in vacuo*. The mixture was then dissolved in anhydrous DCM (400 µL), and Et_3N (17 µL, 0.12 mmol, 1.5 equiv.) and Ac_2O (9 µL, 0.099 mmol, 1.2 equiv.) were added at 0 °C. The reaction solution was stirred at rt for 2 h under N_2. After the reaction was complete, the mixture was extracted with H_2O. The organic layer was dried over $MgSO_4$ and evaporated to dryness to afford **20** (11.7 mg, 58%) as a pink solid. The analytical data of **20** are in agreement with the literature data[4]. **HR-MS (ESI)** calcd. for $C_{12}H_{14}N_5O^+$ $[M+H]^+$ 244.1193, found 244.1193. **^{1}H NMR (400 MHz, CDCl$_3$)** δ 8.54 (d, *J*=8.4 Hz, 2H), 7.49 (d, *J*=8.6 Hz, 2H), 5.94 (s, 1H), 4.55 (d, *J*=5.9 Hz, 2H), 3.09 (s, 3H), 2.08 (s, 3H). **^{13}C NMR (100 MHz, CDCl$_3$)** δ 170.09, 167.27, 163.82, 143.14, 130.98, 128.43, 128.26, 43.36, 23.28, 21.15.

Compound 21

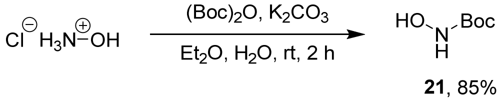

Hydroxylamine hydrochloride (240 mg, 3.4 mmol, 1 equiv.) and K_2CO_3 (475 mg, 3.4 mmol, 1 equiv.) were suspended in Et_2O (5 mL) and H_2O (0.6 mL). The reaction mixture was stirred at rt for 1 h. Then, a solution of $(Boc)_2O$ (500 mg, 2.3 mmol, 0.7 equiv.) dissolved in Et_2O (5 mL) was added dropwise to the stirring suspension at 0 °C. The reaction mixture was stirred at rt for 2 h under N_2. After the reaction was complete, H_2O was added to the mixture until the two layers were separated. The aqueous layer was extracted with Et_2O, and the combined organic layer was dried over Na_2SO_4 and concentrated under reduced pressure. The crude product was purified by column chromatography (EtOAc/Hex=1/2, v/v) to afford **21** (259 mg, 85%) as a colorless solid. The analytical data of **21** are in agreement with the literature[5]. **^{1}H NMR (400 MHz, DMSO-*d*$_6$)** δ 9.26 (s, 1H), 8.48 (s, 1H), 1.39 (s, 9H). **^{13}C NMR (100 MHz, DMSO-*d*$_6$)** δ 157.23, 78.68, 28.18.

Compound 22

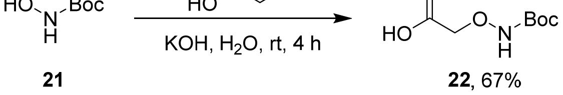

The synthesis of **21** was reported in the previous literature[5]. Compound **21** (100 mg, 0.75 mmol, 1 equiv.) and KOH (105 mg, 1.88 mmol, 2.5 equiv.) were suspended in H_2O (750 µL), and a solution of bromoacetic acid (130 mg, 0.94 mmol, 1.25 equiv.) dissolved in H_2O (375 µL) was added dropwise to the stirring suspension. The reaction mixture was stirred at rt for 4 h under N_2 and acidified to pH 7 with 10% $HCl_{(aq)}$. The solution was extracted with DCM to remove the residue of **21**. Then, the pH of

the solution was adjusted to 1 with 10% HCl $_{(aq)}$, and the solution was extracted with DCM. The organic layer was dried over Na_2SO_4 and evaporated *in vacuo* to afford **22** (96.2 mg, 67%) as a white solid. The analytical data of **22** are in agreement with literature data[6]. **HR-MS (ESI)** calcd. for $C_7H_{13}NO_5Na^+$ $[M+Na]^+$ 214.0686, found 214.0660. **^{1}H NMR (400 MHz, CDCl$_3$)** δ 7.87 (s, 1H), 4.49 (s, 2H), 1.51 (s, 9H). **^{13}C NMR (100 MHz, CDCl$_3$)** δ 171.04, 159.19, 84.74, 74.86, 28.04.

Compound 23

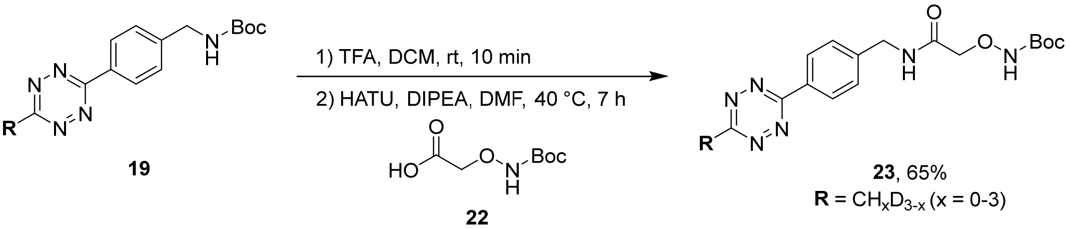

Crude **19** (18.0 mg, 0.060 mmol, 1 equiv.) was dissolved in anhydrous DCM (600 µL), and TFA was added (600 µL). After stirred at rt for 10 min, the solvent was evaporated *in vacuo*. The mixture was then dissolved in anhydrous DMF (1050 µL).

The synthesis of **22** was reported in the previous literature[6]. In another round-bottom flask, compound **22** (17.1 mg, 0.090 mmol, 1.5 equiv.) was dissolved in anhydrous DMF (370 µL), and HATU (40.9 mg, 0.11 mmol, 1.8 equiv.) dissolved in anhydrous DMF (260 µL) was added. The mixture was stirred for 10 min, and a DMF solution of crude **19** was added. The reaction mixture was stirred for 20 min, after which DIPEA (26 µL, 0.15 mmol, 2.5 equiv.) was added. The mixture was stirred at 40 °C for 7 h under N_2. After the reaction was complete, EtOAc (10 mL) was added to the mixture, which was subsequently extracted with H_2O. The organic layer was dried over $MgSO_4$ and concentrated under reduced pressure. The crude product was purified by column chromatography (EtOAc/Hex=3/1, v/v) to afford crude compound **23** (13.4 mg, 65%) as a pink solid. The isotope ratio of **23** was determined by HR-MS. **HR-MS (ESI)** calcd. for $C_{17}H_{22}N_6O_4Na^+$, $C_{17}H_{21}DN_6O_4Na^+$, $C_{17}H_{20}D_2N_6O_4Na^+$, and $C_{17}H_{19}D_3N_6O_4Na^+$; $[M+Na]^+$ 397.1595, 398.1658, 399.1720, and 400.1783; found 397.1567 (7.2%), 398.1639 (21.3%), 399.1699 (36.0%), and 400.1754 (35.5%) **(Figure S4)**. **^{1}H NMR (400 MHz, CDCl$_3$)** δ 8.78 (br, s, 1H), 8.53 (d, *J*=8.4 Hz, 2H), 7.64 (s, 1H), 7.53 (d, *J*=8.5 Hz, 2H), 4.62 (d, *J*=6.1 Hz, 2H), 4.41 (s, 2H), 3.11 – 3.03 (m, 1H), 1.41 (s, 9H). **^{13}C NMR (100 MHz, CDCl$_3$)** δ 169.00, 167.15, 163.94, 157.92, 143.12, 130.68, 128.41, 128.12, 83.42, 76.31, 42.59, 28.00, 22.64 – 18.42 (m).

Compound 4: D-Tz-ONH$_2$

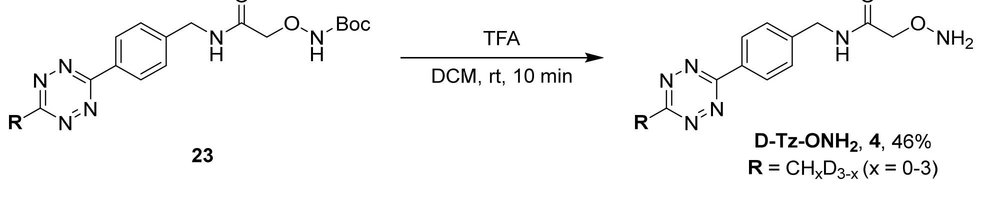

Crude **23** (13.4 mg, 0.036 mmol, 1 equiv.) was dissolved in anhydrous DCM (390 µL), and TFA was added (390 µL). After stirred at rt for 10 min, the solvent was evaporated *in vacuo*. The crude product was purified by semi-preparative RP-HPLC (H_2O/ACN=100/0 over 6 min, 100/0 to 0/100 over 8 min, 0/100 over 6 min, flow rate=3 mL/min, retention time: 15 min), affording crude compound **4** (7.8 mg, 46%) as a pink solid. The isotope ratio of **4** was determined by HR-MS. **HR-MS (ESI)** calcd. for

$C_{12}H_{15}N_6O_2^+$, $C_{12}H_{14}DN_6O_2^+$, $C_{12}H_{13}D_2N_6O_2^+$, and $C_{12}H_{12}D_3N_6O_2^+$; $[M+H]^+$ 275.1251, 276.1314, 277.1377, and 278.1440; found 275.1208 (8.1%), 276.1275 (20.5%), 277.1340 (36.2%), and 278.1395 (35.2%) **(Figure S4)**. **^{1}H NMR (400 MHz, DMSO-*d*$_6$)** δ 8.46 (t, *J*=6.2 Hz, 1H), 8.42 (d, *J*=8.4 Hz, 2H), 7.56 (d, *J*=8.5 Hz, 2H), 6.40 (s, 2H), 4.46 (d, *J*=6.2 Hz, 2H), 4.05 (s, 2H), 3.01 – 2.93 (m, 1H). **^{13}C NMR (100 MHz, DMSO-*d*$_6$)** δ 170.21, 167.05, 163.26, 144.38, 130.37, 128.06, 127.42, 74.48, 41.49, 21.28 – 19.90 (m).

Compound 5: TCO-SS-biotin

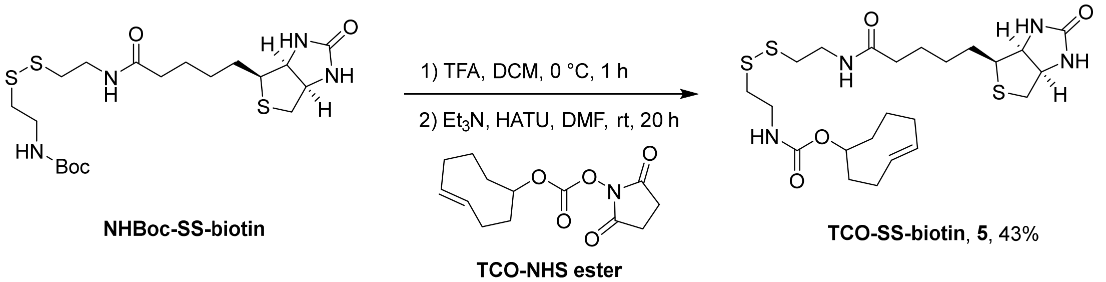

The synthesis of **NHBoc-SS-biotin** was reported in our previous literature[7]. Homemade **NHBoc-SS-biotin** (20.0 mg, 0.042 mmol, 1 equiv.) was dissolved in anhydrous DCM (400 µL), and TFA (400 µL) was added. The reaction mixture was stirred in an ice bath for 1 h, and the solvent was evaporated *in vacuo*. The mixture was then dissolved in anhydrous DMF (400 µL). After Et_3N (11.6 µL, 0.084 mmol, 2 equiv.) was added, the solution was stirred for 10 min, followed by the addition of HATU (23.8 mg, 0.063 mmol, 1.5 equiv.) and TCO-NHS ester (22.3 mg, 0.084 mmol, 2 equiv.) in the dark. Then, the reaction mixture was stirred in the dark at rt for 20 h under N_2. After the reaction was complete, the mixture was concentrated under reduced pressure. The crude product was purified by semi-preparative RP-HPLC (H_2O/ACN=100/0 over 4 min, 100/0 to 0/100 over 12 min, 0/100 over 4 min, flow rate=3 mL/min, retention time: 17.5 min) in the dark, affording **5** (9.5 mg, 43%) as a white solid. **HR-MS (ESI)** calcd. for $C_{23}H_{39}N_4O_4S_3^+$ $[M+H]^+$ 531.2128, found 531.2214. **^{1}H NMR (400 MHz, DMSO-*d*$_6$)** δ 7.97 (q, *J*=5.4 Hz, 1H), 7.28 (q, *J*=5.6 Hz, 1H), 6.39 (s, 1H), 6.33 (s, 1H), 5.63 (ddd, *J*=14.5, 10.9, 3.4 Hz, 1H), 5.52 (ddd, *J*=14.5, 10.8, 3.5 Hz, 1H), 4.73 (dd, *J*=10.3, 5.3 Hz, 1H), 4.31 (ddd, *J*=7.4, 5.2, 0.9 Hz, 1H), 4.13 (ddt, *J*=7.9, 4.5, 1.6 Hz, 1H), 3.31 – 3.24 (m, 4H), 3.10 (dtd, *J*=9.1, 4.7, 2.5 Hz, 1H), 2.86 – 2.72 (m, 5H), 2.58 (d, *J*=12.4 Hz, 1H), 2.26 (qd, *J*=11.6, 4.5 Hz, 1H), 2.19 – 2.08 (m, 3H), 2.08 (t, *J*=7.2 Hz, 2H), 2.04 – 1.98 (m, 1H), 1.80 (qd, *J*=11.2, 4.7 Hz, 1H), 1.71 – 1.56 (m, 3H), 1.56 – 1.39 (m, 4H), 1.35 – 1.25 (m, 2H), 1.23 – 1.13 (m, 1H). **^{13}C NMR (100 MHz, DMSO-*d*$_6$)** δ 172.25, 162.72, 155.78, 134.99, 131.51, 68.55, 61.04, 59.21, 55.42, 40.44, 39.86, 37.89, 37.61, 37.33, 35.15, 33.90, 32.15, 29.59, 28.19, 28.03, 27.44, 25.23.

Compound 24

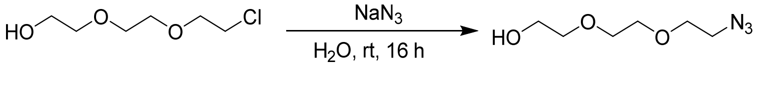

2-(2-(2-Chloroethoxy)ethoxy)ethanol (2.0 g, 11.9 mmol, 1 equiv.) and sodium azide (1.5 g, 23.7 mmol, 2 equiv.) were dissolved in H_2O (5 mL). The reaction solution was stirred at rt for 16 h under N_2. After the reaction was complete, the mixture was extracted with DCM. The organic layer was dried over $MgSO_4$ and evaporated *in vacuo* to afford **24** as a colorless oil (1.7 g, 82%). The analytical data of **24** are in agreement with the literature[8]. **^{1}H NMR (400 MHz, $CDCl_3$)** δ 3.74 – 3.69 (m, 2H),

3.70 – 3.66 (m, 6H), 3.64 – 3.59 (m, 2H), 3.40 (t, *J*=5.0 Hz, 2H), 2.29 (s, 1H). **^{13}C NMR (100 MHz, CDCl$_3$)** δ 72.46, 70.62, 70.35, 70.01, 61.73, 50.62.

Compound 25

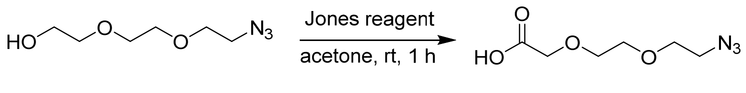

24 **25**, 52%

The synthesis of **24** was reported in the previous literature[8]. Compound **24** (1.7 g, 9.7 mmol, 1 equiv.) was dissolved in acetone (98 mL) and cooled to 0 °C. Then, freshly prepared 3 M Jones reagent was added dropwise, and the resulting reaction mixture was stirred at rt for 1 h. The reaction was then quenched by the addition of propan-2-ol (4.4 mL). After 15 min, acetone (100 mL) was added, and the green precipitate was removed by filtration over Celite. The filtrate was evaporated *in vacuo* to afford **25** (955 mg, 52%) as a yellow oil.

To prepare 3 M Jones reagent, 2.9 g CrO_3 was first dissolved in 4.9 mL H_2O, and 2.4 mL H_2SO_4 was added to the stirring solution in an ice bath. Then, 2.4 mL H_2O was added to the solution, and the mixture was stirred until the solid was thoroughly dissolved.

The analytical data of **25** are in agreement with the literature[9]. **^{1}H NMR (400 MHz, CDCl$_3$)** δ 9.66 (br, s, 1H), 4.16 (s, 2H), 3.74 – 3.69 (m, 2H), 3.68 – 3.66 (m, 2H), 3.64 (dd, *J*=4.9, 3.8 Hz, 2H), 3.37 (t, *J*=5.0 Hz, 2H). **^{13}C NMR (100 MHz, CDCl$_3$)** δ 174.50, 70.89, 70.34, 69.85, 68.16, 50.41.

Compound 15: azido-SS-biotin

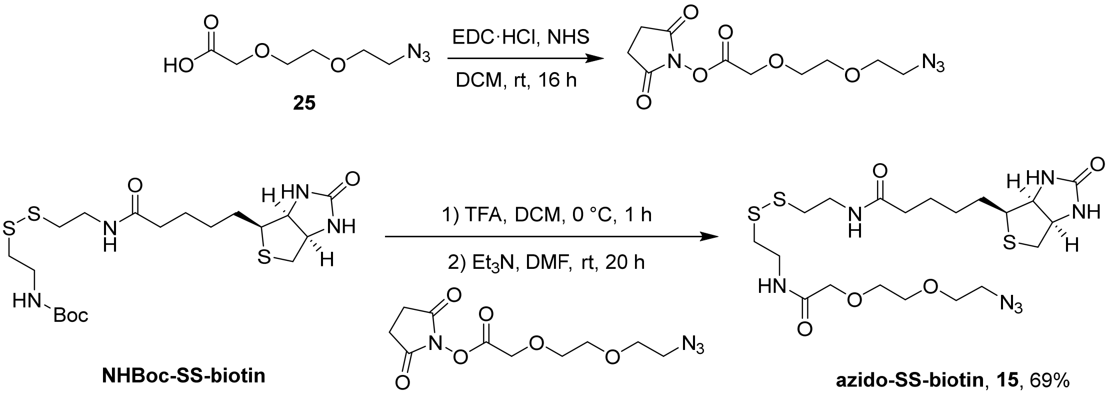

25

NHBoc-SS-biotin

azido-SS-biotin, **15**, 69%

The synthesis of **25** was reported in the previous literature[9]. Compound **25** (50.0 mg, 0.26 mmol, 1 equiv.) was dissolved in anhydrous DCM (265 µL), and NHS (36.5 mg, 0.32 mmol, 1.2 equiv.) was added. Then, EDC·HCl (60.8 mg, 0.32 mmol, 1.2 equiv.) dissolved in anhydrous DCM (265 µL) was added to the above solution in an ice bath. The reaction mixture was stirred at rt for 16 h under N_2 and concentrated *in vacuo* to afford the NHS-activated compound **25**.

Homemade **NHBoc-SS-biotin** (7.5 mg, 0.016 mmol, 1 eq) was dissolved in anhydrous DCM (300 µL), and TFA (300 µL) was added. The reaction mixture was stirred in an ice bath for 1 h, and the solvent was evaporated *in vacuo*. The mixture was then dissolved in anhydrous DMF (300 µL). After Et_3N (6.6 µL, 0.047 mmol, 3 eq) was added, the solution was stirred for 10 min, followed by the addition of freshly prepared NHS-activated compound **25**. Then, the reaction mixture was stirred at rt for 20 h under N_2 and concentrated under reduced pressure. The crude product was purified by semi-preparative RP-HPLC (H_2O/MeOH=100/0 over 6 min, 100/0 to 0/100 over 8 min, 0/100 over 6

min, flow rate=3 mL/min, retention time: 16.7 min), affording **15** (5.9 mg, 69%) as a white solid. **HR-MS (ESI)** calcd. for $C_{20}H_{36}N_7O_5S_3^+$ [M+H]$^+$ 550.1935, found 550.1984. **^{1}H NMR (400 MHz, DMSO-*d*$_6$)** δ 7.97 (q, *J*=5.6 Hz, 1H), 7.82 (t, *J*=5.7 Hz, 1H), 6.40 (s, 1H), 6.34 (s, 1H), 4.28 (dd, *J*=7.7, 5.2 Hz, 1H), 4.11 (dt, *J*=8.0, 2.3 Hz, 1H), 3.87 (s, 2H), 3.64 – 3.59 (m, 6H), 3.42 – 3.37 (m, 4H), 3.30 – 3.26 (m, 1H), 3.07 (dt, *J*=6.2, 1.7 Hz, 1H), 2.83 – 2.76 (m, 3H), 2.74 (t, *J*=6.7 Hz, 2H), 2.55 (d, *J*=12.4 Hz, 1H), 2.05 (t, *J*=7.4 Hz, 2H), 1.62 – 1.56 (m, 1H), 1.54 – 1.38 (m, 3H), 1.35 – 1.22 (m, 2H). **^{1}H NMR (400 MHz, MeOH-*d*$_4$)** δ 4.49 (ddd, *J*=7.9, 5.0, 1.0 Hz, 1H), 4.31 (dd, *J*=7.9, 4.4 Hz, 1H), 4.05 – 3.97 (m, 2H), 3.72 – 3.65 (m, 6H), 3.58 (td, *J*=6.8, 1.7 Hz, 2H), 3.49 (td, *J*=6.4, 2.1 Hz, 2H), 3.42 (dd, *J*=5.6, 4.3 Hz, 2H), 3.21 (ddd, *J*=8.7, 5.9, 4.4 Hz, 1H), 2.98 – 2.89 (m, 1H), 2.86 (q, *J*=7.0 Hz, 4H), 2.71 (d, *J*=12.7 Hz, 1H), 2.22 (t, *J*=7.6 Hz, 2H), 1.81 – 1.54 (m, 4H), 1.45 (p, *J*=7.6 Hz, 2H). **^{13}C NMR (100 MHz, MeOH-*d*$_4$)** δ 176.22, 172.89, 166.11, 72.03, 71.37, 71.28, 71.10, 63.37, 61.63, 57.00, 51.78, 41.05, 39.48, 39.16, 38.66, 38.33, 36.73, 29.74, 29.47, 26.82.

Compound 26

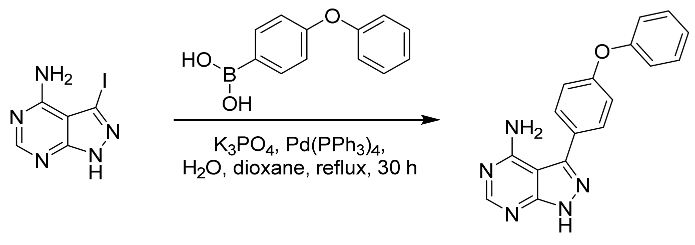

26, 65%

3-iodo-1*H*-pyrazolo[3,4-*d*]pyrimidin-4-amine (1.1 g, 4.0 mmol, 1 equiv.), 4-phenoxyphenylboronic acid (1.7 g, 8.0 mmol, 2 equiv.), and K_3PO_4 (2.5 g, 12.0 mmol, 3 equiv.) were dissolved in 1,4-dioxane/H_2O (4:1, 50 mL). The solvent was degassed using a stream of N_2 for 30 min, and $Pd(PPh_3)_4$ (270 mg, 0.20 mmol, 0.05 equiv.) was added. The reaction mixture was stirred and refluxed for 30 h under N_2, and was cooled to rt and filtered. The filtrate was washed with H_2O, MeOH and EtOH to afford compound **26** (765 mg, 65%) as a white solid. The analytical data of **26** are in agreement with the literature data[10]. **HR-MS (ESI)** calcd. for $C_{17}H_{14}N_5O^+$ [M+H]$^+$, 304.1192; found, 304.1155. **^{1}H NMR (400 MHz, DMSO-*d*$_6$)** δ 13.56 (s, 1H), 8.22 (s, 1H), 7.67 (d, *J*=8.5 Hz, 2H), 7.44 (t, *J*=7.9 Hz, 2H), 7.20 (t, *J*=7.2 Hz, 1H), 7.14 (t, *J*=8.4 Hz, 4H). **^{13}C NMR (100 MHz, DMSO-*d*$_6$)** δ 158.05, 156.97, 156.30, 156.13, 155.71, 143.82, 130.12, 130.00, 128.47, 123.77, 118.99, 118.91, 96.92.

Compound 27

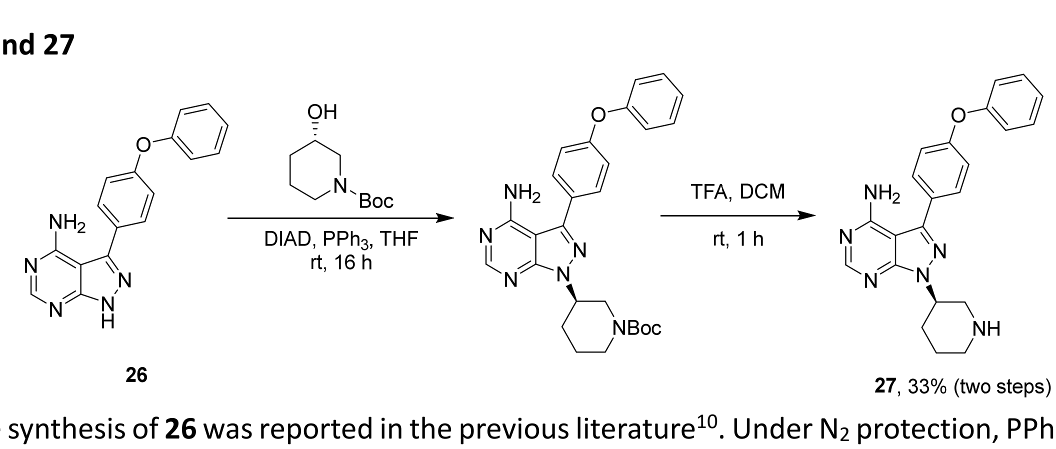

26

27, 33% (two steps)

The synthesis of **26** was reported in the previous literature[10]. Under N_2 protection, PPh_3 (195 mg, 0.75 mmol, 1.5 equiv.) and DIAD (145 μL, 0.75 mmol, 1.5 equiv.) dissolved in anhydrous THF were cooled to 0 °C and stirred for 30 min. Then, *tert*-butyl (*S*)-3-hydroxypiperidine-1-carboxylate (150 mg,

0.38 mmol, 0.75 equiv.) dissolved in anhydrous THF was added. After stirring for 30 min, compound **26** (150 mg, 0.5 mmol, 1 equiv.) dissolved in anhydrous THF was added. The mixture was stirred at rt for 16 h under N_2. After the reaction was complete, the reaction mixture was concentrated under reduced pressure. Then, anhydrous DCM (3 mL) and TFA (3 mL) were added. The reaction mixture was stirred at rt for 1 h, and the mixture was extracted with DCM and H_2O. Then, the aqueous layer was adjusted to pH=12 by sat. $NaHCO_{3(aq)}$, and the solution was extracted with DCM. The combined organic layer was dried over $MgSO_4$ and concentrated under reduced pressure. After evaporated *in vacuo*, compound **27** (63 mg, 33% over two steps) was obtained as a white solid. **HR-MS (ESI)** calcd. for $C_{22}H_{23}N_6O^+$ $[M+H]^+$, 387.1927; found, 387.2032. **^{1}H NMR (400 MHz, $CDCl_3$)** δ 8.23 (s, 1H), 7.61 (d, *J*=8.8 Hz, 2H), 7.38 (t, *J*=7.6 Hz, 2H), 7.19 – 7.07 (m, 5H), 5.17 – 5.10 (m, 1H), 3.60 – 3.49 (m, 2H), 3.33 – 3.27 (m, 1H), 3.09 – 2.99 (m, 1H), 2.33 – 2.16 (m, 2H), 1.97 – 1.92 (m, 2H). **^{13}C NMR (100 MHz, $CDCl_3$)** δ 158.9, 158.19, 156.3, 154.9, 153.9, 144.7, 130.1, 130.0, 127.2, 124.3, 119.7, 119.2, 98.5, 51.7, 47.2, 43.9, 29.2, 22.2.

Compound 28

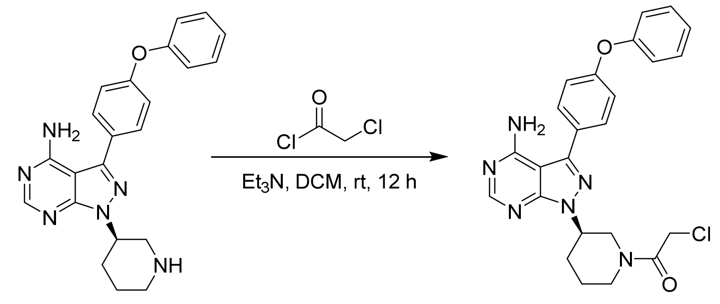

27 **28**, 51%

Compound **27** (40 mg, 0.1 mmol, 1 equiv.) was dissolved in DCM (1 mL), and Et_3N (14 µL, 0.1 mmol, 1 equiv.) was added. Chloroacetyl chloride (10 mg, 0.1 mmol, 1 equiv.) was then added slowly at 0 °C. After stirring at 0 °C for 1 h and then stirring at rt for 12 h under N_2, the reaction mixture was concentrated under reduced pressure. Then, the crude product was purified by column chromatography (MeOH/DCM=1/50, v/v) to afford compound **28** (24 mg, 51%) as a white solid. **HR-MS (ESI)** calcd. for $C_{24}H_{24}ClN_6O_2^+$ $[M+H]^+$, 463.1643; found, 463.1522. **^{1}H NMR (400 MHz, $CDCl_3$)** δ 8.34 (d, *J*=7.3 Hz, 1H), 7.62 (d, *J*=8.1 Hz, 2H), 7.37 (t, *J*=7.8 Hz, 2H), 7.17 – 7.12 (m, 3H), 7.07 (d, *J*=7.9 Hz, 2H), 5.61 (s, 2H), 4.98 – 4.82 (m, 1H), 4.73 (d, *J*=12.2 Hz, 0.5H), 4.52 – 4.38 (m, 0.5H), 4.14 – 4.02 (m, 2.5H), 3.90 – 3.79 (m, 1H), 3.40 (t, *J*=11.8 Hz, 0.5H), 3.24 (t, *J*=12.8 Hz, 0.5H), 2.92 (t, *J*=11.8 Hz, 0.5H), 2.45 – 2.19 (m, 2H), 2.02 – 1.90 (m, 1H), 1.87 – 1.66 (m, 1H). **^{13}C NMR (100 MHz, $CDCl_3$)** δ 165.38, 165.25, 158.68, 157.77, 157.70, 156.30, 155.79, 155.59, 154.32, 144.11, 144.02, 129.97, 129.91, 127.66, 127.47, 124.12, 124.05, 119.59, 119.55, 119.10, 53.13, 52.31, 50.33, 46.38, 46.12, 42.44, 41.14, 30.07, 29.82, 24.87, 23.67.

Compound 8: IbrPPh$_3$

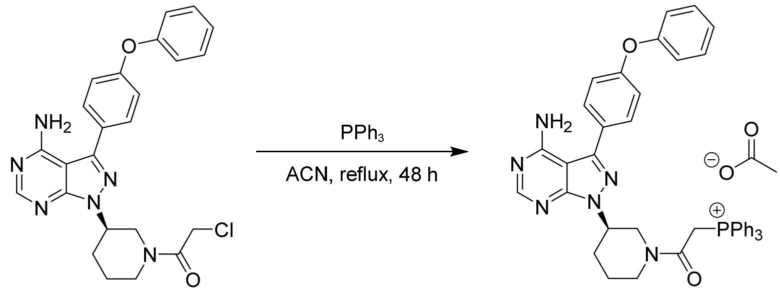

28 **IbrPPh$_3$**, **8**, 59%

Compound **28** (10 mg, 0.02 mmol, 1 equiv.) was dissolved in ACN, and PPh$_3$ (9 mg, 0.03 mmol, 1.5 equiv.) was added. The mixture was stirred and refluxed for 48 h under N$_2$. After cooling to rt, the mixture was concentrated under reduced pressure. The crude product was purified by semi-preparative RP-HPLC (H$_2$O/ACN+0.1% AcOH=100/0 over 6 min, 100/0 to 0/100 over 12 min, 0/100 over 6 min, flow rate=3 mL/min, retention time: 21 min), affording compound **8** (8 mg, 59%) as a white solid. **HR-MS (ESI)** calcd. for $C_{42}H_{38}N_6O_2P^+$ [M]$^+$, 689.2788; found 689.2759. **^{1}H NMR (400 MHz, DMSO-*d$_6$*)** δ 8.23 (s, 1H), 7.68 – 7.55 (m, 17H), 7.44 (tt, *J*=7.5, 2.2 Hz, 2H), 7.22 – 7.17 (m, 1H), 7.17 – 7.10 (m, 4H), 4.73 (tt, *J*=10.6, 4.1 Hz, 1H), 4.26 (d, *J*=10.3 Hz, 1H), 4.05 (d, *J*=13.0 Hz, 1H), 3.30 (s, 1H), 2.85 (t, *J*=12.2 Hz, 1H), 2.29 – 2.05 (m, 2H), 1.86 – 1.85 (m, 1H), 1.66 – 1.59 (m, 1H). **^{13}C NMR (100 MHz, DMSO-*d$_6$*)** δ 173.01, 158.52, 157.42, 156.68, 155.91, 154.29, 143.38, 133.20 (d, J_{PC}=9.9 Hz), 132.64, 131.84 (d, J_{PC}=9.5 Hz), 130.48, 130.42, 129.29 (d, J_{PC}=12.0 Hz), 128.37, 124.13, 119.35, 119.30, 97.78, 53.03, 48.53, 44.18, 30.79 (d, J_{PC}=116.1 Hz), 30.14, 24.65, 22.88. **^{31}P NMR (162 MHz, DMSO-*d$_6$*)** δ 24.56

Compound 29

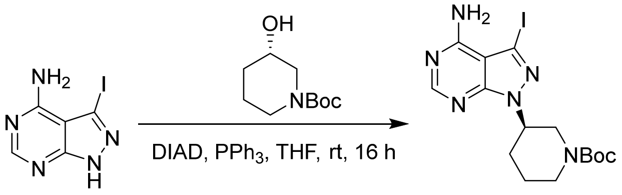

29, 28%

tert-Butyl-(*S*)-3-hydroxypiperidine-1-carboxylate (768 mg, 3.8 mmol, 2 equiv.) and PPh$_3$ (751 mg, 2.9 mmol, 1.5 equiv.) were dissolved in anhydrous THF (7.5 mL), and DIAD (560 µL, 2.9 mmol, 1.5 equiv.) was added at 0 °C. Then, 3-iodo-1*H*-pyrazolo[3,4-*d*]pyrimidin-4-amine (500 mg, 1.9 mmol, 1 equiv.) was added to the reaction solution, and the mixture was stirred at rt for 16 h under N$_2$. After the reaction mixture was filtered, the filtrate was concentrated under reduced pressure. The crude product was purified by column chromatography (EtOAc/Hex=1/1, v/v) to afford **29** as a white solid (237 mg, 28%). The analytical data of **29** are in agreement with the literature data[11]. **HR-MS (ESI)** calcd. for $C_{15}H_{22}IN_6O_2^+$ [M+H]$^+$ 445.0844, found 445.0827. **^{1}H NMR (400 MHz, CDCl$_3$)** δ 8.29 (s, 1H), 6.27 (s, 2H), 4.72 (tt, *J*=10.5, 4.6 Hz, 1H), 4.34 – 3.97 (m, 2H), 3.31 (br, s, 1H), 2.80 (t, *J*=12.3 Hz, 1H), 2.20 – 2.07 (m, 2H), 1.85 (dt, *J*=14.1, 3.5 Hz, 1H), 1.63 (qt, *J*=12.8, 4.4 Hz, 1H), 1.41 (s, 9H). **^{13}C NMR (100 MHz, CDCl$_3$)** δ 157.34, 155.94, 154.57, 153.59, 104.05, 86.09, 79.97, 53.43, 30.21, 28.37.

Compound 30

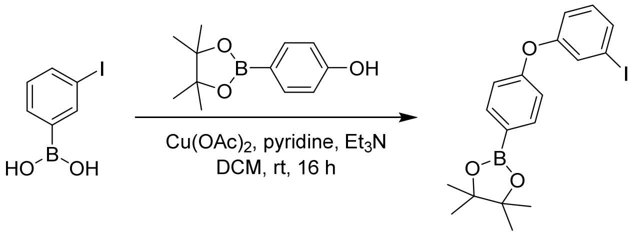

30, 37%

Two round-bottom flasks were charged with 4 Å molecular sieves (1 g each) and dried *in vacuo*. Both flasks were then charged with anhydrous DCM (25 mL each). $Cu(OAc)_2$ (1.1 g, 5.9 mmol, 1.3 equiv.), Et_3N (1.3 mL, 9.1 mmol, 2 equiv.), and 4-(4,4,5,5-tetramethyl-1,3,2-dioxaborolan-2-yl)phenol (1 g, 4.5 mmol, 1 equiv.) were added to one flask. In the other flask, (3-iodophenyl)boronic acid (1.2 g, 5.5 mmol, 1.1 equiv.) and pyridine (366 μL, 4.5 mmol, 1 equiv.) were added. Both flasks were stirred at rt for 4 h under N_2, and the pyridine solution was then added to the other flask. The reaction mixture was stirred for 16 h at rt under O_2. After the reaction was complete, the mixture was filtered, and the filtrate was then concentrated under reduced pressure. The crude product was purified by column chromatography (EtOAc/Hex=1/9 to 1/4, v/v) to afford **30** as a pale-yellow oil (710 mg, 37%). The analytical data of **30** are in agreement with the literature data[12]. **HR-MS (ESI)** calcd. for $C_{18}H_{21}BIO_3^+$ $[M+H]^+$ 423.0623, found 423.0552. **^{1}H NMR (400 MHz, $CDCl_3$)** δ 7.80 (d, *J*=8.5 Hz, 2H), 7.45 (dt, *J*=7.7, 1.4 Hz, 1H), 7.36 (t, *J*=1.9 Hz, 1H), 7.06 (t, *J*=8.1 Hz, 1H), 7.01 – 6.96 (m, 3H), 1.35 (s, 12H). **^{13}C NMR (100 MHz, $CDCl_3$)** δ 159.25, 157.39, 136.74, 132.53, 131.06, 128.05, 118.47, 118.12, 94.17, 83.81, 24.85.

Compound 31

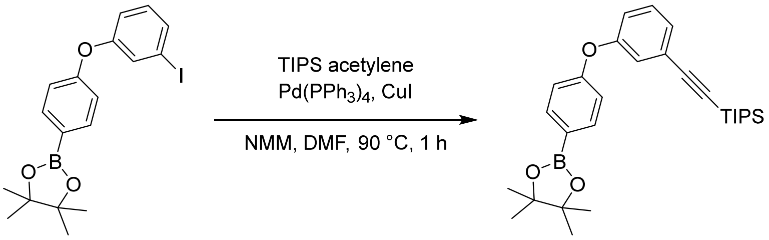

30 **31**, 87%

The synthesis of **30** was reported in the previous literature[12]. Compound **30** (200 mg, 0.47 mmol, 1 equiv.) was dissolved in DMF (670 μL), and *N*-methyl-morpholine (521 μL, 4.7 mmol, 10 equiv.) and ethynyltriisopropylsilane (328 mg, 1.8 mmol, 3.8 equiv.) were added. The solution was degassed using a stream of N_2 for 1 h, and $Pd(PPh_3)_4$ (32.8 mg, 0.028 mmol, 0.06 equiv.) and CuI (1.8 mg, 0.0094 mmol, 0.02 equiv.) were added to the solution in the dark. The mixture was then thoroughly degassed and stirred at 90 °C for 1 h under N_2. After the reaction was complete, the reaction mixture was extracted with Et_2O and H_2O. The organic layer was dried over $MgSO_4$ and concentrated under reduced pressure. The crude product was purified by column chromatography (EtOAc/Hex=1/19, v/v) to afford **31** as a yellow oil (196 mg, 87%). The analytical data of **31** are in agreement with the literature[12]. **HR-MS (ESI)** calcd. for $C_{29}H_{42}BO_3Si^+$ $[M+H]^+$ 477.2991, found 477.2911. **^{1}H NMR (400 MHz, $CDCl_3$)** δ 7.76 (d, *J*=8.6 Hz, 2H), 7.27 – 7.20 (m, 2H), 7.12 (dt, *J*=2.9, 0.8 Hz, 1H), 6.97 – 6.92 (m, 3H), 1.31 (s, 12H), 1.09 (s, 21H). **^{13}C NMR (100 MHz, $CDCl_3$)** δ 159.88, 156.21, 136.66, 129.61, 127.59, 125.12, 122.82, 119.79, 117.63, 106.15, 91.39, 83.76, 24.85, 18.64, 11.26.

Compound 32

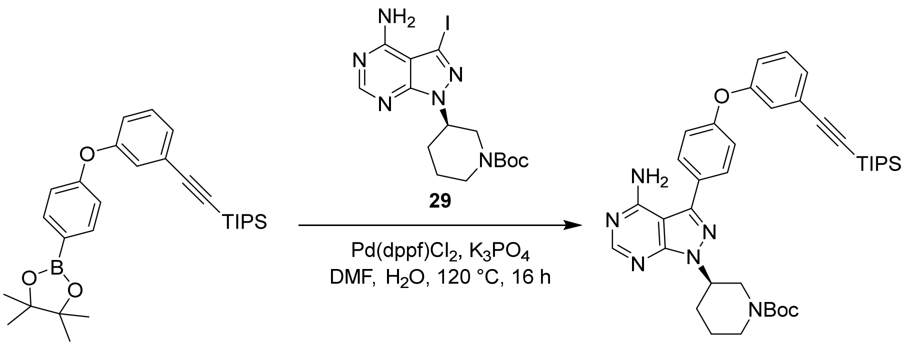

31 **32**, 56%

The synthesis of **29** and **31** was reported in the previous literature[11, 12]. Compound **31** (100 mg, 0.21 mmol, 1 equiv.), **29** (103 mg, 0.23 mmol, 1.1 equiv.) and K_3PO_4 (66.8 mg, 0.31 mmol, 1.5 equiv.) were dissolved in 3:2 DMF/H_2O (840 µL), and the reaction mixture was degassed using a stream of N_2 for 30 min. Pd(dppf)Cl_2 (6.9 mg, 0.0084 mmol, 0.04 equiv.) was then added. After degassed, the reaction mixture was stirred at 120 °C for 16 h under N_2. After the reaction was complete, the mixture was extracted with EtOAc and H_2O, and the organic layer was dried over $MgSO_4$ and concentrated under reduced pressure. The crude product was purified by column chromatography (EtOAc/Hex=1/1, v/v) to afford **32** as a white solid (78.4 mg, 56%). The analytical data of **32** are in agreement with literature data[12]. **HR-MS (ESI)** calcd. for $C_{38}H_{51}N_6O_3Si^+$ $[M+H]^+$ 667.3787, found 667.3804. **^{1}H NMR (400 MHz, CDCl$_3$)** δ 8.34 (s, 1H), 7.63 (d, *J*=8.6 Hz, 2H), 7.30 – 7.25 (m, 2H), 7.14 (dt, *J*=1.9, 1.1 Hz, 1H), 7.11 (d, *J*=8.6 Hz, 2H), 7.00 (dt, *J*=7.4, 2.3 Hz, 1H), 5.61 (s, 2H), 4.81 (tt, *J*=10.8, 4.6 Hz, 1H), 4.24 – 4.01 (m, 2H), 3.46 – 3.30 (m, 1H), 2.82 (t, *J*=12.5 Hz, 1H), 2.32 – 2.11 (m, 2H), 2.02 (s, 1H), 1.67 (m, 1H), 1.42 (s, 9H), 1.10 (s, 21H). **^{13}C NMR (100 MHz, CDCl$_3$)** δ 158.23, 157.43, 156.07, 154.81, 154.62, 153.97, 143.86, 130.02, 129.78, 127.93, 127.90, 125.32, 122.77, 119.78, 119.23, 105.99, 98.43, 79.88, 53.00, 30.17, 28.37, 18.63, 11.26.

Compound 33

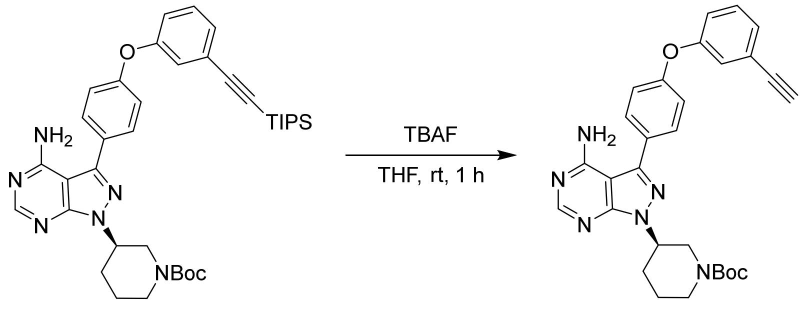

32 **33**, quan.

The synthesis of **32** was reported in the previous literature[12]. Compound **32** (40 mg, 0.060 mmol, 1 equiv.) was dissolved in anhydrous THF (250 µL), and a solution of TBAF in THF (1 M, 60 µL) was added. The reaction solution was stirred at rt for 1 h. After the reaction was complete, the solvent was evaporated under reduced pressure. The crude product was purified by semi-preparative RP-HPLC (H_2O/ACN=80/20 over 6 min, 80/20 to 0/100 over 12 min, 0/100 over 7 min, flow rate=3 mL/min, retention time: 23.5 min), affording **33** (30.6 mg, quan.) as a white solid. **HR-MS (ESI)** calcd. for $C_{29}H_{31}N_6O_3^+$ $[M+H]^+$ 511.2452, found 511.2422. **^{1}H NMR (400 MHz, CDCl$_3$)** δ 8.36 (s, 1H), 7.66 (d, *J*=8.6

Hz, 2H), 7.35 – 7.27 (m, 2H), 7.21 – 7.13 (m, 3H), 7.08 (ddd, *J*=8.0, 2.5, 1.4 Hz, 1H), 5.81 (s, 2H), 4.84 (tt, *J*=10.7, 4.5 Hz, 1H), 4.44 – 4.04 (m, 2H), 3.58 – 3.29 (m, 1H), 3.10 (s, 1H), 2.85 (t, *J*=11.9 Hz, 1H), 2.30 – 2.13 (m, 2H), 1.95 – 1.80 (m, 1H), 1.80 – 1.64 (m, 1H), 1.44 (s, 9H). **^{13}C NMR (100 MHz, $CDCl_3$)** δ 157.87, 157.53, 156.39, 155.05, 154.62, 154.04, 143.71, 130.07, 129.94, 128.26, 127.69, 123.79, 122.57, 120.09, 119.54, 98.45, 82.73, 79.87, 77.99, 52.97, 48.33, 43.21, 30.18, 28.37, 24.35.

Compound 34

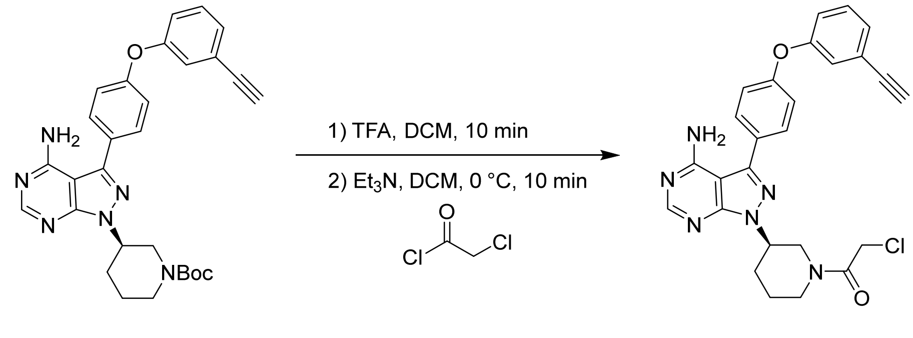

33 **34**, 54%

Compound **33** (30.6 mg, 0.060 mmol, 1 equiv.) was dissolved in anhydrous DCM (600 μL), and TFA was added (600 μL). After stirred at rt for 10 min, the solvent was evaporated *in vacuo*. The mixture was then dissolved in anhydrous DCM (600 μL). After Et_3N (16.8 μL, 0.12 mmol, 2 equiv.) was added, the solution was stirred for 30 min. Then, chloroacetyl chloride (4.8 μL, 0.060 mmol, 1 equiv.) was added to the solution in an ice bath. The reaction mixture was stirred at 0 °C for 10 min and concentrated under reduced pressure. The crude product was purified by semi-preparative RP-HPLC (H_2O/ACN=100/0 over 4 min, 100/0 to 0/100 over 14 min, 0/100 over 6 min, flow rate=3 mL/min, retention time: 21 min), affording **34** (11.4 mg, 54%) as a white solid. **HR-MS (ESI)** calcd. for $C_{26}H_{24}ClN_6O_2^+$ $[M+H]^+$ 487.1644, found 487.1632. **^{1}H NMR (400 MHz, $CDCl_3$)** δ 8.36 (d, *J*=8.7 Hz, 1H), 7.65 (d, *J*=7.6 Hz, 2H), 7.35 – 7.26 (m, 2H), 7.18 – 7.15 (m, 1H), 7.14 (d, *J*=8.2 Hz, 2H), 7.09 (dt, *J*=8.0, 2.0 Hz, 1H), 5.93 (s, 2H), 4.94 (td, *J*=10.3, 5.2 Hz, 0.5 H), 4.87 (td, *J*=10.7, 5.2 Hz, 0.5 H), 4.74 (d, *J*=12.9 Hz, 0.5H), 4.47 (d, *J*=13.4 Hz, 0.5H), 4.17 – 4.04 (m, 2.5H), 3.91 (d, *J*=13.6 Hz, 0.5H), 3.83 (dd, *J*=13.3, 10.2 Hz, 0.5H), 3.42 (t, *J*=11.7 Hz, 0.5H), 3.27 (t, *J*=12.8 Hz, 0.5H), 3.10 (s, 1H), 2.93 (t, *J*=12.4 Hz, 0.5H), 2.45 – 2.26 (m, 2H), 2.07 – 1.96 (m, 2H), 1.84 (d, *J*=13.1 Hz, 0.5H), 1.73 (d, *J*=12.9 Hz, 0.5H). **^{13}C NMR (100 MHz, $CDCl_3$)** δ 165.36, 165.29, 158.16, 157.15, 156.79, 156.22, 154.51, 154.01, 153.68, 144.42, 144.34, 129.99, 127.85, 127.82, 127.71, 123.84, 122.70, 120.21, 119.53, 98.43, 98.19, 82.70, 78.05, 53.29, 52.52, 50.31, 46.36, 46.07, 42.42, 41.11, 30.03, 29.84, 24.82, 23.66.

Compound 10: IbrPPh$_3$-yne

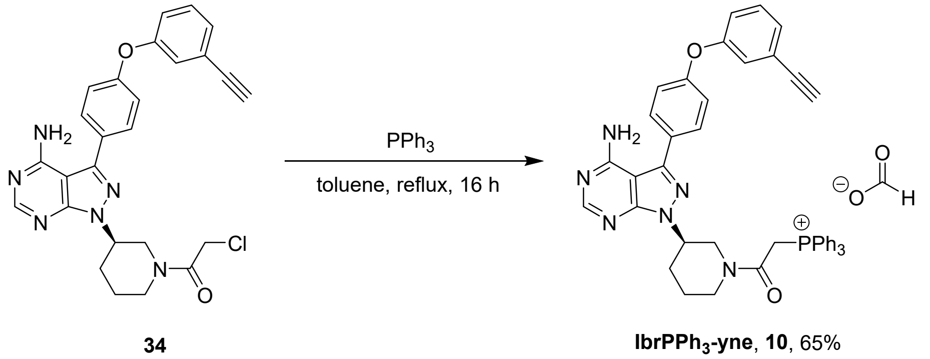

34 **IbrPPh$_3$-yne**, **10**, 65%

Compound **34** (11.4 mg, 0.023 mmol, 1 equiv.) was dissolved in anhydrous toluene (230 µL), and PPh_3 (6.8 mg, 0.026 mmol, 1.1 equiv.) was added. The reaction mixture was stirred and refluxed for 16 h under N_2. After the reaction was complete, the mixture was concentrated under reduced pressure. The crude product was purified by semi-preparative RP-HPLC (H_2O+0.1% formic acid/ACN=100/0 over 6 min, 100/0 to 40/60 over 4 min, 40/60 to 30/70 over 2 min, 30/70 to 20/80 over 2 min, 20/80 to 10/90 over 2 min, 10/90 to 0/100 over 1 min, 0/100 over 3 min, flow rate=3 mL/min, retention time: 16.5 min), affording **10** (8.7 mg, 65%) as a pale-yellow solid. Unknown minor phospho-containing organic impurities were observed and were difficult to remove completely. **HR-MS (ESI)** calcd. for $C_{44}H_{38}N_6O_2P^+$ $[M]^+$ 713.2788, found 713.2879. **^{1}H NMR (800 MHz, DMSO-*d*$_6$)** δ 8.23 (s, 1H), 7.74 – 7.53 (m, 17H), 7.44 – 7.40 (m, 1H), 7.27 (d, *J*=7.6 Hz, 1H), 7.18 – 7.12 (m, 4H), 4.69 (s, 1H), 4.25 (s, 2H), 4.06 – 4.00 (m, 1H), 2.85 (m, 1H), 2.23 – 2.09 (m, 2H), 1.92 – 1.78 (m, 1H), 1.72 – 1.42 (m, 1H). **^{13}C NMR (200 MHz, DMSO-*d*$_6$)** δ 165.11, 158.18, 156.63, 156.36, 155.61, 153.97, 143.09, 132.71 (d, J_{PC}=102.5 Hz), 132.04 (d, J_{PC}=2.2 Hz), 131.47 (d, J_{PC}=9.7 Hz), 130.57, 130.18, 128.76 (d, J_{PC}=11.7 Hz), 128.49, 127.02, 123.39, 121.29, 119.61, 119.55, 97.41, 82.73, 81.57, 52.60, 30.84, 29.57, 24.34. **^{31}P NMR (162 MHz, DMSO-*d*$_6$)** δ 26.71.

Compound 35

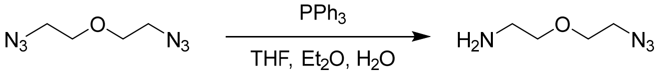

To a solution of 1-azido-2-(2-azidoethoxy)ethane (1 g, 6.4 mmol, 1 equiv.) in THF/Et_2O/H_2O (v/v=5:1:5), PPh_3 (1.6 g, 6.4 mmol, 1 equiv.) dissolved in Et_2O was slowly added over 1 h. After stirred at rt for 12 h under N_2, the mixture was extracted with H_2O and Et_2O. The combined aqueous layer was basified with solid $NaHCO_3$ to pH=11 and extracted with DCM. The combined organic layer was dried over $MgSO_4$ and concentrated under reduced pressure to afford compound **35** (0.58 g, 70%) as a clear oil. The analytical data of **35** are in agreement with the literature data[13]. **^{1}H NMR (400 MHz, $CDCl_3$)** δ 3.64 (t, *J*=5.0 Hz, 2H), 3.51 (t, *J*=5.1 Hz, 2H), 3.37 (t, *J*=5.0 Hz, 2H), 2.86 (t, *J*=5.1 Hz, 2H). **^{13}C NMR (100 MHz, $CDCl_3$)** δ 71.95, 69.09, 49.91, 40.73.

Compound 36

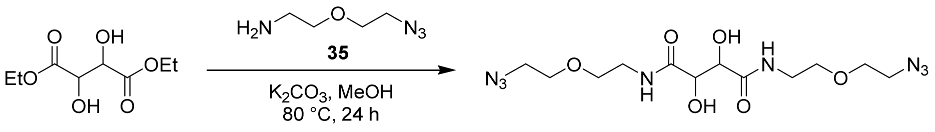

The synthesis of **35** was reported in the previous literature[13]. To a solution of diethyl tartrate (100 µL, 0.58 mmol, 1 equiv.) in MeOH (10 mL) was added compound **35** (150 mg, 1.15 mmol, 2 equiv.), followed by the addition of anhydrous K_2CO_3 (13 mg, 0.08 mmol, 0.14 equiv.). Then, the reaction mixture was stirred at 80 °C for 24 h. After the reaction was complete, the crude product was purified by column chromatography (MeOH/DCM=7/93, v/v) to afford compound **36** (119 mg, 55%) as a white solid. **HR-MS (ESI)** calcd. for $C_{12}H_{23}N_8O_6^+$ $[M+H]^+$, 375.1735; found 375.1678. **^{1}H NMR (400 MHz, $CDCl_3$)** δ 7.36 (s, 2H), 5.04 (d, *J*=7.0 Hz, 2H), 4.30 (d, *J*=6.8 Hz, 2H), 3.66 (t, *J*=4.9 Hz, 4H), 3.59 – 3.43 (m, 8H), 3.39 (t, *J*=4.9 Hz, 4H). **^{13}C NMR (100 MHz, $CDCl_3$)** δ 173.6, 71.1, 70.1, 69.5, 50.8,

39.2.

Compound 16: AldN3

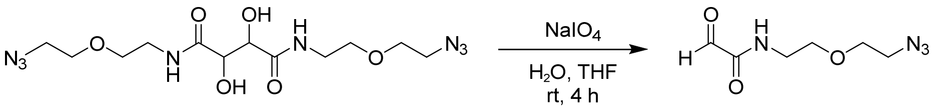

$NaIO_4$ (20 mg, 0.11 mmol, 1 equiv.) in H_2O was added to a solution of compound **36** (40 mg, 0.11 mmol, 1 equiv.) in THF (1 mL) at 0 °C. The mixture was stirred at rt for 4 h. After the reaction was complete, the mixture was dried over $MgSO_4$. Then, all the solids were filtered, and the filtrate was concentrated. The crude product was purified by column chromatography (EtOAc/Hex=7/3, v/v) to afford **16** (12 mg, 60%) as a clear oil. **^{1}H NMR (400 MHz, D_2O)** δ 5.35 (s, 1H), 3.78 (t, *J*=4.8 Hz, 2H), 3.74 (t, *J*=5.4 Hz, 2H), 3.54 (t, *J*=4.8 Hz, 2H), 3.52 (t, *J*=5.4 Hz, 2H). **^{13}C NMR (100 MHz, D_2O)** δ 172.3, 86.7, 69.0, 68.5, 50.1, 38.6.

Compound 37

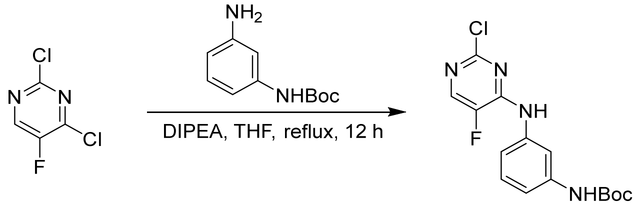

Under N_2 protection, 2,4-dichloro-5-fluoropyrimidine (400 mg, 2.40 mmol, 1 equiv.), *tert*-butyl (3-aminophenyl)carbamate (500 mg, 2.40 mmol, 1 equiv.) and DIPEA (500 µL, 2.80 mmol, 1.2 equiv.) were dissolved in anhydrous THF (12 mL). The reaction mixture was stirred and refluxed for 12 h. After cooling to rt, the mixture was extracted with H_2O and brine. The combined organic layer was dried over $MgSO_4$ and concentrated under reduced pressure. After recrystallization from DCM, compound **37** (533 mg, 64%) was obtained as a crystalline solid. The analytical data of **37** are in agreement with the literature[14]. **HR-MS (ESI)** calcd. for $C_{15}H_{17}ClFN_4O_2^+$ $[M+H]^+$, 339.1018; found, 339.0955. **^{1}H NMR (400 MHz, $CDCl_3$)** δ 8.06 – 8.06 (d, *J*=2.7 Hz, 1H), 7.81 – 7.80 (t, *J*=2.2 Hz, 1H), 7.50 – 7.46 (m, 1H), 7.32 – 7.28 (t, *J*=8.2 Hz, 1H), 7.04 – 7.01 (m, 1H), 7.00 (s, 1H), 6.58 (s, 1H), 1.53 (s, 9H). **^{13}C NMR (100 MHz, $CDCl_3$)** δ 154.35, 152.64, 150.89 (d, J_{FC}=10.1 Hz), 145.02 (d, J_{FC}=259.0 Hz), 140.89 (d, J_{FC}=20.4 Hz), 139.21, 137.54, 129.76, 114.92, 114.56, 110.25, 80.93, 28.31. **^{19}F NMR (376 MHz, $CDCl_3$)** δ -157.64.

Compound 38

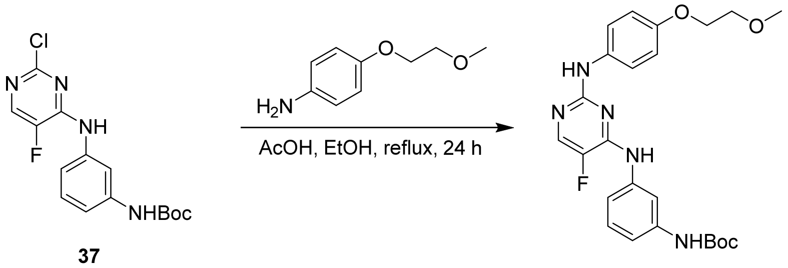

The synthesis of **37** was reported in the previous literature[14]. Compound **37** (106 mg, 0.31 mmol,

1 equiv.) was dissolved in EtOH (10 mL), and 4-(2-methoxyethoxy)aniline (80 mg, 0.48 mmol, 1.5 equiv.) and AcOH (5 drops) were added. The mixture was stirred and refluxed for 24 h. After cooling to rt, the mixture was extracted with H_2O and brine. The combined organic layer was dried over $MgSO_4$ and concentrated under reduced pressure. The crude product was purified by column chromatography (EtOAc/Hex=3/7, v/v) to afford compound **38** (128 mg, 87%) as a white solid. The analytical data of **38** are in agreement with the literature data[14]. **HR-MS (ESI)** calcd. for $C_{24}H_{29}FN_5O_4^+$ $[M+H]^+$, 470.2198; found, 470.2221. **^{1}H NMR (400 MHz, CDCl$_3$)** δ 7.88 (d, *J*=1.6 Hz, 1H), 7.51 (s, 1H), 7.44 – 7.40 (m, 2H), 7.32 – 7.31 (m, 1H), 7.23 (t, *J*=8.0 Hz, 1H), 7.05 – 7.02 (m, 1H), 6.97 (s, 1H), 6.91 – 6.83 (m, 2H), 6.58 (s, 1H), 4.15 – 4.06 (m, 2H), 3.76 – 3.73 (m, 2H), 3.45 (s, 3H), 1.53 (s, 9H). **^{13}C NMR (100 MHz, CDCl$_3$)** δ 155.86, 154.93, 152.87, 150.26 (d, J_{FC}=10.2 Hz), 140.91 (d, J_{FC}=245.0 Hz), 139.79 (d, J_{FC}=21.0 Hz), 139.24, 138.67, 133.21, 129.53, 122.00, 115.23, 115.11, 113.91, 110.77, 80.93, 71.28, 67.85, 59.31, 28.49. **^{19}F NMR (376 MHz, CDCl$_3$)** δ -167.61.

Compound 39

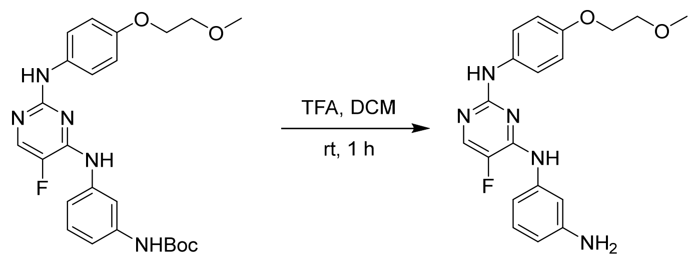

38 **39**, quan.

The synthesis of **38** was reported in the previous literature[14]. Compound **38** (210 mg) was dissolved in anhydrous DCM (1 mL), and TFA (1 mL) was added. After the reaction was stirred at rt for 1 h, the reaction mixture was extracted with sat. $NaHCO_{3(aq)}$ and brine. The combined organic layer was dried over $MgSO_4$ and concentrated under reduced pressure. The residue was evaporated *in vacuo* to afford compound **39** (198 mg, quant.) as a white solid. The analytical data of **39** are in agreement with the literature data[14]. **HR-MS (ESI)** calcd. for $C_{19}H_{21}FN_5O_2^+$ $[M+H]^+$, 370.1673; found 370.1608. **^{1}H NMR (400 MHz, CDCl$_3$)** δ 7.91 (d, *J*=3.3 Hz, 1H), 7.45 – 7.40 (m, 2H), 7.09 (t, *J*=8.0 Hz, 1H), 6.94 – 6.90 (m, 2H), 6.78 (s, 1H), 6.75 – 6.73 (m, 1H), 6.66 (s, 1H), 6.43 – 6.40 (m, 1H), 4.13 – 4.10 (m, 2H), 3.76 – 3.73 (m, 2H), 3.65 (s, 1H), 3.46 (s, 3H). **^{13}C NMR (100 MHz, DMSO-*d*$_6$)** δ 155.72 (d, J_{FC}=2.8 Hz), 152.97, 149.83 (d, J_{FC}=10.6 Hz), 148.76, 140.38 (d, J_{FC}=245 Hz), 140.20 (d, J_{FC}=19.8 Hz), 139.39, 134.24, 128.66, 120.31, 114.22, 109.77, 109.46, 107.48, 70.50, 67.00, 58.15. **^{19}F NMR (376 MHz, CDCl$_3$)** δ -167.25.

Compound 40

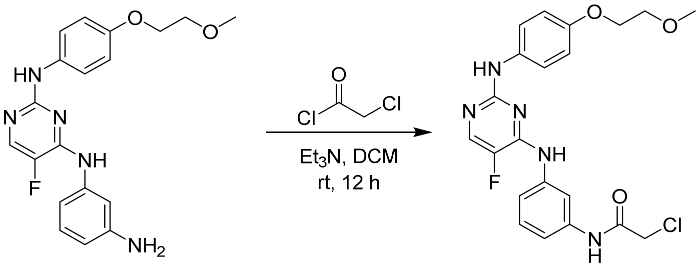

39 **40**, 58%

The synthesis of **39** was reported in the previous literature[14]. Under N_2 protection, chloroacetyl chloride (17 mg, 0.15 mmol, 1.1 equiv.) dissolved in DCM was slowly added to a solution of compound **39** (50 mg, 0.13 mmol, 1 equiv.) and Et_3N (20 µL, 0.13 mmol, 1 equiv.) in DCM at 0 °C. After stirring at 0 °C for 1 h and then stirring at rt for an additional 12 h, the reaction mixture was concentrated under reduced pressure. The crude product was purified by column chromatography (MeOH/DCM=1/19, v/v) to afford compound **40** (34 mg, 58%) as a yellow solid. **HR-MS (ESI)** calcd. for $C_{21}H_{22}ClFN_5O_3^+$ $[M+H]^+$, 446.1389; found 446.1344. **^{1}H NMR (400 MHz, DMSO-*d*$_6$)** δ 10.27 (s, 1H), 9.38 (s, 1H), 8.96 (s, 1H), 8.07 (d, *J*=3.6 Hz, 1H), 7.84 (s, 1H), 7.59 (d, *J*=7.2, 1H), 7.54 – 7.50 (m, 2H), 7.32 – 7.28 (m, 2H), 6.80 – 6.76 (m, 2H), 4.25 (s, 2H), 4.04 – 4.00 (m, 2H), 3.64 – 3.62 (m, 2H), 3.30 (s, 3H). **^{13}C NMR (100 MHz, DMSO-*d*$_6$)** δ 164.66, 155.77 (d, J_{FC}=2.7 Hz), 153.15, 149.76 (d, J_{FC}=10.5 Hz), 140.88 (d, J_{FC}=19.7 Hz), 140.41 (d, J_{FC}=245 Hz), 139.31, 138.54, 134.13, 128.83, 120.52, 117.64, 114.76, 114.21, 113.26, 70.58, 67.08, 58.24, 43.61. **^{19}F NMR (376 MHz, DMSO-*d*$_6$)** δ -164.89.

Compound 9: SpePPh$_3$

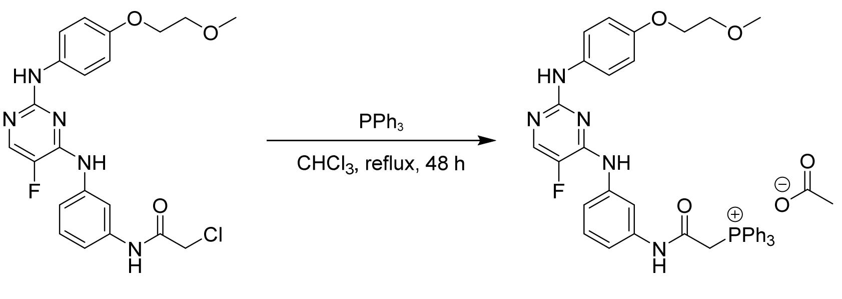

40 **SpePPh$_3$**, **9**, 66%

PPh_3 (16 mg, 0.06 mmol, 1.5 equiv.) was added to a solution of compound **40** (20 mg, 0.04 mmol, 1 equiv.) in $CHCl_3$. The mixture was stirred and refluxed for 48 h under N_2. After cooling to rt, the mixture was concentrated under reduced pressure. The crude product was purified by semi-preparative RP-HPLC (H_2O/ACN+0.1% AcOH=100/0 over 6 min, 100/0 to 0/100 over 6 min, flow rate=3 mL/min, retention time: 21 min), affording compound **9** (17 mg, 66% yield) as an orange to brown solid. **HR-MS (ESI)** calcd. for $C_{39}H_{36}FN_5O3P^+$ $[M]^+$, 672.2534, found, 672.2551. **^{1}H NMR (400 MHz, DMSO-*d*$_6$)** δ 10.58 (s, 1H), 9.43 (s, 1H), 9.02 (s, 1H), 8.08 (d, *J*=4.0 Hz, 1H), 7.92 – 7.74 (m, 16H), 7.70 (d, *J*=8.0, 1H), 7.54 – 7.45 (m, 2H), 7.51 – 7.48 (m, 1H), 7.25 (t, *J*=8.0, 1H), 7.04 (d, *J*=8.0, 1H), 6.82 – 6.80 (m, 2H), 5.17 (d, *J*=14.4 Hz, 2H), 4.04 – 4.00 (m, 2H), 3.64 – 3.62 (m, 2H), 3.30 (s, 3H). **^{13}C NMR (100 MHz, DMSO-*d*$_6$)** δ 165.78, 155.62 (d, J_{FC}=2.9 Hz), 153.03, 149.62 (d, J_{FC}=10.9 Hz), 140.44 (d, J_{FC}=19.4 Hz), 140.38 (d, J_{FC}=246 Hz), 134.48, 134.26, 133.67 (d, J_{PC}=10.5 Hz), 129.87 (d, J_{PC}=12.7 Hz), 128.79 (d, J_{PC}=11.9 Hz), 128.51, 120.25, 116.61, 114.23, 114.10, 112.47, 70.52, 67.03, 58.17, 32.25 (d, J_{PC}=66.1 Hz). **^{19}F NMR (376 MHz, DMSO-*d*$_6$)** δ -165.54. **^{31}P NMR (162 MHz, DMSO-*d*$_6$)** δ 21.35.

Compound 41

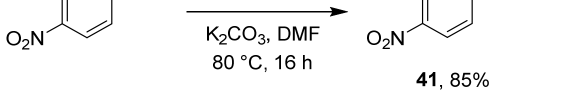

41, 85%

4-Nitrophenol (512.5 mg, 1 equiv.) and 80% propargyl bromide in toluene (702.6 mg, 1.3 equiv.) were dissolved in DMF (10 mL), followed by the addition of K_2CO_3 (644.4 mg, 1.3 equiv.). The reaction

mixture was heated to 80 °C and stirred for 16 h. After cooling to rt, EtOAc was added, and the mixture was extracted with H_2O and brine. The combined organic layer was dried over Na_2SO_4 and concentrated under reduced pressure. The residue was then purified by column chromatography (DCM/Hex=1/1, v/v) to afford pure **41** (551.5 mg, 85%). The analytical data of **41** are in agreement with literature data[15]. **^{1}H NMR (400 MHz, $CDCl_3$)** δ 8.25 – 8.20 (m, 2H), 7.08 – 7.03 (m, 2H), 4.80 (d, *J*=2.4 Hz, 2H), 2.59 (t, *J*=2.4 Hz, 1H). **^{13}C NMR (100 MHz, $CDCl_3$)** δ 162.31, 142.14, 125.83, 114.97, 77.08, 76.74, 56.28.

Compound 42

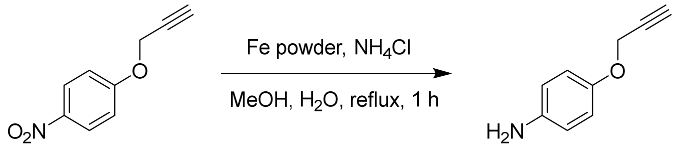

41 **42**, 85%

The synthesis of **41** was reported in the previous literature[15]. Compound **41** (512.5 mg, 1 equiv.) and NH_4Cl (136 mg, 0.9 equiv.) were dissolved in MeOH/H_2O (5:1, 30 mL), followed by the addition of Fe powder (1094.2 mg, 6.8 equiv.). The reaction mixture was stirred and refluxed for 1 h. After cooling to rt, EtOAc was added, and the mixture was extracted with sat. $Na_2CO_{3(aq)}$ and brine. The combined organic layer was dried over Na_2SO_4 and concentrated under reduced pressure. Compound **42** (398.86 mg, 85%) was obtained without further purification. **^{1}H NMR (400 MHz, $CDCl_3$)** δ 6.85 – 6.80 (m, 2H), 6.67 – 6.62 (m, 2H), 4.61 (d, *J*=2.4 Hz, 2H), 3.47 (s, 2H), 2.49 (t, *J*=2.4 Hz, 1H). **^{13}C NMR (100 MHz, $CDCl_3$)** δ 150.69, 140.88, 116.38, 116.21, 79.09, 75.10, 56.72.

Compound 43

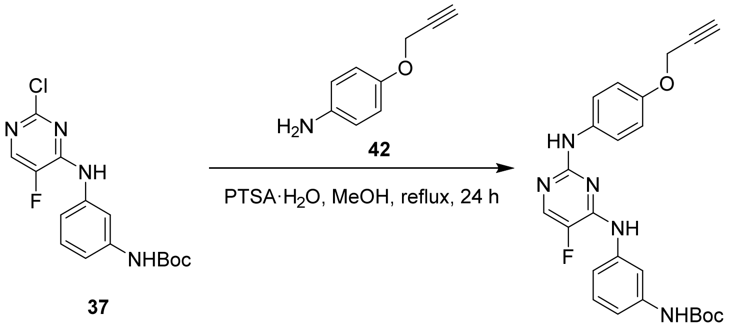

37 **43**, 56%

The synthesis of **37** was reported in the previous literature[14]. Compound **42** (176.7 mg, 1 equiv.), compound **37** (625 mg, 1.5 equiv.) and *p*-toluenesulfonic acid monohydrate (13.2 mg, 0.05 equiv.) were dissolved in MeOH (4 mL). The reaction mixture was stirred and refluxed for 24 h. After cooling to rt, EtOAc was added, and the mixture was extracted with H_2O and brine. The combined organic layer was dried over Na_2SO_4 and concentrated under reduced pressure. The residue was then purified by column chromatography (EtOAc/Hex=2/3, v/v) to afford pure **43** (301.2 mg, 56%). **HR-MS (ESI)** calcd. for $C_{24}H_{25}FN_5O_3^+$ $[M+H]^+$, 450.1941; found, 450.1923. **^{1}H NMR (400 MHz, DMSO-*d*$_6$)** δ 9.30 (s, 1H), 9.28 (s, 1H), 8.94 (s, 1H), 8.03 (d, *J*=3.8 Hz, 1H), 7.81 (t, *J*=2.1 Hz, 1H), 7.55 – 7.51 (m, 2H), 7.37 – 7.33 (m, 1H), 7.18 (t, *J*=7.9 Hz, 1H), 7.13 (dt, *J*=8.3, 1.6 Hz, 1H), 6.83 – 6.79 (m, 2H), 4.69 (d, *J*=2.4 Hz, 2H), 3.51 (t, *J*=2.4 Hz, 1H), 1.44 (s, 9H). **^{13}C NMR (100 MHz, DMSO-*d*$_6$)** δ 155.62 (d, J_{FC}=2.8 Hz), 152.73, 151.74, 149.76 (d, J_{FC}=10.8 Hz), 141.59, 140.59 (d, J_{FC}=245 Hz, 1H), 140.65 (d, J_{FC}=20.6 Hz), 139.59,

139.14, 139.02, 134.73, 128.42, 120.14, 115.89, 114.68, 113.55, 112.22, 79.54, 79.00, 77.92, 55.67, 28.12. **^{19}F NMR (376 MHz, DMSO-*d$_6$*)** δ -165.36.

Compound 44

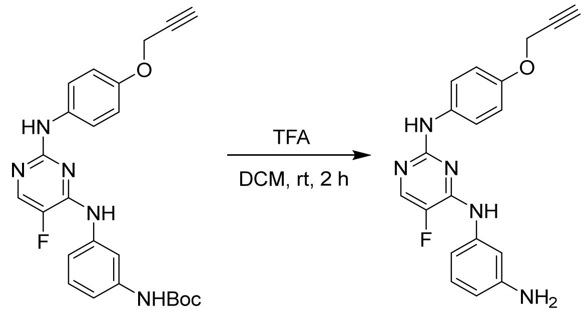

43 **44**, 90%

Compound **43** (199 mg) was dissolved in DCM (7 mL), and then TFA (1 mL) was slowly added in an ice bath. The reaction mixture was stirred at rt for 2 h, and the reaction mixture was quenched with sat. $Na_2CO_{3(aq)}$, extracted with DCM. The combined organic layer was dried over Na_2SO_4 and concentrated under reduced pressure. The residue was then purified by column chromatography (EtOAc/Hex=2/3, v/v) to afford pure **44** (139.7 mg, 90%). **HR-MS (ESI)** calcd. for $C_{19}H_{17}FN_5O^+$ [M+H]$^+$, 350.1417; found, 350.1394. **^{1}H NMR (400 MHz, DMSO-*d$_6$*)** δ 8.99 (s, 1H), 8.92 (s, 1H), 7.99 (d, *J*=3.9 Hz, 1H), 7.59 – 7.53 (m, 2H), 6.97 – 6.93 (m, 2H), 6.90 (dt, *J*=8.1, 1.5 Hz, 1H), 6.87 – 6.82 (m, 2H), 6.30 (ddd, *J*=7.7, 2.3, 1.3 Hz, 1H), 4.95 (s, 2H), 4.69 (d, *J*=2.4 Hz, 2H), 3.51 (t, *J*=2.3 Hz, 1H). **^{13}C NMR (100 MHz, DMSO-*d$_6$*)** δ 155.68 (d, J_{FC}=2.9 Hz), 151.71, 149.87 (d, J_{FC}=10.6 Hz), 148.78, 141.66, 141.66, 140.44 (d, J_{FC}=245 Hz) 140.21 (d, J_{FC}=19.1 Hz), 139.40, 139.21, 139.21, 134.89, 128.70, 120.17, 114.81, 109.82, 109.52, 107.47, 79.62, 77.95, 55.71. **^{19}F NMR (376 MHz, DMSO-*d$_6$*)** δ -165.33.

Compound 45

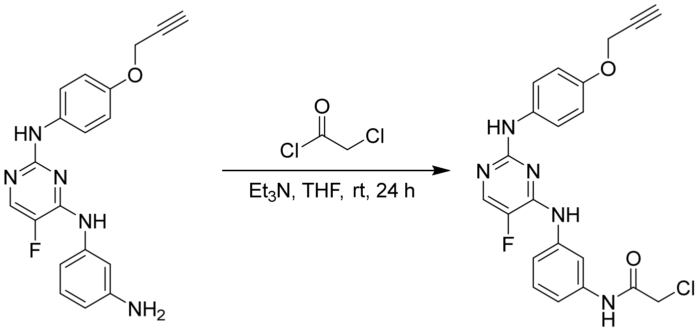

44 **45**, 62%

Compound **44** (49.1 mg, 1 equiv.) and Et_3N (12 µL, 1.1 equiv.) were dissolved in THF (3 mL), and then, chloroacetyl chloride (13 µL, 1.1 equiv.) was added in an ice bath. The reaction mixture was stirred at rt for 24 h. As the reaction proceeded, precipitate was formed. After 24 h, the reaction mixture was filtered, and the filter cake was washed with THF. The residue was then purified by column chromatography (EtOAc/Hex=2/3, v/v) to afford pure **45** (37.3 mg, 62%). **HR-MS (ESI)** calcd. for $C_{21}H_{18}ClFN_5O_2^+$ [M+H]$^+$, 426.1133; found, 426.1145. **^{1}H NMR (400 MHz, DMSO-*d$_6$*)** δ 10.25 (s, 1H), 9.38 (s, 1H), 8.98 (s, 1H), 8.05 (d, *J*=3.7 Hz, 1H), 7.84 – 7.77 (m, 1H), 7.61 – 7.56 (m, 1H), 7.55 – 7.49 (m, 2H), 7.29 – 7.26 (m, 2H), 6.83 – 6.79 (m, 2H), 4.69 (d, *J*=2.4 Hz, 2H), 4.23 (s, 2H), 3.51 (t, *J*=2.4 Hz, 1H). **^{13}C NMR (100 MHz, DMSO-*d$_6$*)** δ 164.55, 155.63, 151.77, 149.70 (d, J_{FC}=10.9 Hz), 140.78 (d,

J_{FC}=19.5 Hz), 140.40 (d, J_{FC}=245 Hz), 139.23, 138.49, 134.71, 128.72, 120.25, 117.56, 114.71, 113.13, 79.58, 77.91, 55.68, 43.55. **^{19}F NMR (376 MHz, DMSO-*d*$_6$)** δ -165.14.

Compound 11: SpePPh$_3$-yne

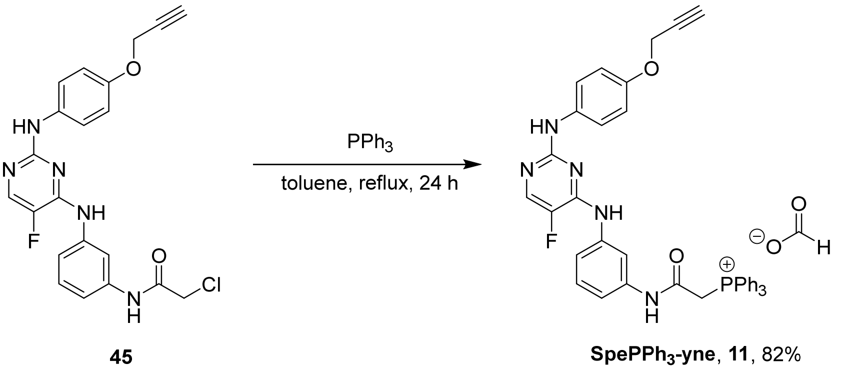

Compound **45** (49.2 mg, 1 equiv.) and PPh_3 (32.8 mg, 1.4 equiv.) were dissolved in toluene (5 mL). The reaction mixture was refluxed and stirred for 24 h. As the reaction proceeded, precipitate was formed. After cooling to rt, the reaction mixture was filtered, and the filter cake was washed with toluene. The residue was then purified by column chromatography (EtOAc/Hex=1/1, v/v) to afford **11** (49.2 mg, 82%). Small portions of compound **11** were further purified by semi-preparative RP-HPLC (H_2O+0.1% formic acid/ACN=100/0 over 6 min, 100/0 to 0/100 over 12 min, 0/100 over 6 min, flow rate=3 mL/min, retention time: 15 min) for biological experiments. **HR-MS (ESI)** calcd. for $C_{39}H_{32}FN_5O_2P^+$ [M]$^+$, 652.2272; found, 652.2169. **^{1}H NMR (400 MHz, DMSO-*d*$_6$)** δ 9.30 (s, 1H), 9.12 (s, 1H), 8.43 (s, 1H), 8.02 (d, *J*=3.7 Hz, 1H), 7.98 (s, 1H), 7.79 – 7.73 (m, 9H), 7.69 – 7.64 (m, 6H), 7.54 (d, *J*=8.4 Hz, 2H), 7.43 (s, 1H), 7.16 – 7.12 (m, 1H), 6.84 (d, *J*=8.5 Hz, 2H), 4.68 (d, *J*=2.3 Hz, 2H), 3.51 (t, *J*=2.4 Hz, 1H). **^{13}C NMR (100 MHz, DMSO-*d*$_6$)** δ 155.60, 151.79, 149.65 (d, J_{FC}=10.8 Hz), 140.48 (d, J_{FC}=20.1 Hz), 140.45 (d, J_{FC}=245 Hz),139.44, 138.76, 134.87, 134.62, 133.71 (d, $J_{PC=}$10.6 Hz), 129.93 (d, $J_{PC=}$12.8 Hz), 128.80 (d, J_{PC}=11.8 Hz), 128.58, 120.18, 116.87, 114.82, 114.22, 112.54, 79.63, 77.96, 55.73, 32.28 (d, J_{PC}=63.8 Hz). **^{19}F NMR (376 MHz, DMSO-*d*$_6$)** δ -73.84. **^{31}P NMR (162 MHz, DMSO-*d*$_6$)** δ 26.39.

Compound 6: Tz-AAM-Ph

10 µL of **Tz-PPh$_3$ (1)** (10 mM in DMSO) and 10 µL of benzaldehyde (5 mM in H_2O) were added to 80 µL of 1× PBS (pH 7.4) with 26 mM $NaHCO_3$. The reaction mixture was incubated at 37 °C for 4 h and purified by analytical RP-HPLC (H_2O+0.1% formic acid/ACN=100/0 over 4 min, 100/0 to 0/100 over 16 min, 0/100 over 4 min, flow rate=1 mL/min, retention time: 19.8 min). Each HPLC fraction was detected by MS, and the fractions containing **Tz-AAM-Ph (6)** were collected and lyophilized. **HR-MS (ESI)** calcd. for $C_{19}H_{18}N_5O^+$ [M+H]$^+$ 332.1506, found 332.1460 **(Figures S4** and **S15)**.

Compound 7: D-Tz-AAM-Ph

10 µL of **D-Tz-PPh$_3$ (3)** (10 mM in DMSO) and 10 µL of benzaldehyde (5 mM in H_2O) were added to 80 µL of 1× PBS (pH 7.4) with 26 mM $NaHCO_3$. The reaction mixture was incubated at 37 °C for 4 h and purified by analytical RP-HPLC (H_2O+0.1% formic acid/ACN=100/0 over 4 min, 100/0 to 0/100 over 16 min, 0/100 over 4 min, flow rate=1 mL/min, retention time: 19.8 min). Each HPLC fraction was detected by MS, and the fractions containing **D-Tz-AAM-Ph (7)** were collected and lyophilized.

HR-MS (ESI) calcd. for $C_{19}H_{18}N_5O^+$, $C_{19}H_{17}DN_5O^+$, $C_{19}H_{16}D_2N_5O^+$, and $C_{19}H_{15}D_3N_5O^+$; $[M+H]^+$ 332.1506, 333.1569, 334.1632, and 335.1694; found 332.1453 (8.1%), 333.1511 (22.0%), 334.1566 (34.3%), and 335.1640 (28.8%) **(Figure S15)**.

Ibrutinib (Ibr)

5 µL of **IbrPPh$_3$ (8)** (10 mM in DMSO) and 30 µL of formaldehyde (5 mM in H_2O) were added to 65 µL of 1× PBS (pH 7.4) with 26 mM $NaHCO_3$. The reaction mixture was incubated at 37 °C for 1 h and purified by analytical RP-HPLC (H_2O/ACN=100/0 over 4 min, 100/0 to 0/100 over 12 min, 0/100 over 4 min, flow rate=1 mL/min, retention time: 18.0 min). Each HPLC fraction was detected by MS, and the fractions containing Ibr were collected and lyophilized. **HR-MS (ESI)** calcd. for $C_{25}H_{25}N_6O_2^+$ $[M+H]^+$ 441.2034, found 441.2070 **(Figure S27)**.

Spebrutinib (Spe)

5 µL of **SpePPh$_3$ (9)** (10 mM in DMSO) and 30 µL of formaldehyde (5 mM in H_2O) were added to 65 µL of 1× PBS (pH 7.4) with 26 mM $NaHCO_3$. The reaction mixture was incubated at 37 °C for 1 h and purified by analytical RP-HPLC (H_2O/ACN=100/0 over 4 min, 100/0 to 0/100 over 12 min, 0/100 over 4 min, flow rate=1 mL/min, retention time: 15.7 min). Each HPLC fraction was detected by MS, and the fractions containing Spe were collected and lyophilized. **HR-MS (ESI)** calcd. for $C_{22}H_{23}FN_5O_3^+$ $[M+H]^+$ 424.1780, found 424.1799 **(Figure S27)**.

SpeCH$_3$

In the purification process of Spe, the corresponding side product **SpeCH$_3$** were also collected (retention time: 13.5 min) and lyophilized. **HR-MS (ESI)** calcd. for $C_{21}H_{23}FN_5O_3^+$ $[M+H]^+$ 412.1780, found 412.1769 **(Figure S27)**.

3. Biological Materials and Instrumentation

3.1 Chemicals, critical commercial assays, and antibodies

Chemicals	Source	Identifier
RPMI 1640 Medium	Simply	Cat# CC142-0500
Fetal Bovine Serum, FBS	Gibco	Cat# 10437028
Penicillin-Streptomycin, PS, 100×	Simply	Cat# CC502-0100
0.25% Trypsin-EDTA, 1×	Simply	Cat# CC508-0100
Bovine Serum Albumin, BSA	Bioshop	N/A
Acrylamide/Bis-Acrylamide (37.5:1) 30% Solution	Bioshop	Cat# ACR010.500
RIPA lysis buffer A ([Tris-HCl]=10 mM, pH 8.0, [NaCl]=150 mM, [SDS]= 0.1%, [Triton X-100]=1%, [sodium deoxycholate, SDC]= 0.1%)	Lab stock	N/A
RIPA lysis buffer B ([Tris-HCl]=50 mM, pH 8.0, [NaCl]=150 mM, [SDS]= 0.1%, [Triton X-100]=1%, [sodium deoxycholate, SDC]= 0.5%)	Lab stock	N/A
4× Sampling buffer ([Tris-HCl]=160 mM, pH 6.5, [SDS]=4%)	Lab stock	N/A
4× SDS-PAGE loading buffer ([Tris-HCl]=200 mM, pH 6.8, [DTT]=400 mM, [SDS]=8%, 0.4% bromophenol blue, 40% glycerol)	Lab stock	N/A
100× Protease Inhibitor Cocktail	APExBIO	Cat# K1007
Iodoacetamide, IAA	Sigma-Aldrich	CAS 144-48-9
Western Blot Blocking Buffer (TBS buffer, pH 7.4, 5% BSA (w/v))	Lab stock	N/A
Western Blot Washing Buffer (PBS buffer, pH 7.4, 0.1% Tween 20 (v/v))	Lab stock	N/A
Streptavidin Sepharose High Performance	Cytiva	Cat# 17-5113-01
Dithiothreitol, DTT	Nova-Matls®	CAS 3483-12-3
Trichloroacetic acid, TCA	Nova-Matls®	CAS 76-03-9
**Critical commercial assays**	**Source**	**Identifier**
Dual-Range Bradford Reagent	Visual Protein	Cat# BR01-500
T-Pro LumiLong Plus Chemilumine-scence Detection Kit	T-Pro Biotechnology	Cat# JT96-K004S
**Antibodies**	**Source**	**Identifier**
Btk (D3H5) Rabbit mAb	Cell Signaling Technology	Cat# 8547
Anti GAPDH Rabbit Polyclonal Antibody	Bioman	Cat# GAP001R
Peroxidase AffiniPure™ Goat Anti-Rabbit IgG (H+L)	Jackson Immunoresearch	Cat# 111-035-003

3.2 Instrumentation

The detection of in-gel fluorescence was performed on an Odyssey® CLx Infrared Imaging System (LI-COR Biosciences). The enhanced chemiluminescence (ECL) was recorded by a Vilber Lourmat Fusion FX7 Imaging System using a T-pro LumiLong Plus Chemiluminescent Substrate Kit. The

detection of fluorescence signals in alamarBlue assays was performed on a BioTek Synergy H1 hybrid multimode microplate reader.

All the HPLC mobile phases were prepared with HPLC grade solvents or Milli-Q water (18.2 $M\Omega cm^{-1}$) and filtered through a 0.2 µm filter before use. HPLC analysis of **Tz-PPh$_3$ (1)** and **Tz-ONH$_2$ (2)** was performed on an Agilent 1260 HPLC system with a YMC-Triart C18 column (5 µm, 4.6 i.d. × 250 mm) (H_2O+0.1% TFA/ACN+0.1% TFA=100/0 over 4 min, 100/0 to 30/70 over 8 min, 30/70 to 0/100 over 8 min, then 0/100 to 100/0 over 1 min, flow rate=1 mL/min). HPLC analysis of **IbrPPh$_3$-yne (10)** and **SpePPh$_3$-yne (11)** was performed on an Agilent 1100 Series HPLC system with a Waters Atlantis® dC18 column (5 µm, 4.6 i.d. × 250 mm) (H_2O+0.1% formic acid/ACN=100/0 over 4 min, 100/0 to 30/70 over 8 min, 30/70 to 0/100 over 8 min, then 0/100 to 100/0 over 1 min, flow rate=1 mL/min). The HPLC analysis of stability was performed on an Agilent 1100 Series HPLC system with a YMC-Triart C18 column (5 µm, 4.6 i.d. × 250 mm) (H_2O+0.1% TFA/ACN+0.1% TFA=100/0 over 4 min, 100/0 to 0/100 over 18 min, 0/100 over 4 min, then 0/100 to 100/0 over 1 min, flow rate=1 mL/min).

All LC–MS experiments were performed on a Dionex Ultimate 3000 or an Agilent 1260 HPLC system coupled with a Bruker microTOF QII mass spectrometer with a Waters Atlantis® dC18 column (5 µm, 4.6 i.d. × 250 mm) (H_2O+0.1% formic acid/ACN=100/0 over 4 min, 100/0 to 30/70 over 8 min, 30/70 to 0/100 over 8 min, then 0/100 to 100/0 over 4 min, flow rate=1.2 mL/min, column oven temperature=30 °C).

4. Chemoselective Labeling of Model Carbonyls and Metabolites

4.1 Labeling experiments of model carbonyls using triphenylphosphonium probes

In the experiments of evaluating reactivity differences toward several carbonyl species **(Figures 2a**, **S6** and **S20)**, 95 µL of PBS (pH 7.4) with 26 mM $NaHCO_3$, 4 µL of the substrate stock solution (aldehydes, 5 mM in H_2O; ketones or sugars, 125 mM in H_2O) and 1 µL of the probe stock solution (**Tz-PPh$_3$ (1)**, **IbrPPh$_3$-yne (10)** or **SpePPh$_3$-yne (11)**, 10 mM in DMSO) were added and mixed sequentially. The mixture was incubated at 37 °C. After the given time, 100 µL of the sample was taken, mixed with 100 µL of DMSO and H_2O, and stored at -20 °C immediately. The samples were analyzed by HPLC, and the absorbance at 280 nm was recorded.

4.2 Labeling experiments of a carbonyl mixture using triphenylphosphonium probes

In the experiments of labeling a carbonyl mixture, 84 µL of PBS (pH 7.4) with 26 mM $NaHCO_3$ **(Figure 2b)**, 4 µL of the substrate stock solution (pyruvic acid, 125 mM in H_2O; glucose, 125 mM in H_2O; ribose, 125 mM in H_2O), 1 µL of the substrate stock solution (formaldehyde (FA), 5 mM in H_2O; methylglyoxal (MGO), 5 mM in H_2O; glyoxylic acid (GA)**,** 5 mM in H_2O) and 1 µL of the probe stock solution (**Tz-PPh$_3$ (1)** or **D-Tz-PPh$_3$ (3)**, 10 mM in DMSO) were added and mixed sequentially. The mixture was incubated at 37 °C for 16 h, and both samples were analyzed by LC–MS. The absorbance at 254 nm was recorded.

4.3 Labeling experiments of metabolites using triphenylphosphonium probes

In the experiments of labeling metabolites *in vitro* **(Figures 2d**, **2e**, **5e**, **S10-S12**, **S22**, **S25b** and **S26)**, to 198 µL of serum-free RPMI or cell lysate (adjusted to pH=8.0 with 4 M $NaOH_{(aq)}$), 2 µL of the probe stock solution (**Tz-PPh$_3$ (1)**, **D-Tz-PPh$_3$ (3)**, **IbrPPh$_3$-yne (10)** or **SpePPh$_3$-yne (11)**, 10 mM in

DMSO) was added. The reaction mixture was incubated at 37 °C for 16 h and subjected to the following click reaction.

In the experiments of labeling metabolites *ex vivo* **(Figures 2d**, **2e**, **3**, **S10**, **S13**, **S14**, **S16** and **S17)**, 5x10^6 cells in 500 µL of serum-free RPMI adjusted to pH 8.0 with 4 M NaOH $_{(aq)}$ were seeded in a 48-well cell culture plate and treated with 2 µL of the probe stock solution (**Tz-PPh₃** or **D-Tz-PPh₃**, 10 mM in DMSO) at 37 °C for 16 h. In the relative quantification experiments, 1 µL of the corresponding internal standard (IS) stock solution (**Tz-AAM-Ph (6)** or **D-Tz-AAM-Ph (7)**, 2 mM in DMSO) was added. Then, the cells were lysed through a freeze–thaw process (frozen in liquid nitrogen and then thawed at 37 °C for 3 cycles in total) and subjected to the following click reaction.

4.4 Labeling experiments of model carbonyls using aminooxy probes

In the experiments of evaluating reactivity differences toward several carbonyl species **(Figures 2a** and **S5)**, 95 µL of 0.1 M ammonium acetate buffer (pH 4.5) with 10 mM aniline, 4 µL of the substrate stock solution (aldehydes or ketones, 5 mM in H_2O; sugars, 125 mM in H_2O) and 1 µL of probe stock solution (**Tz-ONH₂ (2)**, 10 mM in DMSO) were added and mixed sequentially. The mixture was incubated at 37 °C. After the given time, 100 µL of the sample was taken, mixed with 100 µL of MeOH and H_2O, and stored at -20 °C immediately. The samples were analyzed by HPLC, and the absorbance at 280 nm was recorded.

4.5 Labeling experiments of metabolites using aminooxy probes

In the experiments of labeling metabolites *in vitro* **(Figures 2d**, **2e** and **S7-S9)**, 196 µL of serum-free RPMI or cell lysate was adjusted to pH 4.5 with 10% HCl $_{(aq)}$. After the protein precipitates were removed by centrifugation (26000 RCF, 5 min), the supernatants were treated with 2 µL of the aniline stock solution (1 M in DMSO) and 2 µL of the probe stock solution (**Tz-ONH₂ (2)** or **D-Tz-ONH₂ (4)**, 10 mM in DMSO) at 37 °C for 16 h and subjected to the following click reaction.

4.6 Experimental methods for the click reaction when labeling metabolites

For the samples labeled with tetrazine probes (compounds **1**, **2**, **3** and **4**), after labeling, the solutions were then treated with 2 µL of **TCO-SS-biotin (5)** stock solution (10 mM in DMSO). In the relative quantification experiment, the solutions were treated with 2.2 µL of **TCO-SS-biotin** stock solution (10 mM in DMSO). The reaction mixture was incubated at 37 °C for 3 h in the dark.

For alkyne probes (compounds **10** and **11**), after labeling, 180 µL of the reaction solution was mixed with 2 µL of **azido-SS-biotin (15)** stock solution (10 mM in DMSO). After the addition of the premix solution of 4 µL of $CuSO_4$ stock solution (100 mM in H_2O), 4 µL of tris(benzyltriazolyl methyl)amine (TBTA) stock solution (10 mM in 1:4 DMSO/*t*-BuOH) and freshly prepared 10 µL of sodium ascorbate (NaAsc) stock solution (40 mM in H_2O), the reaction mixture was incubated at 37 °C for 3 h in the dark.

4.7 Quantitative methods of relative reactivity in heatmaps

In the heatmaps depicting the reactions between chemoselective probes and model carbonyls (**Figure 2a**), the color scale of "relative reactivity" was quantified based on the area under the curves (AUCs) from chromatographs. The relative reactivity was determined by the probe consumption time

and the amount of the formed product.

In the heatmaps of reactions involving chemoselective probes or TCI precursors with metabolites (**Figures 2e**, **5e** and **S25b**), the labeling efficiency of different labeled species was quantified using their AUCs of MS intensity integrated from extracted ion chromatographs (EICs) and normalized with the highest AUC in each labeling group to represent relative reactivity. Specifically, in the **Tz-PPh$_3$** labeling groups, the AUCs were normalized to acetaldehyde in the lysate (**Figure 2e**, Column 4, A2), and in the **Tz-ONH$_2$** labeling groups, the AUCs were normalized to pyruvic acid in media (**Figure 2e**, Column 1, K1). In the **IbrPPh$_3$-yne (10)** labeling groups, the AUCs were normalized to the acetaldehyde in the lysate (**Figure 5e**, Column 2, A2), and in the **SpePPh$_3$-yne (11)** labeling groups, the AUCs were normalized to the acetaldehyde in the lysate (**Figure S25b**, Column 2, A2).

5. Sample Preparations, Purifications and Analysis Methods for Metabolome Experiments

5.1 Cell culture

The cells were grown at 37 °C under a humidified 5% CO_2 atmosphere in RPMI 1640 medium. The media were supplemented with 1% penicillin-streptomycin (PS) and 10% fetal bovine serum (FBS).

5.2 Preparations of samples for metabolite labeling experiments

In the experiment using tetrazine probes (compounds **1**, **2**, **3** or **4**) for labeling, 5 × 10^7 Ramos cells in 5 mL of serum-free RPMI were seeded in a 24-well cell culture plate (1 mL per well) for 24 h. Then, the cells were transferred to five different microtubes (each containing 1 mL of cells) and spun down by centrifugation (1000 RCF, 3 min) to yield cell pellets. The supernatant was removed, leaving only one microtube containing 500 µL of medium. This microtube was gently vortexed, and the cells were lysed through a freeze–thaw process. Next, the lysate was transferred to another microtube containing a cell pellet, where it was lysed again. After the cell pellets in the five microtubes were all lysed, the resulting lysates containing 5 × 10^7 cells in 500 µL of medium were centrifuged at 26000 RCF for 20 min to yield a soluble proteome and metabolome.

In the experiments using **IbrPPh$_3$-yne (10)** or **SpePPh$_3$-yne (11)** for labeling, 1 × 10^7 Ramos cells in 1 mL of serum-free RPMI were seeded in a 24-well cell culture plate for 24 h. Then, the cells were transferred to a microtube, and were lysed through a freeze–thaw process. The lysate was centrifuged at 26000 RCF for 20 min to yield a soluble proteome and metabolome.

5.3 Biotin-streptavidin affinity purification

The biotin-streptavidin enrichment process was performed using Poly-Prep® chromatography columns (5 mL, Bio-Rad). In the relative quantification experiment, the enrichment process was performed in an Eppendorf tube (1.5 mL). After labeling and attaching biotin, the biotinylated samples were loaded onto 50 µL of streptavidin Sepharose resin slurry, which had been prewashed four times with 200 µL of H_2O. In the relative quantification experiment, 100 µL of streptavidin resin slurry was used. The mixture was incubated at 37 °C for 1 h with gentle shaking to ensure that the resins were evenly suspended in the solution. Then, the resins were washed four times with 200 µL of H_2O. Finally, the biotinylated compounds were eluted by incubation with 200 µL of DTT (50 mM in H_2O) for 3 h at 37 °C. After elution, the resins were washed four times with 200 µL of H_2O. The

combined solution was then lyophilized to afford the eluate powder.

5.4 Liquid-liquid extraction for desalting

The eluate powder from biotin-streptavidin affinity purification was dissolved in 50 µL of sat. $MgSO_{4(aq)}$ and 200 µL of ACN. After vigorously pipetting and spinning the solution, the ACN layer (upper layer) was collected. The $MgSO_{4(aq)}$ layer was then extracted with ACN, and the resulting ACN layer was combined with the previous layer. The combined ACN layer was further extracted once with sat. $MgSO_{4(aq)}$, and the resulting ACN layer was collected and concentrated using a centrifugal evaporator. The obtained solid residue was dissolved in 100 µL of 1:1 H_2O/MeOH and analyzed by LC–MS.

5.5 Estimation methods for RCS relative quantification

The observed light/heavy MS patterns of a given labeled species, i.e., IS, A1 or unknown 4 (unk4), between two cell lines resulted from the combinations of light and heavy MS patterns mixed in a certain ratio to be determined. In other words, any given mixed light/heavy MS pattern can be expressed as a linear interpolation of the extracted light and heavy MS patterns of the eluted acetaldehyde (A2) products labeled by **Tz-PPh₃ (1)** or **D-Tz-PPh₃ (3)** (**Figure S18a**).

More specifically, the intensity of each isotope (dn, n=0, 1-4) peak (I_{dn}^{obs}) in observed mixed light/heavy MS patterns can be expressed as a linear combination of the corresponding light isotope peak (I_{dn}^{light}) and heavy isotope peak (I_{dn}^{heavy}) using weighting factors *a* and *b*, respectively.

$$I_{\mathrm{d0}}^{obs} = aI_{\mathrm{d0}}^{light} + bI_{\mathrm{d0}}^{heavy}$$
$$I_{\mathrm{dn}}^{obs} = aI_{\mathrm{dn}}^{light} + bI_{\mathrm{dn}}^{heavy}$$

Therefore, by solving these simultaneous linear equations, the weighting factors *a* and *b* can be obtained. Moreover, the light/heavy ratio of any given mixed MS pattern was estimated as *a*/*b*. By setting "Ramos cells" as 1 in all ratios and normalizing to the corresponding IS (**Tz-AAM-Ph (6)** or **D-Tz-AAM-Ph (7)**) in each "mixed" sample, these normalized relative amounts of their corresponding labeled species can be compared across different cell types.

Typically, for most labeled species, the isotope peaks with the highest intensities, d0 (light) and d2 (heavy), were used for calculations. However, for labeled unknown3 (unk3), due to high standard deviations among triplicates when using only d0 and d2 for calculation, the ratios were estimated from d0 and dn (n=1-4).

6. TCI Generation Experiments

6.1 Methods for clickable TCI generation experiments

1×10^6 Ramos cells in 1 mL of medium were seeded in a 24-well cell culture plate. Then, the cells were treated with the indicated concentrations of compounds and incubated at 37 °C for 18 h under a humidified 5% CO_2 atmosphere.

In competitive experiments, cells were first treated with the indicated concentration of Ibr or Spe or DMSO (final=0.1%) at 37 °C for 1 h. In "co-treatment" experiments, cells were sequentially treated with the indicated concentration of **IbrPPh₃-yne (10)** or **SpePPh₃-yne (11)** and RCS (FA, MGO and GA, each 5 mM in H_2O, final conc.=each 10 µM) or **AldN₃ (16)**. In "pre-assembly" experiments, 16 µL of PBS (pH 7.4) with 26 mM $NaHCO_3$, 1 µL of **IbrPPh₃-yne** stock solution (5 mM in DMSO) and

3 µL of **AldN$_3$** stock solution (10 mM in DMSO) were added and mixed sequentially. After incubation at 37 °C for 1 h, the crude solution was diluted to the indicated concentrations and added to the cells (**Figures 5b, 5c** and **S25a**).

After treatment, the cells were harvested, washed with PBS (pH 7.4), and lysed with RIPA lysis buffer A with intermittent vortex mixing (vortexing for 10 s, followed by incubation on ice for 10 min, for 4 cycles in total). The lysate was centrifuged at 26000 RCF for 20 min to yield a soluble proteome. Ramos cell lysates were quantified by Bradford protein assay.

30 µL of the cell lysate (~containing 20 µg of total protein) was treated with a premix solution of 1 µL of **azido-SulfoCy5.5 (12)** or **alkynyl-SulfoCy5.5 (13)** stock solution (0.5 mM in DMSO), 1 µL of $CuSO_4$ stock solution (2.5 mM in H_2O), 5 µL of TBTA stock solution (2.5 mM in 1:4 DMSO/*t*-BuOH) and 3 µL of H_2O. After 10 µL of the freshly prepared NaAsc stock solution (50 mM in H_2O) was added, the lysate was incubated at 37 °C for 1 h in the dark. The proteins were precipitated with MeOH/$CHCl_3$ and stored at -80 °C. For in-gel fluorescence, proteins dissolved in 8 M urea and 1× SDS-PAGE loading buffer were resolved by 12% SDS-PAGE and detected by fluorescence.

6.2 Methods for double-clickable ibrutinib derivative experiments

3 × 10^6 Ramos cells in 3 mL of medium were seeded in a 24-well cell culture plate. With RCS competition, 21.75 µL of PBS (pH 7.4) with 26 mM $NaHCO_3$, 3 µL of **IbrPPh$_3$-yne (10)** stock solution (2.5 mM in DMSO), 0.75 µL of FA stock solution (5 mM in H_2O), 0.75 µL of MGO stock solution (5 mM in H_2O), and 0.75 µL of GA stock solution (5 mM in H_2O) were assembled at 37 °C for 1 h. Then, 3 µL of **AldN$_3$ (16)** stock solution (7.5 mM in DMSO) was added, and the solution was incubated at 37 °C for an additional 1 h. Without RCS competition, 24 µL of PBS (pH 7.4) with 26 mM $NaHCO_3$, 3 µL of **IbrPPh$_3$-yne** stock solution (2.5 mM in DMSO) and 3 µL of **AldN$_3$** stock solution (7.5 mM in DMSO) were assembled at 37 °C for 1 h (**Figures 5d** and **S21**).

After the cells were lysed, 191 µL of the cell lysates (~containing 400 µg of total proteins) were first blocked with 8 µL of the freshly prepared IAA stock solution (1 M in H_2O) at 37 °C for 1 h in the dark and were treated with 1 µL of **DBCO-SS-biotin (14)** stock solution (10 mM in DMSO) at 37 °C for 2 h. After IAA blocking and SPAAC, 198 µL of the solution was then treated with the premix solution of 12.5 µL of **azido-SulfoCy5.5 (12)** stock solution (1 mM DMSO), 5 µL of $CuSO_4$ stock solution (100 mM in H_2O), 5 µL of TBTA stock solution (10 mM in 1:4 DMSO/*t*-BuOH), 19.5 µL of H_2O and 10 µL of the freshly prepared NaAsc stock solution (50 mM in H_2O). Then, the lysates were incubated at 37 °C for 1 h in the dark. The proteins were precipitated with MeOH/$CHCl_3$, and one-eighth of the precipitated proteins were stored as "input". Other portions of proteins were dissolved in 100 µL of 2% SDS in PBS and then diluted to 0.2% SDS by adding 900 µL of PBS. The soluble proteins were enriched with streptavidin Sepharose to obtain "flow-through" and "output" samples. After enrichment, proteins from "input", "flow-through" and "output" samples were dissolved in 8 M urea and 1× SDS-PAGE loading buffer, resolved by 12% SDS-PAGE, and detected by fluorescence.

6.3 Methods for ROS modulation experiments

2 × 10^6 Ramos cells in 2 mL of medium were seeded in a 24-well cell culture plate. In the ROS trigger experiments (**Figure S23a**), freshly prepared $FeSO_4$ or H_2O_2 (final conc.=1 mM) was applied to the cells for 1 h. In the ROS quencher experiments (**Figure S23b**), freshly prepared NaAsc (final

conc.=0.2 mM), pyruvic acid (final conc.=5 mM), or glutathione (final conc.=10 mM) was applied to the cells for 24 h. After treatment, the cells were spun down, and the supernatant medium was removed and replaced with PBS (pH 7.4) for washing. The cells were spun down again, and after the removal of PBS, medium with the indicated concentration of **IbrPPh$_3$-yne (10)** was added to the cells. After incubation at 37 °C for 18 h under a humidified 5% CO_2 atmosphere, the cells were lysed and clicked with **azido-SulfoCy5.5 (12)**. Then, the proteins were detected by in-gel fluorescence and western blotting.

6.4 Western blotting

The proteins were transferred to a PVDF membrane, incubated with Blocking Buffer at rt for 1 h and probed with primary antibody (anti-BTK, 1000× dilution; anti-GAPDH, 2000× dilution) at 4 °C overnight. After being washed with Washing Buffer (10 min each time, for three times in total), the membrane was probed with secondary antibodies (5000× dilution) at rt for 1 h. After being washed with Washing Buffer (10 min each time, three times in total), the membrane was detected using ECL.

7. Cytotoxicity Experiments with the AlamarBlue Cell Viability Assay

In the cytotoxicity experiments, 6x10^4 Ramos cells in 0.1 mL of serum-free RPMI were seeded in a 96-well cell culture plate and treated with serial concentrations of the indicated compounds. For the control group, cells were treated with 1% DMSO. After incubation at 37 °C for 48 h under a humidified 5% CO_2 atmosphere, the cells were treated with 10 µL of prewarmed resazurin sodium solution (0.01 mg/mL in PBS) and incubated at 37 °C for 4 h in the dark. After the fluorescence signals were measured (excitation/emission=544/590 nm from the top with a gain of 60), the IC_{50} was calculated by using OriginLab software.

For the **IbrPPh$_3$+FA** or **SpePPh$_3$+FA** sample, 6 µL of PBS (pH 7.4) with 26 mM $NaHCO_3$, 1 µL of **IbrPPh$_3$ (8)** or **SpePPh$_3$ (9)** stock solution (10 mM in DMSO) and 3 µL of FA stock solution (5 mM in H_2O) were added and mixed sequentially. For the **IbrPPh$_3$+RCS** or **SpePPh$_3$+RCS** sample, 6 µL of PBS (pH 7.4) with 26 mM $NaHCO_3$, 1 µL of FA stock solution (5 mM in H_2O), 1 µL of MGO stock solution (5 mM in H_2O), 1 µL of GA stock solution (5 mM in H_2O) and 1 µL of **IbrPPh$_3$** or **SpePPh$_3$** stock solution (10 mM in DMSO) were added and mixed sequentially. After incubation at 37 °C for 1 h, the crude solution was diluted to the indicated concentration and added to the cells.

8. Protocols and Analysis Methods for Proteome Experiments

8.1 Cell treatment

3 × 10^6 Ramos cells in 3 mL of medium were seeded in a 24-well cell culture plate (1.5 mL per well). For competitive experiments, cells were first treated with Ibr (1 mM DMSO stock, final conc.=1 µM) or DMSO (final=0.1%) at 37 °C for 1 h. Then, the cells were treated with **IbrPPh$_3$-yne (10)** preassembled with RCS (final conc.=2.5 µM) at 37 °C for 18 h under a humidified 5% CO_2 atmosphere. For the pre-assembly of **IbrPPh$_3$-yne** with RCS, 3 µL of PBS with 26 mM $NaHCO_3$, 2 µL of FA stock solution (5 mM in H_2O), 2 µL of MGO stock solution (5 mM in H_2O), 2 µL of GA stock solution (5 mM in H_2O) and 1 µL of **IbrPPh$_3$-yne** stock solution (10 mM in DMSO) were mixed sequentially and incubated at 37 °C for 1 h.

Then, the Ramos cells were harvested, washed with PBS (pH 7.4), and lysed. 15 µL of the cell

lysates (~containing 50 µg of total protein) were treated with a premix solution of 1 µL of **azido-SulfoCy5.5 (12)** stock solution (0.5 mM in DMSO), 0.5 µL of $CuSO_4$ stock solution (2.5 mM in H_2O), 2.5 µL of TBTA stock solution (2.5 mM in 1:4 DMSO/*t*-BuOH) and 1.5 µL of H_2O. After 5 µL of the freshly prepared NaAsc stock solution (50 mM in H_2O) was added, the reaction mixture was incubated at 37 °C for 1 h in the dark. The proteins were precipitated with MeOH/$CHCl_3$ and stored as input.

180 µL of the cell lysates (~containing 600 µg of total protein) were treated with 4 µL of **azido-SS-biotin (15)** stock solution (10 mM in DMSO). After the premix solution of 4 µL of $CuSO_4$ stock solution (100 mM in H_2O), 4 µL of TBTA stock solution (10 mM in 1:4 DMSO/*t*-BuOH) and freshly prepared 8 µL of NaAsc stock solution (50 mM in H_2O) were added, the reaction mixture was incubated at 37 °C for 1 h in the dark. The proteins were precipitated with MeOH/$CHCl_3$. The precipitated proteins were dissolved in 100 µL of 2% SDS in PBS and then diluted to 0.2% SDS by adding 900 µL of PBS. The soluble proteins were enriched with streptavidin Sepharose to obtain "flow-through" and "output" samples. After enrichment, proteins from "input", "flow-through" and "output" samples were subjected to in-gel fluorescence and western blotting.

8.2 Biotin-streptavidin affinity purification

The biotin-streptavidin enrichment process was performed in an Eppendorf tube (1.5 mL). The solution with biotinylated labeled proteins was loaded onto a streptavidin Sepharose resin slurry, which was prewashed with 200 µL of PBS four times. After incubation at 4 °C overnight with end-over-end rotation, the resins were spun down (1000 RCF, 5 s), and the supernatant was collected. Then, the resins were sequentially washed with different ice-cold buffers (1 mL of RIPA lysis buffer B twice; 1 mL of KCl, 1 M in H_2O; 1 mL of $NaHCO_3$, 0.1 M in H_2O; 1 mL of urea, 2 M in 10 mM Tris-HCl (pH 8.0); and 1 mL of RIPA lysis buffer B w/ Triton X-100 twice). All the supernatants collected from the washing process were combined as "flow-through". After the washing process, the biotinylated compounds were eluted by incubation with DTT (4% in 1× sampling buffer) at rt for 1 h and incubation with 1% formic acid at rt for 0.5 h. All the supernatants collected from the elution process were combined as the "output" sample.

The "flow-through" and "output" samples were then precipitated with TCA for western blotting. After confirming that enrichment was effective, proteins precipitated from the eluate were sent to Mass Spectrometry service for the subsequent reduction, alkylation, trypsin digestion and LC–MS/MS analysis.

8.3 LC–MS/MS analysis

Desalted peptides were analyzed on an Orbitrap Fusion Lumos Tribrid quadrupole-ion trap-Orbitrap mass spectrometer (Thermo Fisher Scientific) coupled to an Ultimate system 3000 nanoLC system (Thermo Fisher Scientific) with a C18 Acclaim PepMap NanoLC column (2 µm, 100 Å, 75 µm i.d. × 25 cm) (Thermo Fisher Scientific). The samples were separated using a 50 min segmented gradient from 2% to 40% solvent B in solvent A (solvent A: 0.1% formic acid in H_2O; solvent B: 0.1% formic acid in ACN) with a flow rate of 300 nL/min. MS1 analysis was performed in a data-dependent mode with Full-MS (mass accuracy of < 5 ppm, resolution of 120,000 at m/z=200, AGC target 5e5). MS/MS fragmentation (resolution of 15,000) was performed to the most intense ions in 3 s using higher-energy collisional dissociation (HCD). The isolation width was set to 1.4 Da, the normalized

collision energy was set to 32%, the AGC target was set to 5e4, and the dynamic exclusion time was set to 60 s. Max injection time was set as 50 ms.

8.4 MS data analysis and peptide identification

LC–MS/MS data were analyzed and quantified with MaxQuant software version 2.1.4.0 with default settings. Carbamidomethyl (cysteine) was selected as the fixed modification, and oxidation (methionine) and acetylation (protein N-term) were selected as variable modifications. The LC–MS/MS data were searched via MaxQuant's Andromeda search engine. The protein identification was searched against the UniProt database (*Homo sapiens*) (downloaded August 11th, 2022). The false discovery rate (FDR) was set to 1% for proteins and peptides. The minimum peptide length was set to seven amino acids. Label-free quantification was performed by the MaxLFQ algorithm in MaxQuant with the default settings.

Database searching was performed on UniProt (https://www.uniprot.org/) and the PANTHER Classification System (http://www.pantherdb.org/). All the searches were set to the defaults, and the websites were visited from October to December in 2023.

9. Stability Evaluation of Triphenylphosphonium Probes

In the stability experiments **(Figure S29** and **S30)**, 99 µL of PBS (pH 7.4) with 26 mM $NaHCO_3$ and 1 µL of the probe stock solution (**Tz-PPh$_3$ (1)**, **D-Tz-PPh$_3$ (3)**, **IbrPPh$_3$ (8)**, **SpePPh$_3$ (9)**, **IbrPPh$_3$-yne (10)** or **SpePPh$_3$-yne (11)**) were mixed, and the solution was incubated at 37 °C. After the given time, 100 µL of the sample was taken, mixed with 100 µL of DMSO and H_2O, and stored at -20 °C immediately. The samples were analyzed by HPLC, and the absorbance at 280 nm was recorded.

10. Supplementary Schemes, Figures and Tables

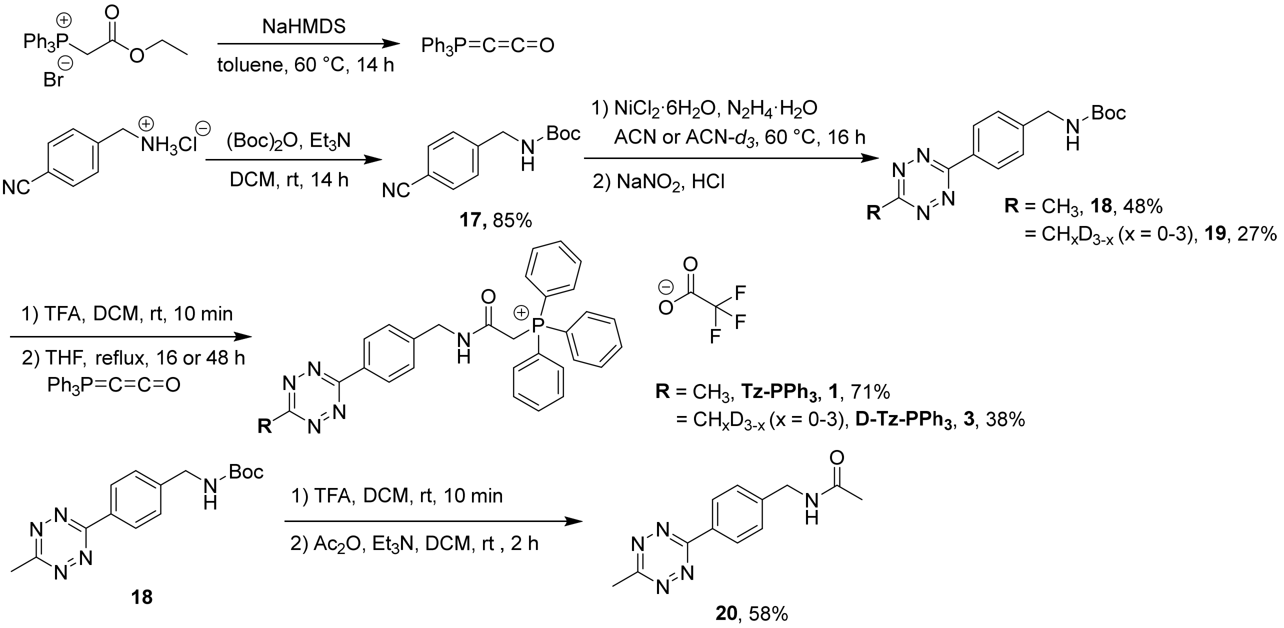

Scheme S1. Synthetic routes of **Tz-PPh₃ (1)**, **D-Tz-PPh₃ (3)**, and the acetamido side product (**20**). The synthesis started from the amine protection of commercial 4-(aminomethyl)benzonitrile hydrochloride using the *tert*-butyloxycarbonyl (Boc) group to afford **17**. Subsequently, the tetrazine group was constructed through the condensation of **17** with acetonitrile (or deuterated acetonitrile, CD_3CN) and hydrazine, yielding intermediate **18** or **19**, respectively. After Boc deprotection, the corresponding free primary amine underwent an amide coupling reaction with freshly prepared Bestmann's ylide to afford **Tz-PPh₃ (1)** or **D-Tz-PPh₃ (3)**. The proposed **Tz-PPh₃** side product was synthesized through acetylation of the deprotection product of **18**.

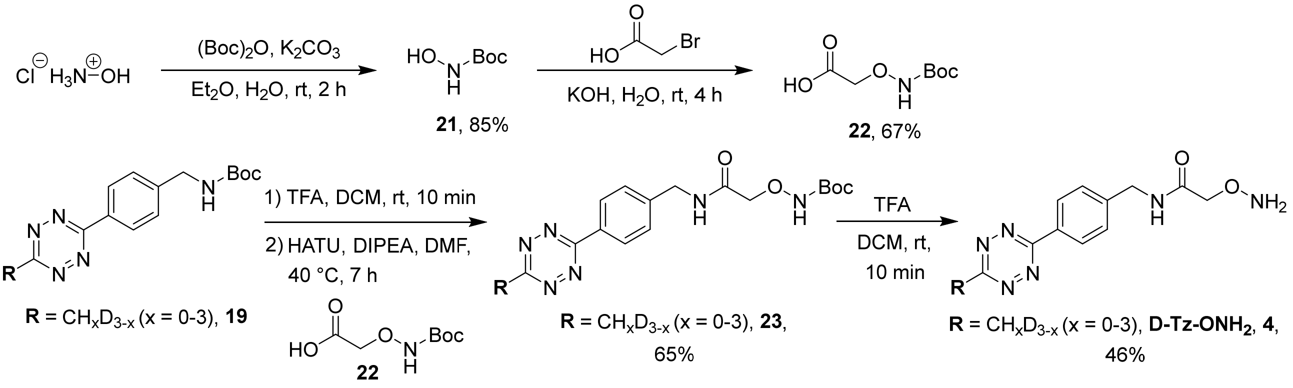

Scheme S2. Synthetic routes of **D-Tz-ONH₂ (4)**. The synthesis started from the amine protection of hydroxylamine hydrochloride using the Boc group to afford intermediate **21**. Subsequent alkylation of **21** with bromoacetic acid yielded **22**. Finally, amide formation between **22** and Boc-deprotected **19**, followed by a second Boc deprotection, afforded **D-Tz-ONH₂ (4)**.

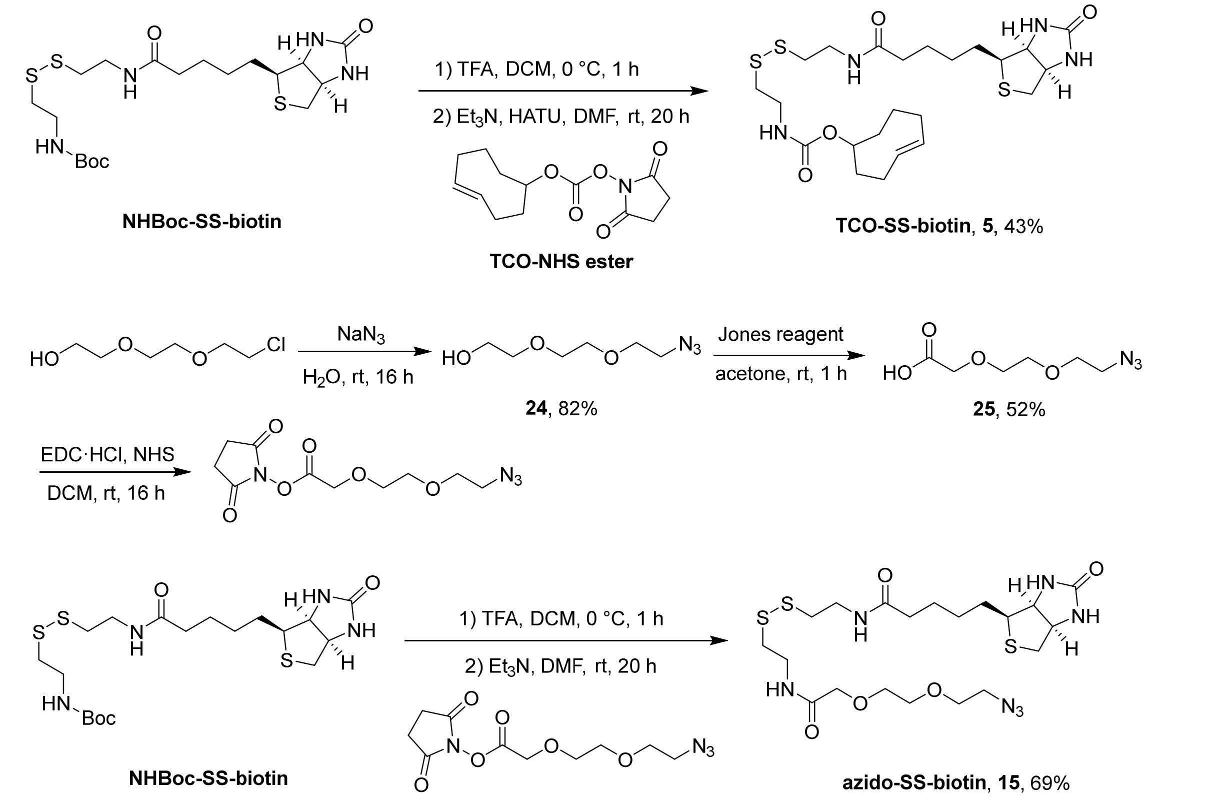

Scheme S3. Synthetic routes of **TCO-SS-biotin (5)** and **azido-SS-biotin (15)**. **TCO-SS-biotin (5)** was obtained from amide formation between the Boc-deprotected **NHBoc-SS-biotin**[7] and the commercial **TCO-NHS ester**. To obtain **azido-SS-biotin (15)**, azido-substituted **24** was oxidized to give **25**, and the desired product was afforded by amide formation between deprotected **NHBoc-SS-biotin** and NHS-activated **25**.

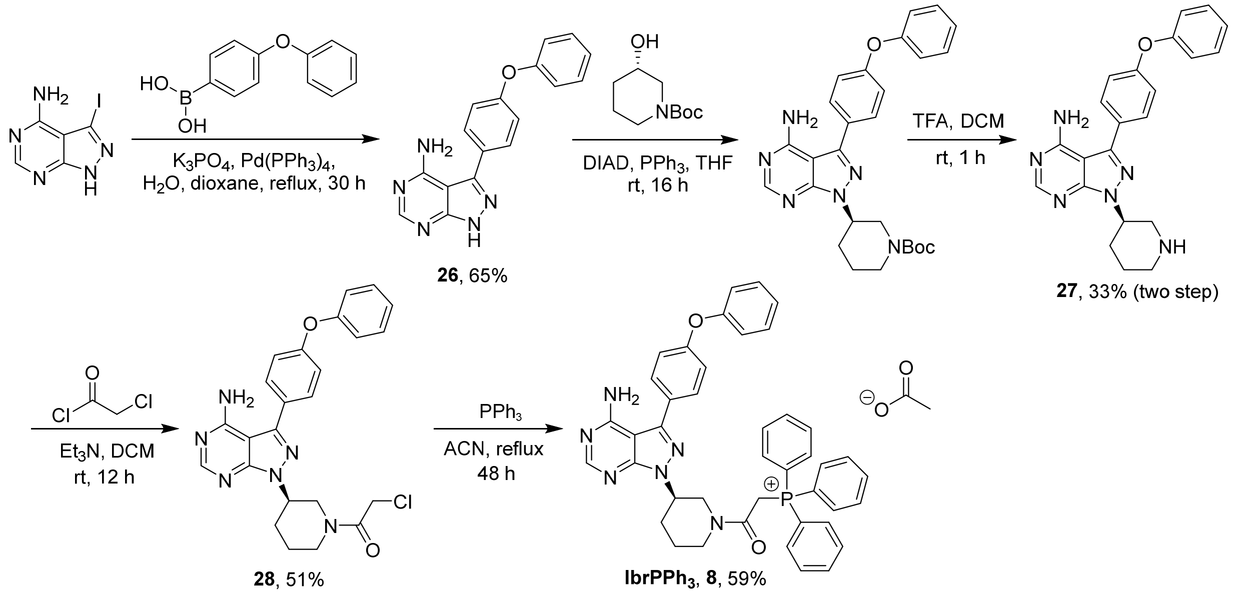

Scheme S4. Synthetic routes of **IbrPPh$_3$ (8)**. We constructed ibrutinib heterocyclic core **26** from 3-iodo-1*H*-pyrazolo[3,4-*d*]pyrimidin-4-amine and 4-phenoxybenzene boronic acid through Suzuki coupling. After the Mitsunobu reaction and Boc deprotection, free secondary amine **27** was obtained. Compound **27** was reacted with chloroacetyl chloride to give **28**, and **IbrPPh$_3$ (8)** was obtained through triphenylphosphine substitution.

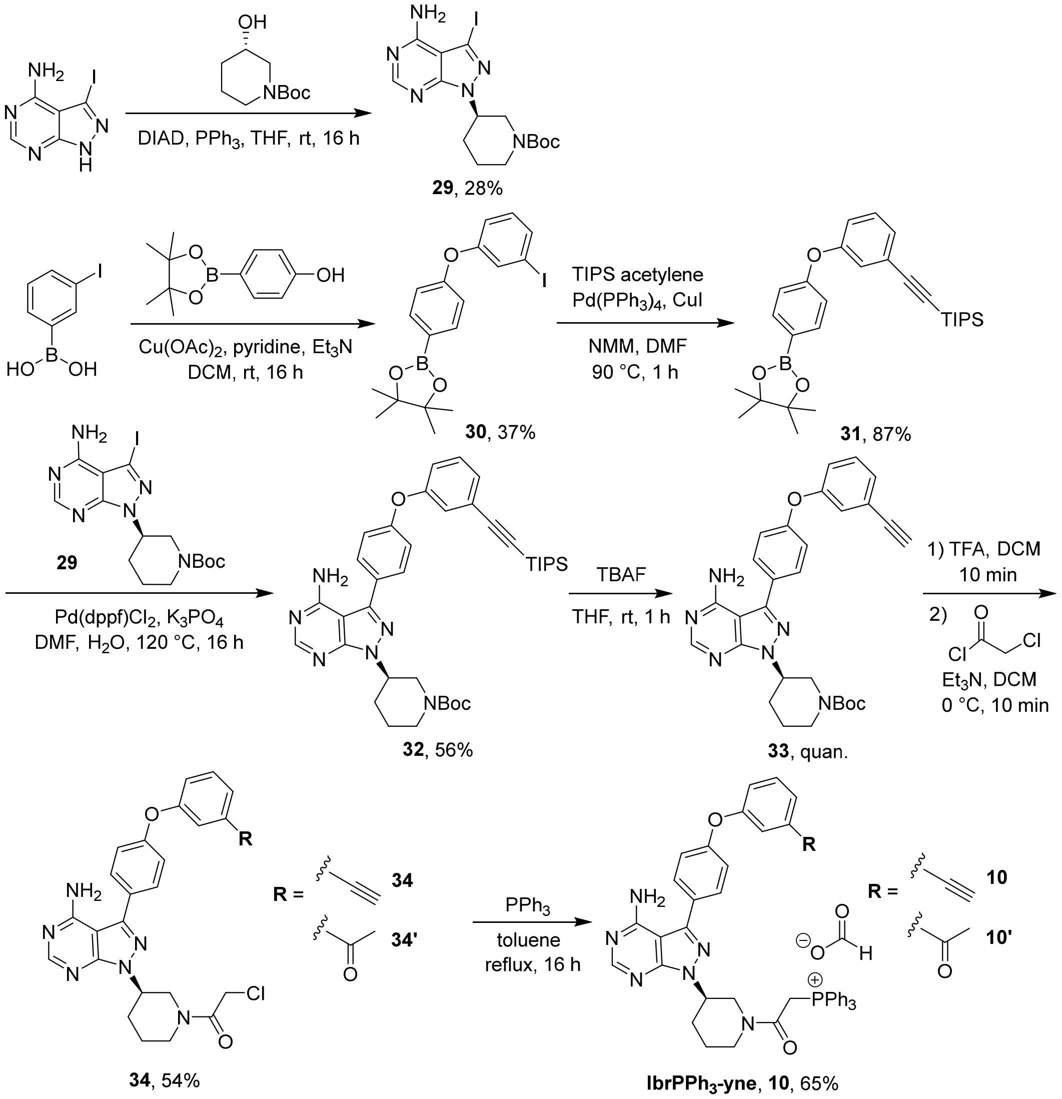

Scheme S5. Synthetic routes of **IbrPPh3-yne (10)**. Chan-Lam coupling between (3-iodophenyl)-boronic acid and 4-(4,4,5,5-tetramethyl-1,3,2-dioxaborolan-2-yl)phenol was used to afford boronic ester **30**. Subsequent Sonogashira reaction introduced an alkynyl group to give boronic ester **31**. The ibrutinib heterocyclic core was then installed through Suzuki coupling between compound **29** (derived from the Mitsunobu reaction) and **31** to afford Boc-protected **32**. Then, the TBAF deprotection removed the triisopropylsilyl ether (TIPS) group in **32** to afford **33**. After Boc deprotection, the resulting free secondary amine from **33** was reacted with chloroacetyl chloride to give **34**. During *N*-acylation, a minor methyl ketone side product **(34')** was formed due to alkyne hydration (**Figure S31**). Subsequently, **IbrPPh3-yne (10)** was obtained through triphenylphosphine substitution. Notably, both **IbrPPh3-yne (10)** (m/z=713 Da) and an unknown +40-Da adduct (m/z=753 Da) were observed after HPLC purification. The +40-Da adduct could transform into the desired product **IbrPPh3-yne (10)** when dissolved in PBS with $NaHCO_3$. Similarly, after triphenylphosphine substitution with **34'**, the corresponding +40-Da adduct (m/z=771 Da) associated with the methyl ketone triphenylphos-phonium product (**10'**) (m/z=731 Da) could also be formed (**Figure S32**). Given the consistent mass loss (-40 Da) and conversion from the +40-Da adducts to give the desired product, we hypothesized that the formation of unknown +40-Da adducts might result from the highly nucleophilic nature of the phosphonium ylide. Solvent molecules might be responsible for these adducts and impurities. Nevertheless, after tedious conversion and purification, **IbrPPh3-yne (10)** can be obtained successfully.

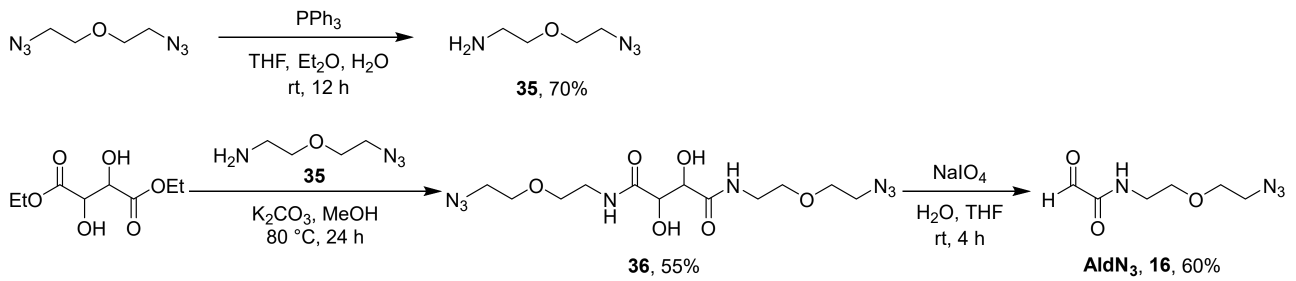

Scheme S6. Synthetic routes of **AldN$_3$ (16)**. The synthesis began with Staudinger azide reduction of 1-azido-2-(2-azidoethoxy)ethane to give linker **35**. Subsequently, tartardiamide **36** was obtained by amide formation between diethyl tartarate and **35**. Finally, the oxidative cleavage of **36** by sodium periodate yielded **AldN$_3$ (16)**.

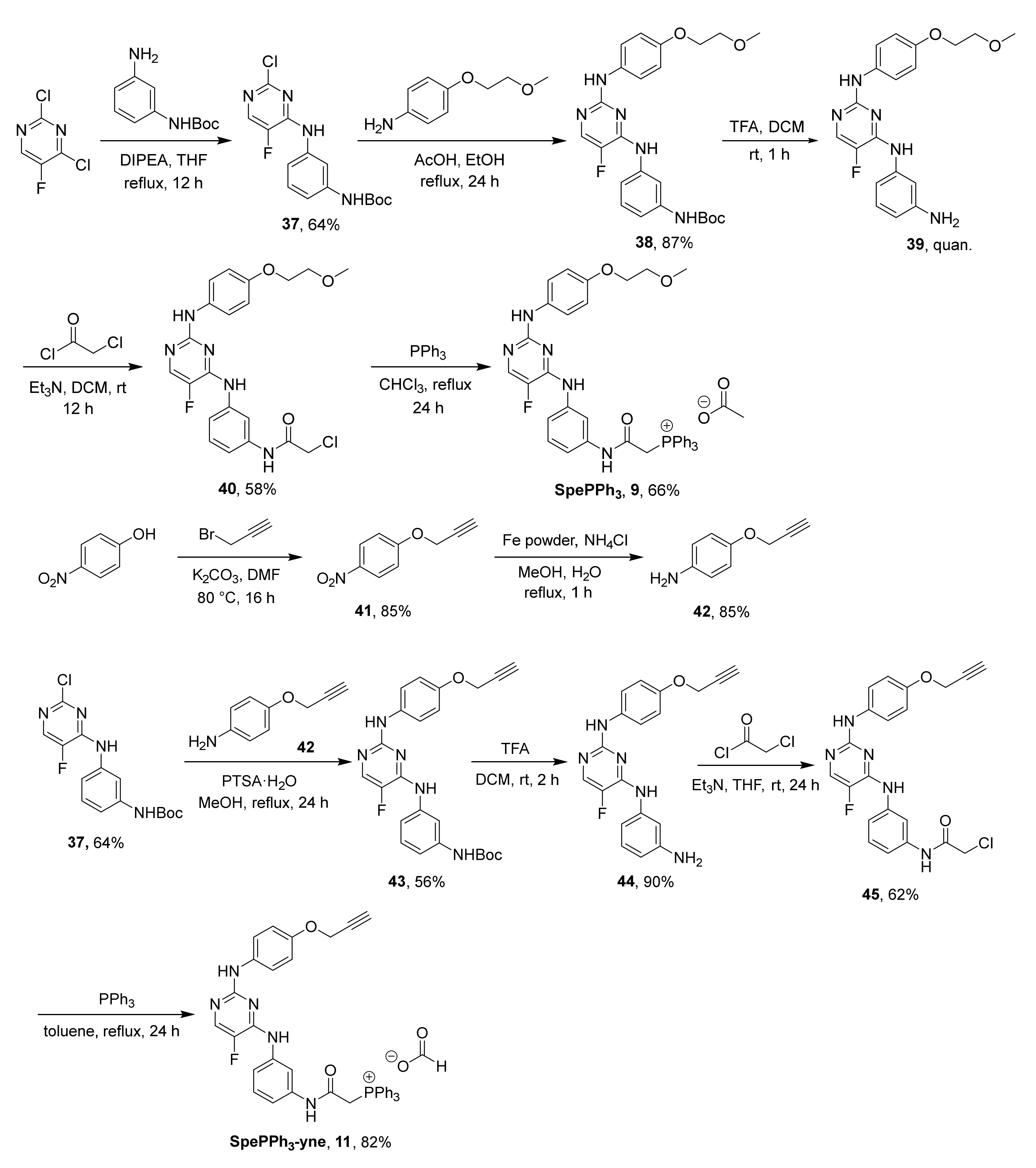

Scheme S7. Synthetic routes of **SpePPh$_3$ (9)** and **SpePPh$_3$-yne (11)**. We constructed the spebrutinib heterocyclic core from 2,4-dichloro-5-fluoropyrimidine through nucleophilic aromatic substitution with *tert*-butyl (3-aminophenyl)carbamate regioselectively at the C-4 position to afford **37**. To introduce the alkynyl click handle, aniline **42** was obtained from the alkylation of 4-nitrophenol by propargyl bromide, followed by the reduction of the nitro group. The second nucleophilic substitution occurred at the C-2 position of the pyrimidine in **37**, either by reacting with 4-(2-methoxyethoxy) aniline or **42** to yield Boc-protected **38** or **43**, respectively. After Boc deprotection, free amine **39** or **44** reacted with chloroacetyl chloride to yield **40** or **45**. Finally, **SpePPh$_3$ (9)** and **SpePPh$_3$-yne (11)** were obtained through triphenylphosphine substitution.

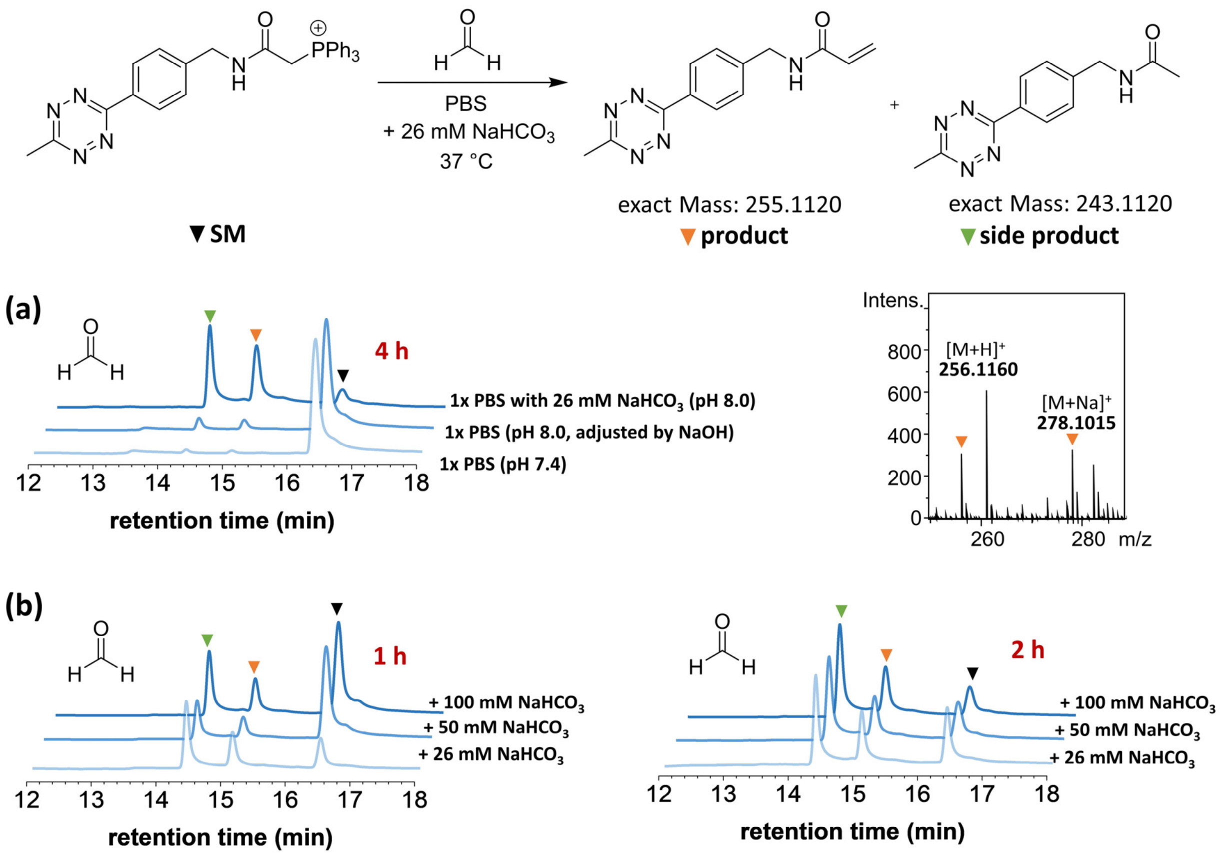

Figure S1. Evaluating the conditions of the aqueous Wittig reaction. (a) Chromatographs of the aqueous Wittig reaction in different buffers. **Tz-PPh₃ (1)** (100 µM) was reacted with FA (200 µM) for 4 h in PBS buffer in the absence or presence of different bases. (b) Chromatographs of the aqueous Wittig reaction with different concentrations of $NaHCO_3$. **Tz-PPh₃** was reacted with FA for 1 and 2 h in PBS buffer with different concentrations of $NaHCO_3$. The starting material (SM), product and side product are labeled with black, orange, and green triangles, respectively. The MS spectrum of the product is shown.

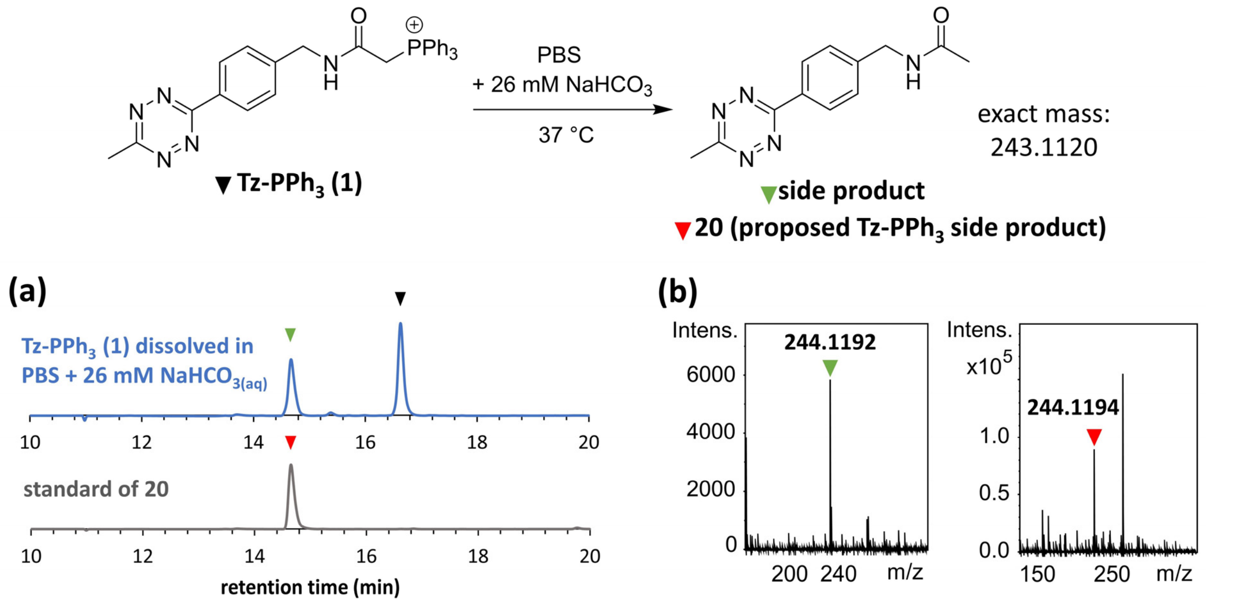

Figure S2. Characterization of the side product observed in PBS (pH 7.4) with $NaHCO_3$. (a) Chromatographs of **Tz-PPh₃ (1)** (100 μM) dissolved in PBS (pH 7.4) with 26 mM $NaHCO_3$ (upper) and a standard of synthesized compound **20** (lower). (b) MS spectra of the reaction side product (left) and synthesized compound **20** (right). **Tz-PPh₃**, the side product and compound **20** are labeled with black, green, and red triangles, respectively.

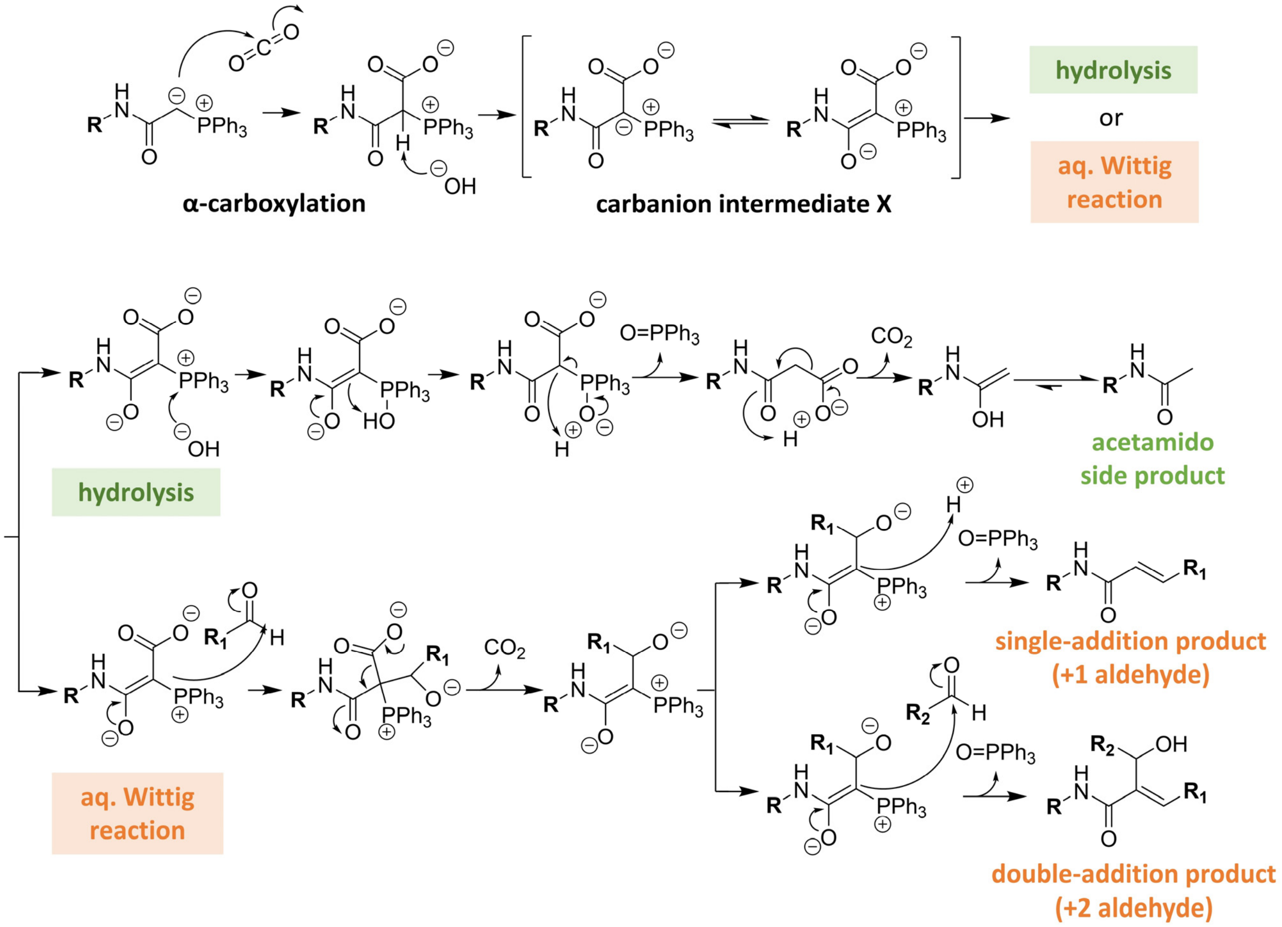

Figure S3. The proposed mechanism of hydrolysis and aqueous Wittig reaction in the presence of $NaHCO_3$.

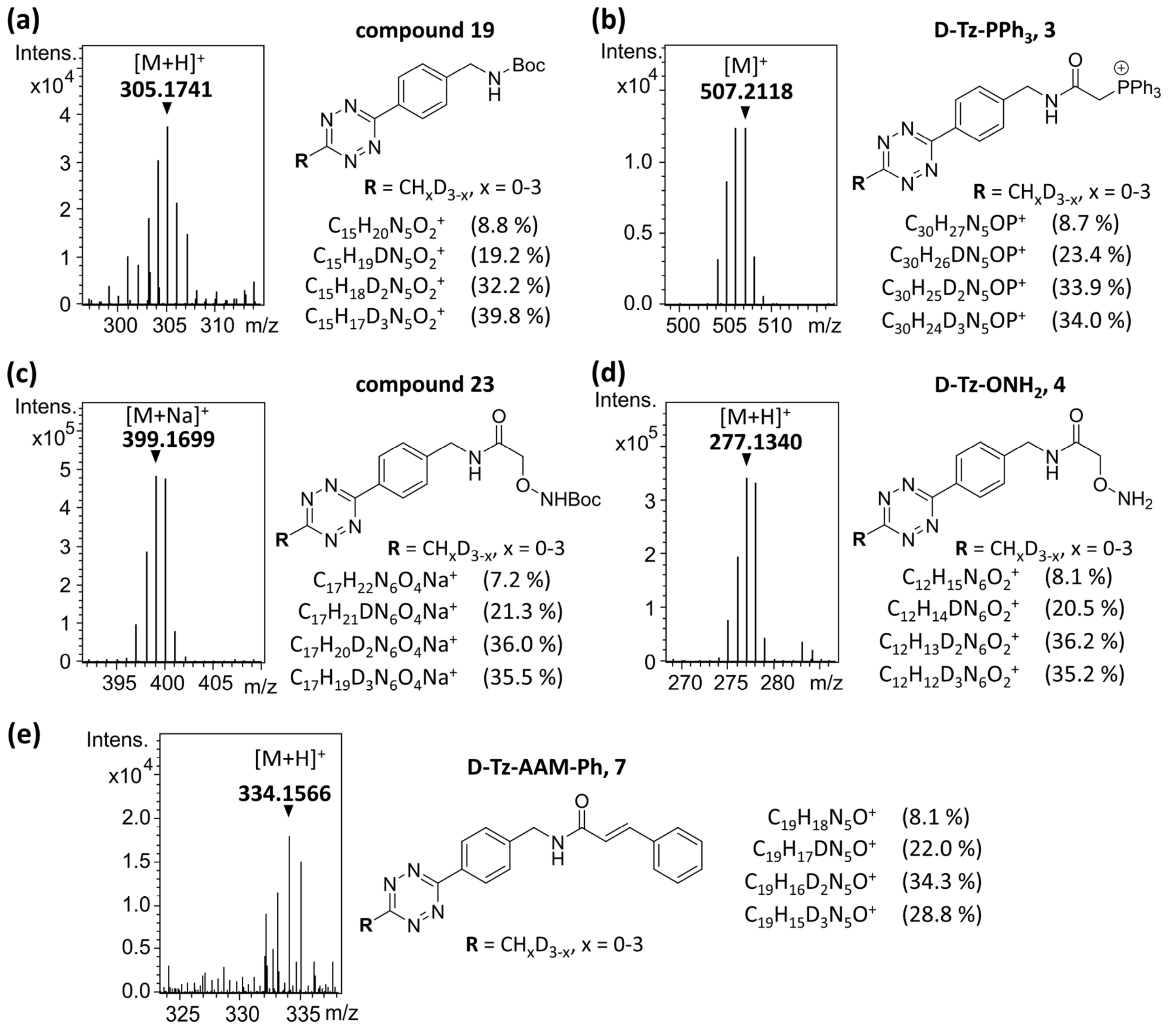

Figure S4. MS isotope patterns. (a) Compound **19**. (b) **D-Tz-PPh₃ (3)**. (c) Compound **23**. (d) **D-Tz-ONH₂ (4)** and (e) **D-Tz-AAM-Ph (7)**.

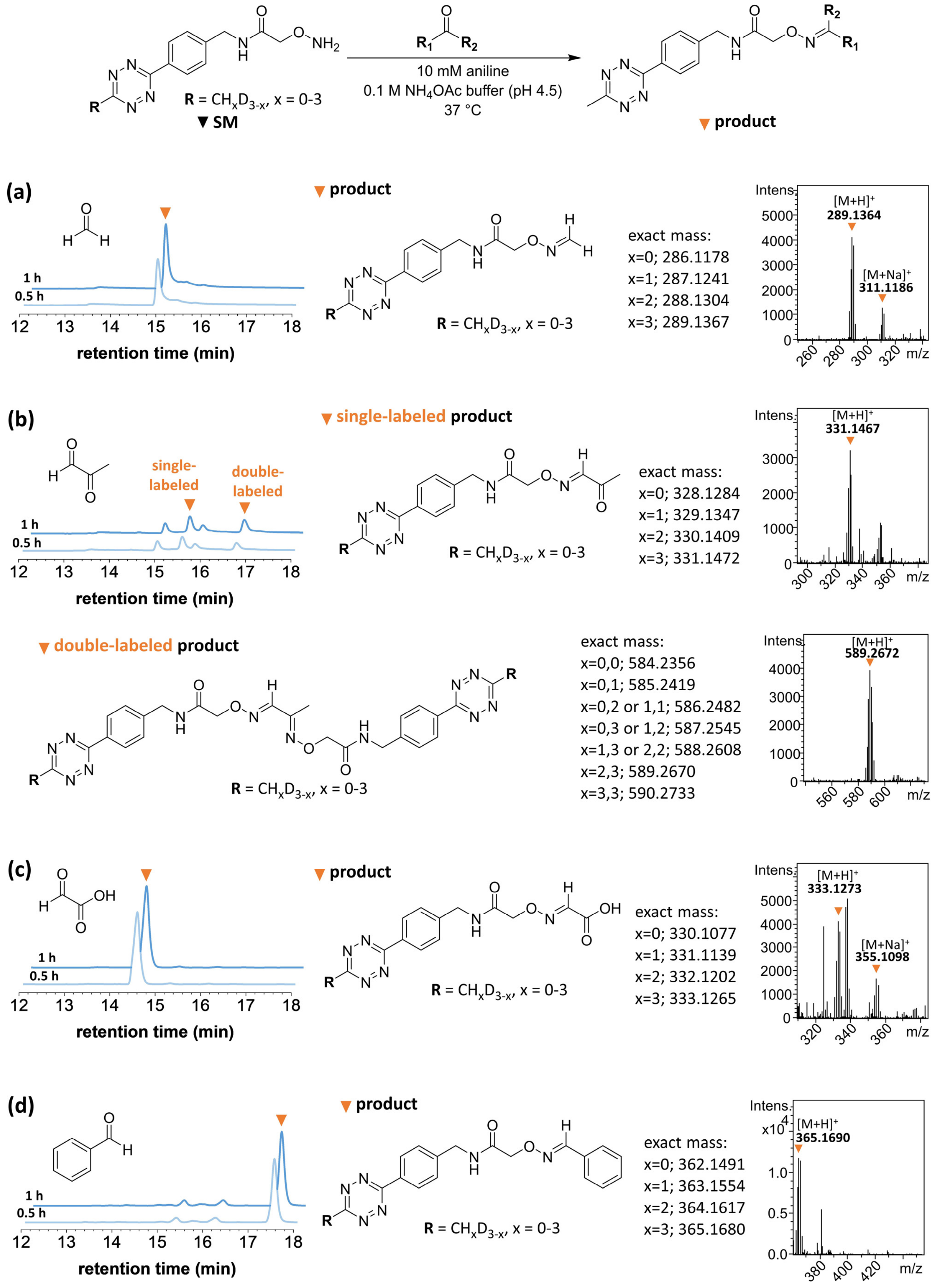

R = CHxD3-x, x = 0-3
▼ SM
10 mM aniline
0.1 M NH4OAc buffer (pH 4.5)
37 °C
▼ product
(a)
1 h
0.5 h
retention time (min)
▼ product
R = CHxD3-x, x = 0-3
exact mass:
x=0; 286.1178
x=1; 287.1241
x=2; 288.1304
x=3; 289.1367
Intens.
[M+H]+
289.1364
[M+Na]+
311.1186
m/z
(b)
single-labeled
double-labeled
1 h
0.5 h
retention time (min)
▼ single-labeled product
R = CHxD3-x, x = 0-3
exact mass:
x=0; 328.1284
x=1; 329.1347
x=2; 330.1409
x=3; 331.1472
Intens.
[M+H]+
331.1467
m/z
▼ double-labeled product
R = CHxD3-x, x = 0-3
exact mass:
x=0,0; 584.2356
x=0,1; 585.2419
x=0,2 or 1,1; 586.2482
x=0,3 or 1,2; 587.2545
x=1,3 or 2,2; 588.2608
x=2,3; 589.2670
x=3,3; 590.2733
Intens.
[M+H]+
589.2672
m/z
(c)
1 h
0.5 h
retention time (min)
▼ product
R = CHxD3-x, x = 0-3
exact mass:
x=0; 330.1077
x=1; 331.1139
x=2; 332.1202
x=3; 333.1265
Intens.
[M+H]+
333.1273
[M+Na]+
355.1098
m/z
(d)
1 h
0.5 h
retention time (min)
▼ product
R = CHxD3-x, x = 0-3
exact mass:
x=0; 362.1491
x=1; 363.1554
x=2; 364.1617
x=3; 365.1680
Intens.
x10^4
[M+H]+
365.1690
m/z

(e)

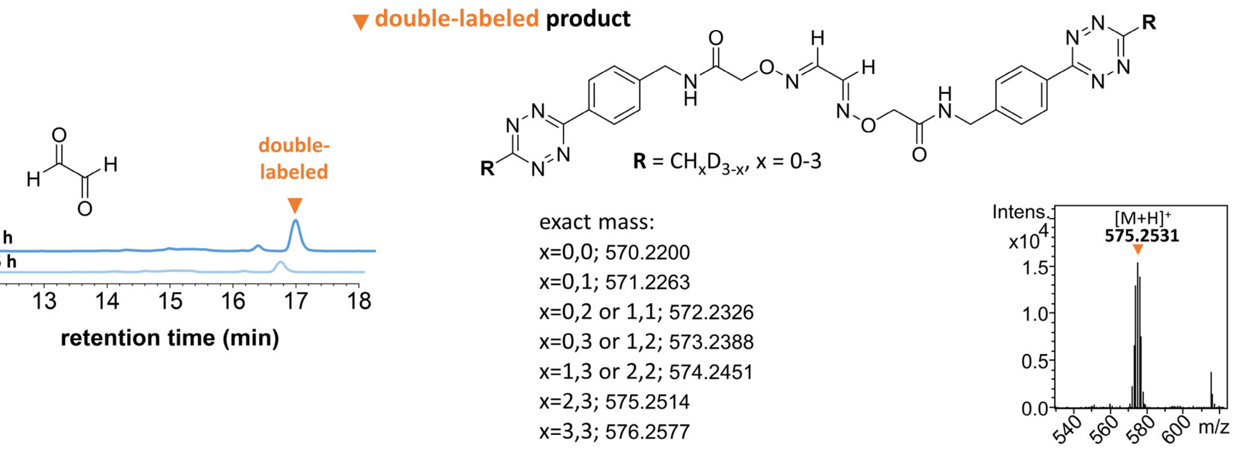
double-labeled product
double-labeled
1 h
0.5 h
retention time (min)
R = CH$_x$D$_{3-x}$, x = 0-3
exact mass:
x=0,0; 570.2200
x=0,1; 571.2263
x=0,2 or 1,1; 572.2326
x=0,3 or 1,2; 573.2388
x=1,3 or 2,2; 574.2451
x=2,3; 575.2514
x=3,3; 576.2577
Intens. x10^4
[M+H]$^+$
575.2531
m/z

(f)

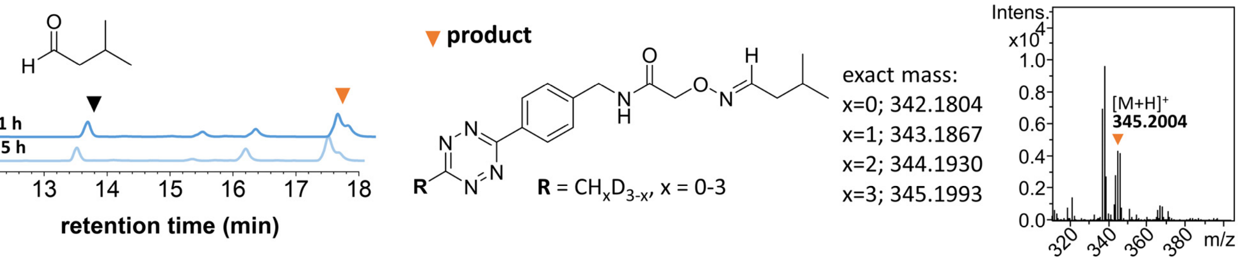
product
1 h
0.5 h
retention time (min)
R = CH$_x$D$_{3-x}$, x = 0-3
exact mass:
x=0; 342.1804
x=1; 343.1867
x=2; 344.1930
x=3; 345.1993
Intens. x10^4
[M+H]$^+$
345.2004
m/z

(g)

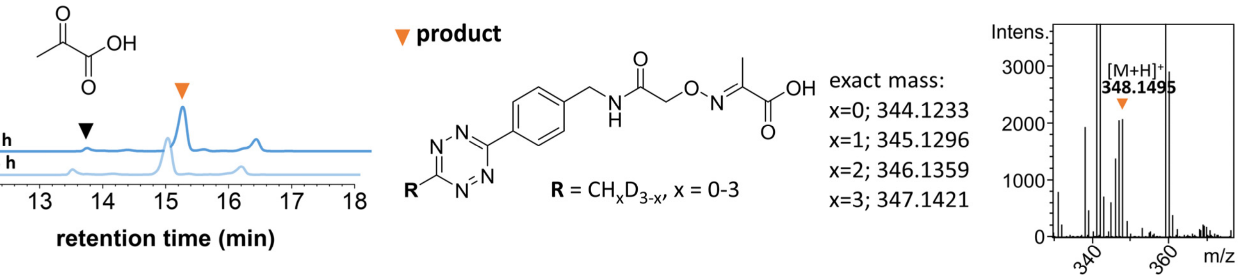
product
1 h
0.5 h
retention time (min)
R = CH$_x$D$_{3-x}$, x = 0-3
exact mass:
x=0; 344.1233
x=1; 345.1296
x=2; 346.1359
x=3; 347.1421
Intens.
[M+H]$^+$
348.1495
m/z

(h)

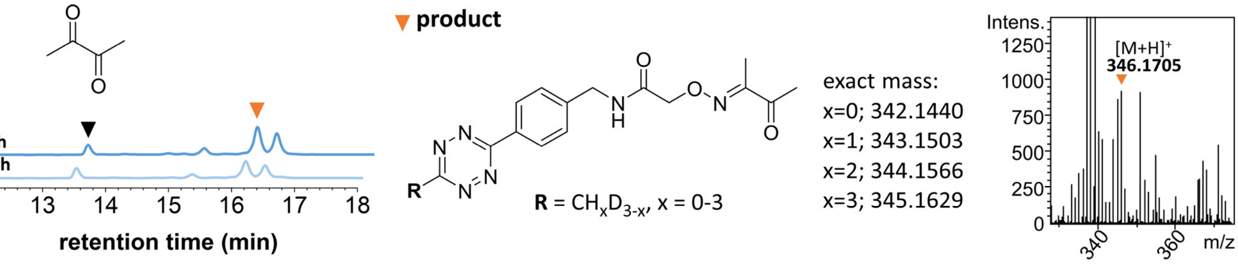
product
1 h
0.5 h
retention time (min)
R = CH$_x$D$_{3-x}$, x = 0-3
exact mass:
x=0; 342.1440
x=1; 343.1503
x=2; 344.1566
x=3; 345.1629
Intens.
[M+H]$^+$
346.1705
m/z

(i)

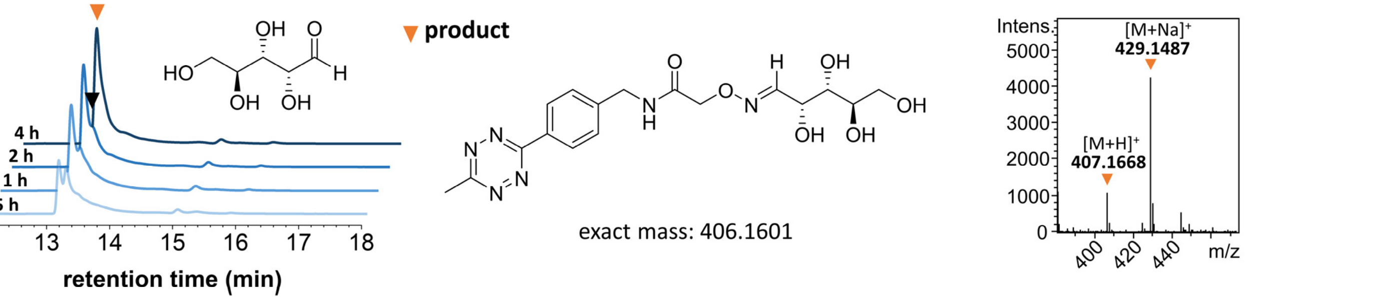
product
4 h
2 h
1 h
0.5 h
retention time (min)
exact mass: 406.1601
Intens.
[M+Na]$^+$
429.1487
[M+H]$^+$
407.1668
m/z

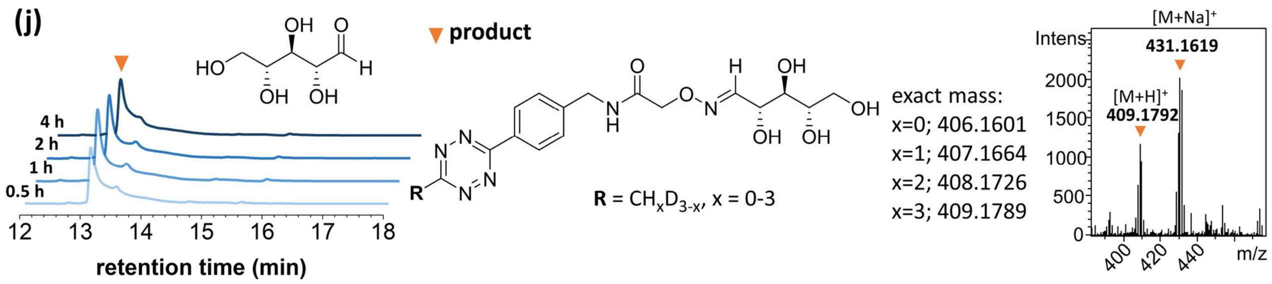

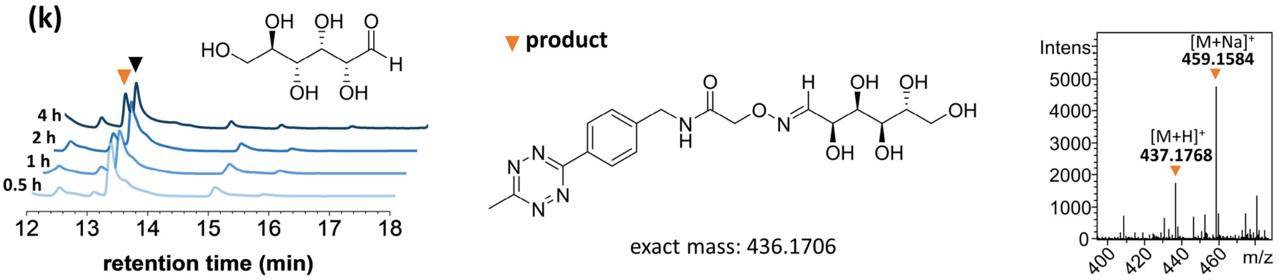

Figure S5. Chromatographs of the reactions between **Tz-ONH$_2$ (2)** or **D-Tz-ONH$_2$ (4)** (100 μM) and several model carbonyls and MS spectra of the relevant products. (a) Formaldehyde (FA). (b) Methylglyoxal (MGO). (c) Glyoxylic acid (GA). (d) Benzaldehyde. (e) Glyoxal. (f) Isovaleraldehyde. (g) Pyruvic acid. (h) Butanedione. (i) Arabinose. (j) Ribose. (k) Glucose. The starting material (SM) and product are labeled with black and orange triangles, respectively. To easily identify the products in MS, **D-Tz-ONH$_2$ (4)** was used in some experiments.

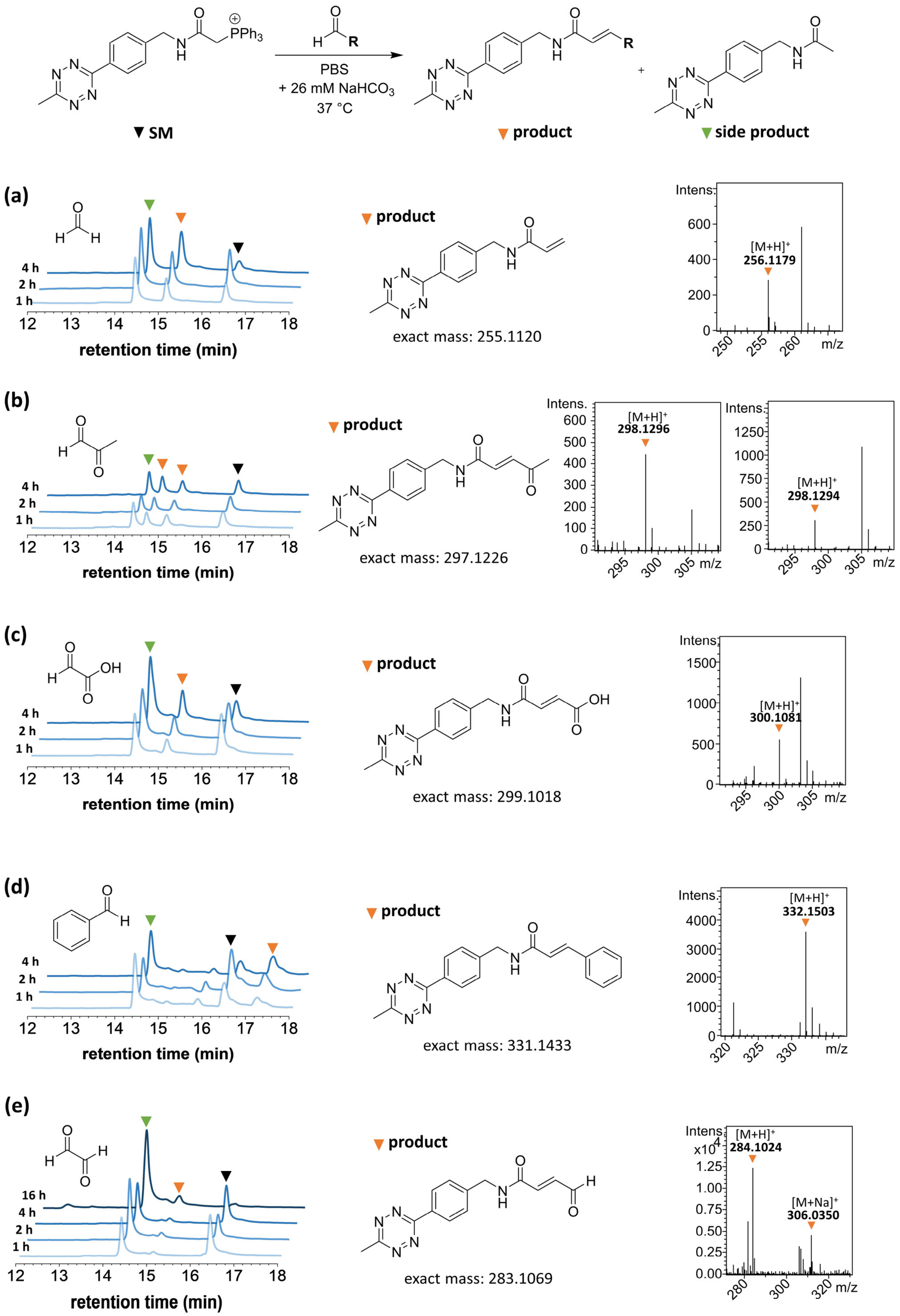

SM
PBS
+ 26 mM NaHCO3
37 °C
product
side product
(a)
4 h
2 h
1 h
retention time (min)
product
exact mass: 255.1120
Intens.
[M+H]+
256.1179
m/z
(b)
4 h
2 h
1 h
retention time (min)
product
exact mass: 297.1226
Intens.
[M+H]+
298.1296
Intens.
[M+H]+
298.1294
m/z
(c)
4 h
2 h
1 h
retention time (min)
product
exact mass: 299.1018
Intens.
[M+H]+
300.1081
m/z
(d)
4 h
2 h
1 h
retention time (min)
product
exact mass: 331.1433
Intens.
[M+H]+
332.1503
m/z
(e)
16 h
4 h
2 h
1 h
retention time (min)
product
exact mass: 283.1069
Intens.
x10^4
[M+H]+
284.1024
[M+Na]+
306.0350
m/z

(f)

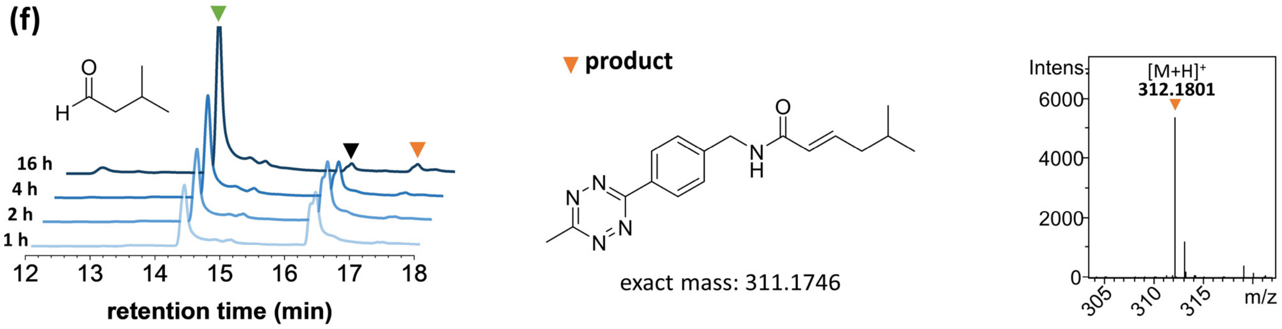

(g)

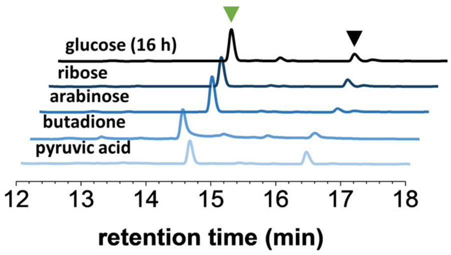

no corresponding products were observed

Figure S6. Chromatographs of the reactions between **Tz-PPh$_3$ (1)** (100 μM) and several model carbonyls and MS spectra of the relevant products. (a) Formaldehyde (FA). (b) Methylglyoxal (MGO). (c) Glyoxylic acid (GA). (d) Benzaldehyde. (e) Glyoxal. (f) Isovaleraldehyde. (g) Pyruvic acid, butanedione, arabinose, ribose, and glucose. The starting material (SM), product and side product are labeled with black, orange, and green triangles, respectively.

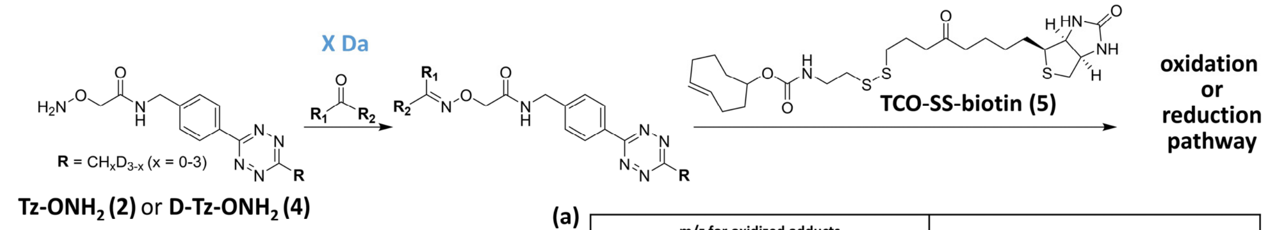

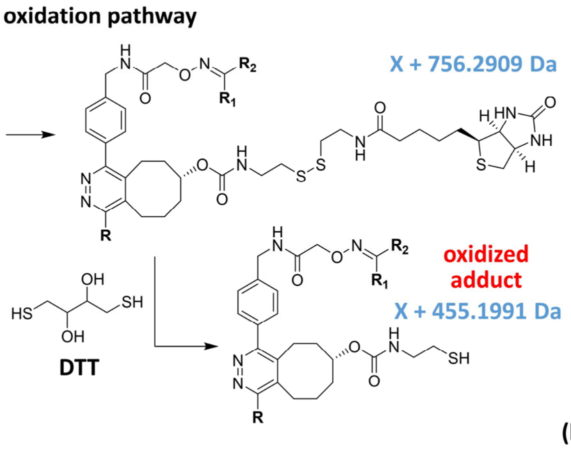

(a)

	m/z for oxidized adducts			potential labeled species (exact mass = X)
	observed ([M+H]$^+$: [X+456.2064]$^+$)		calculated [M+H]$^+$	
	media	Ramos cell lysate		
aldehydes	486.2061	486.2106	486.2170	formaldehyde (**A1**, FA), exact mass = 30.0106
aldehydes	500.2294	500.2231	500.2326	acetaldehyde (**A2**), exact mass = 44.0262
aldehydes	514.2445	514.2455	514.2483	propionaldehyde (**A10**), exact mass = 58.0419
aldehydes	ND	516.2214	516.2275	glycolaldehyde (**A5**), exact mass = 60.0211
aldehydes	530.1955	530.2038	530.2068	glyoxylic acid (**A7**, GA), exact mass = 74.0004
ketones	544.2261	544.2200	544.2225	pyruvic acid (**K1**), exact mass = 88.0160
ketones	560.2085	560.2120	560.2174	hydroxypyruvic acid (**K2**), exact mass = 104.0110
sugars	ND	606.2567	606.2592	pentose (**S1**), exact mass = 150.0528
sugars	636.2574	636.2663	636.2698	hexose (**S2**), exact mass = 180.0634

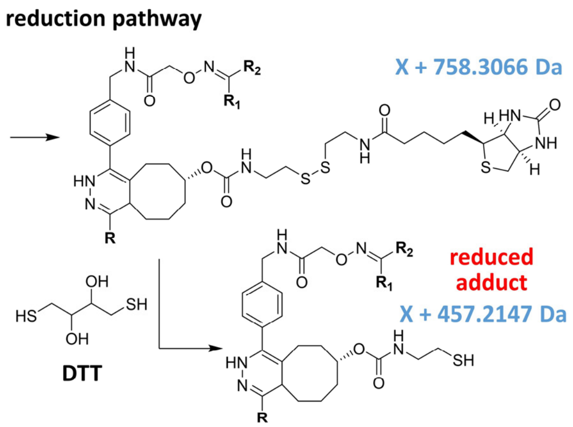

(b)

	m/z for reduced adducts			potential labeled species (exact mass = X)
	observed ([M+H]$^+$: [X+458.2220]$^+$)		calculated [M+H]$^+$	
	media	Ramos cell lysate		
aldehydes	488.2223	ND	488.2326	formaldehyde (**A1**, FA), exact mass = 30.0106
aldehydes	502.2333	502.2359	502.2483	acetaldehyde (**A2**), exact mass = 44.0262
aldehydes	516.2515	516.2576	516.2639	propionaldehyde (**A10**), exact mass = 58.0419
aldehydes	ND	ND	518.2432	glycolaldehyde (**A5**), exact mass = 60.0211
aldehydes	532.1988	ND	532.2225	glyoxylic acid (**A7**, GA), exact mass = 74.0004
ketones	546.2279	546.2304	546.2381	pyruvic acid (**K1**), exact mass = 88.0160
ketones	562.2339	562.2406	562.2330	hydroxypyruvic acid (**K2**), exact mass = 104.0110
sugars	ND	ND	608.2749	pentose (**S1**), exact mass = 150.0528
sugars	638.2715	ND	638.2854	hexose (**S2**), exact mass = 180.0634

Figure S7. Reaction scheme and the summarized labeling results using **H/D-Tz-ONH$_2$ (2/4).** Based on the appearance of both light and heavy isotope patterns at the same retention time in the light/heavy labeled chromatographs, the labeled peaks were first identified as either the reduced or oxidation adduct. The observed "light" mass ([M$_L$+H]$^+$, **X**+456.2064 or **X**+458.2220 Da) of each labeled peak was then used to estimate the mass of potential labeled species (**X** Da) by subtracting the mass of the remaining positively charged probe fragment (456.2064 or 458.2220 Da). The summarized results of the observed (a) oxidized adducts and (b) reduced adducts in media and Ramos cell lysates. The observed and calculated [M+H]$^+$ of the **Tz-ONH$_2$**-labeled species are shown. The potential labeled species and their exact masses are shown.

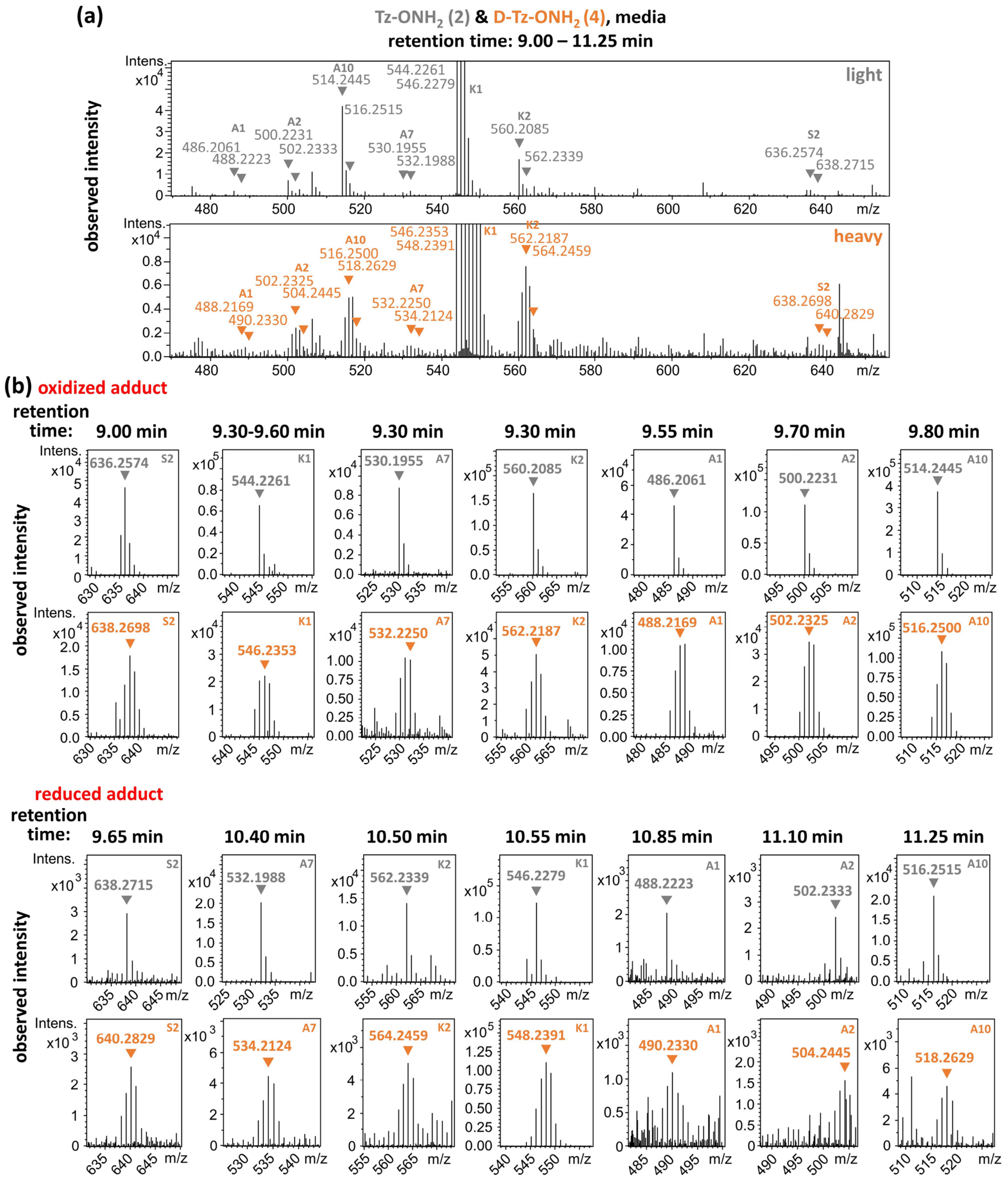

Figure S8. MS spectra of adducts labeled with **Tz-ONH₂ (2)** and **D-Tz-ONH₂ (4)** in media. (a) MS spectra of **H/D-Tz-ONH₂ (2/4)**-labeled oxidized and reduced adducts. The MS spectra were integrated from 9.00 to 11.25 min, and the m/z ranged from 470 to 650 Da. (b) MS spectra and HPLC retention times of **H/D-Tz-ONH₂**-labeled adducts. The labeled species are abbreviated as **S7**.

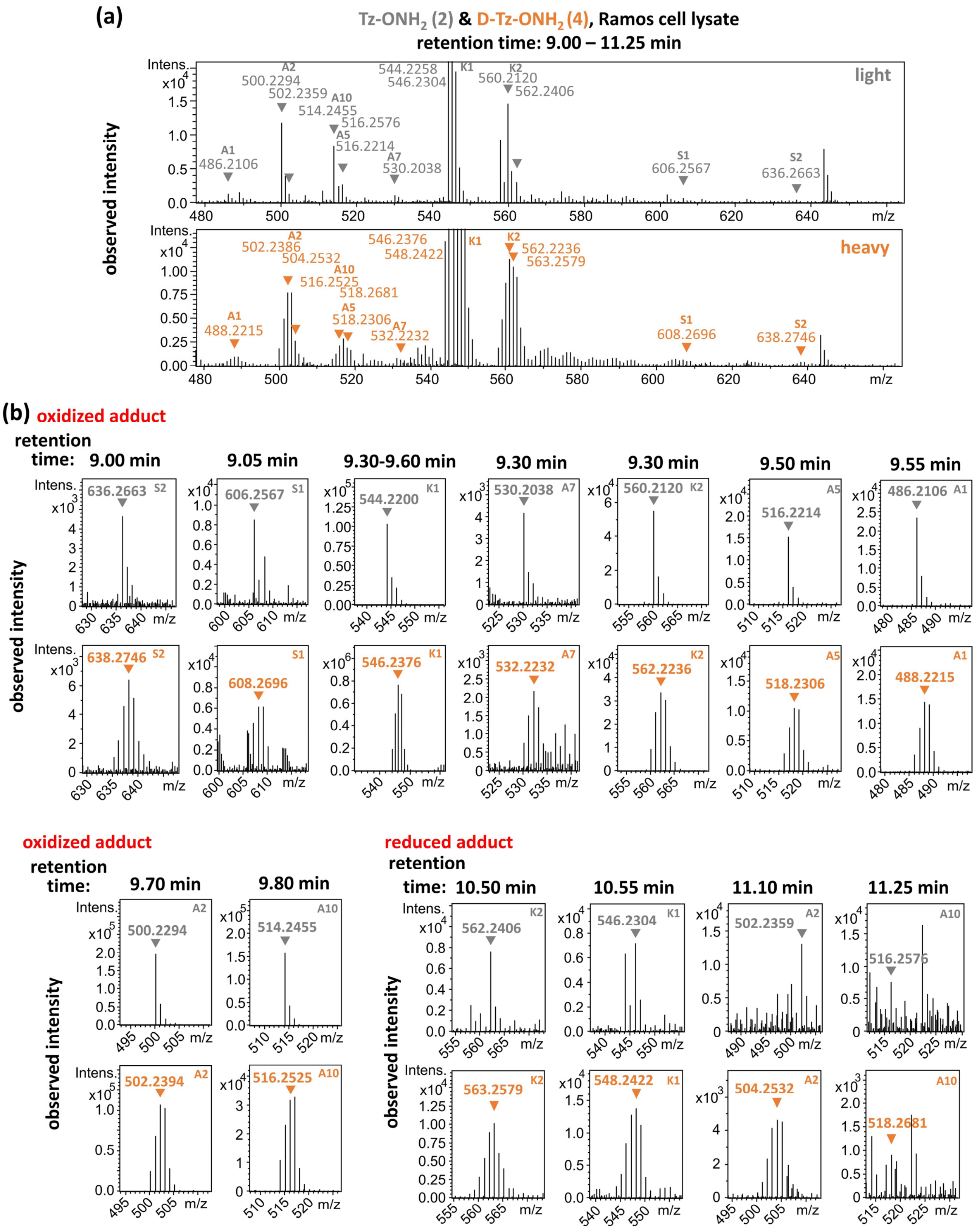

Figure S9. MS spectra of adducts labeled with **Tz-ONH₂ (2)** and **D-Tz-ONH₂ (4)** in Ramos cell lysates. (a) MS spectra of **H/D-Tz-ONH₂ (2/4)**-labeled oxidized and reduced adducts. The MS spectra were integrated from 9.00 to 11.25 min, and the m/z ranged from 480 to 660 Da. (b) MS spectra and HPLC retention times of **H/D-Tz-ONH₂**-labeled adducts. The labeled species are abbreviated as **S7**.

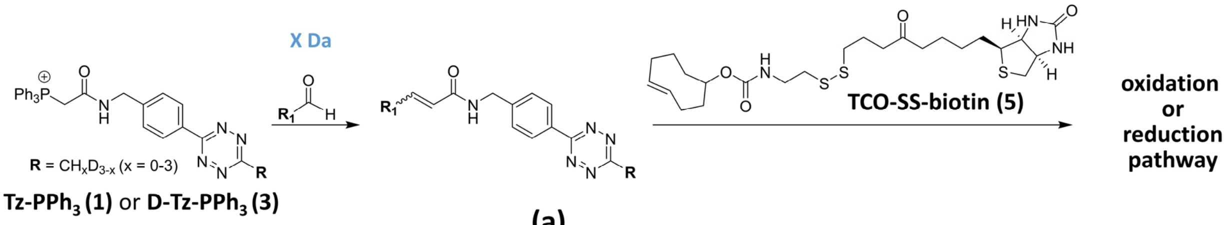

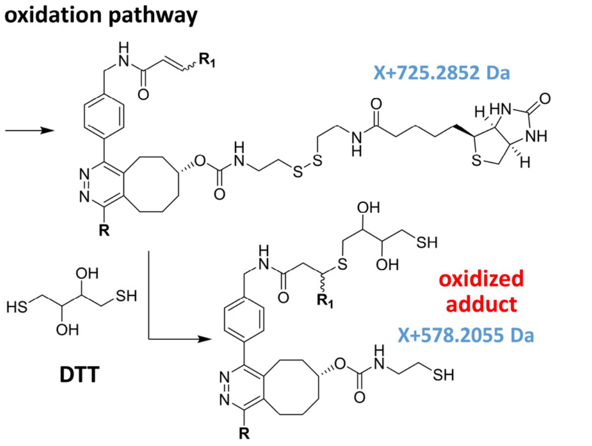

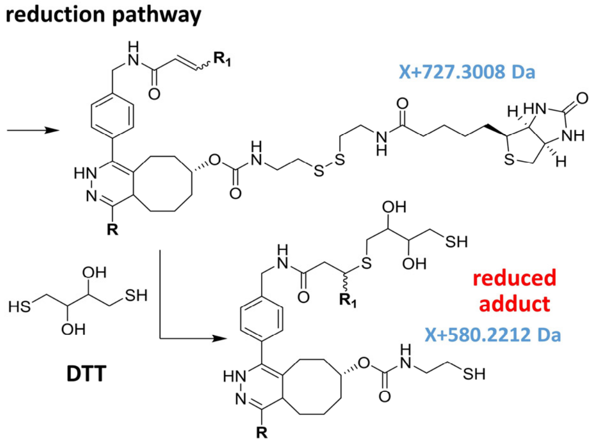

(a)

m/z for oxidized adducts				potential labeled species (exact mass = X)
observed ([M+H]+: [X+579.2128]+)			calculated [M+H]+	
media	Ramos cell lysate	Ramos cell		
609.2202	609.2101	ND	609.2234	formaldehyde (**A1**, FA), exact mass[a] = 30.0106
619.2032	ND	ND	NA	**unknown 1**, calculated mass[b] = 39.9904
623.2374	623.2317	623.2302	623.2390	acetaldehyde (**A2**), exact mass = 44.0262
ND	633.2110	ND	633.2234	propynal (**A3**), exact mass = 54.0106
ND	635.2258	ND	635.2390	acrolein (**A4**), exact mass = 56.0262
ND	639.2217	639.2330	639.2339	glycolaldehyde (**A5**), exact mass = 60.0211
ND	ND	643.2422	NA	**unknown 2**, calculated mass = 64.0294
ND	ND	643.2735	NA	**unknown 3**, calculated mass = 64.0607
ND	651.2228	651.2218	651.2339	methylglyoxal (**A6**, MGO), exact mass = 72.0211
653.2106	ND	653.2039	653.2132	glyoxylic acid (**A7**, GA), exact mass = 74.0004
667.2270	667.2155	ND	667.2289	malonic semialdehyde (**A8**), exact mass = 88.0160
690.2853	ND	690.2804	NA	**unknown 4**, calculated mass = 111.0701
691.2727	ND	691.2653	691.2652	4-oxo-2-hexenal (**A9**), exact mass = 112.0524

(b)

m/z for reduced adducts				potential labeled species (exact mass = X)
observed ([M+H]+: [X+581.2285]+)			calculated [M+H]+	
media	Ramos cell lysate	Ramos cell		
611.2343	ND	ND	611.2390	formaldehyde (**A1**, FA), exact mass = 30.0106
ND	ND	ND	NA	**unknown 1**, calculated mass = 39.9904
625.2519	625.2448	ND	625.2547	acetaldehyde (**A2**), exact mass = 44.0262
ND	635.2262	ND	635.2390	propynal (**A3**), exact mass = 54.0106
ND	637.2365	ND	637.2547	acrolein (**A4**), exact mass = 56.0262
ND	ND	ND	641.2496	glycolaldehyde (**A5**), exact mass = 60.0211
ND	ND	ND	NA	**unknown 2**, calculated mass = 64.0294
ND	ND	ND	NA	**unknown 3**, calculated mass = 64.0607
ND	653.2369	ND	653.2496	methylglyoxal (**A6**, MGO), exact mass = 72.0211
655.2240	655.2188	ND	655.2289	glyoxylic acid (**A7**, GA), exact mass = 74.0004
669.2360	669.2312	ND	669.2445	malonic semialdehyde (**A8**), exact mass = 88.0160
ND	ND	ND	NA	**unknown 4**, calculated mass = 111.0701
ND	ND	ND	693.2809	4-oxo-2-hexenal (**A9**), exact mass = 112.0524

[a] theoretical exact mass of aldehydes
[b] calculated exact mass from our results

Figure S10. Reaction scheme and the summarized labeling results using **H/D-Tz-PPh$_3$ (1/3).** Based on the appearance of both light and heavy isotope patterns at the same retention time in the light/heavy labeled chromatographs, the labeled peaks were first identified as either the reduced or oxidation adduct. The observed "light" mass ($[M_L+H]^+$, **X**+579.2128 or **X**+581.2285 Da) of each labeled peak was then used to estimate the mass of potential labeled species (**X** Da) by subtracting the mass of the remaining positively charged probe fragment (579.2128 or 581.2285 Da). The summarized results of the observed (a) oxidized adducts and (b) reduced adducts in media and Ramos cell lysates and Ramos cells. The observed and calculated $[M+H]^+$ of the **Tz-PPh$_3$**-labeled species are shown. The potential labeled species and their exact masses are shown.

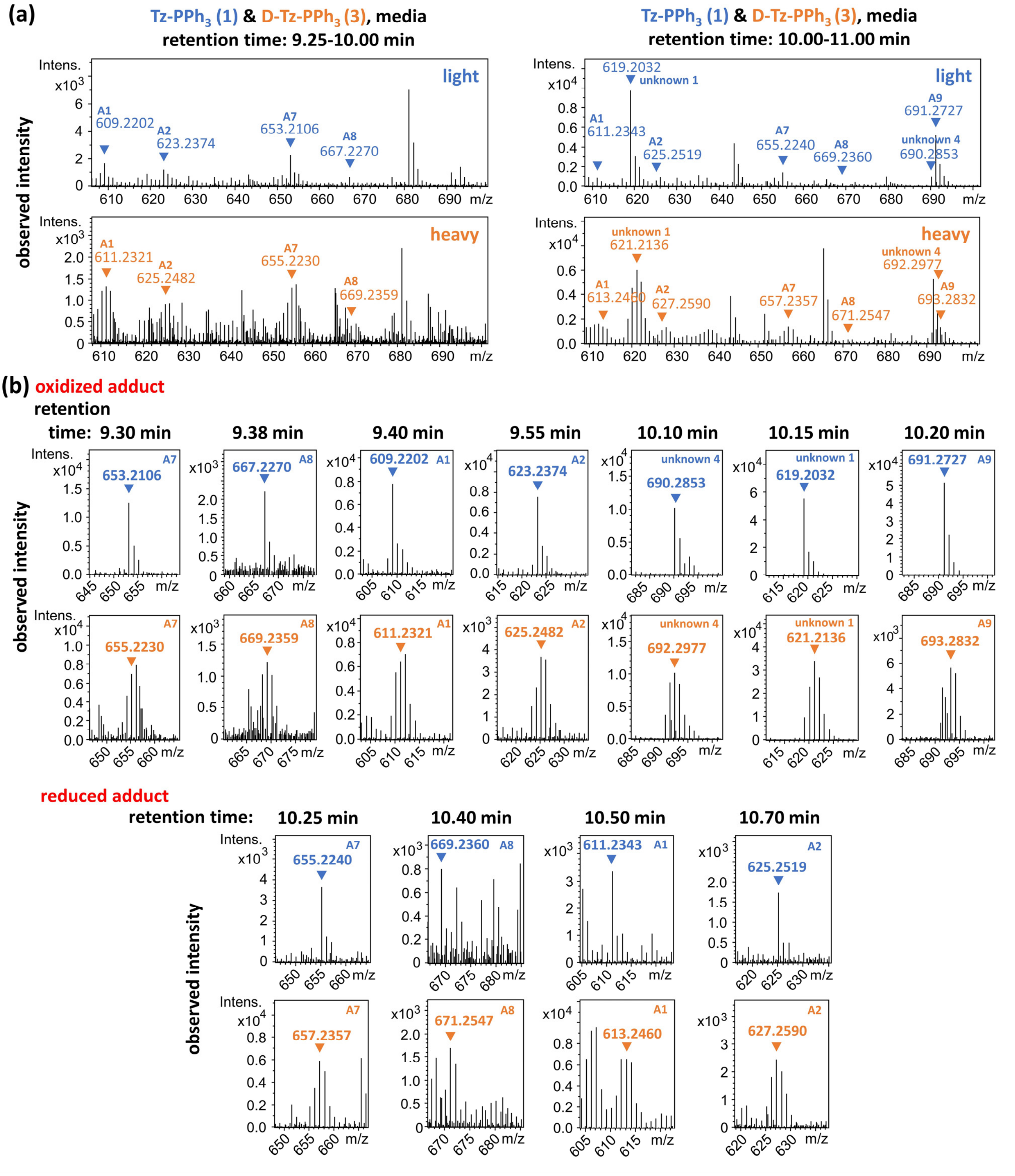

Figure S11. MS spectra of adducts labeled with **Tz-PPh₃ (1)** and **D-Tz-PPh₃ (3)** in media. (a) MS spectra of **H/D-Tz-PPh₃ (1/3)**-labeled oxidized and reduced adducts. The MS spectra were integrated from 9.25 to 10.00 min (for most oxidized adducts) and 10.00 to 11.00 min (for most reduced adducts), and the m/z ranged from 608 to 700 Da. (b) MS spectra and HPLC retention times of **H/D-PPh₃**-labeled adducts. The labeled species are abbreviated as **S10**.

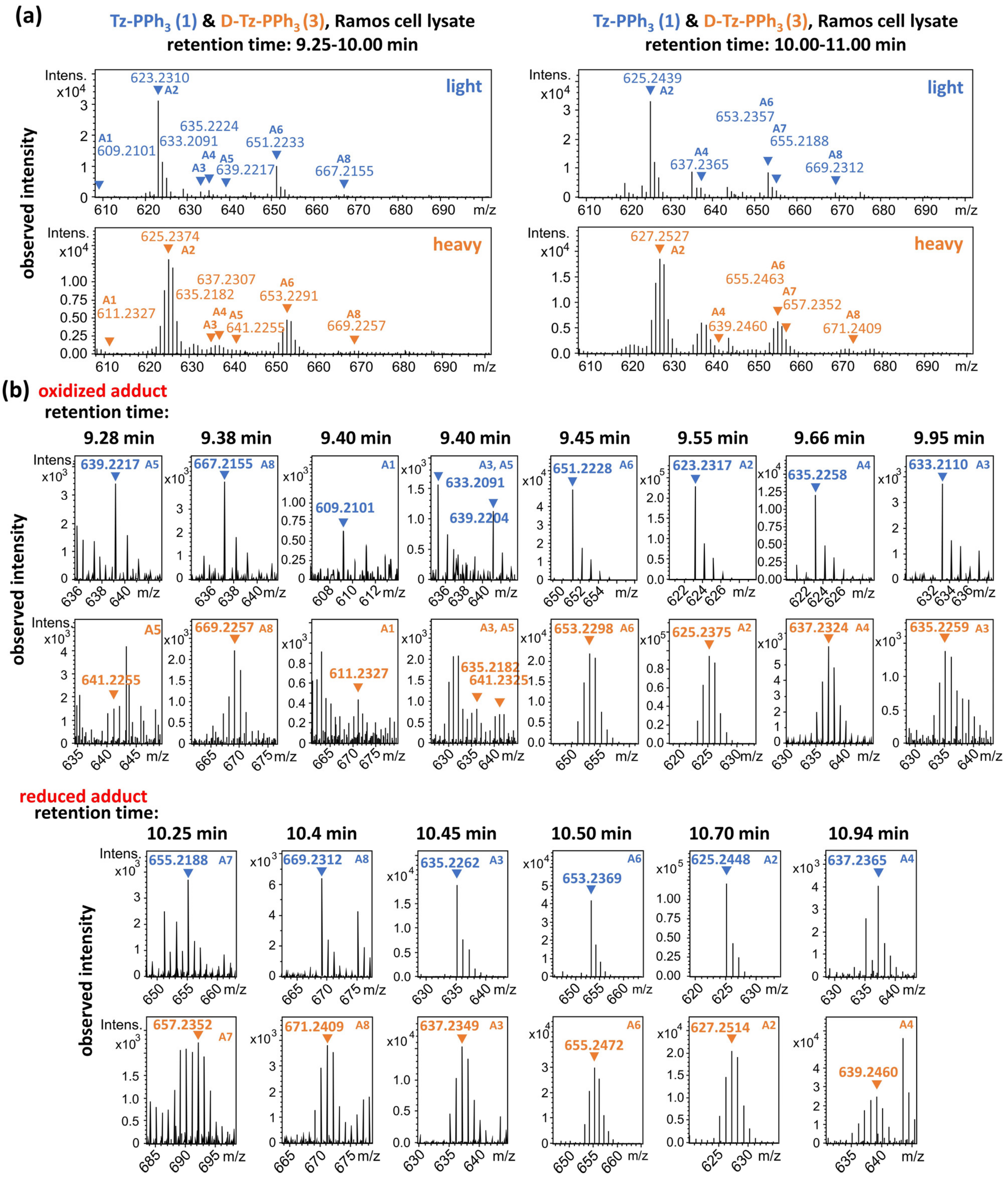

Figure S12. MS spectra of adducts labeled with **Tz-PPh₃ (1)** and **D-Tz-PPh₃ (3)** in Ramos cell lysates. (a) MS spectra of **H/D-Tz-PPh₃ (1/3)**-labeled oxidized and reduced adducts. The MS spectra were integrated from 9.25 to 10.00 min (for most oxidized adducts) and 10.00 to 11.00 min (for most reduced adducts), and the m/z ranged from 608 to 700 Da. (b) MS spectra and HPLC retention times of **H/D-PPh₃**-labeled adducts. The labeled species are abbreviated as **S10**.

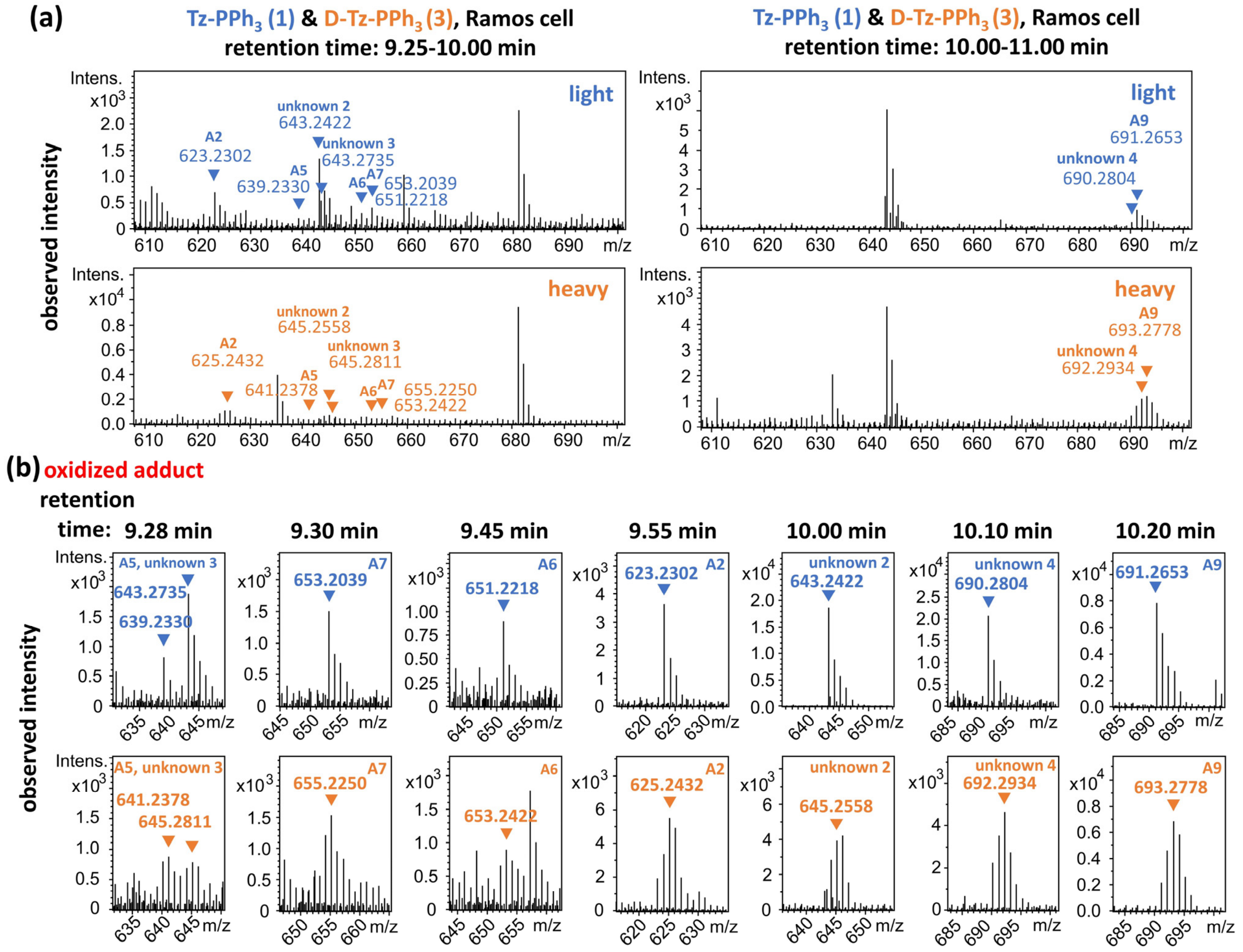

Figure S13. MS spectra of adducts labeled with **Tz-PPh₃ (1)** and **D-Tz-PPh₃ (3)** in Ramos cells. (a) MS spectra of **H/D-Tz-PPh₃ (1/3)**-labeled oxidized adducts. The MS spectra were integrated from 9.25 to 10.00 min and 10.00 to 11.00 min, and the m/z ranged from 608 to 700 Da. (b) MS spectra and HPLC retention times of **H/D-PPh₃**-labeled adducts. The labeled species are abbreviated as **S10**.

(a)

	m/z for oxidized adducts			potential labeled species (exact mass = X)
	observed ([M+H]+: [X+579.2128]+)		calculated [M+H]+	
	MDA-MB-231 cell	HEK293T cell		
aldehydes	643.2476	ND	NA	**unknown 2**, calculated mass = 64.0294
	646.1897	646.1847	NA	**unknown 5**, calculated mass = 66.9719
	672.2324	ND	NA	**unknown 6**, calculated mass = 93.0196
	691.2709	691.2641	691.2652	4-oxo-2-hexenal (**A9**), exact mass = 112.0524

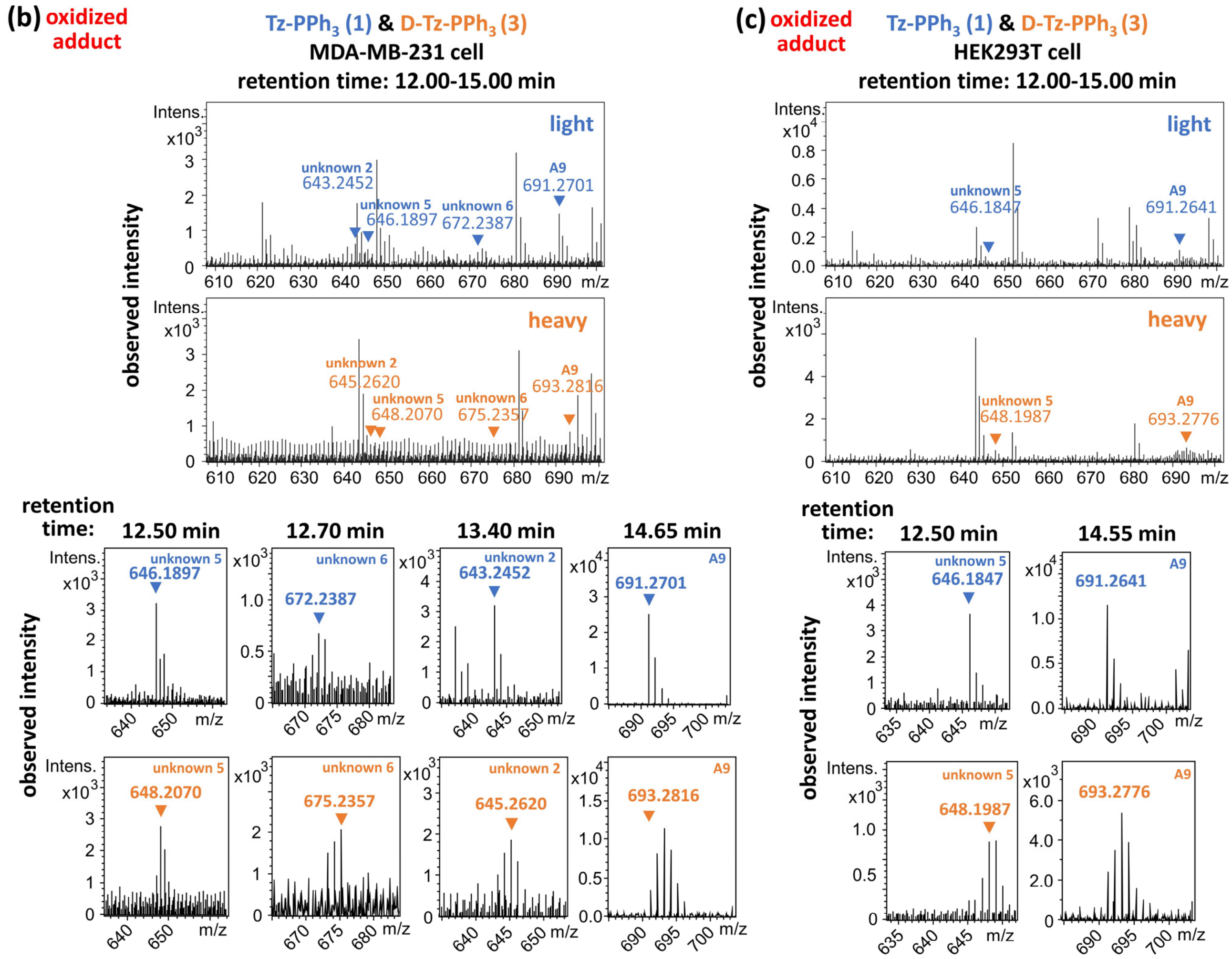

Figure S14. MS spectra of adducts labeled with **Tz-PPh₃ (1)** and **D-Tz-PPh₃ (3)** in MDA-MB-231 and HEK293T cells. (a) The summarized results of the observed oxidized adducts in MDA-MB-231 and HEK293T cells. The observed and calculated $[M+H]^+$ of the **Tz-PPh₃**-labeled species are shown. The potential labeled species and their exact masses are shown. (b) MS spectra and HPLC retention times of **H/D-Tz-PPh₃ (1/3)**-labeled oxidized adducts in MDA-MB-231 cells. (c) MS spectra and HPLC retention times of **H/D-Tz-PPh₃**-labeled oxidized adducts in HEK293T cells. The MS spectra were integrated from 12.00 to 15.00 min, and the m/z ranged from 608 to 700 Da.

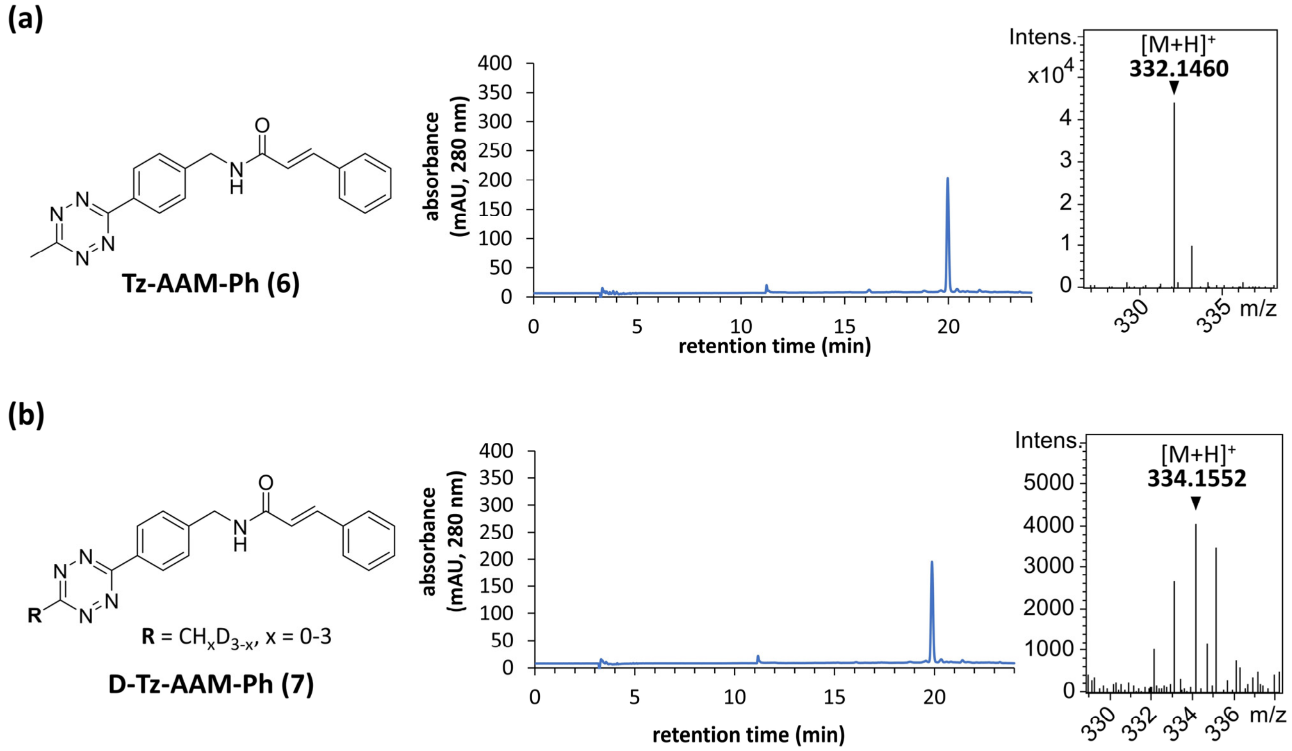

Figure S15. Characterization of (a) **Tz-AAM-Ph (6)** and (b) **D-Tz-AAM-Ph (7)**.

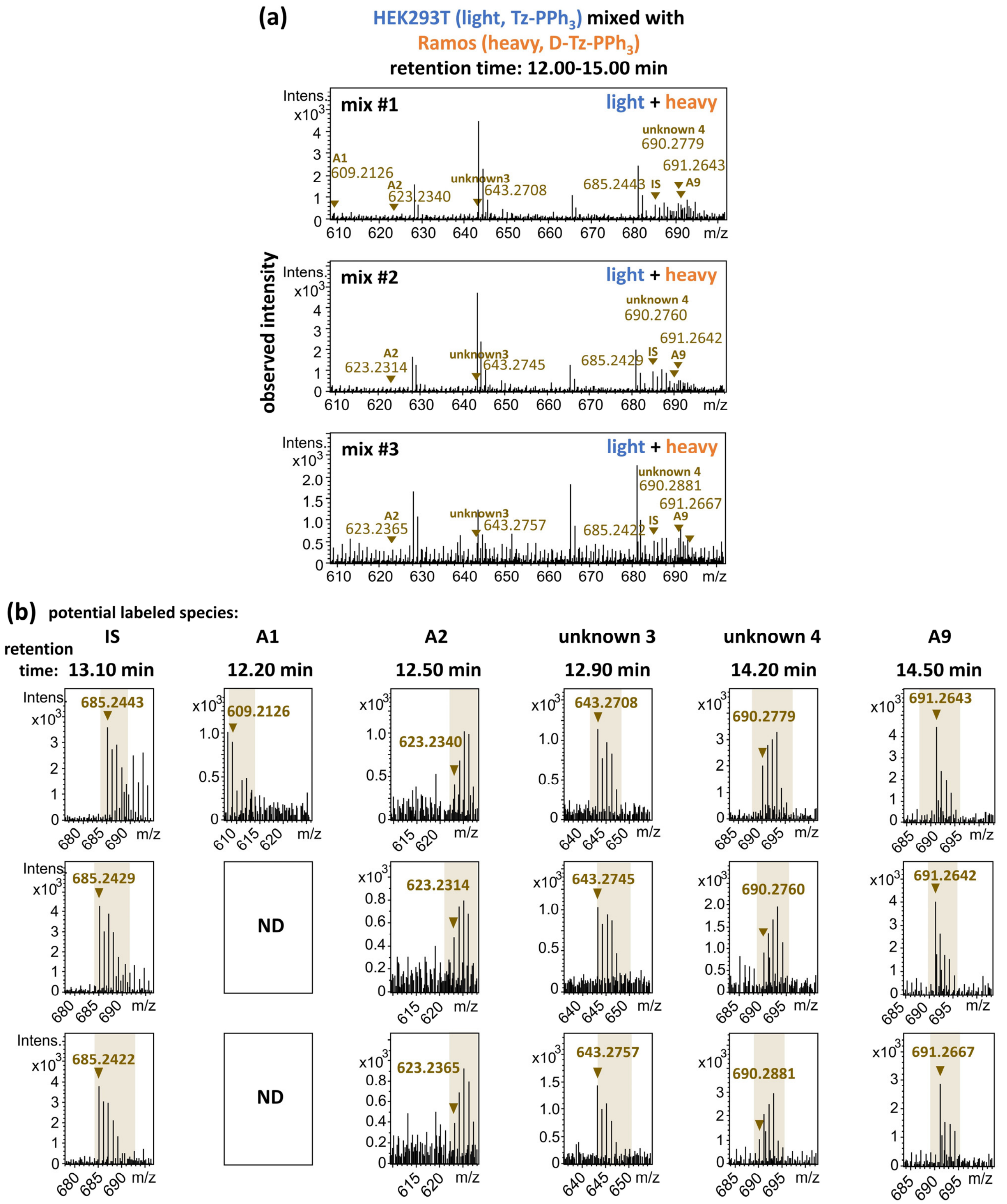

Figure S16. MS spectra of adducts labeled with **Tz-PPh₃ (1)** in HEK293T cells and with **D-Tz-PPh₃ (3)** in Ramos cells. (a) MS spectra of **H/D-Tz-PPh₃ (1/3)**-labeled adducts in HEK293T and Ramos cells. The MS spectra were integrated from 12.00 to 15.00 min, and the m/z ranged from 609 to 700 Da. (b) MS spectra and HPLC retention times of **H/D-PPh₃**-labeled adducts in HEK293T and Ramos cells. IS: internal standard; A1: formaldehyde; A2: acetaldehyde; A9: 4-oxo-2-hexenal.

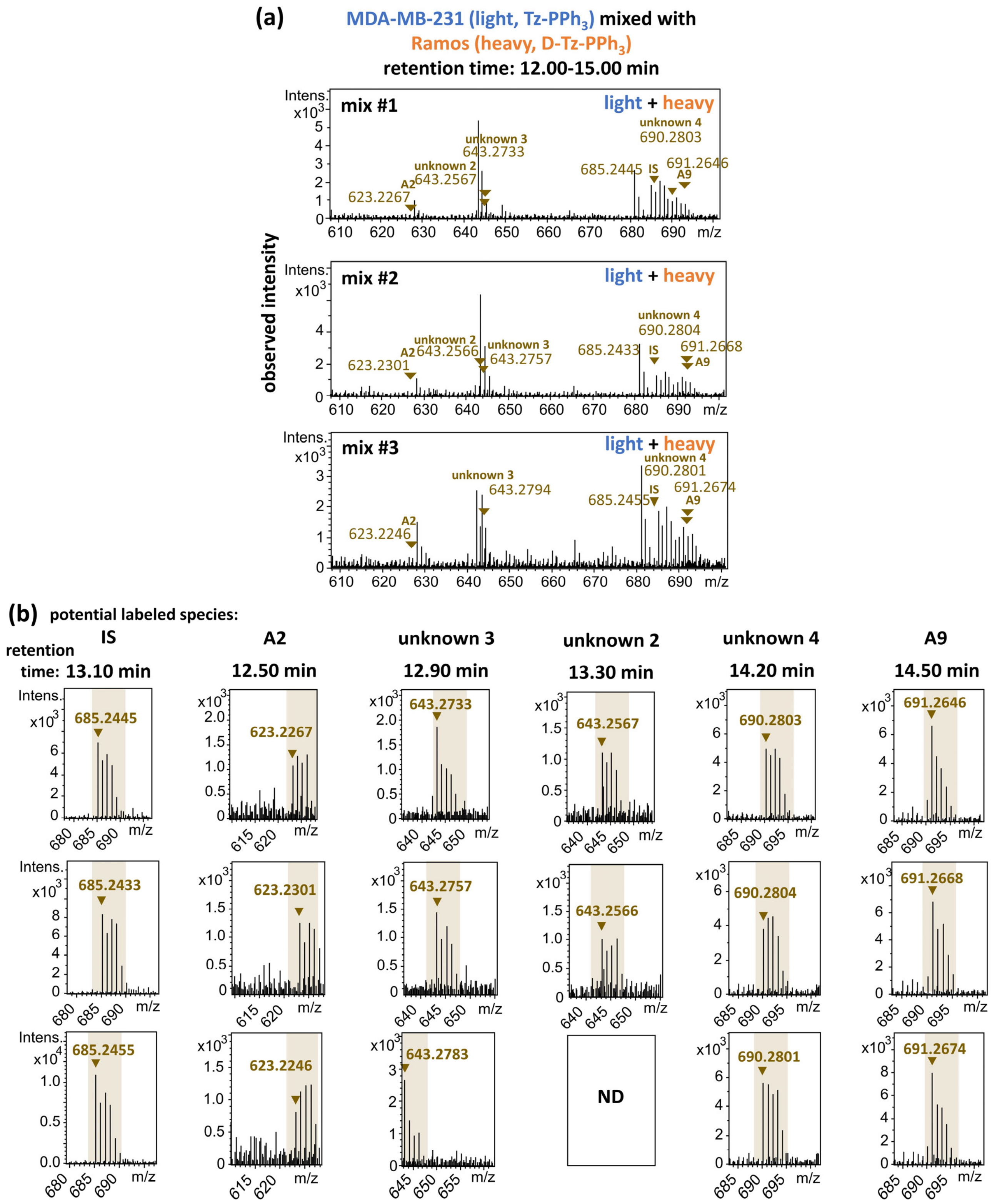

Figure S17. MS spectra of adducts labeled with **Tz-PPh$_3$ (1)** in MDA-MB-231 cells and with **D-Tz-PPh$_3$ (3)** in Ramos cells. (a) MS spectra of **H/D-Tz-PPh$_3$ (1/3)**-labeled adducts in MDA-MB-231 and Ramos cells. The MS spectra were integrated from 12.00 to 15.00 min, and the m/z ranged from 609 to 700 Da. (b) MS spectra and HPLC retention times of **H/D-PPh$_3$**-labeled adducts in MDA-MB-231 and Ramos cells. IS: internal standard; A2: acetaldehyde; A9: 4-oxo-2-hexenal.

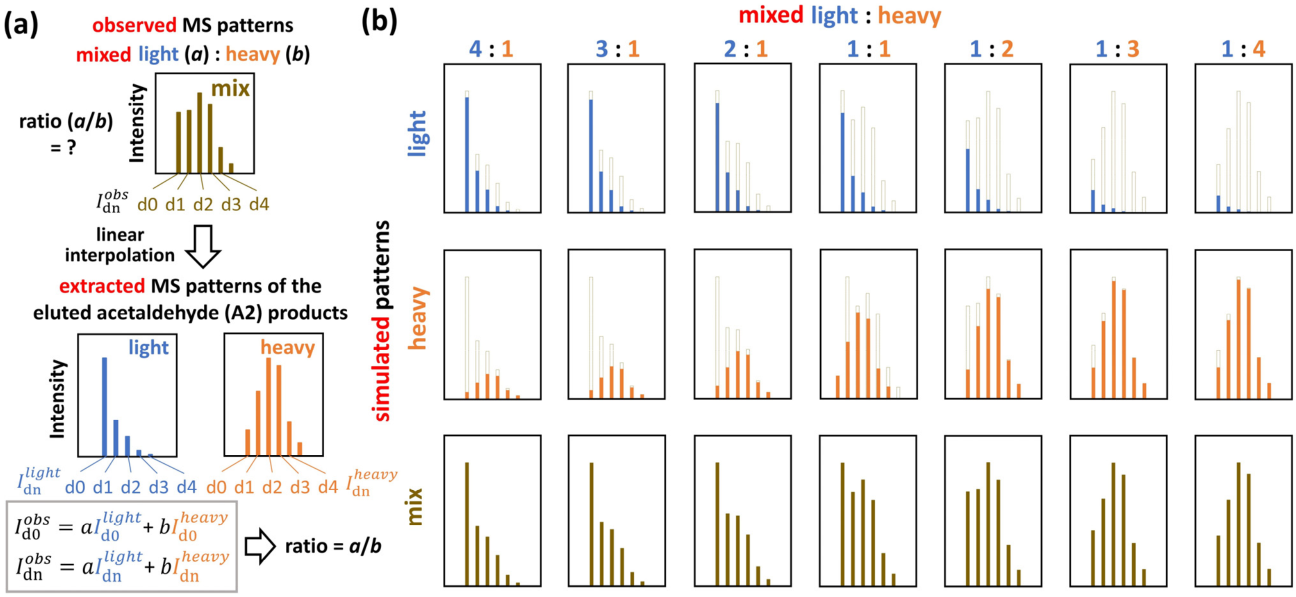

Figure S18. Schematic illustration of linear interpolation for RCS relative quantification. (a) Light/heavy MS patterns of the eluted acetaldehyde (A2) products labeled with **Tz-PPh$_3$ (1)** or **D-Tz-PPh$_3$ (3)**. (b) Light/heavy ratios (4:1 to 1:4) of the simulated MS patterns.

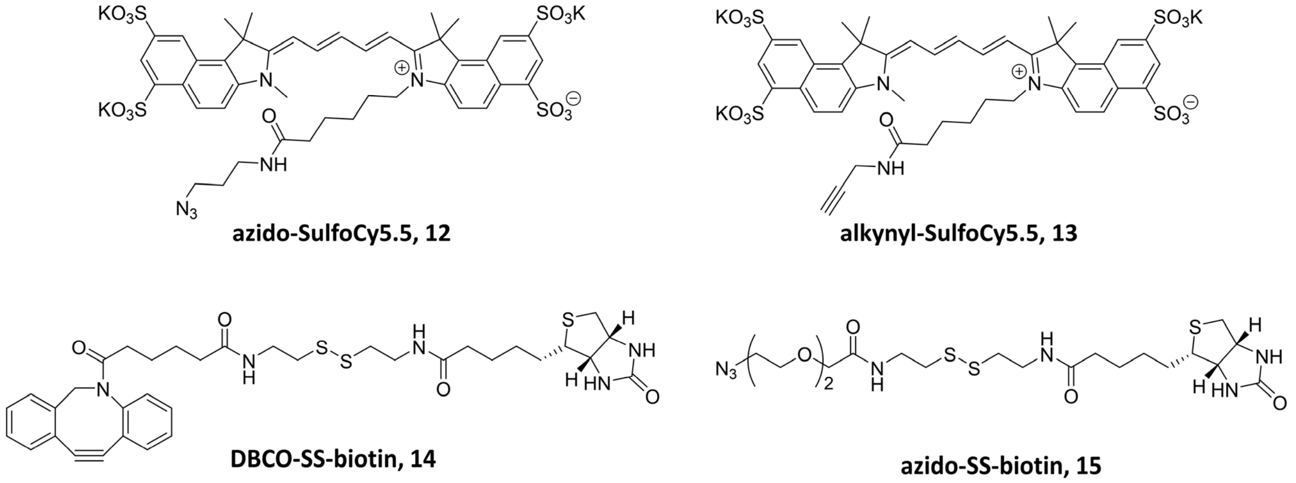

Figure S19. Chemical structures of commercial and homemade functional probes, including **azido-SulfoCy5.5 (12)**, **alkynyl-SulfoCy5.5 (13)**, **DBCO-SS-biotin (14)**, and **azido-SS-biotin (15).**

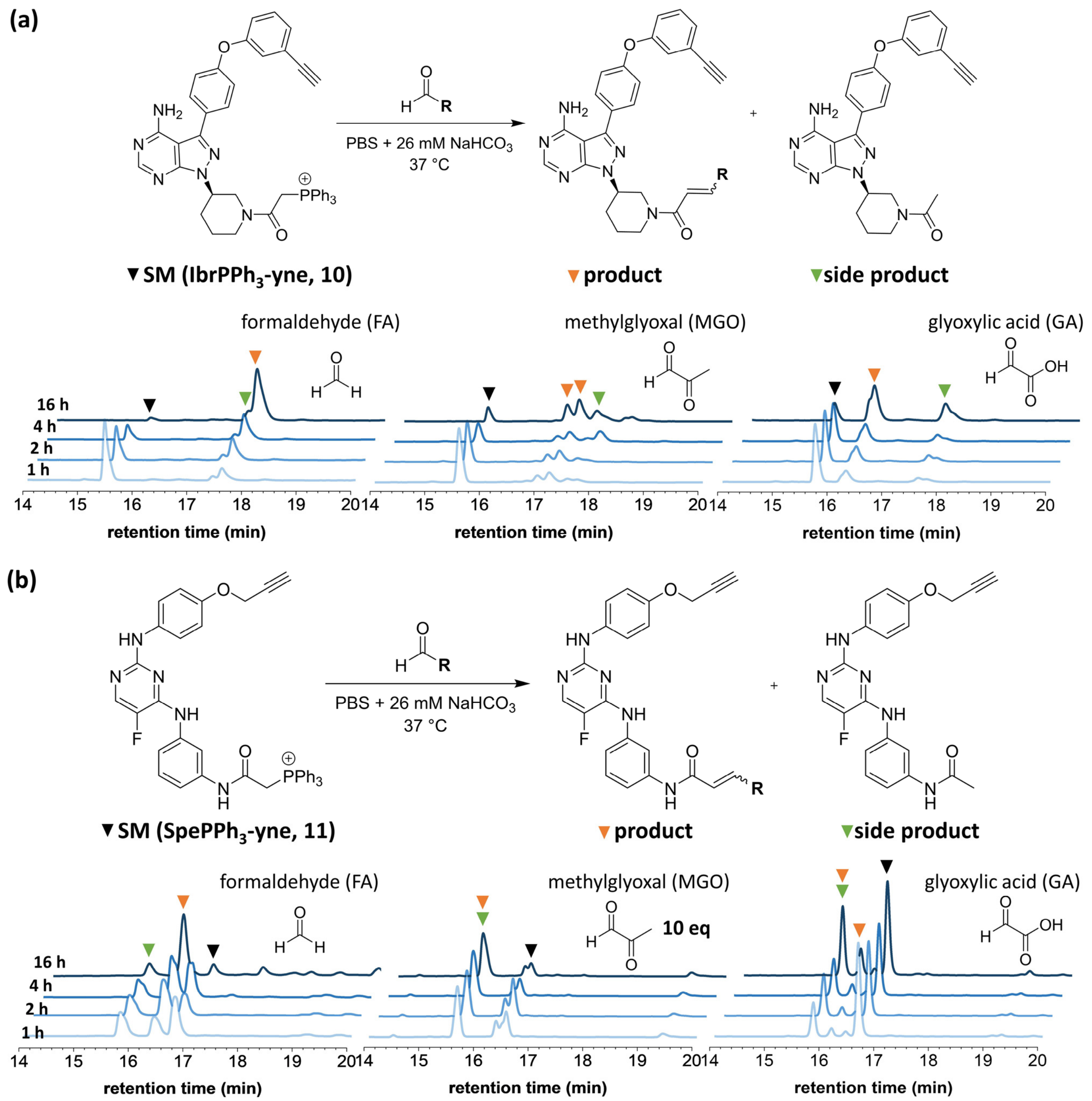

Figure S20. Chromatographs of (a) **IbrPPh$_3$-yne (10)** (100 µM) and (b) **SpePPh$_3$-yne (11)** (100 µM) toward RCS (200 µM) (FA, MGO and GA). The starting material (SM), product and side product are labeled with black, orange, and green triangles, respectively.

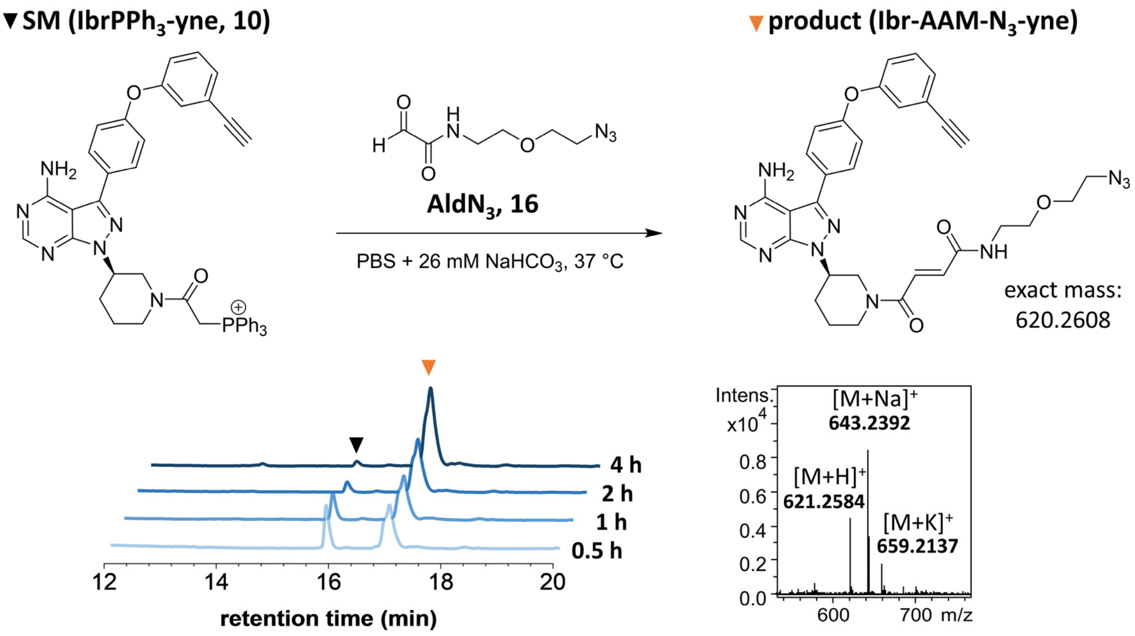

Figure S21. Chromatographs and MS spectrum of the aqueous Wittig reaction between **IbrPPh₃-yne (10)** (100 μM) and **AldN₃ (16)** (300 μM). The starting material (SM) and product are labeled with black and orange triangles, respectively.

(a)

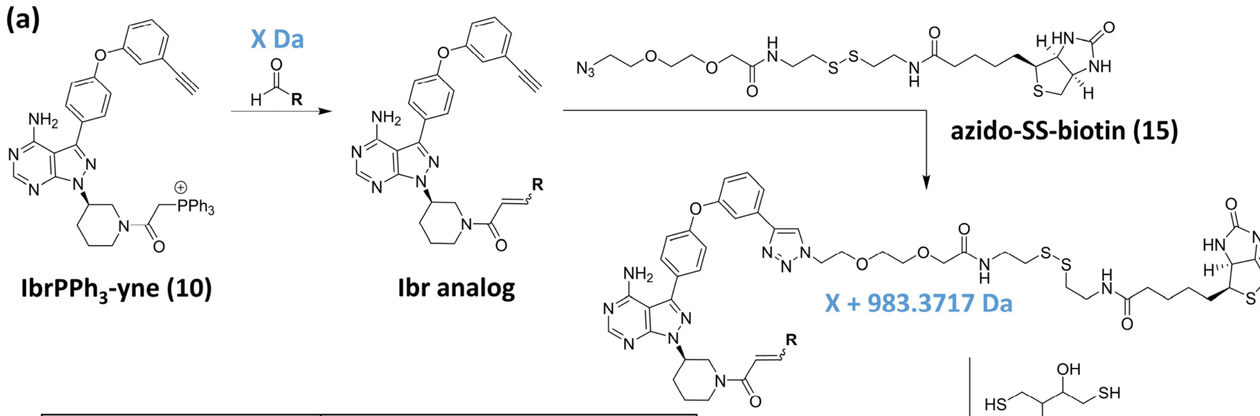

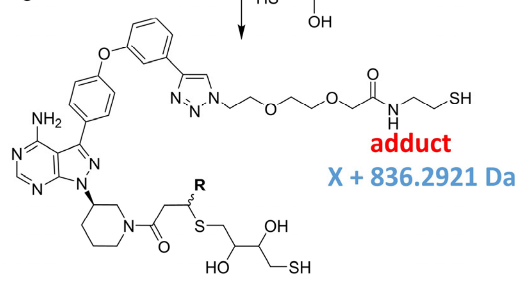

m/z for adducts			potential labeled species (exact mass = X)
observed ([M+H]$^+$: [X+837.2994]$^+$)		calculated [M+H]$^+$	
media	Ramos cell lysate		
867.2831	867.2937	867.3099	formaldehyde (**A1**, FA), exact mass = 30.0106
881.3138	881.3142	881.3256	acetaldehyde (**A2**), exact mass = 44.0262
ND	897.3055	897.3205	glycolaldehyde (**A5**), exact mass = 60.0211
ND	909.3038	909.3205	methylglyoxal (**A6**, MGO), exact mass = 72.0211
911.2895	911.2857	911.2997	glyoxylic acid (**A7**, GA), exact mass = 74.0004

aldehydes

(b)

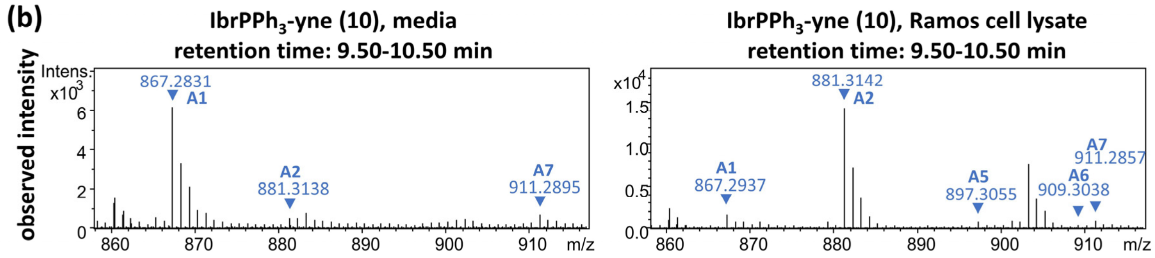

(c)

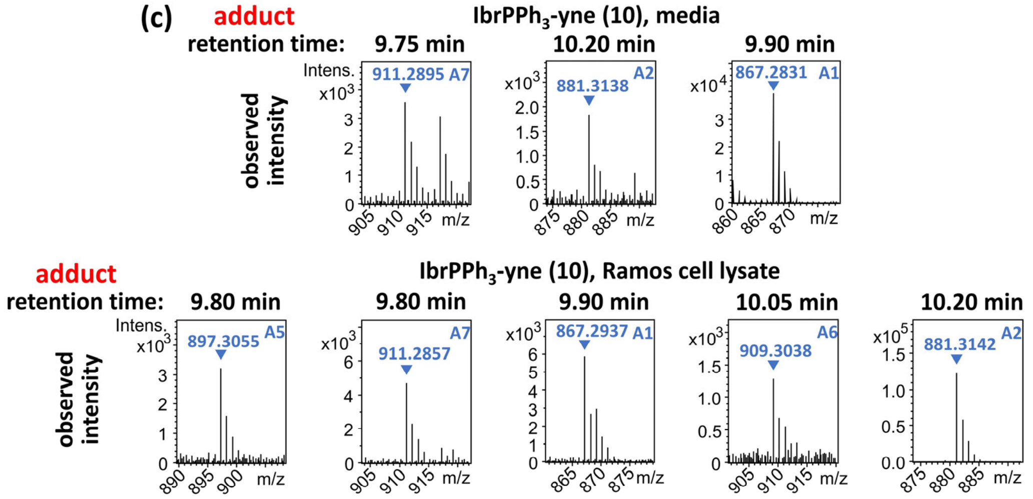

Figure S22. Reaction scheme and labeling results of **IbrPPh$_3$-yne (10)**. (a) Reaction scheme and the summarized results of the observed probe-labeled adducts. The observed and calculated [M+H]$^+$ of labeled species are shown. The observed mass ([M_L+H]$^+$, **X**+837.2994 Da) of each labeled peak was then used to estimate the mass of potential labeled species (**X** Da) by subtracting the mass of the remaining positively charged probe fragment (837.2994 Da). The potential labeled species and their exact masses are shown. (b) MS spectra of probe-labeled adducts in media and Ramos cell lysates. The MS spectra were integrated from 9.50 to 10.50 min, and the m/z ranged from 860 to 916 Da. (c) MS spectra and HPLC retention times of probe-labeled adducts in media and Ramos cell lysates.

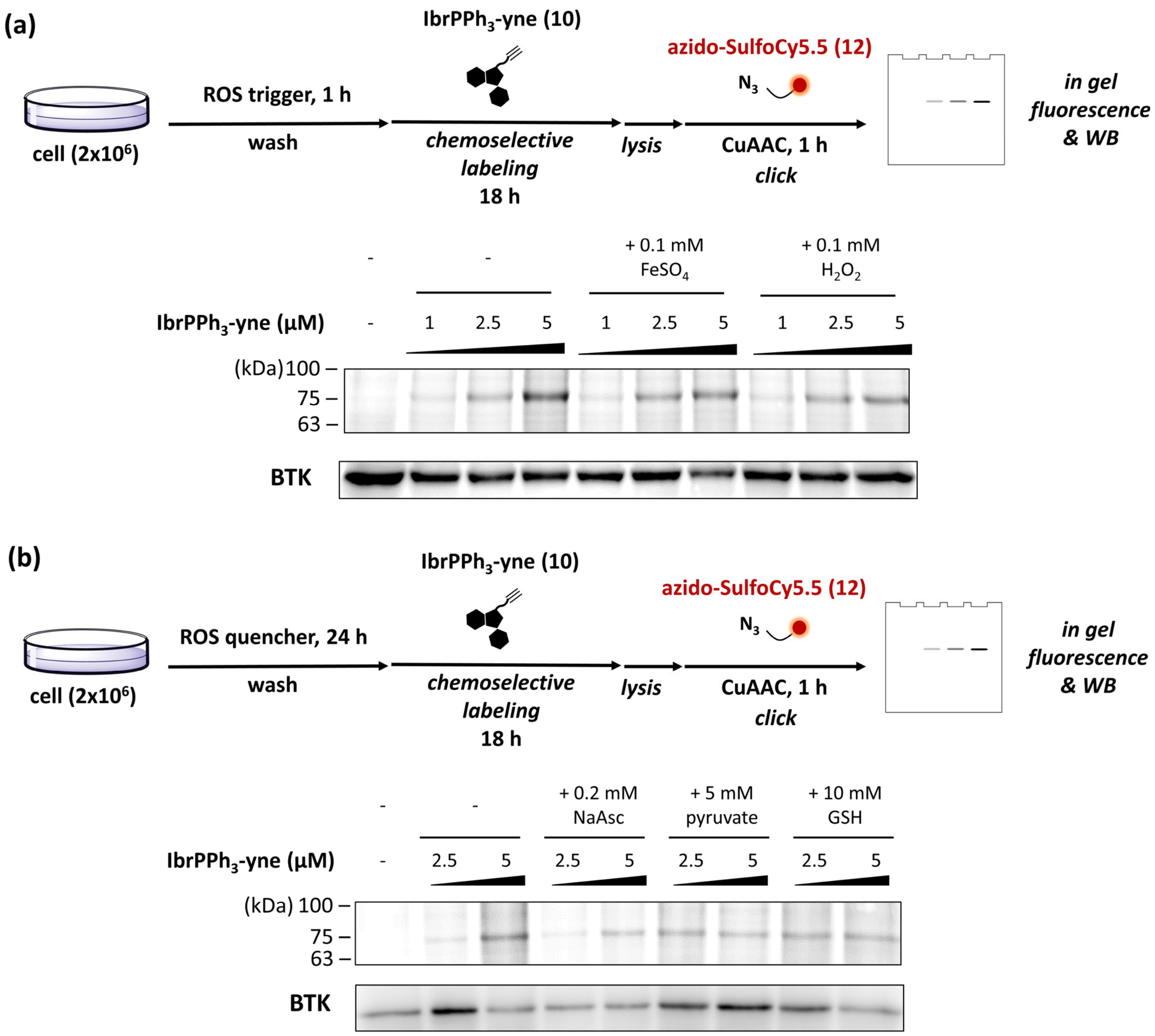

Figure S23. In-gel fluorescence and western blotting results after modulating cellular ROS levels. (a) In-gel fluorescence after treatment with the ROS triggers, $FeSO_4$[16] or H_2O_2. Ramos cells were treated with **IbrPPh3-yne (10)** (1-5 μM) after preincubation with or without ROS triggers, followed by washing, lysis, CuAAC with **azido-SulfoCy5.5 (12)** (10 μM), and detection by in-gel fluorescence. (b) In-gel fluorescence after treatment with the ROS quenchers, NaAsc, pyruvate[17], or GSH[18]. Ramos cells were treated with **IbrPPh3-yne** (2.5 or 5 μM) after preincubation with or without ROS quenchers, followed by washing, lysis, CuAAC with **azido-SulfoCy5.5** (10 μM), and detection by in-gel fluorescence.

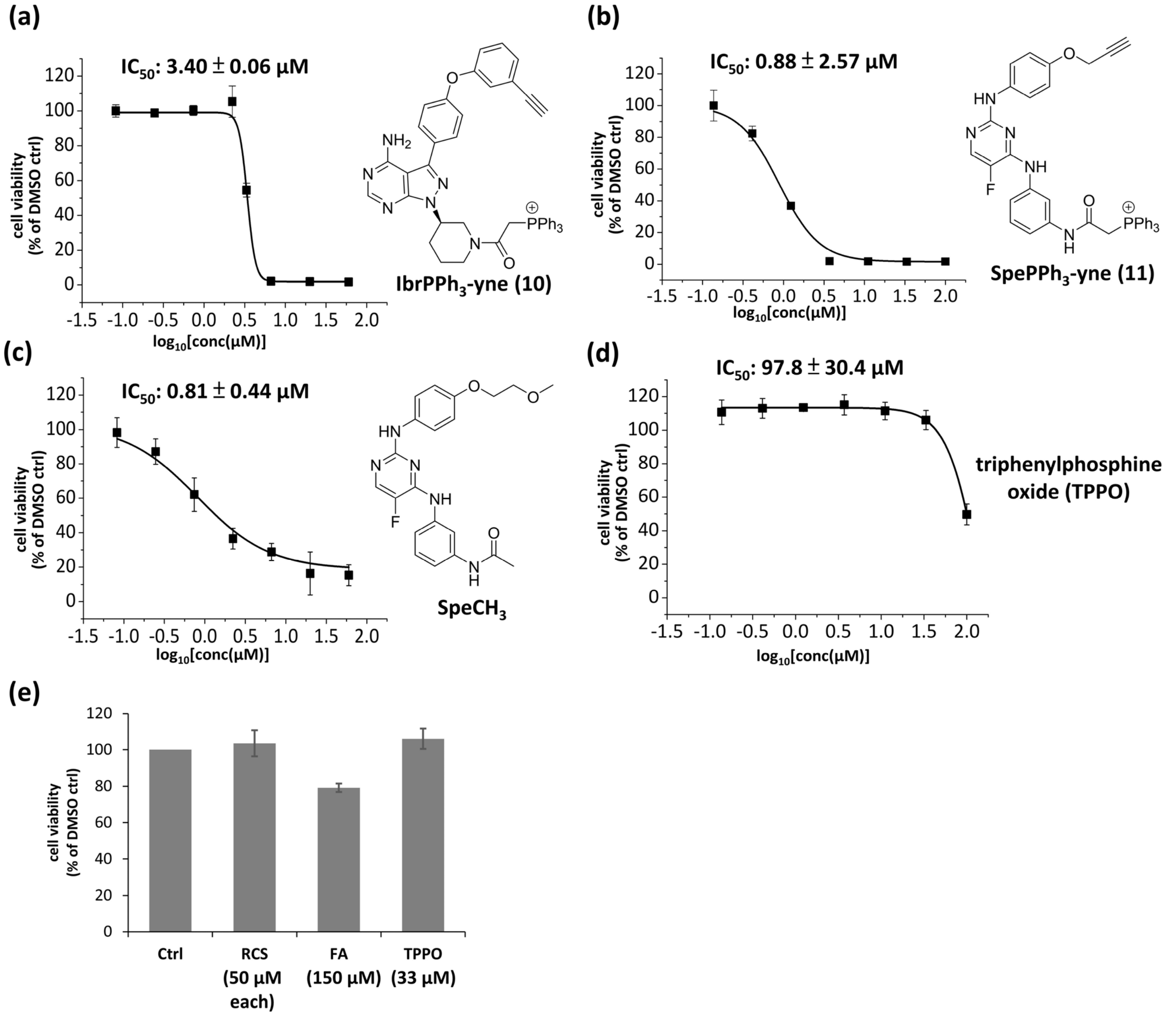

Figure S24. Cytotoxicities and IC_{50} of (a) **IbrPPh$_3$-yne (10)**, (b) **SpePPh$_3$-yne (11),** (c) **SpeCH$_3$**, (d) triphenylphosphine oxide (TPPO), (e) RCS (FA, MGO and GA), formaldehyde (FA) and triphenylphosphine oxide (TPPO).

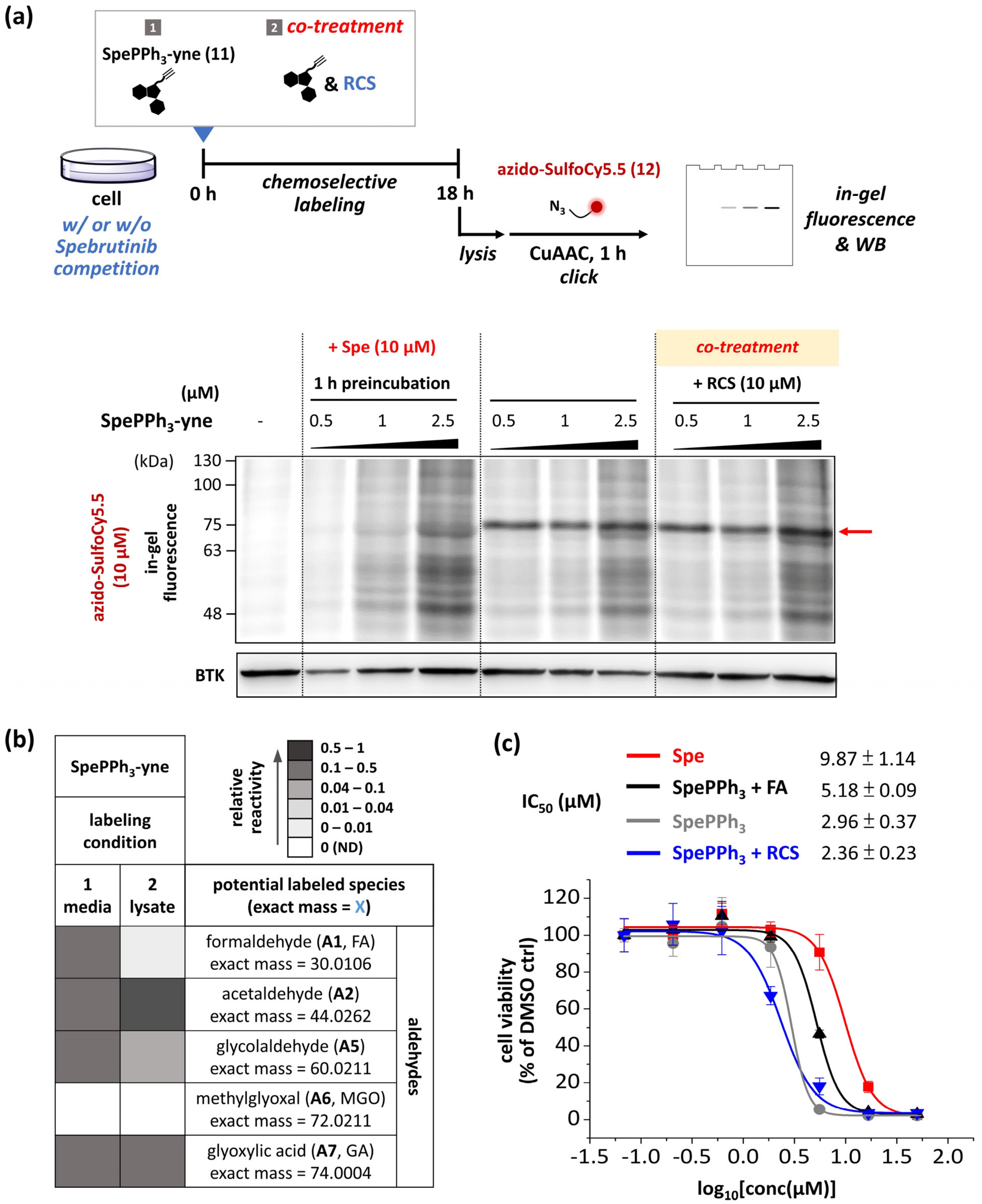

Figure S25. Demonstrating strategy of TCI regeneration in spebrutinib precursors. (a) In-gel fluorescence for validating TCI regeneration in living cells. (b) Metabolome analysis of **SpePPh$_3$-yne (11)** in media and Ramos cell lysates. Media or Ramos cell lysates were treated with **SpePPh$_3$-yne** (100 µM, 37 °C, 16 h) and clicked to **azido-SS-biotin (15)** (100 µM, 37 °C, 3 h) through CuAAC. The mixture was enriched, eluted, desalted, and detected by LC–MS. The color scale is based on the normalized AUC integrated from EICs of labeled species. (c) Cytotoxicities of spebrutinib and its *in situ*-generated analogs. The IC$_{50}$ values are shown.

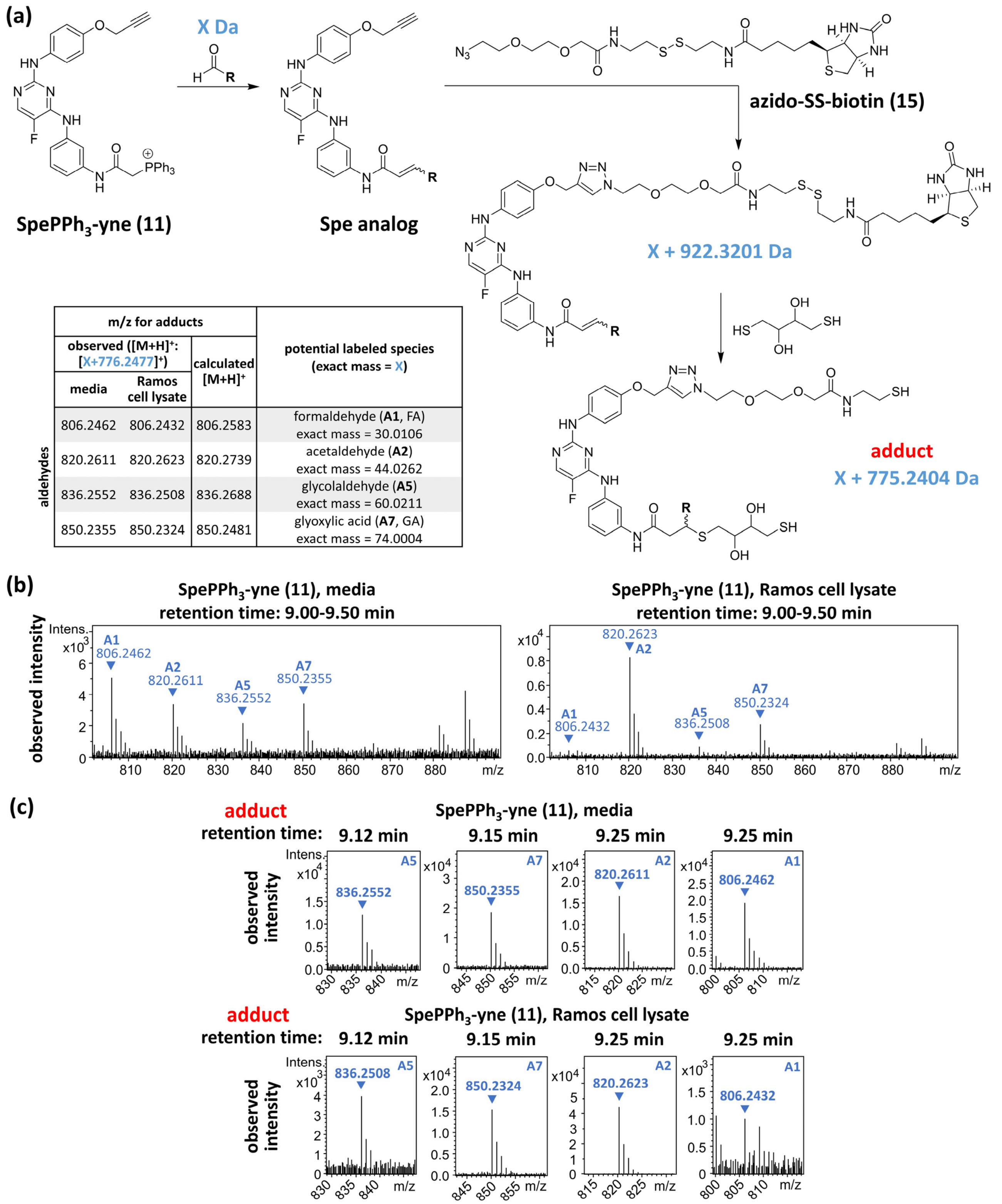

aldehydes	m/z for adducts: observed ([M+H]⁺: [X+776.2477]⁺) media	Ramos cell lysate	calculated [M+H]⁺	potential labeled species (exact mass = X)
	806.2462	806.2432	806.2583	formaldehyde (**A1**, FA) exact mass = 30.0106
	820.2611	820.2623	820.2739	acetaldehyde (**A2**) exact mass = 44.0262
	836.2552	836.2508	836.2688	glycolaldehyde (**A5**) exact mass = 60.0211
	850.2355	850.2324	850.2481	glyoxylic acid (**A7**, GA) exact mass = 74.0004

Figure S26. Reaction scheme and labeling results of **SpePPh3-yne (11)**. (a) Reaction scheme and the summarized results of the observed probe-labeled adducts. The observed and calculated $[M+H]^+$ of labeled species are shown. The observed mass ($[M_L+H]^+$, **X**+776.2477 Da) of each labeled peak was then used to estimate the mass of potential labeled species (**X** Da) by subtracting the mass of the remaining positively charged probe fragment (776.2477 Da). The potential labeled species and their exact masses are shown. (b) MS spectra of probe-labeled adducts in media and Ramos cell lysates. The MS spectra were integrated from 9.00 to 9.50 min, and the m/z ranged from 802 to 894 Da. (c) MS spectra and HPLC retention times of probe-labeled adducts in media and Ramos cell lysates.

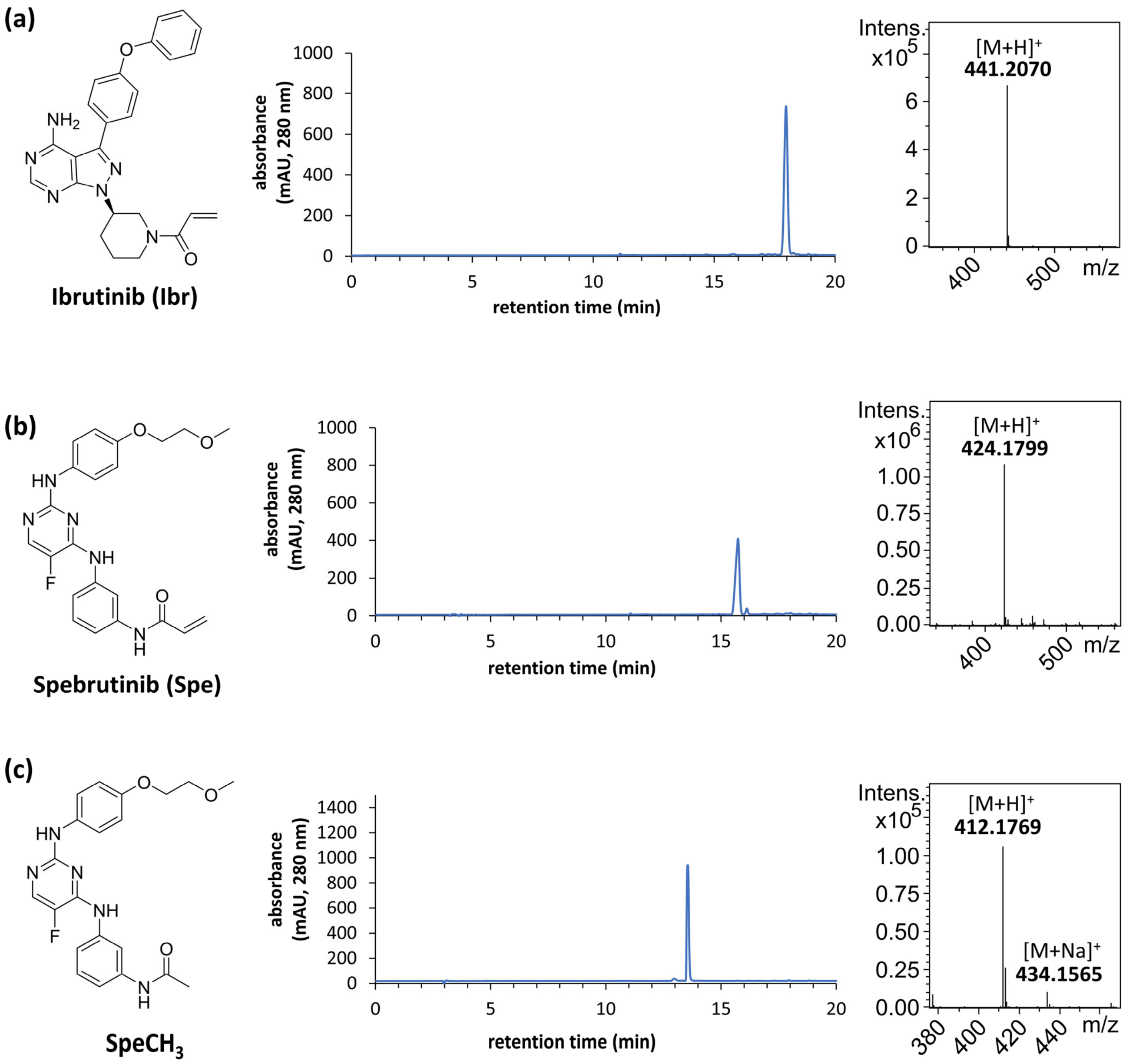

Figure S27. Characterization of (a) ibrutinib (Ibr), (b) spebrutinib (Spe) and (c) **$SpeCH_3$**.

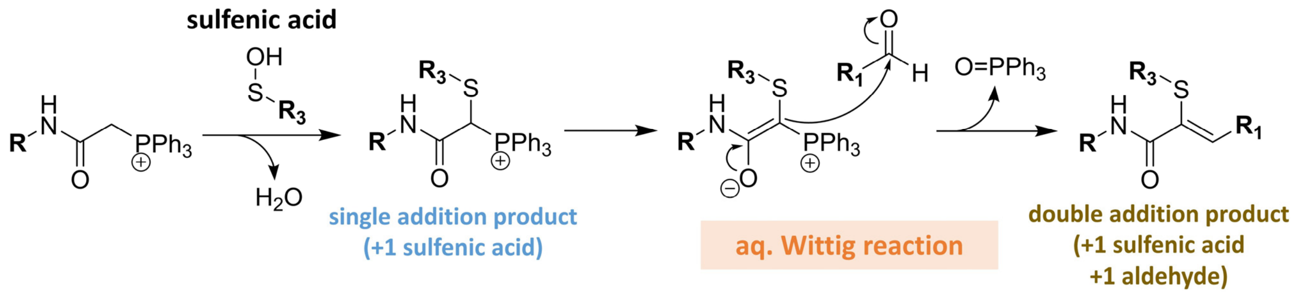

Figure S28. The scheme of aq. Wittig reaction with the sulfenic acid-added product.

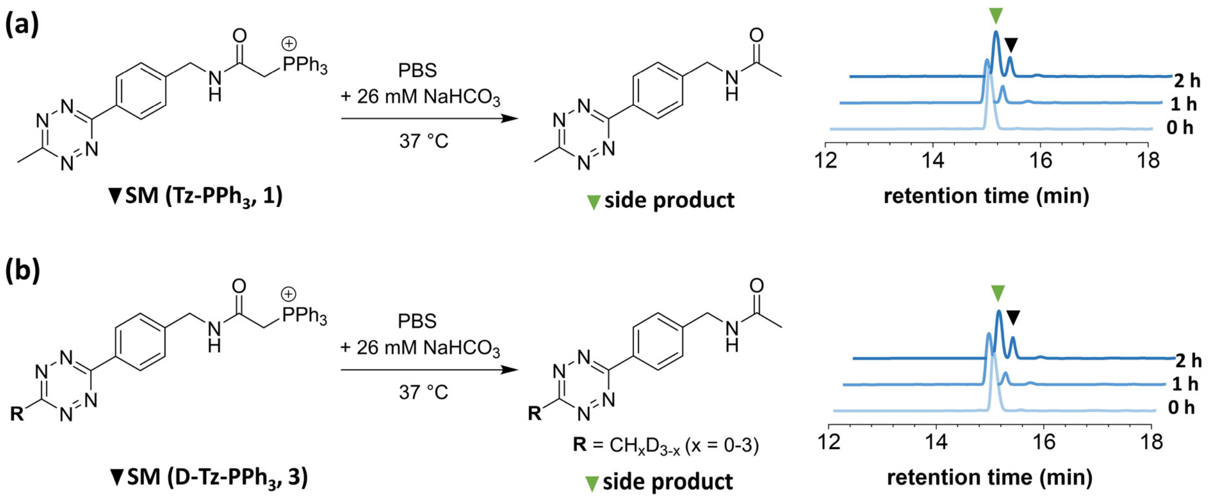

Figure S29. Stability test in PBS (pH 7.4) with 26 mM $NaHCO_3$. (a) **Tz-PPh$_3$ (1)** (100 µM). (b) **D-Tz-PPh$_3$ (3)** (100 µM). The starting material (SM) and side product are labeled with black and green triangles, respectively.

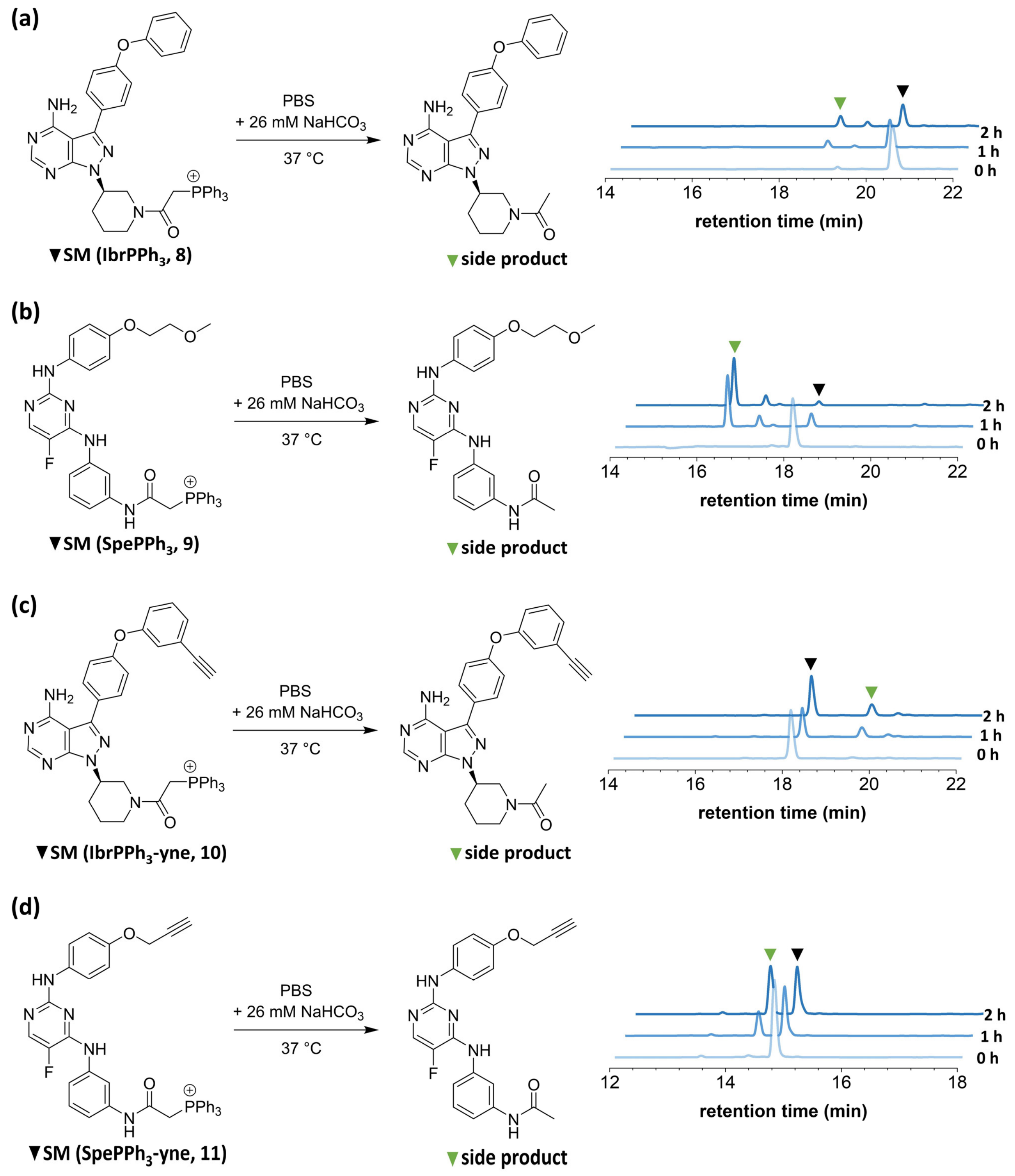

Figure S30. Stability test in PBS (pH 7.4) with 26 mM $NaHCO_3$. (a) **$IbrPPh_3$ (8)** (100 µM). (b) **$SpePPh_3$ (9)** (100 µM). (c) **$IbrPPh_3$-yne (10)** (100 µM). (d) **$SpePPh_3$-yne (11)** (100 µM). The starting material (SM) and side product are labeled with black and green triangles, respectively.

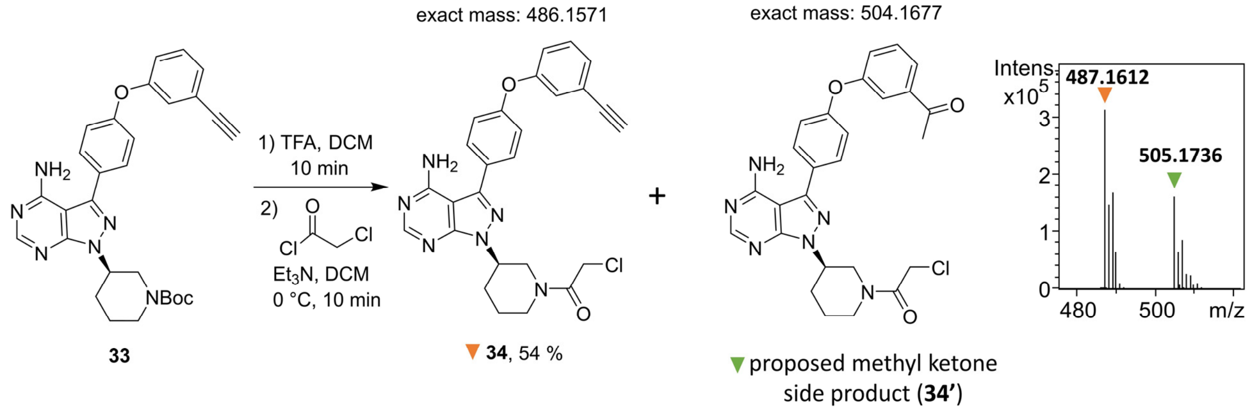

Figure S31. Reaction scheme and MS spectrums of the methyl ketone side product (**34’**).

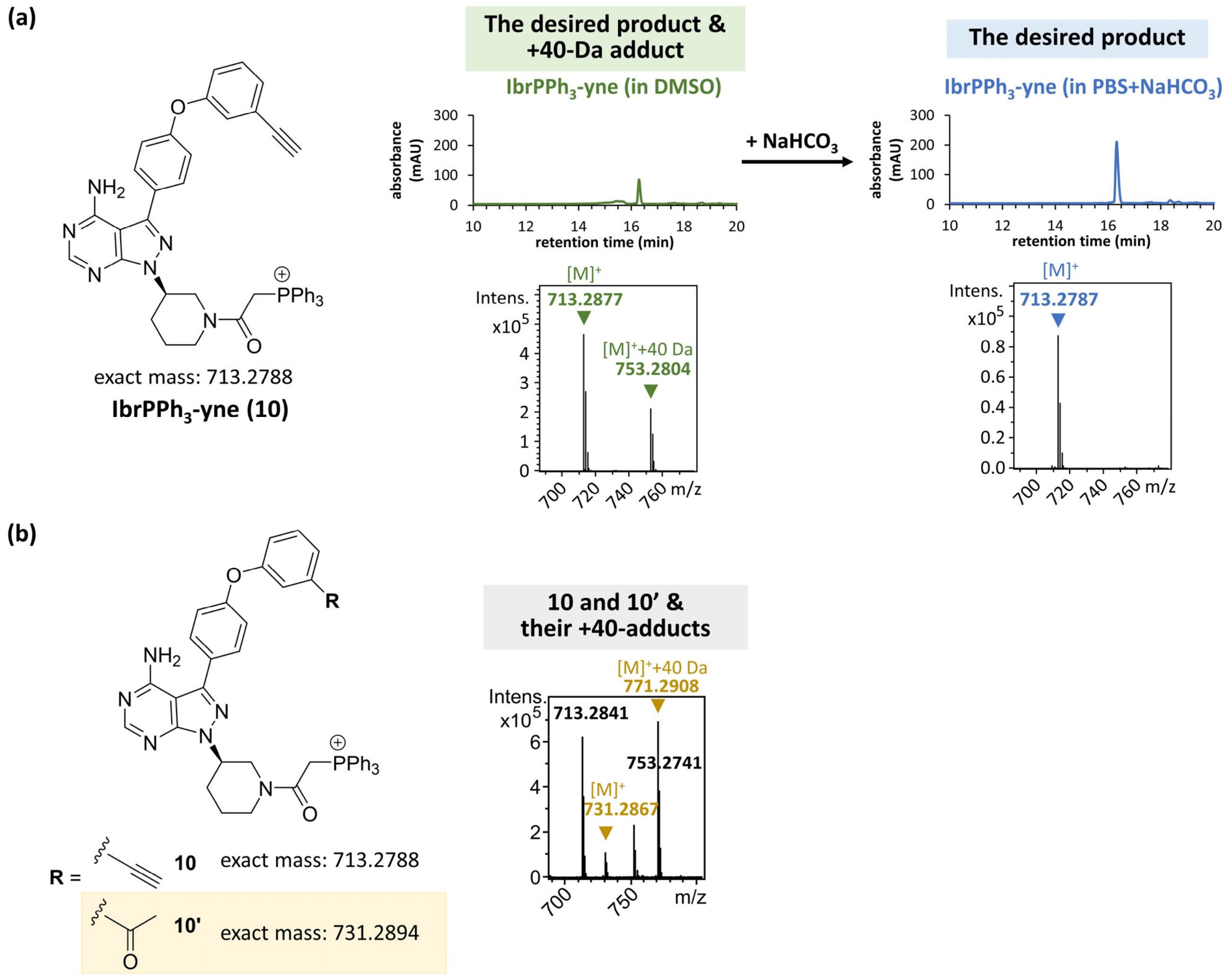

Figure S32. Characterization of **IbrPPh$_3$-yne (10)** and **10’** with their +40-adducts. (a) MS spectra and chromatographs of **IbrPPh$_3$-yne** dissolved in DMSO or PBS (pH 7.4) with 26 mM NaHCO$_3$. (b) MS spectrum of **10** and **10’** with their +40-adducts.

11. References

[1] Willems, L. I.; Li, N.; Florea, B. I.; Ruben, M.; Van Der Marel, G. A.; Overkleeft, H. S. Triple bioorthogonal ligation strategy for simultaneous labeling of multiple enzymatic activities. *Angew. Chem.* **2012**, *124* (18), 4507-4510.

[2] Yang, J.; Karver, M. R.; Li, W.; Sahu, S.; Devaraj, N. K. Metal Catalyzed One-Pot Synthesis of Tetrazines Directly from Aliphatic Nitriles and Hydrazine. *Angew. Chem. Int. Ed.* **2012**, *51* (21), 5222.

[3] Shie, J. J.; Liu, Y. C.; Hsiao, J. C.; Fang, J. M.; Wong, C. H. A cell-permeable and triazole-forming fluorescent probe for glycoconjugate imaging in live cells. *Chem. Commun.* **2017**, *53* (9), 1490-1493.

[4] Xiong, H.; Gu, Y.; Zhang, S.; Lu, F.; Ji, Q.; Liu, L.; Ma, P.; Yang, G.; Hou, W.; Xu, H. Iridium-catalyzed C–H amidation of s-tetrazines. *Chem. Commun.* **2020**, *56* (34), 4692-4695.

[5] Pantoom, S.; Hules, L.; Schöll, C.; Petrosyan, A.; Monticelli, M.; Pospech, J.; Cubellis, M. V.; Hermann, A.; Lukas, J. Mechanistic insight into the mode of action of acid β-glucosidase enhancer ambroxol. *Int. J. Mol. Sci.* **2022**, *23* (7), 3536.

[6] Lamping, M.; Grell, Y.; Geyer, A. Synthesis and conformational analysis of an expanded cyclic ketoxime-hexapeptide. *J. Pept. Sci.* **2016**, *22* (4), 228-235.

[7] Wang, T. S. A.; Wu, R. Y.; Hong, Y.; Wang, Z. C.; Li, T. L.; Shie, J. J.; Hsu, C. C. Labeling and Characterization of Phenol-Containing Glycopeptides Using Chemoselective Probes with Isotope Tags. *ChemBioChem* **2021**, *22* (14), 2415-2419.

[8] Deng, L.; Norberg, O.; Uppalapati, S.; Yan, M.; Ramström, O. Stereoselective synthesis of light-activatable perfluorophenylazide-conjugated carbohydrates for glycoarray fabrication and evaluation of structural effects on protein binding by SPR imaging. *Org. Biomol. Chem.* **2011**, *9* (9), 3188-3198.

[9] Clave, G.; Boutal, H.; Hoang, A.; Perraut, F.; Volland, H.; Renard, P. Y.; Romieu, A. A novel heterotrifunctional peptide-based cross-linking reagent for facile access to bioconjugates. Applications to peptide fluorescent labelling and immobilisation. *Org. Biomol. Chem.* **2008**, *6* (17), 3065-3078.

[10] Faust, A.; Bäumer, N.; Schlütermann, A.; Becht, M.; Greune, L.; Geyer, C.; Rüter, C.; Margeta, R.; Wittmann, L.; Dersch, P. Tumor-Cell-Specific Targeting of Ibrutinib: Introducing Electrostatic Antibody-Inhibitor Conjugates (AiCs). *Angew. Chem. Int. Ed.* **2022**, *61* (1), e202109769.

[11] Li, X.; Wang, A.; Yu, K.; Qi, Z.; Chen, C.; Wang, W.; Hu, C.; Wu, H.; Wu, J.; Zhao, Z. Discovery of (*R*)-1-(3-(4-Amino-3-(4-phenoxyphenyl)-1 *H*-pyrazolo [3, 4-*d*] pyrimidin-1-yl) piperidin-1-yl)-2-(dimethylamino) ethanone (CHMFL-FLT3-122) as a Potent and Orally Available FLT3 Kinase Inhibitor for FLT3-ITD Positive Acute Myeloid Leukemia. *J. Med. Chem.* **2015**, *58* (24), 9625-9638.

[12] Lanning, B. R.; Whitby, L. R.; Dix, M. M.; Douhan, J.; Gilbert, A. M.; Hett, E. C.; Johnson, T. O.; Joslyn, C.; Kath, J. C.; Niessen, S. A road map to evaluate the proteome-wide selectivity of covalent kinase inhibitors. *Nat. Chem. Biol.* **2014**, *10* (9), 760-767.

[13] El Khoury, A.; Seidler, P. M.; Eisenberg, D. S.; Harran, P. G. Catalytic Synthesis of PEGylated EGCG Conjugates that Disaggregate Alzheimer's Tau. *Synthesis* **2021**, *53* (22), 4263-4271.

[14] Singh, J.; Petter, R. C.; Tester, R. W.; Kluge, A. F.; Mazdiyasni, H.; Westin III, W. F.; Niu, D.; Qiao, L. Heteroaryl compounds and uses thereof. Google Patents: 2013.

[15] Mencio, C. P.; Garud, D. R.; Doi, Y.; Bi, Y.; Vankayalapati, H.; Koketsu, M.; Kuberan, B. Ruthenium (II)-and copper (I)-catalyzed synthesis of click-xylosides and assessment of their glycosaminoglycan priming activity. *Bioorg. Med. Chem. Lett.* **2017**, *27* (22), 5027-5030.

[16] Bansal, A.; Bilaspuri, G. Effect of ferrous sulphate and ascorbic acid on motility, viability and lipid peroxidation of crossbred cattle bull spermatozoa. *Animal* **2008**, *2* (1), 100-104.

[17] Korkmaz, F.; Malama, E.; Siuda, M.; Leiding, C.; Bollwein, H. Effects of sodium pyruvate on viability, synthesis of reactive oxygen species, lipid peroxidation and DNA integrity of cryopreserved bovine sperm. *Anim. Reprod. Sci.* **2017**, *185*, 18-27.

[18] Das, K.; Roychoudhury, A. Reactive oxygen species (ROS) and response of antioxidants as ROS-scavengers during environmental stress in plants. *Front. Environ. Sci.* **2014**, *2*, 53.

12. NMR Spectra

Compound 19

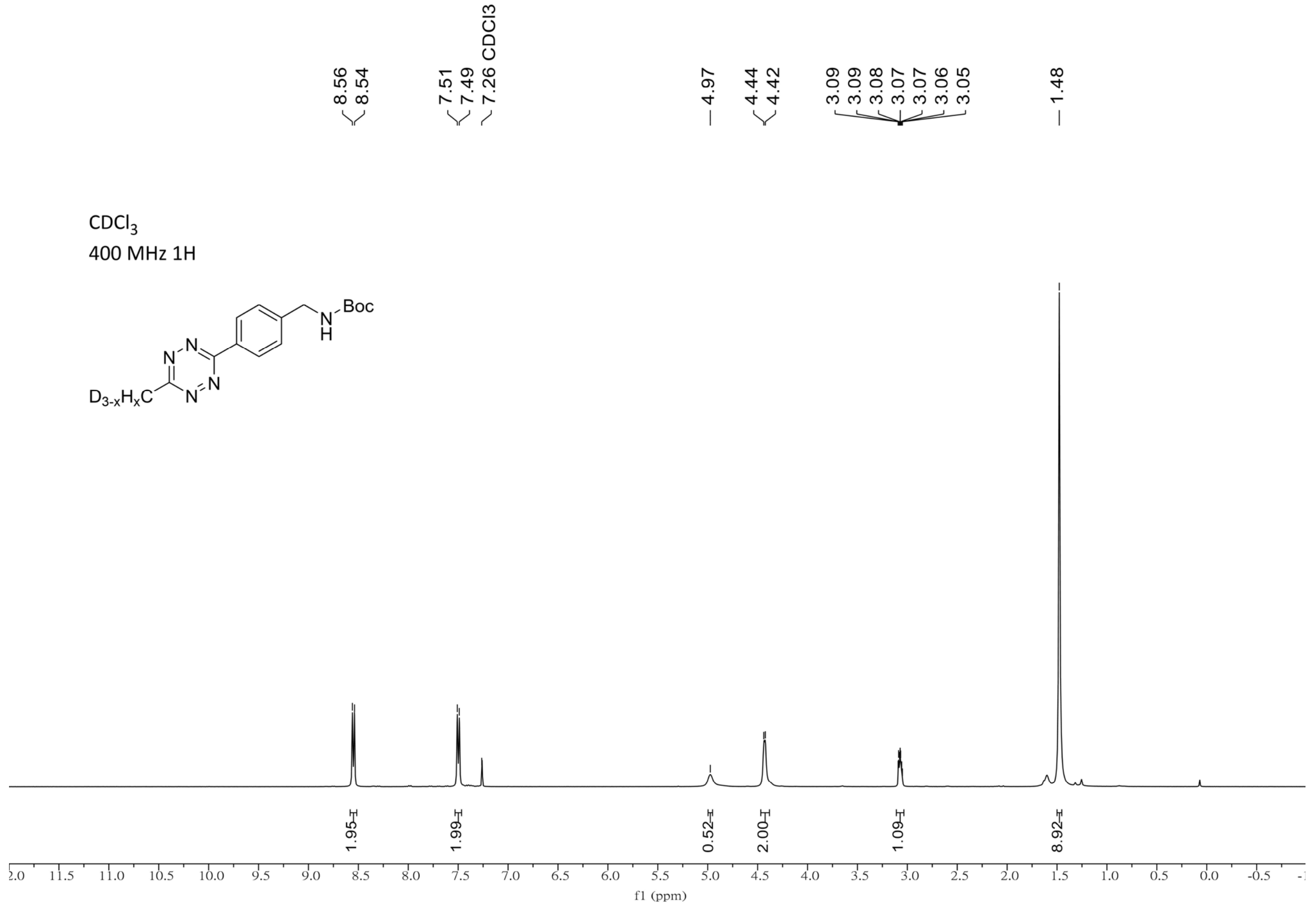

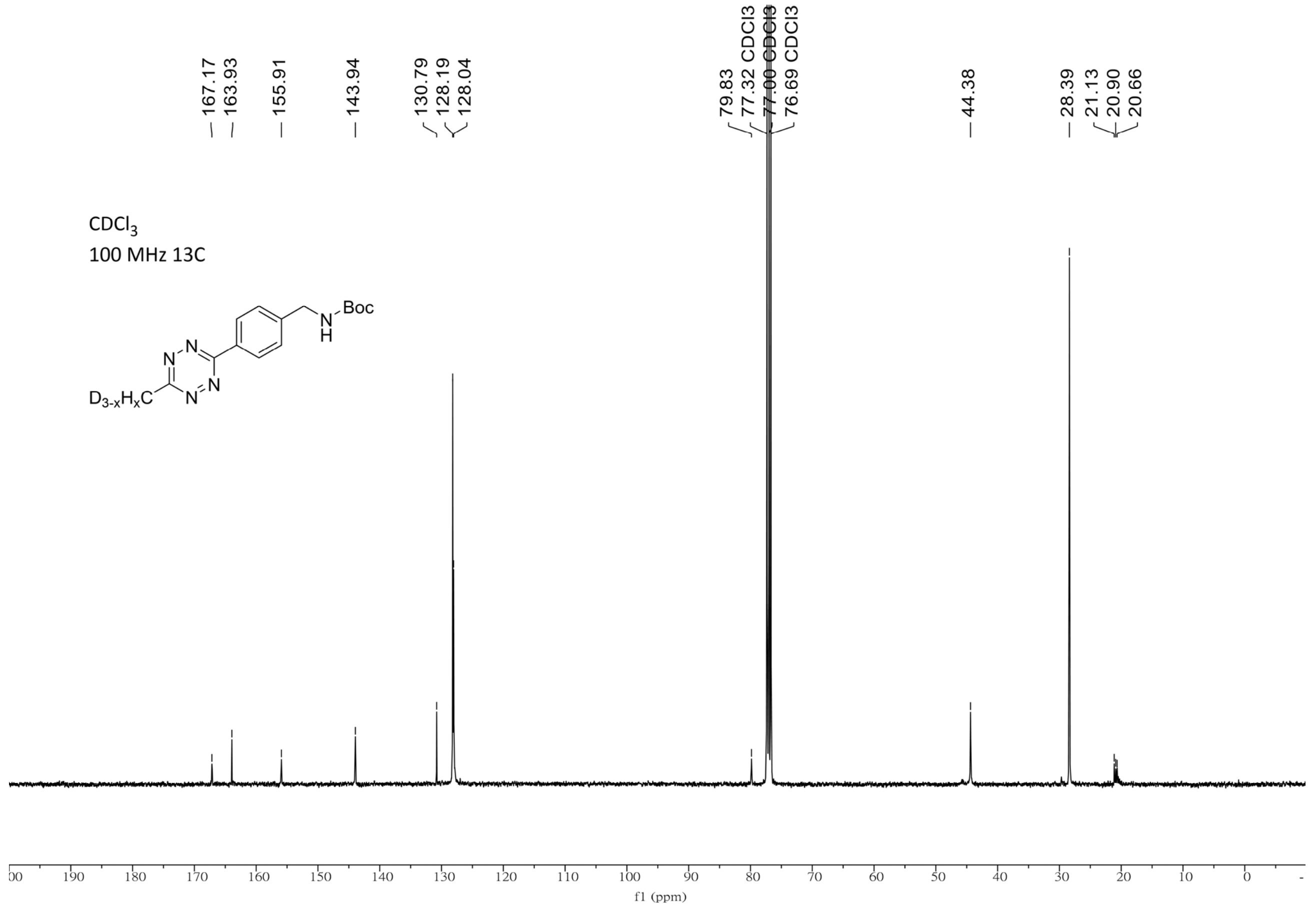

Tz-PPh$_3$ (1)

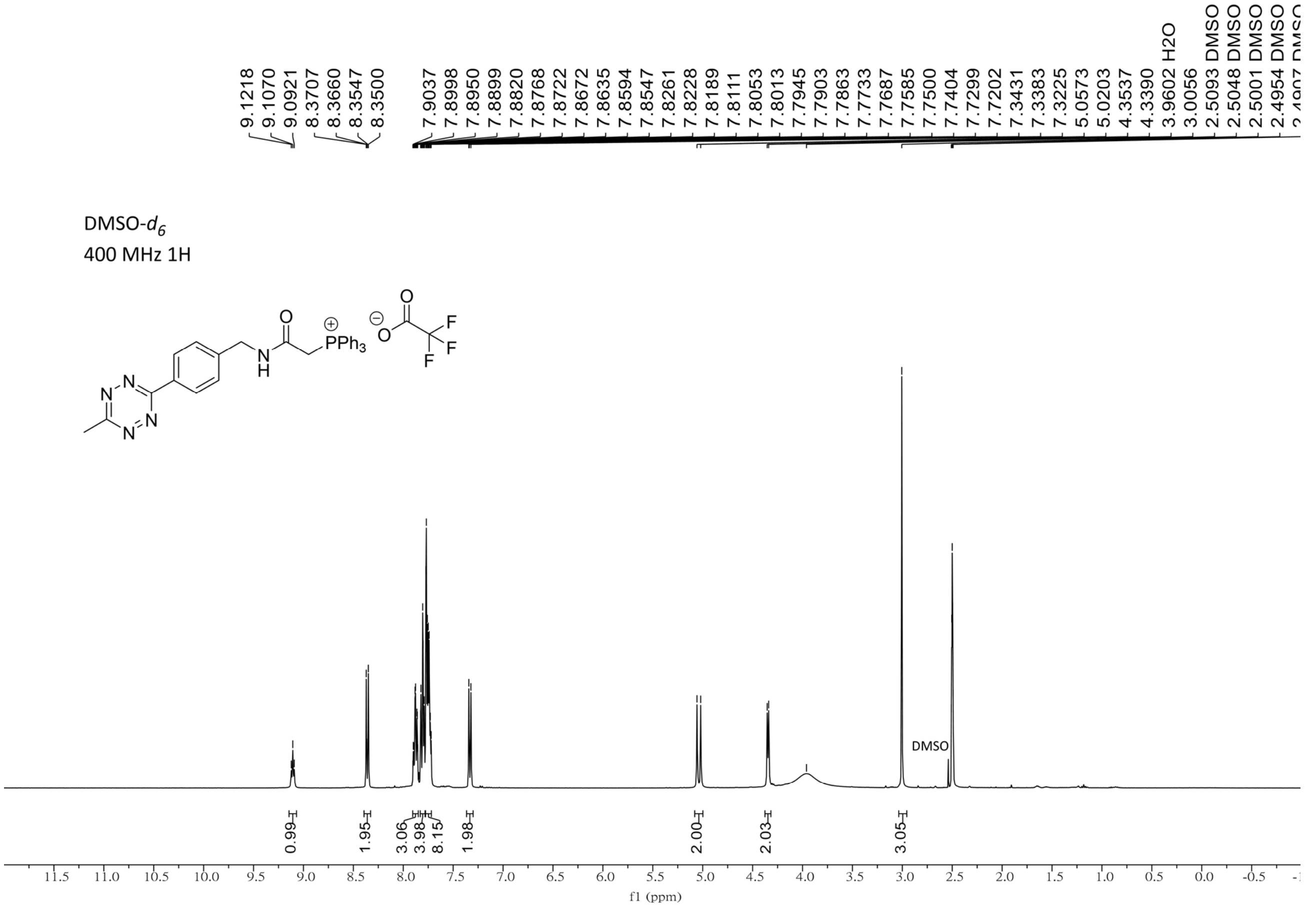

9.1218
9.1070
9.0921
8.3707
8.3660
8.3547
8.3500
7.9037
7.8998
7.8950
7.8899
7.8820
7.8768
7.8722
7.8672
7.8635
7.8594
7.8547
7.8261
7.8228
7.8189
7.8111
7.8053
7.8013
7.7945
7.7903
7.7863
7.7733
7.7687
7.7585
7.7500
7.7404
7.7299
7.7202
7.3431
7.3383
7.3225
5.0573
5.0203
4.3537
4.3390
3.9602 H2O
3.0056
2.5093 DMSO
2.5048 DMSO
2.5001 DMSO
2.4954 DMSO
DMSO-d6
400 MHz 1H
DMSO
0.99
1.95
3.06
3.98
8.15
1.98
2.00
2.03
3.05
f1 (ppm)

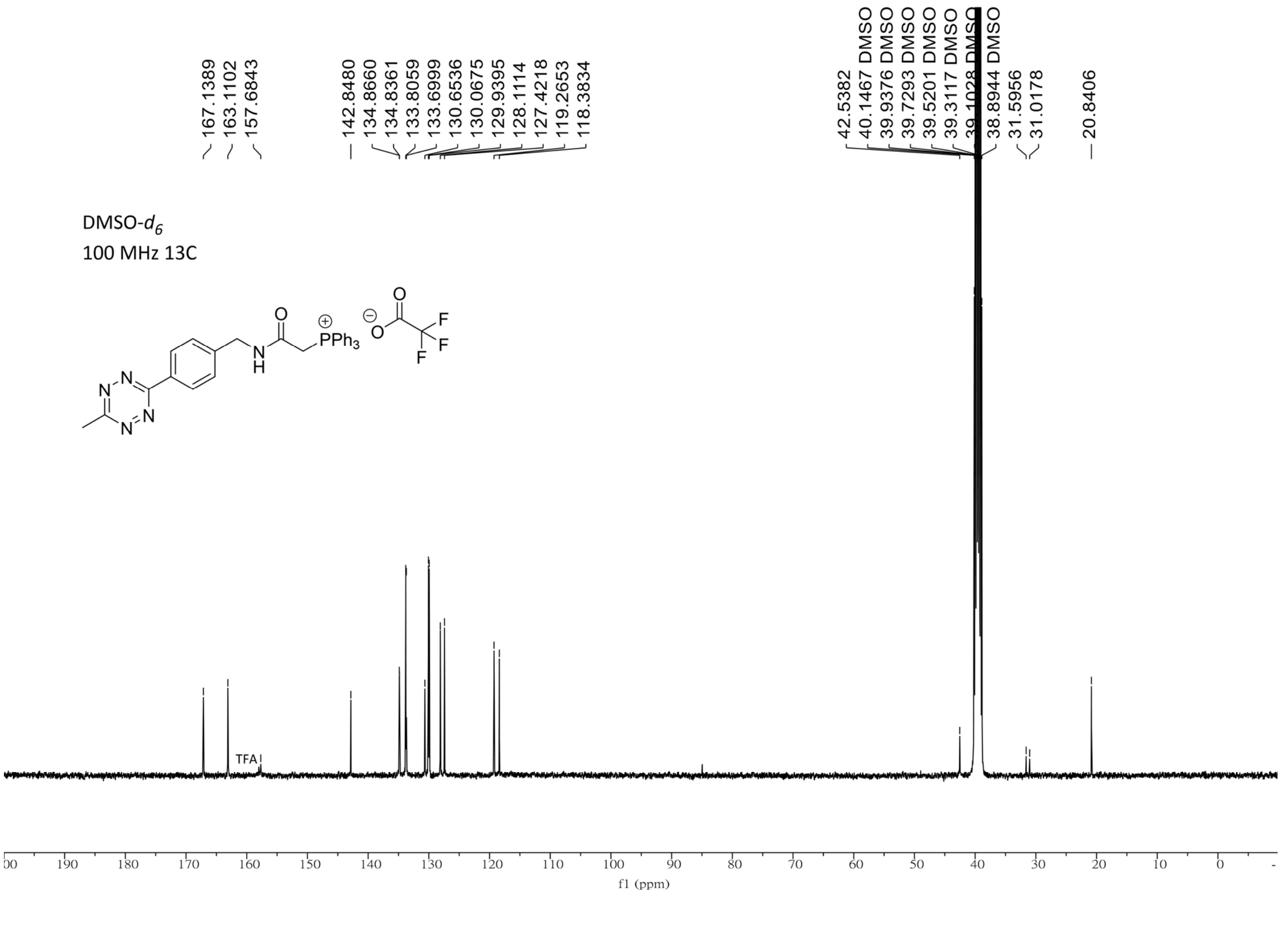

167.1389
163.1102
157.6843
142.8480
134.8660
134.8361
133.8059
133.6999
130.6536
130.0675
129.9395
128.1114
127.4218
119.2653
118.3834
42.5382
40.1467 DMSO
39.9376 DMSO
39.7293 DMSO
39.5201 DMSO
39.3117 DMSO
39.1028 DMSO
38.8944 DMSO
31.5956
31.0178
20.8406
DMSO-d6
100 MHz 13C
TFA
f1 (ppm)

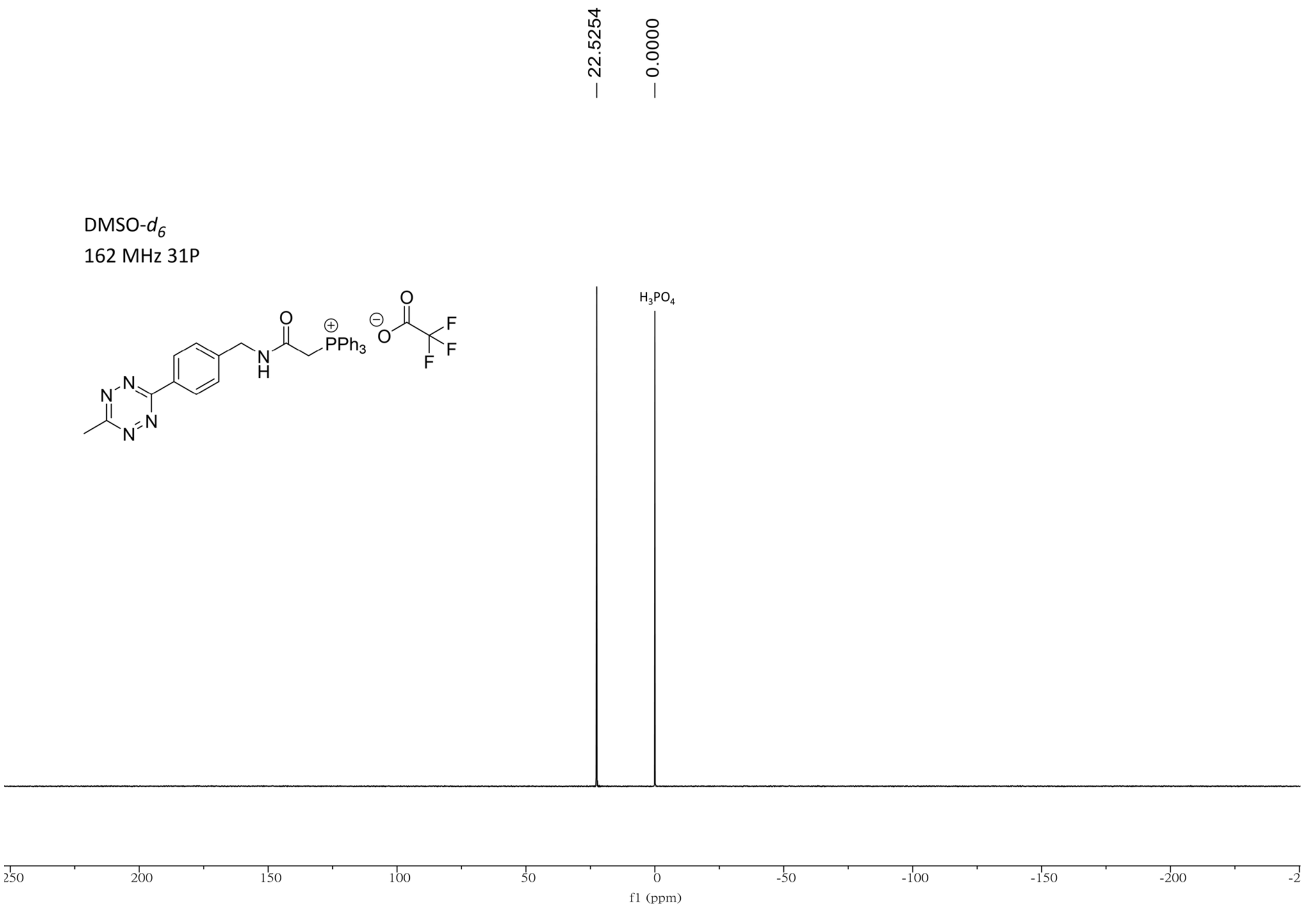
22.5254
0.0000
DMSO-d_6
162 MHz 31P
H_3PO_4
250
200
150
100
50
0
-50
-100
-150
-200
f1 (ppm)

D-Tz-PPh$_3$ (3)

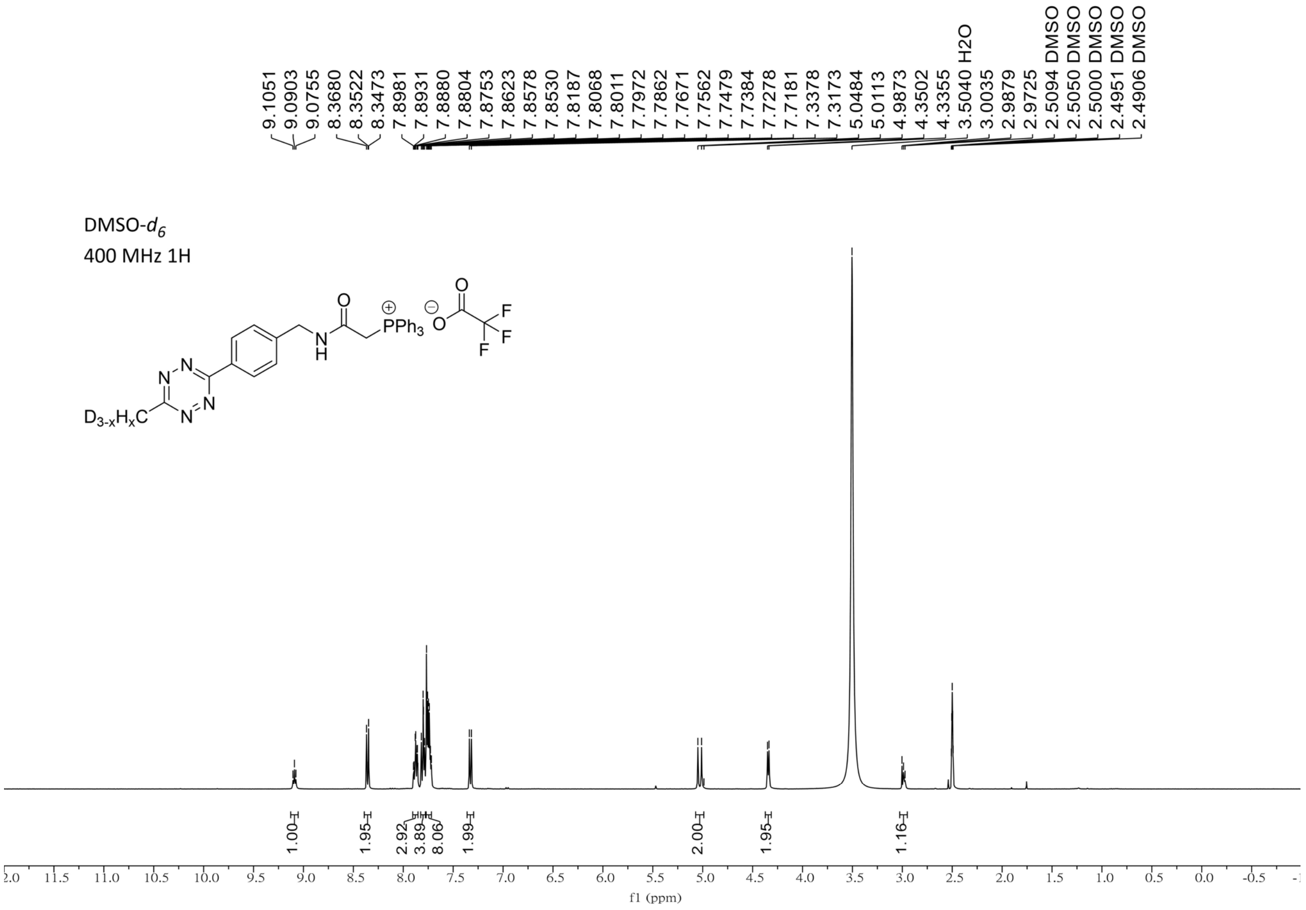
9.1051
9.0903
9.0755
8.3680
8.3522
8.3473
7.8981
7.8931
7.8880
7.8804
7.8753
7.8623
7.8578
7.8530
7.8187
7.8068
7.8011
7.7972
7.7862
7.7671
7.7562
7.7479
7.7384
7.7278
7.7181
7.3378
7.3173
5.0484
5.0113
4.9873
4.3502
4.3355
3.5040 H2O
3.0035
2.9879
2.9725
2.5094 DMSO
2.5050 DMSO
2.5000 DMSO
2.4951 DMSO
2.4906 DMSO
DMSO-d6
400 MHz 1H
D3-xHxC
PPh3
F
1.00
1.95
2.92
3.89
8.06
1.99
2.00
1.95
1.16
f1 (ppm)

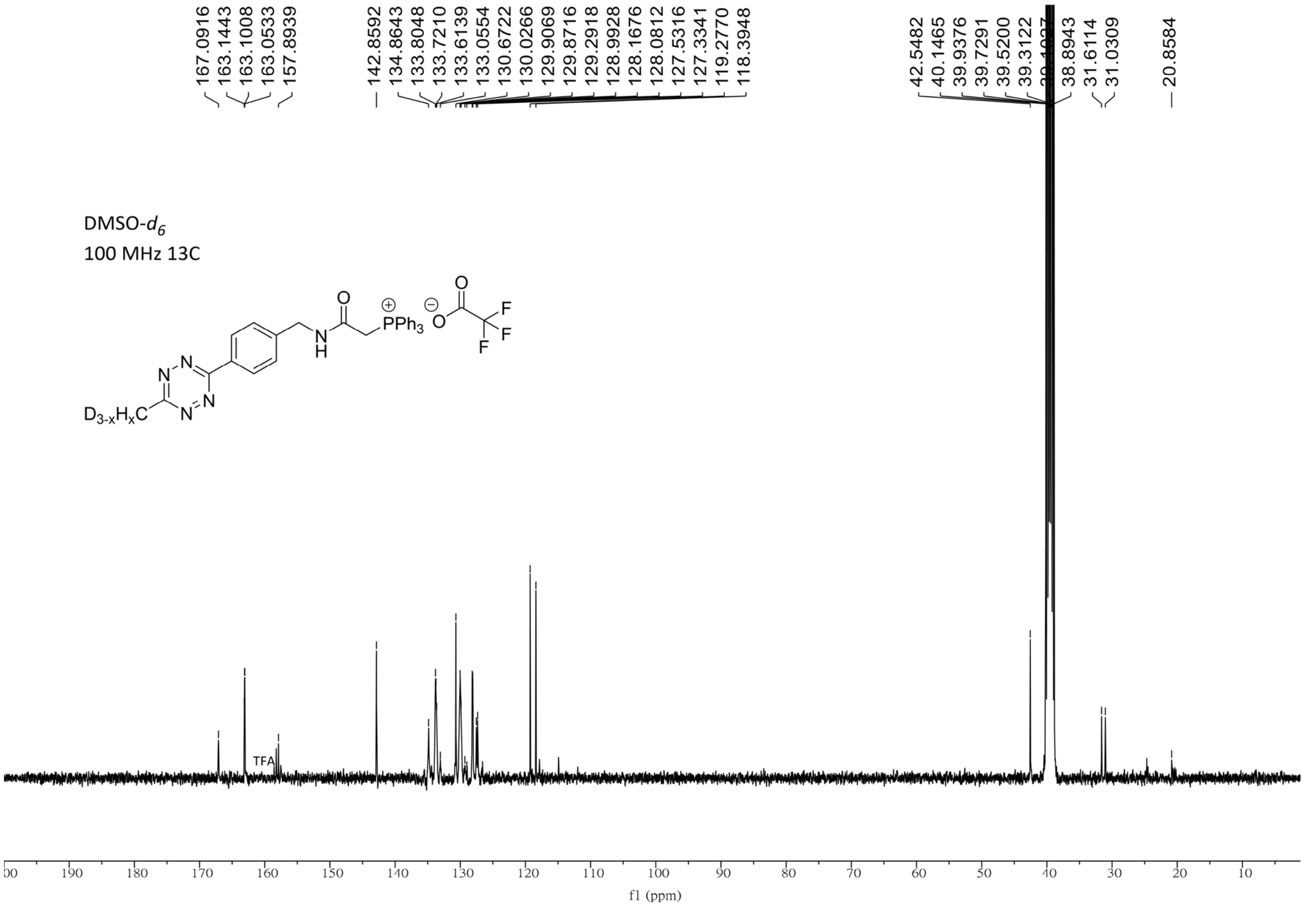
167.0916
163.1443
163.1008
163.0533
157.8939
142.8592
134.8643
133.8048
133.7210
133.6139
133.0554
130.6722
130.0266
129.9069
129.8716
129.2918
128.9928
128.1676
128.0812
127.5316
127.3341
119.2770
118.3948
42.5482
40.1465
39.9376
39.7291
39.5200
39.3122
38.8943
31.6114
31.0309
20.8584
DMSO-d6
100 MHz 13C
D3-xHxC
PPh3
F
TFA
f1 (ppm)

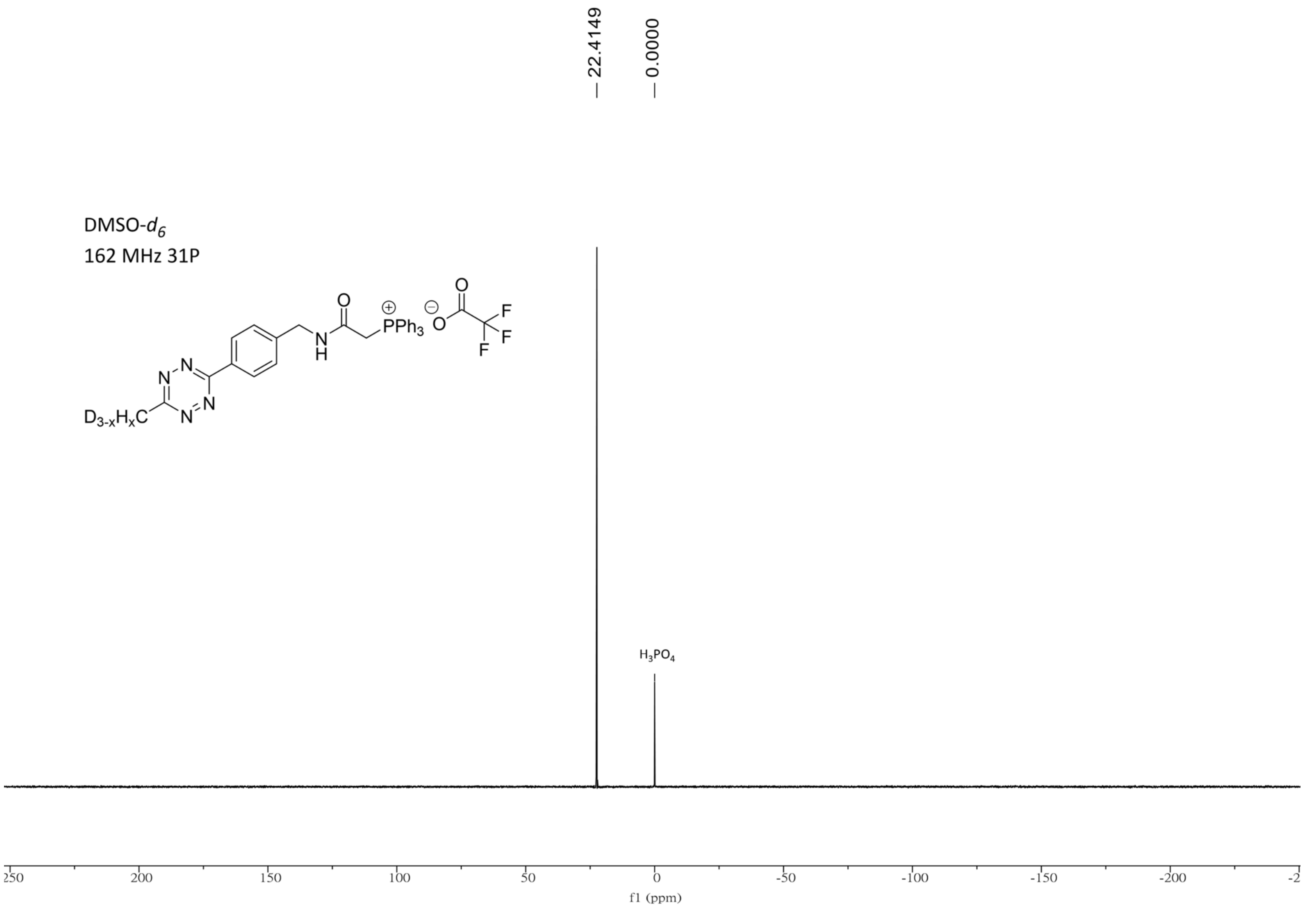

22.4149
0.0000
DMSO-d_6
162 MHz 31P
$D_{3-x}H_xC$
PPh3
H_3PO_4
f1 (ppm)

Compound 23

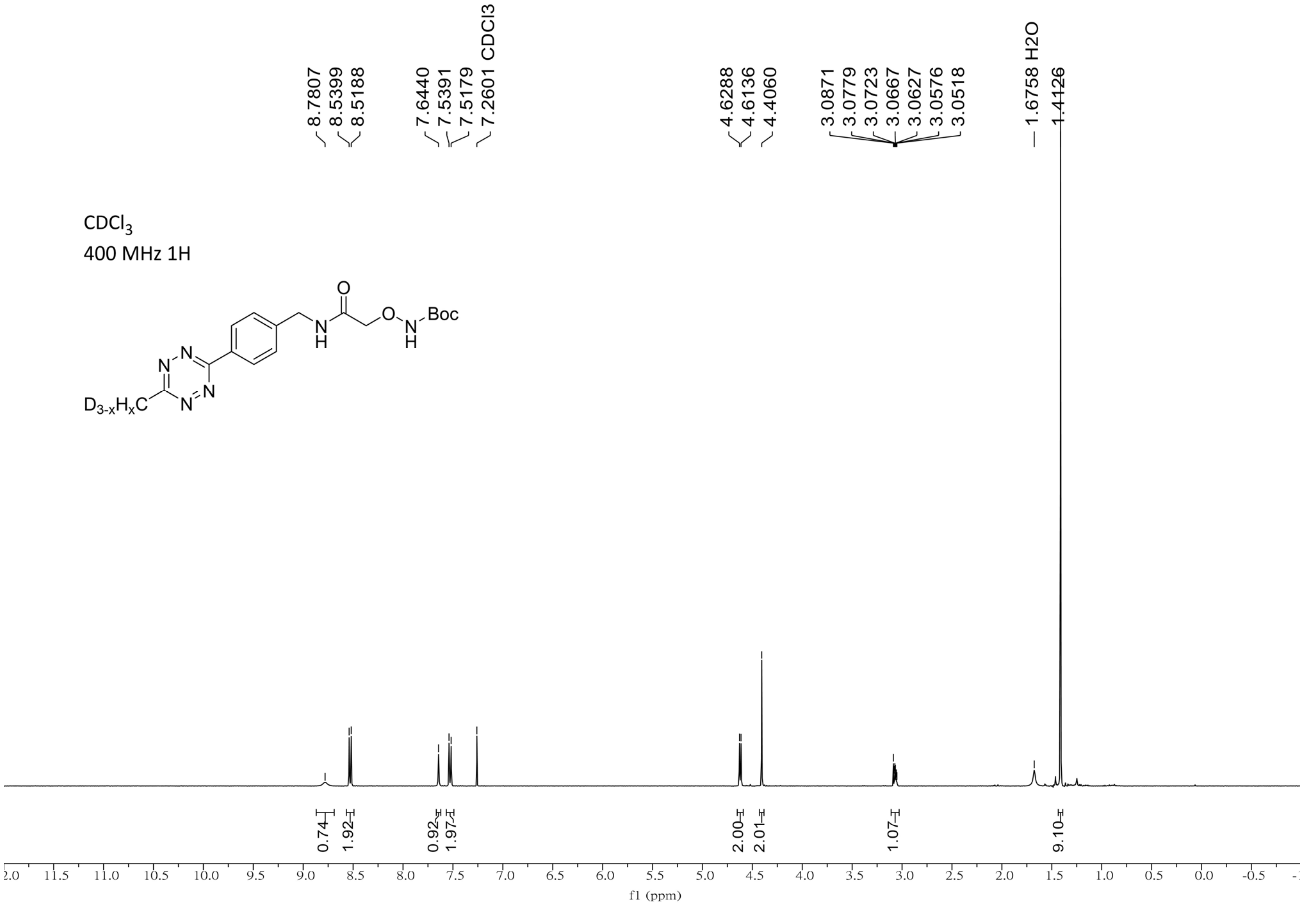

8.7807
8.5399
8.5188
7.6440
7.5391
7.5179
7.2601 CDCl3
4.6288
4.6136
4.4060
3.0871
3.0779
3.0723
3.0667
3.0627
3.0576
3.0518
1.6758 H2O
1.4126
CDCl3
400 MHz 1H
Boc
0.74
1.92
0.92
1.97
2.00
2.01
1.07
9.10
f1 (ppm)

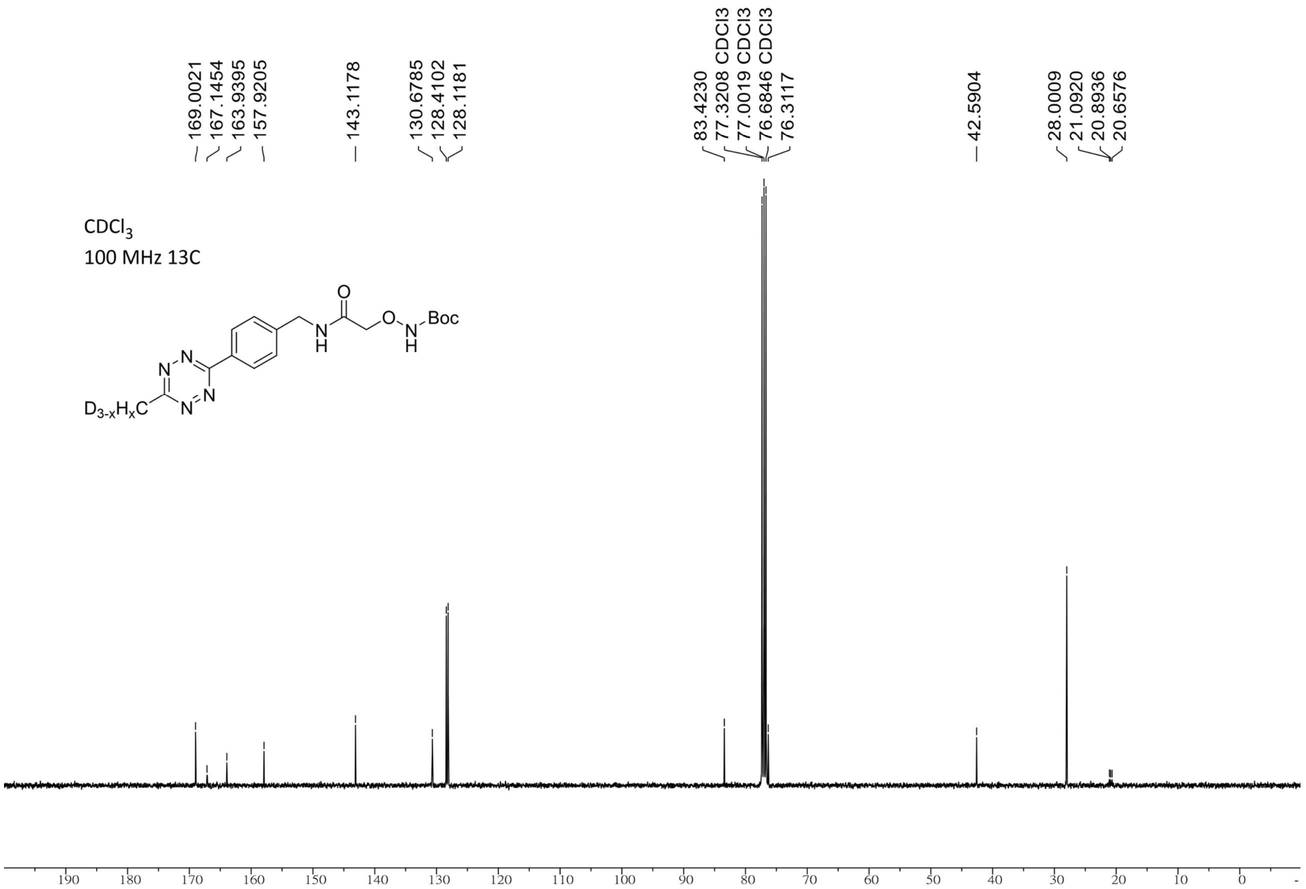

169.0021
167.1454
163.9395
157.9205
143.1178
130.6785
128.4102
128.1181
83.4230
77.3208 CDCl3
77.0019 CDCl3
76.6846 CDCl3
76.3117
42.5904
28.0009
21.0920
20.8936
20.6576
CDCl3
100 MHz 13C
Boc
f1 (ppm)

D-Tz-ONH$_2$ (4)

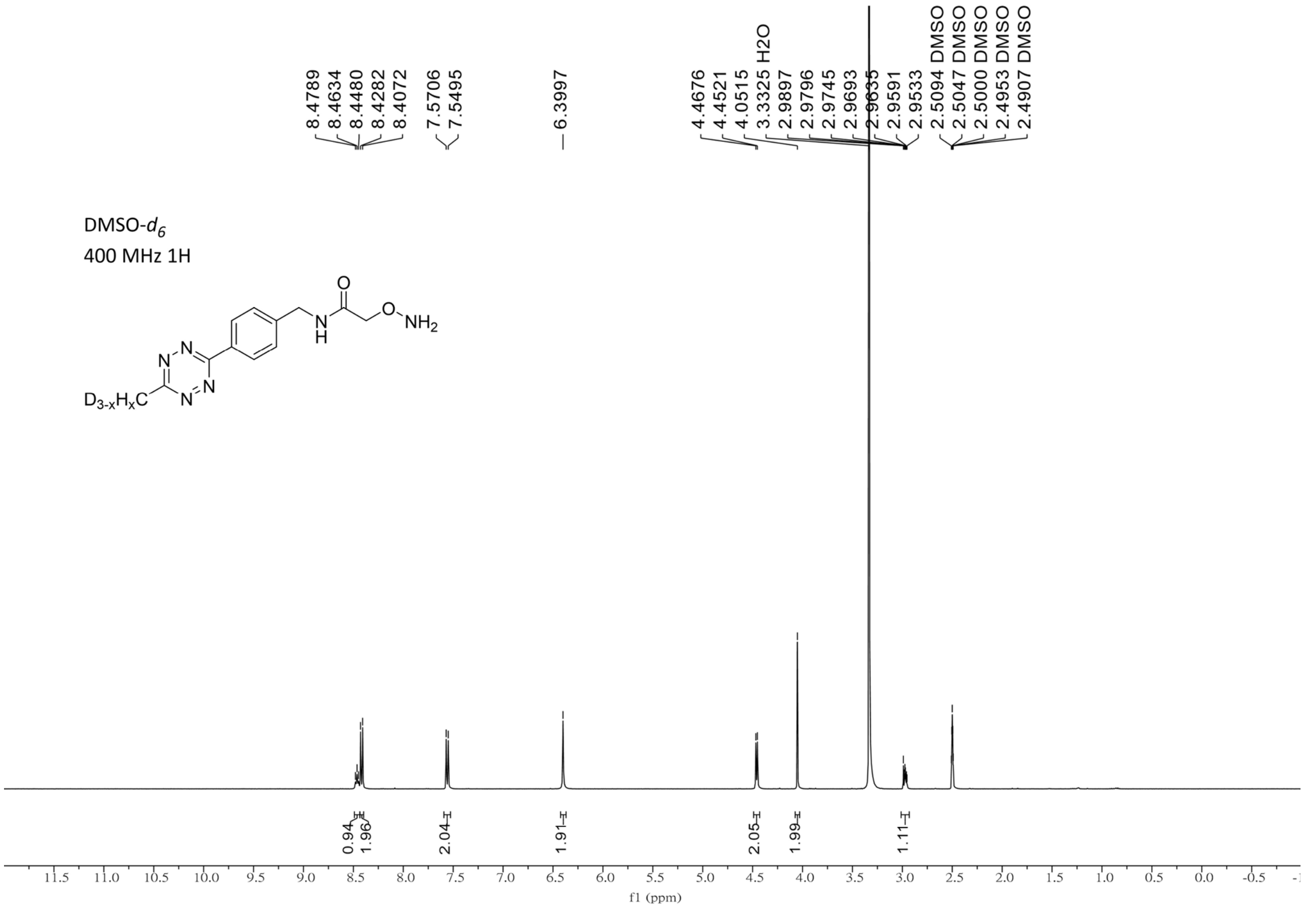

8.4789
8.4634
8.4480
8.4282
8.4072
7.5706
7.5495
6.3997
4.4676
4.4521
4.0515
3.3325 H2O
2.9897
2.9796
2.9745
2.9693
2.9635
2.9591
2.9533
2.5094 DMSO
2.5047 DMSO
2.5000 DMSO
2.4953 DMSO
2.4907 DMSO
DMSO-d6
400 MHz 1H
O
N
H
O
NH2
N
N
N
N
D3-xHxC
0.94
1.96
2.04
1.91
2.05
1.99
1.11
11.5 11.0 10.5 10.0 9.5 9.0 8.5 8.0 7.5 7.0 6.5 6.0 5.5 5.0 4.5 4.0 3.5 3.0 2.5 2.0 1.5 1.0 0.5 0.0 -0.5
f1 (ppm)

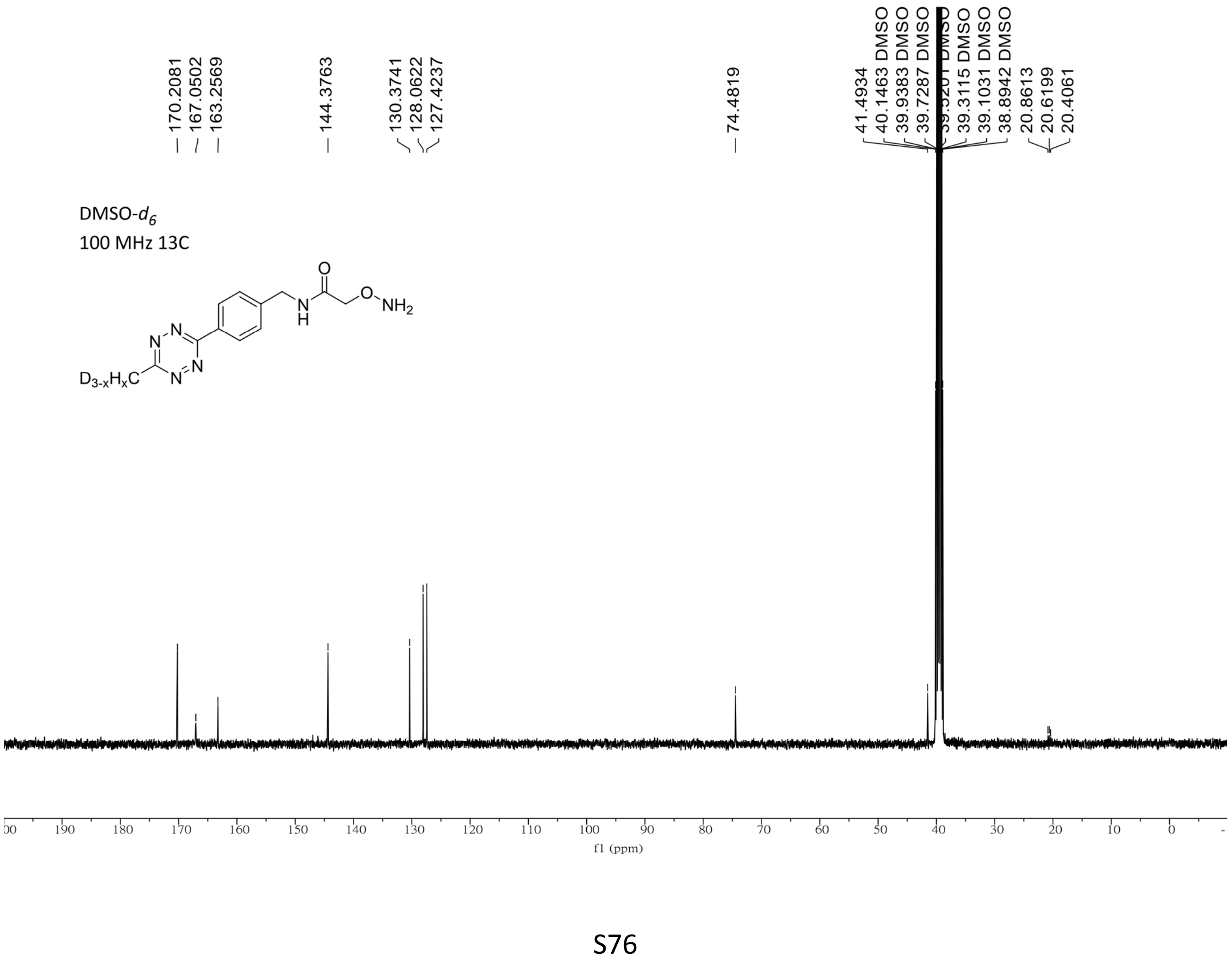

170.2081
167.0502
163.2569
144.3763
130.3741
128.0622
127.4237
74.4819
41.4934
40.1463 DMSO
39.9383 DMSO
39.7287 DMSO
39.5201 DMSO
39.3115 DMSO
39.1031 DMSO
38.8942 DMSO
20.8613
20.6199
20.4061
DMSO-d6
100 MHz 13C
O
N
H
O
NH2
N
N
N
N
D3-xHxC
190 180 170 160 150 140 130 120 110 100 90 80 70 60 50 40 30 20 10 0
f1 (ppm)

TCO-SS-biotin (5)

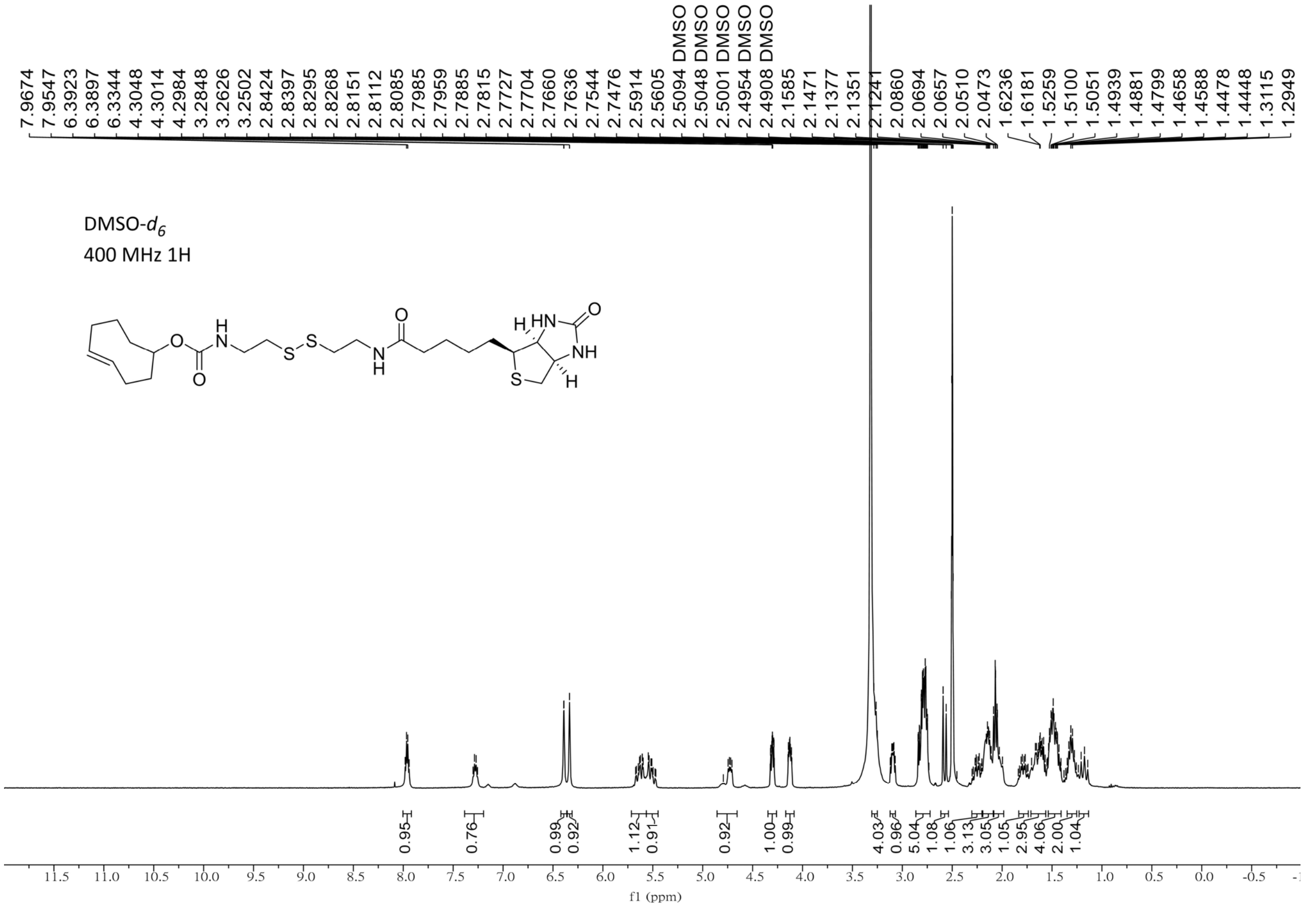

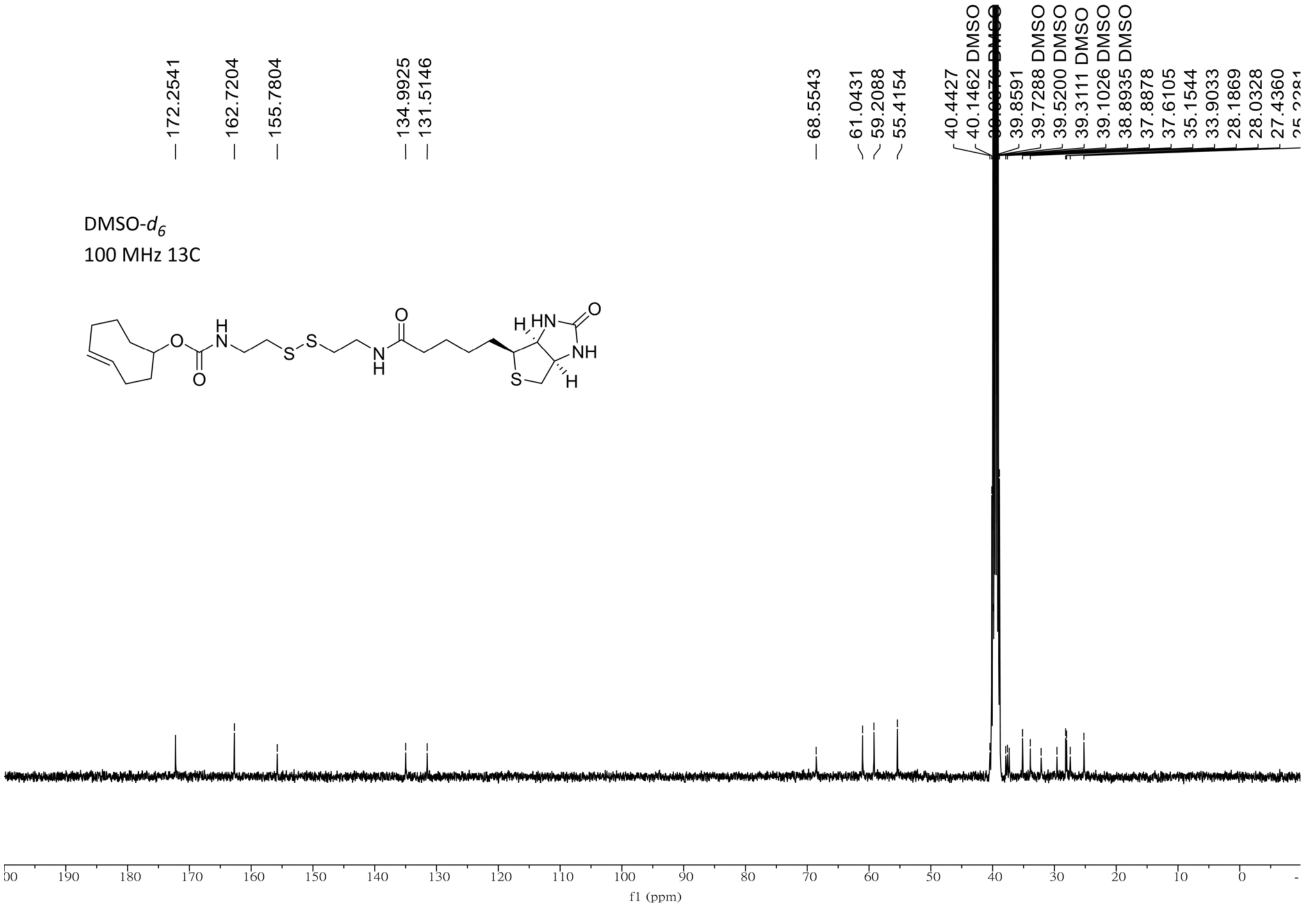

azido-SS-biotin (15)

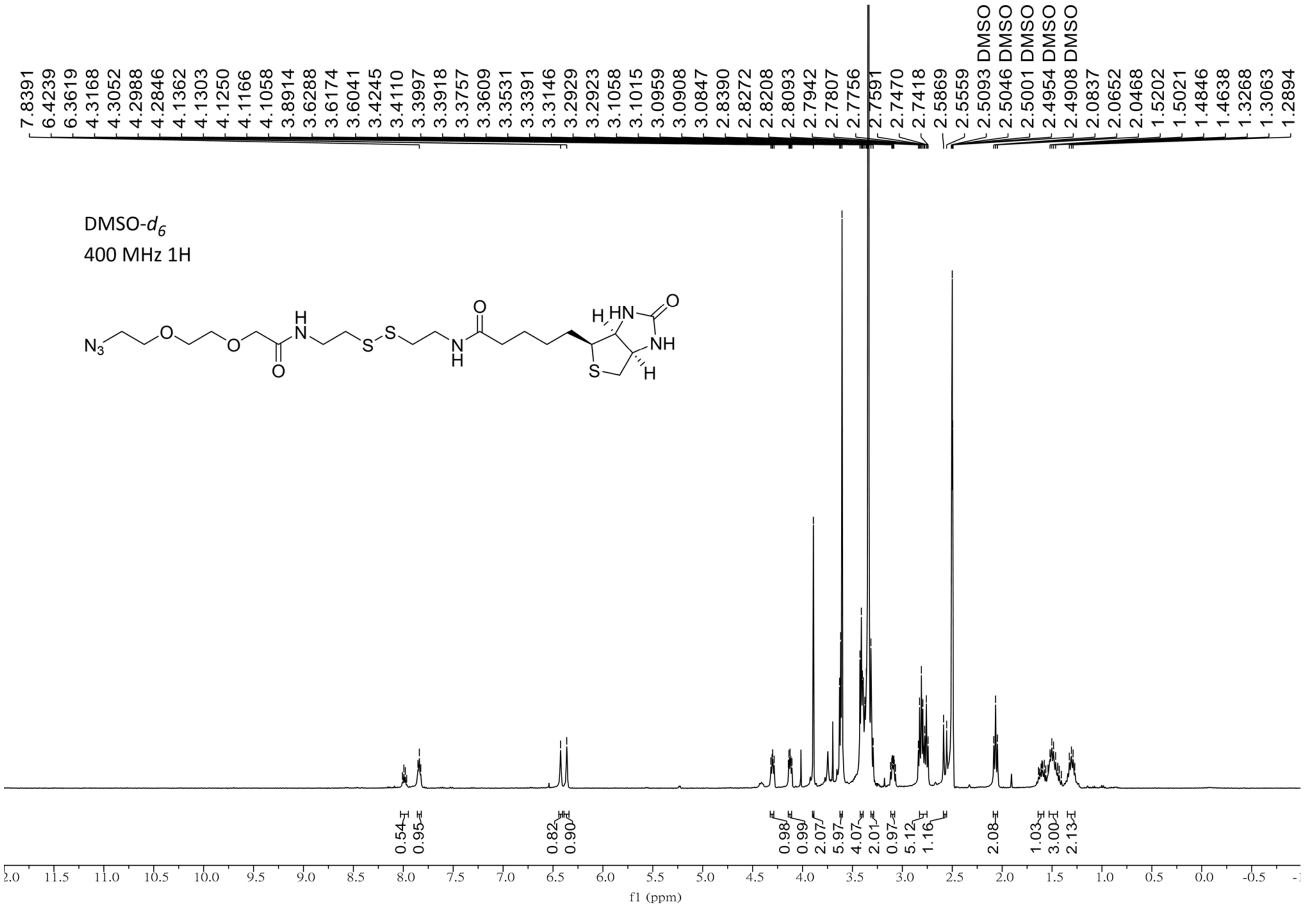

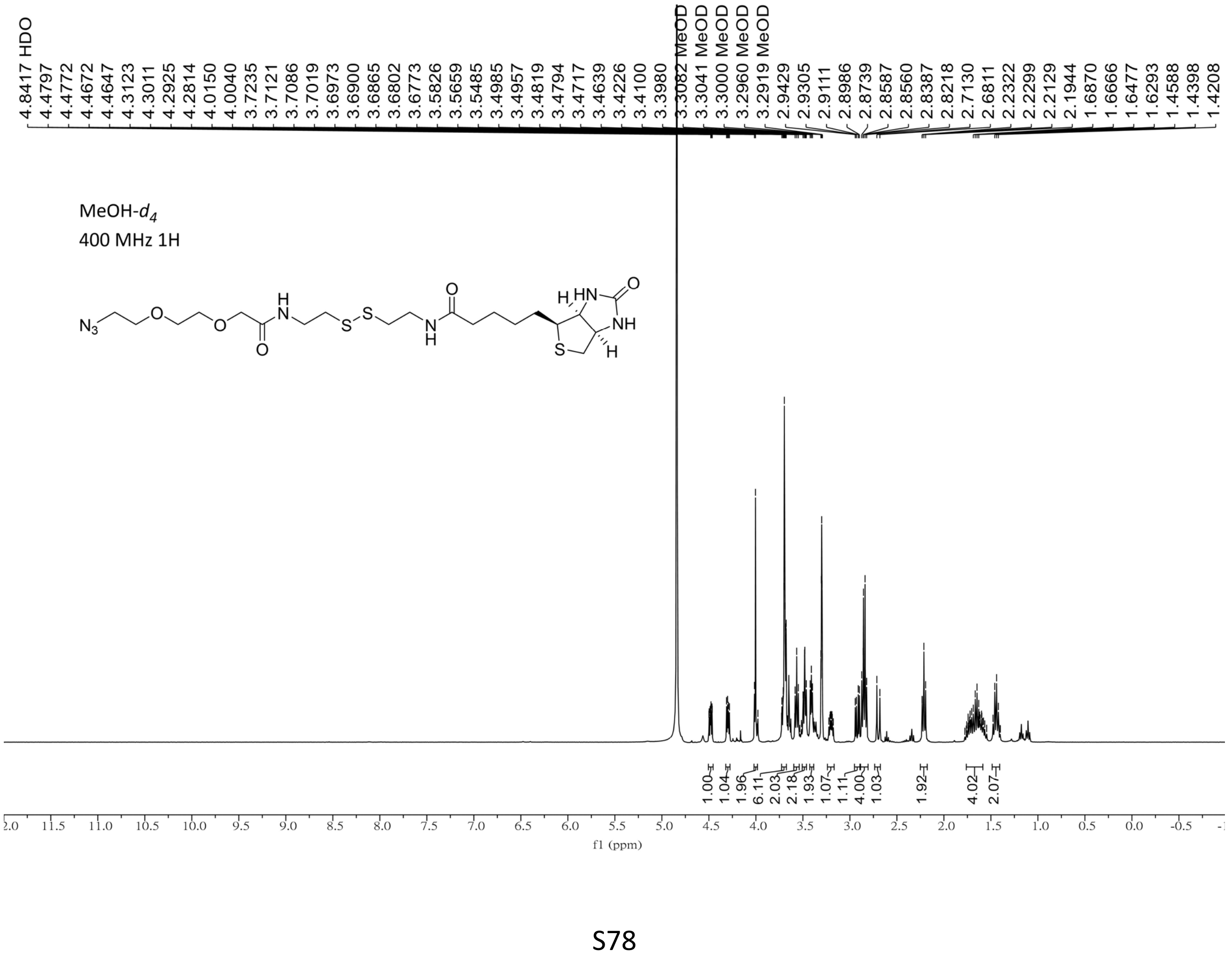

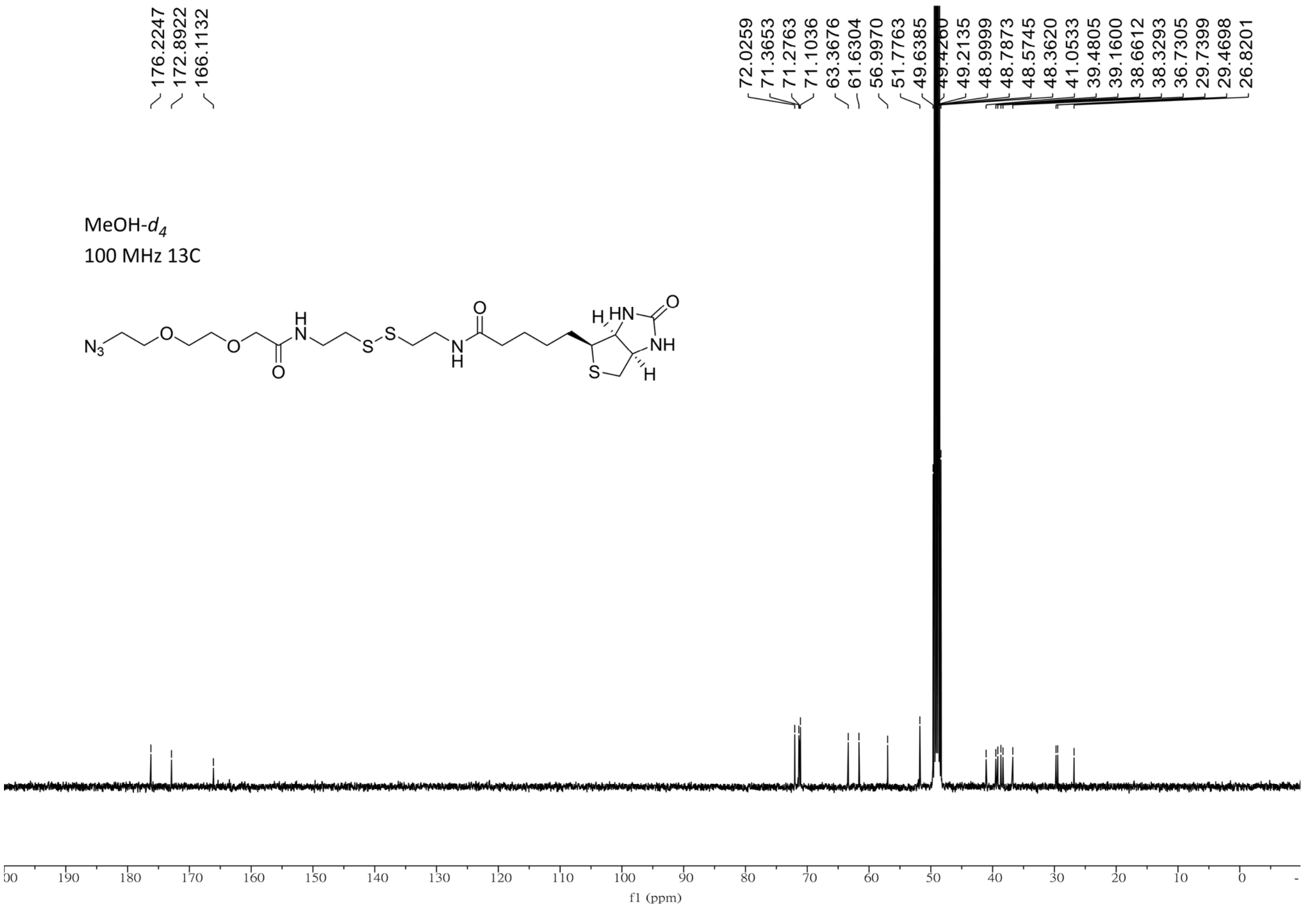
176.2247
172.8922
166.1132
72.0259
71.3653
71.2763
71.1036
63.3676
61.6304
56.9970
51.7763
49.6385
49.4260
49.2135
48.9999
48.7873
48.5745
48.3620
41.0533
39.4805
39.1600
38.6612
38.3293
36.7305
29.7399
29.4698
26.8201
MeOH-d_4
100 MHz 13C
N_3
O
O
H
N
O
S
S
N
H
O
H
HN
O
NH
S
H
f1 (ppm)

Compound 27

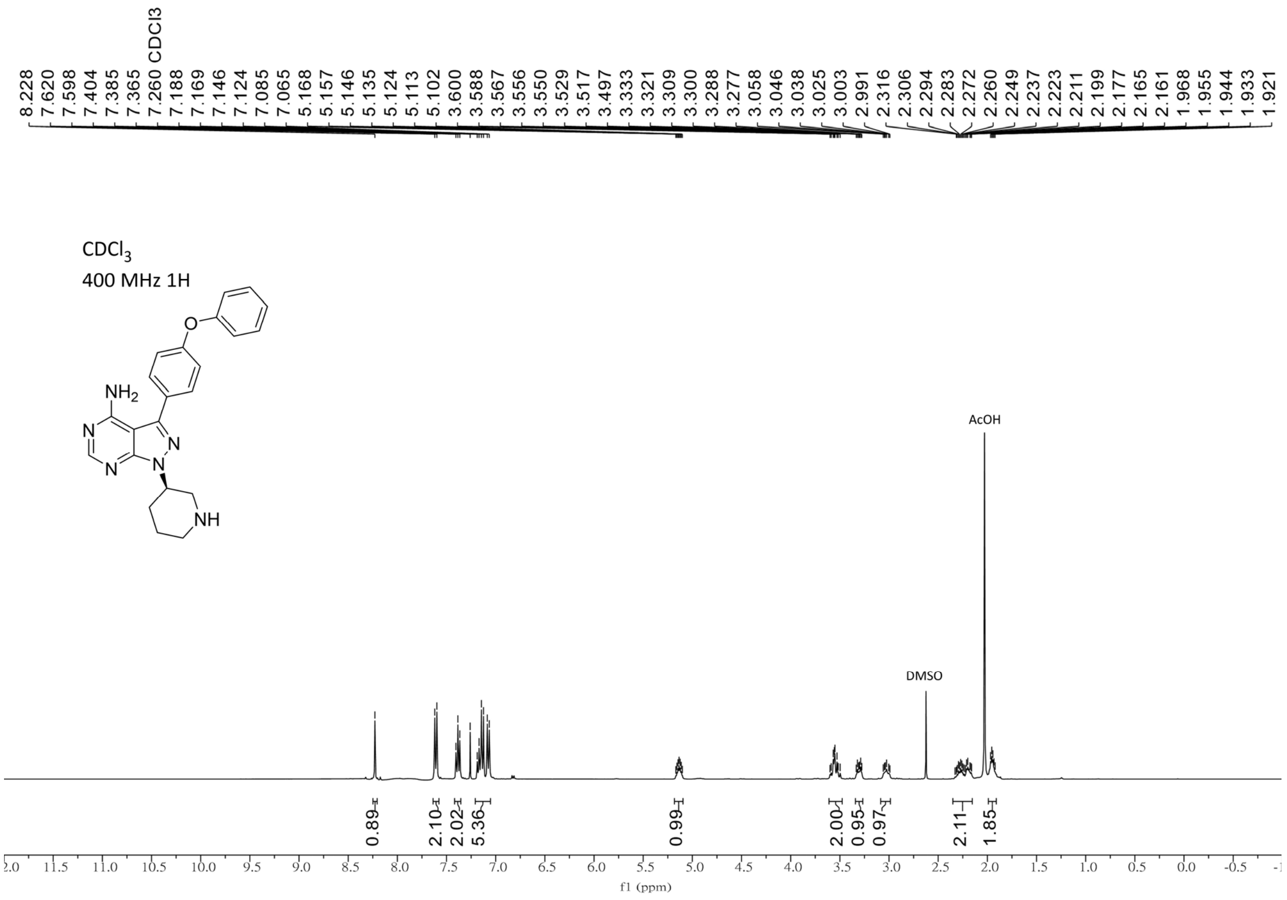
8.228
7.620
7.598
7.404
7.385
7.365
7.260 CDCl3
7.188
7.169
7.146
7.124
7.085
7.065
5.168
5.157
5.146
5.135
5.124
5.113
5.102
3.600
3.588
3.567
3.556
3.550
3.529
3.517
3.497
3.333
3.321
3.309
3.300
3.288
3.277
3.058
3.046
3.038
3.025
3.003
2.991
2.316
2.306
2.294
2.283
2.272
2.260
2.249
2.237
2.223
2.211
2.199
2.177
2.165
2.161
1.968
1.955
1.944
1.933
1.921
CDCl3
400 MHz 1H
NH2
N
N
N
N
O
NH
AcOH
DMSO
0.89
2.10
2.02
5.36
0.99
2.00
0.95
0.97
2.11
1.85
f1 (ppm)

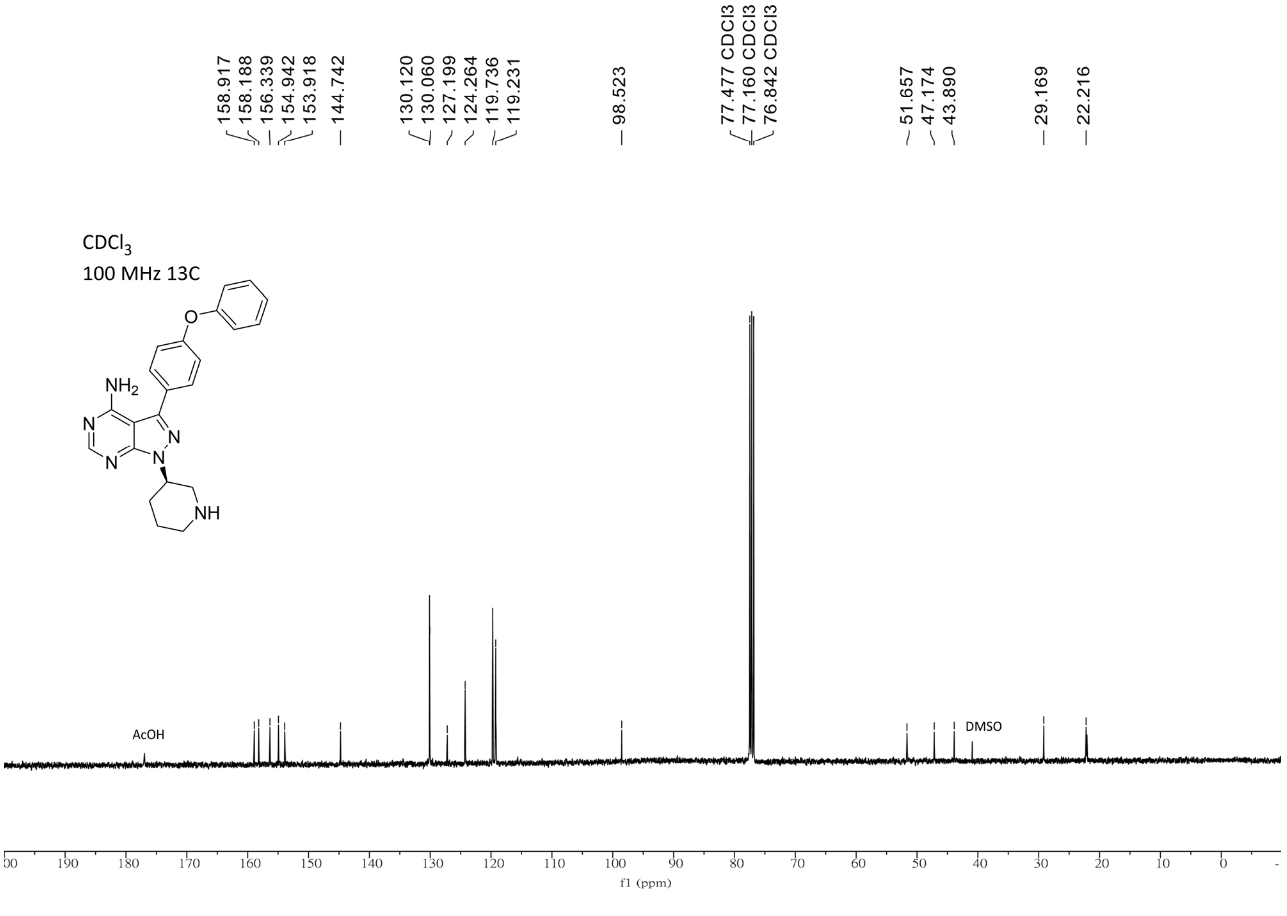
158.917
158.188
156.339
154.942
153.918
144.742
130.120
130.060
127.199
124.264
119.736
119.231
98.523
77.477 CDCl3
77.160 CDCl3
76.842 CDCl3
51.657
47.174
43.890
29.169
22.216
CDCl3
100 MHz 13C
NH2
N
N
N
N
O
NH
AcOH
DMSO
f1 (ppm)

Compound 28

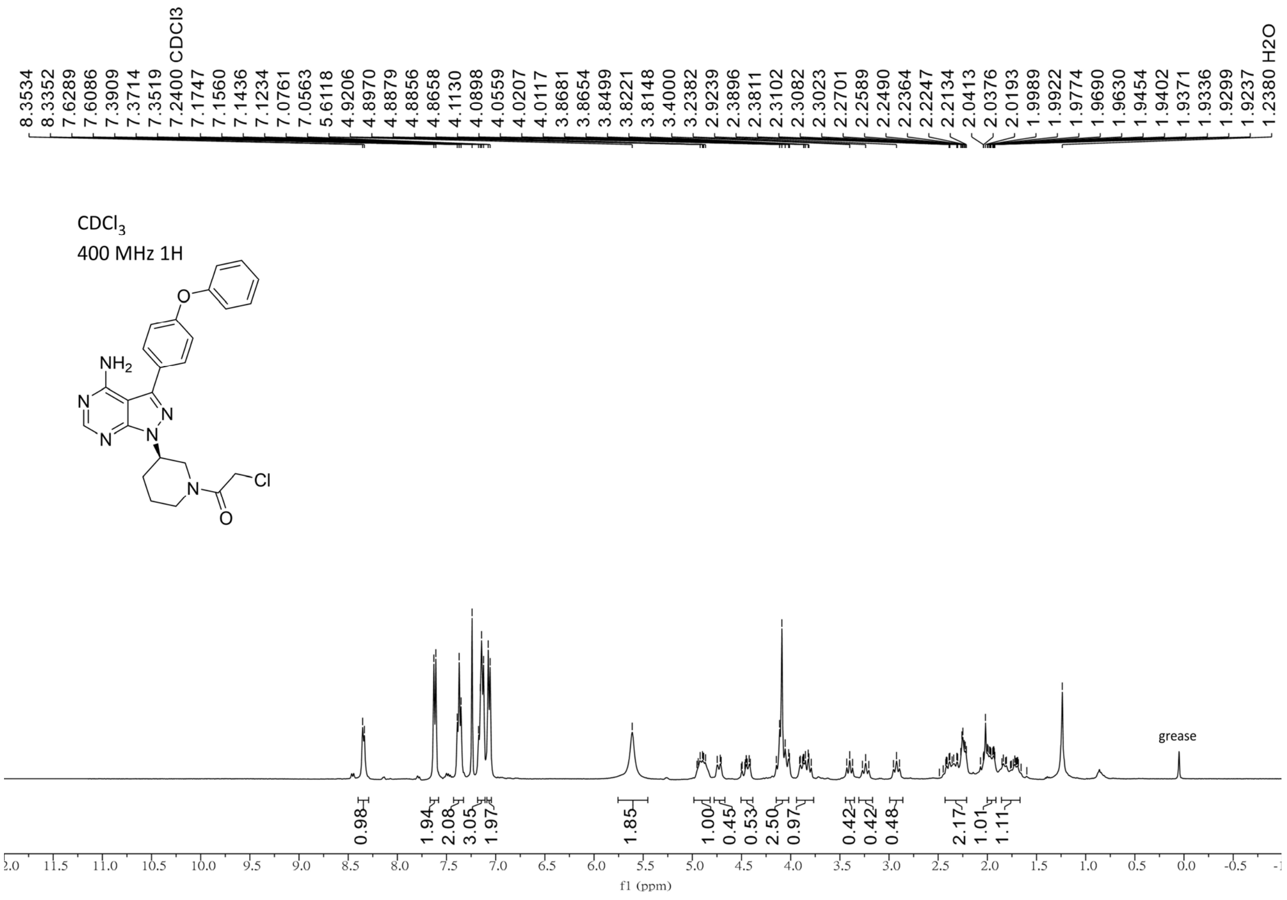
8.3534
8.3352
7.6289
7.6086
7.3909
7.3714
7.3519
7.2400 CDCl3
7.1747
7.1560
7.1436
7.1234
7.0761
7.0563
5.6118
4.9206
4.8970
4.8879
4.8856
4.8658
4.1130
4.0898
4.0559
4.0207
4.0117
3.8681
3.8654
3.8499
3.8221
3.8148
3.4000
3.2382
2.9239
2.3896
2.3811
2.3102
2.3082
2.3023
2.2701
2.2589
2.2490
2.2364
2.2247
2.2134
2.0413
2.0376
2.0193
1.9989
1.9922
1.9774
1.9690
1.9630
1.9454
1.9402
1.9371
1.9336
1.9299
1.9237
1.2380 H2O
CDCl3
400 MHz 1H
NH2
N
N
N
N
O
N
Cl
O
grease
0.98
1.94
2.08
3.05
1.97
1.85
1.00
0.45
0.53
2.50
0.97
0.42
0.42
0.48
2.17
1.01
1.11
f1 (ppm)

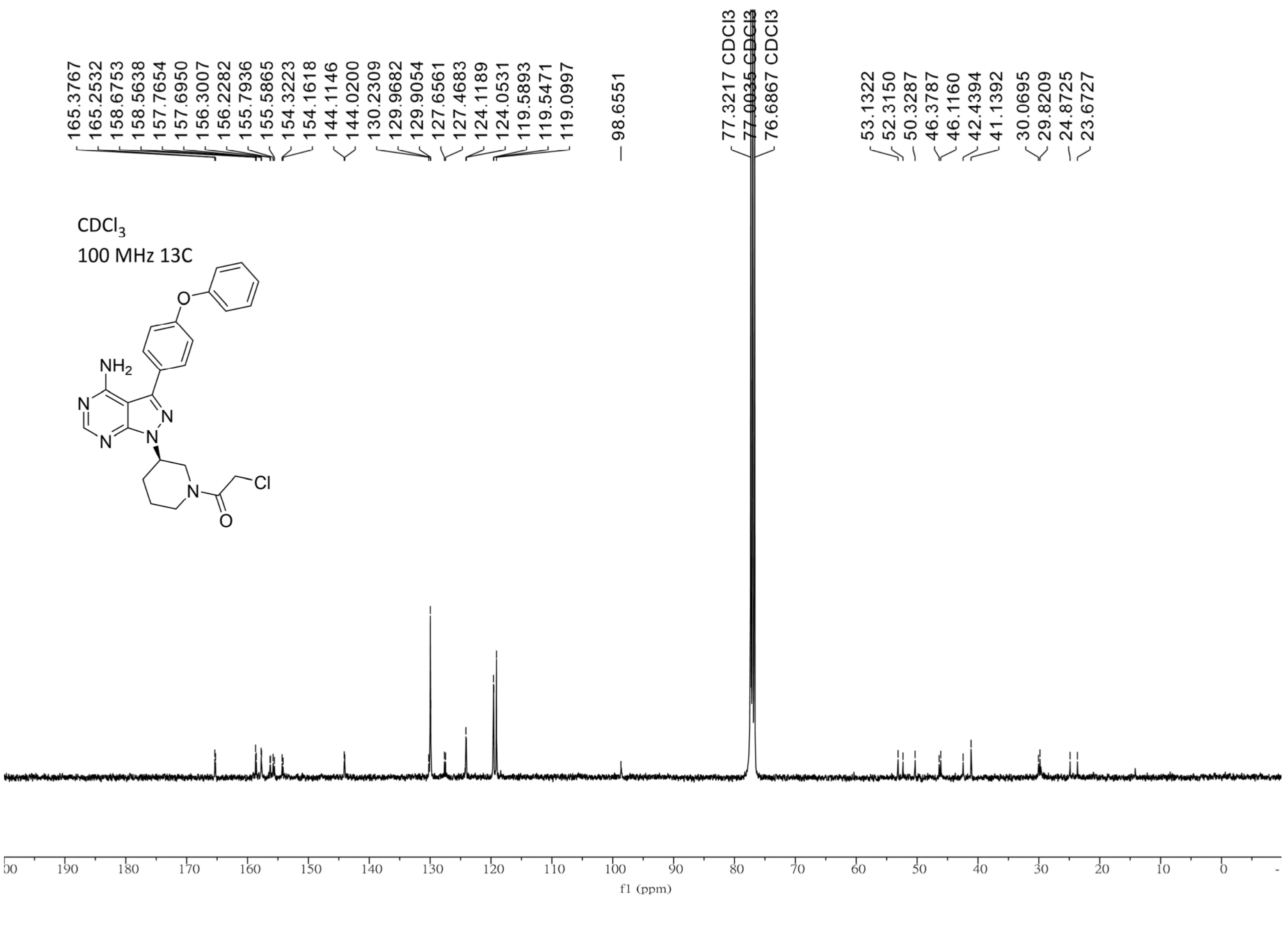
165.3767
165.2532
158.6753
158.5638
157.7654
157.6950
156.3007
156.2282
155.7936
155.5865
154.3223
154.1618
144.1146
144.0200
130.2309
129.9682
129.9054
127.6561
127.4683
124.1189
124.0531
119.5893
119.5471
119.0997
98.6551
77.3217 CDCl3
77.0035 CDCl3
76.6867 CDCl3
53.1322
52.3150
50.3287
46.3787
46.1160
42.4394
41.1392
30.0695
29.8209
24.8725
23.6727
CDCl3
100 MHz 13C
NH2
N
N
N
N
O
N
Cl
O
f1 (ppm)

IbrPPh$_3$ (8)

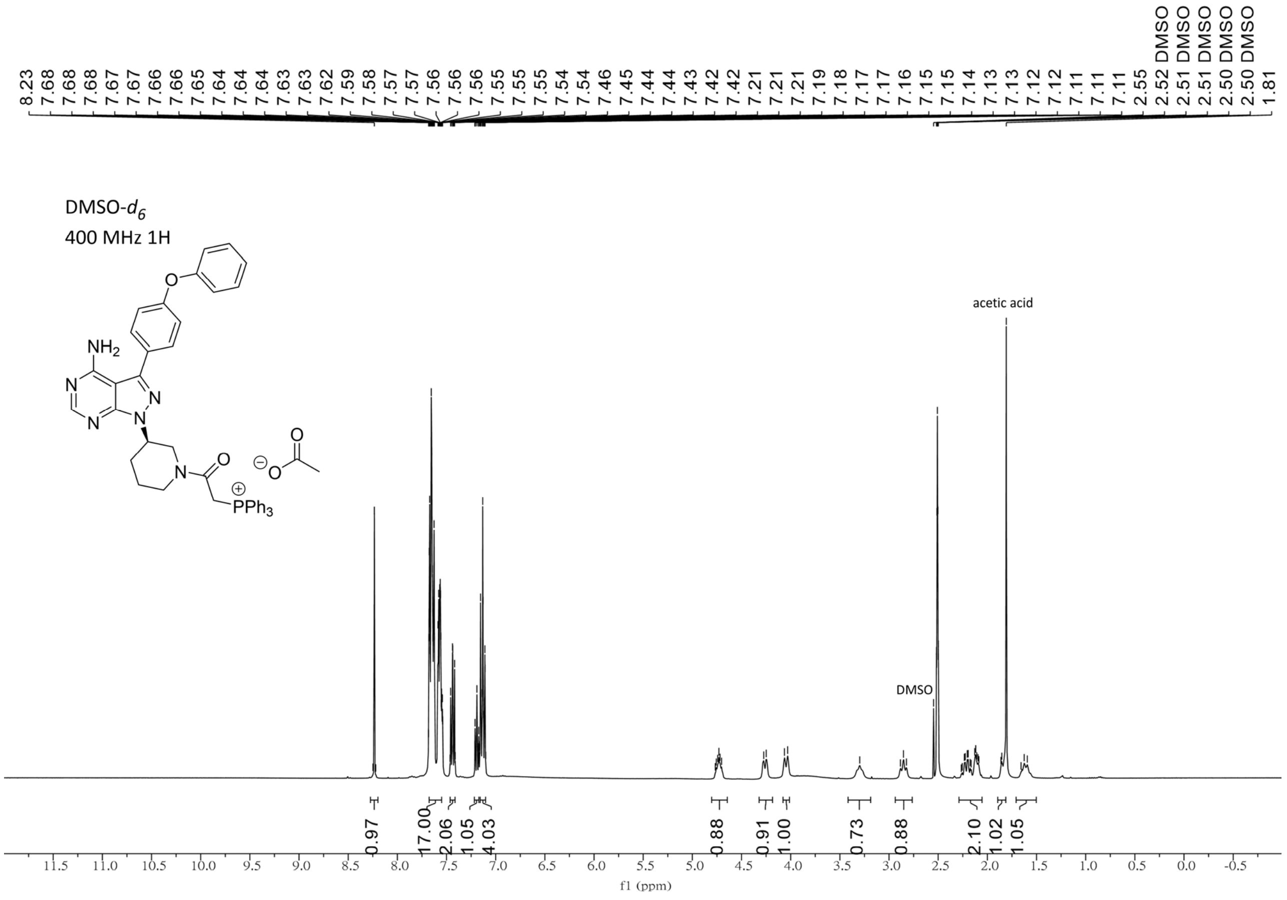
8.23 7.68 7.68 7.68 7.67 7.67 7.66 7.66 7.65 7.64 7.64 7.64 7.63 7.63 7.62 7.59 7.58 7.57 7.57 7.56 7.56 7.56 7.55 7.55 7.55 7.54 7.54 7.46 7.45 7.44 7.44 7.43 7.42 7.42 7.21 7.21 7.21 7.19 7.18 7.17 7.17 7.16 7.15 7.15 7.14 7.13 7.13 7.12 7.12 7.11 7.11 7.11 2.55 2.52 DMSO 2.51 DMSO 2.51 DMSO 2.50 DMSO 2.50 DMSO 1.81
DMSO-d_6
400 MHz 1H
NH$_2$
N
N
N
N
O
N
O
O
PPh$_3$
acetic acid
DMSO
0.97 17.00 2.06 1.05 4.03 0.88 0.91 1.00 0.73 0.88 2.10 1.02 1.05
11.5 11.0 10.5 10.0 9.5 9.0 8.5 8.0 7.5 7.0 6.5 6.0 5.5 5.0 4.5 4.0 3.5 3.0 2.5 2.0 1.5 1.0 0.5 0.0 -0.5
f1 (ppm)

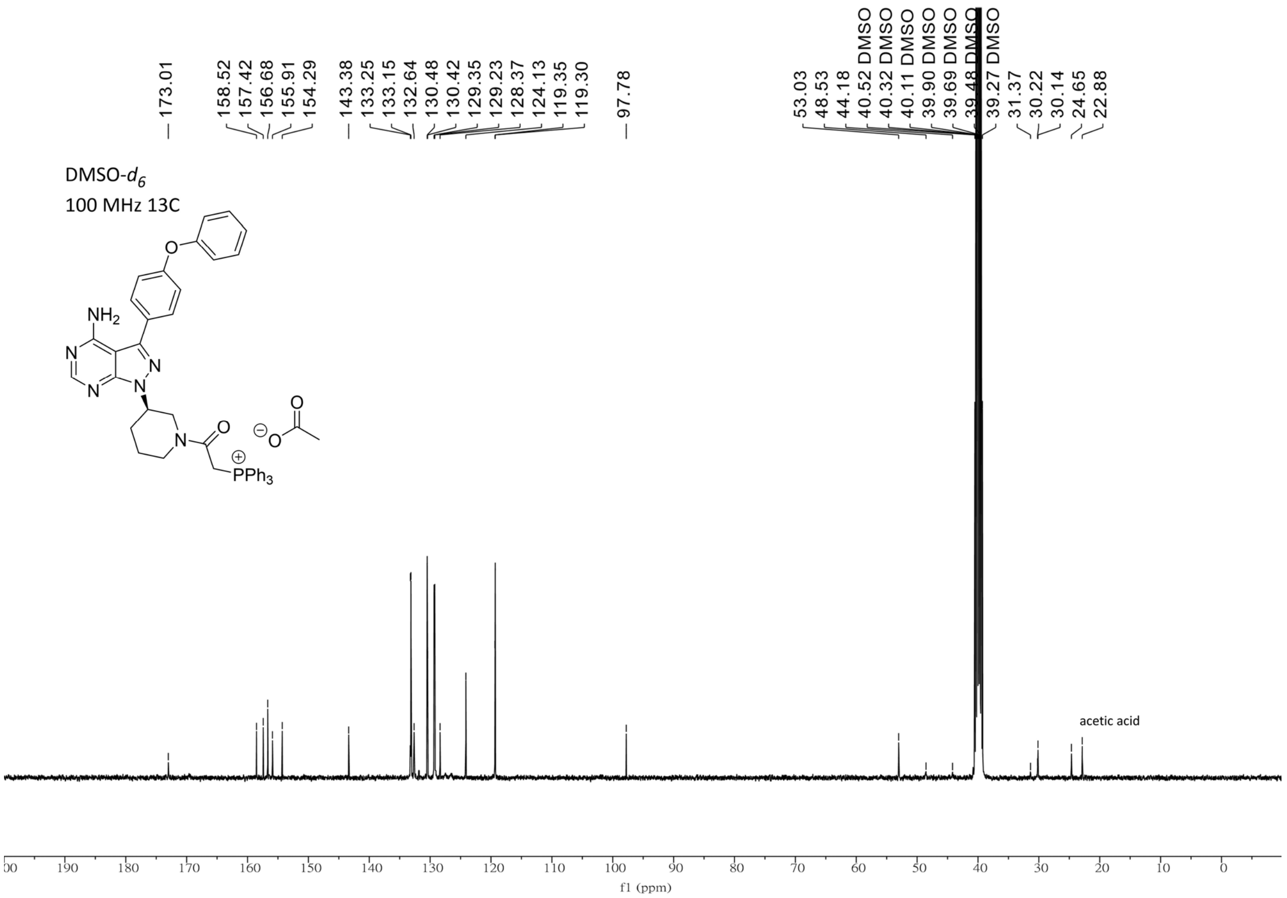
173.01 158.52 157.42 156.68 155.91 154.29 143.38 133.25 133.15 132.64 130.48 130.42 129.35 129.23 128.37 124.13 119.35 119.30 97.78 53.03 48.53 44.18 40.52 DMSO 40.32 DMSO 40.11 DMSO 39.90 DMSO 39.69 DMSO 39.48 DMSO 39.27 DMSO 31.37 30.22 30.14 24.65 22.88
DMSO-d_6
100 MHz 13C
NH$_2$
N
N
N
N
O
N
O
O
PPh$_3$
acetic acid
190 180 170 160 150 140 130 120 110 100 90 80 70 60 50 40 30 20 10 0
f1 (ppm)

DMSO-d_6
162 MHz 31P

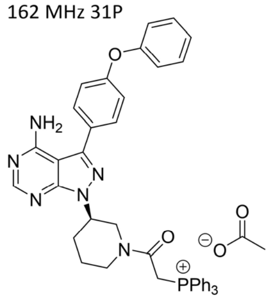

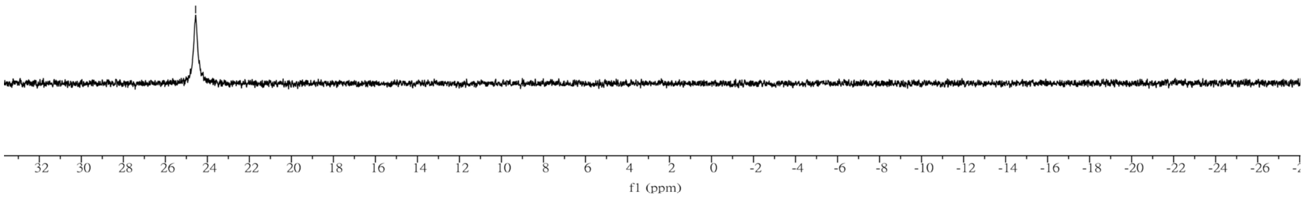

Compound 33

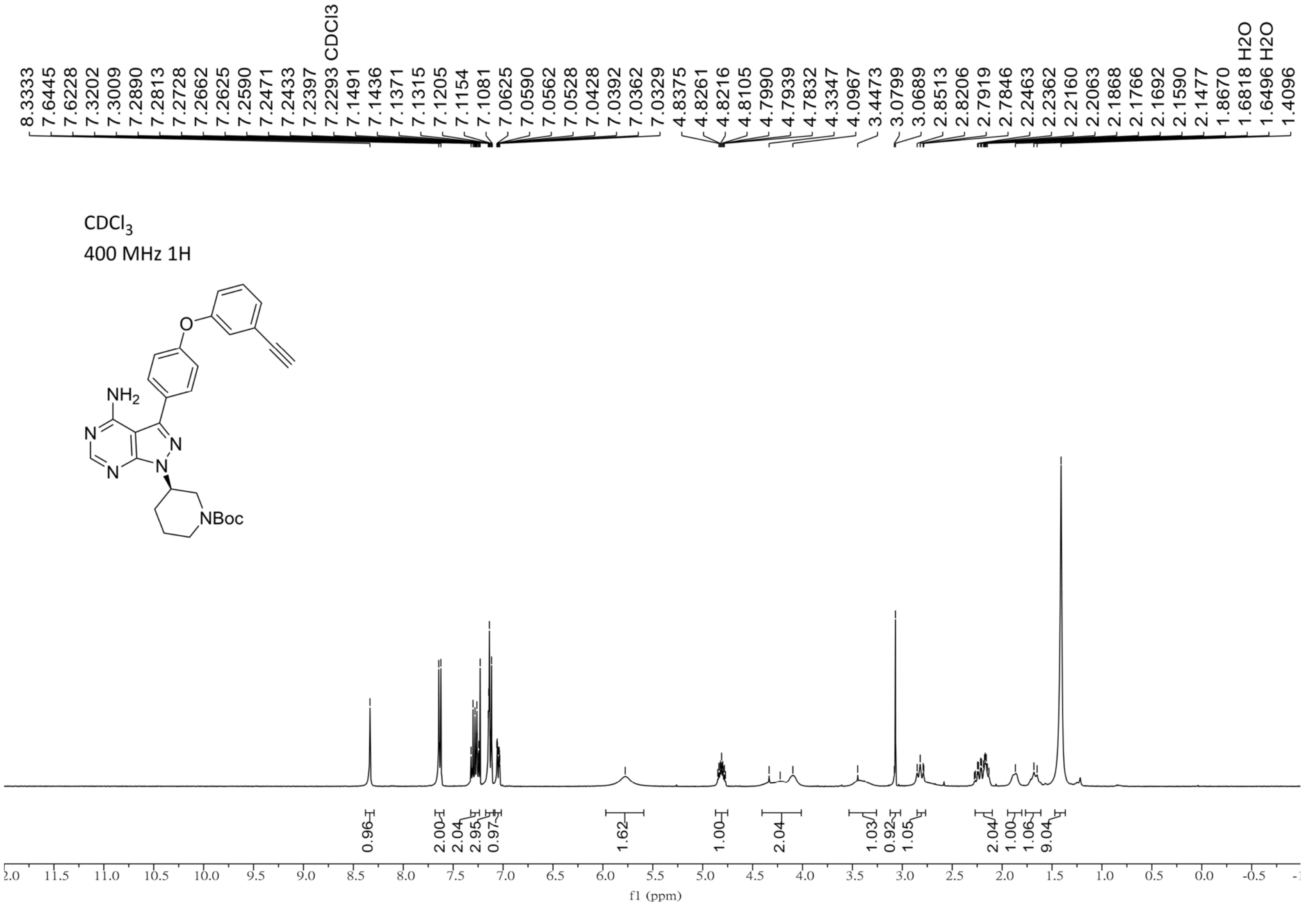
8.3333
7.6445
7.6228
7.3202
7.3009
7.2890
7.2813
7.2728
7.2662
7.2625
7.2590
7.2471
7.2433
7.2397
7.2293 CDCl3
7.1491
7.1436
7.1371
7.1315
7.1205
7.1154
7.1081
7.0625
7.0590
7.0562
7.0528
7.0428
7.0392
7.0362
7.0329
4.8375
4.8261
4.8216
4.8105
4.7990
4.7939
4.7832
4.3347
4.0967
3.4473
3.0799
3.0689
2.8513
2.8206
2.7919
2.7846
2.2463
2.2362
2.2160
2.2063
2.1868
2.1766
2.1692
2.1590
2.1477
1.8670
1.6818 H2O
1.6496 H2O
1.4096
CDCl3
400 MHz 1H
O
NH2
N
N
N
N
NBoc
0.96
2.00
2.04
2.95
0.97
1.62
1.00
2.04
1.03
0.92
1.05
2.04
1.00
1.06
9.04
f1 (ppm)

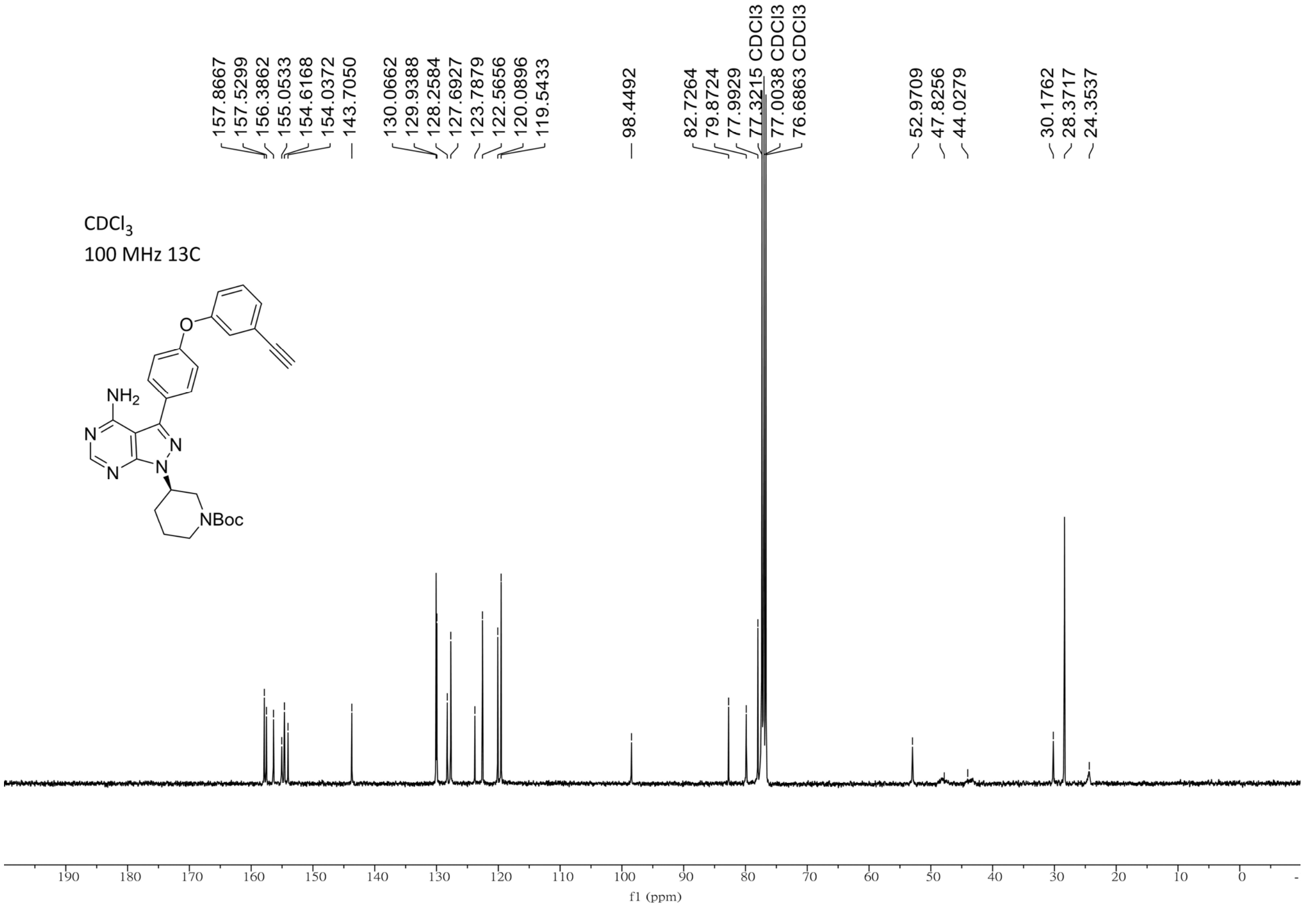
157.8667
157.5299
156.3862
155.0533
154.6168
154.0372
143.7050
130.0662
129.9388
128.2584
127.6927
123.7879
122.5656
120.0896
119.5433
98.4492
82.7264
79.8724
77.9929
77.3215 CDCl3
77.0038 CDCl3
76.6863 CDCl3
52.9709
47.8256
44.0279
30.1762
28.3717
24.3537
CDCl3
100 MHz 13C
O
NH2
N
N
N
N
NBoc
f1 (ppm)

Compound 34

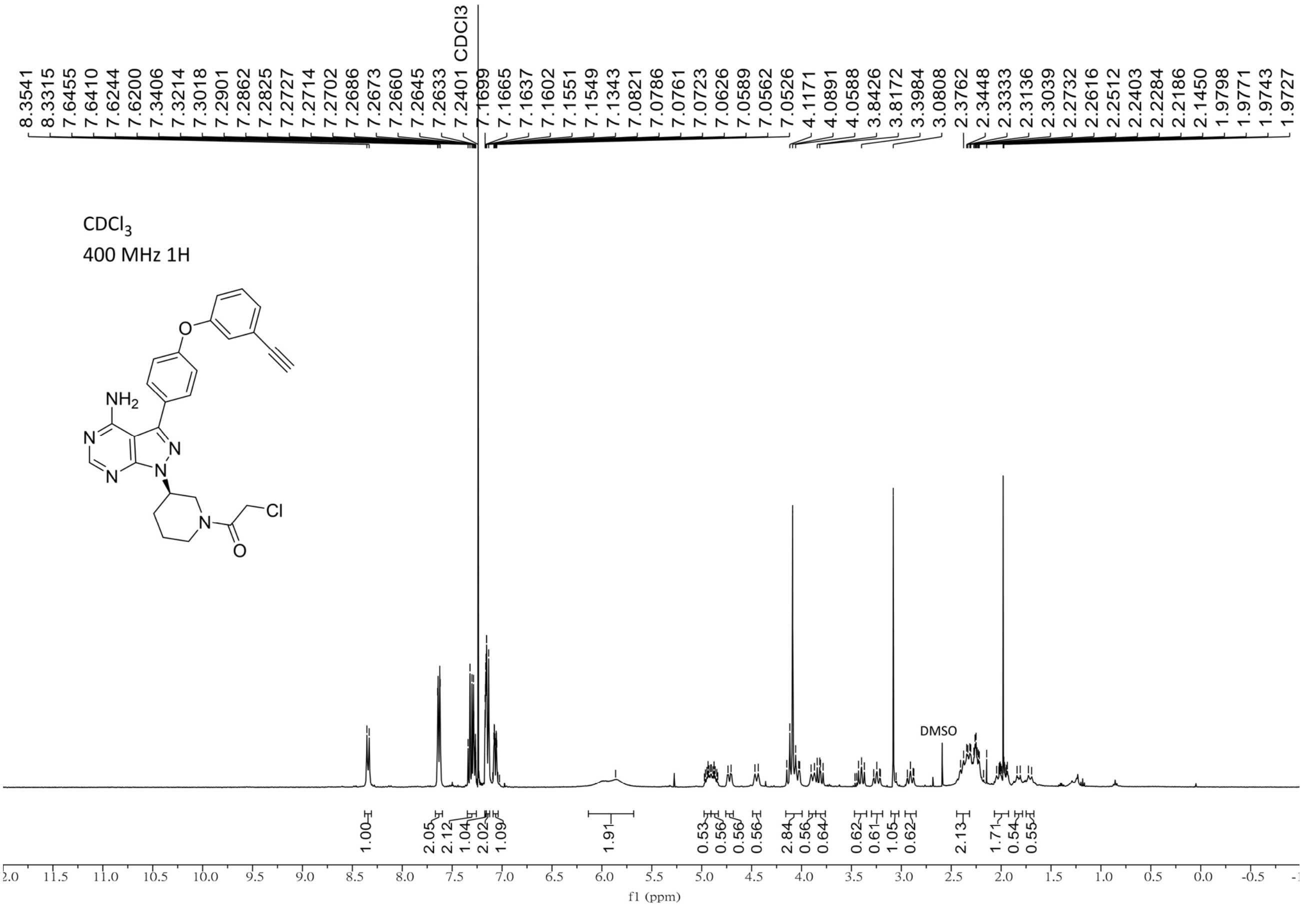
CDCl3
8.3541
8.3315
7.6455
7.6410
7.6244
7.6200
7.3406
7.3214
7.3018
7.2901
7.2862
7.2825
7.2727
7.2714
7.2702
7.2686
7.2673
7.2660
7.2645
7.2633
7.2401 CDCl3
7.1699
7.1665
7.1637
7.1602
7.1551
7.1549
7.1343
7.0821
7.0786
7.0761
7.0723
7.0626
7.0589
7.0562
7.0526
4.1171
4.0891
4.0588
3.8426
3.8172
3.3984
3.0808
2.3762
2.3448
2.3333
2.3136
2.3039
2.2732
2.2616
2.2512
2.2403
2.2284
2.2186
2.1450
1.9798
1.9771
1.9743
1.9727
$CDCl_3$
400 MHz 1H
NH_2
N
N
N
N
O
Cl
O
DMSO
1.00
2.05
2.12
1.04
2.02
1.09
1.91
0.53
0.56
0.56
0.56
2.84
0.56
0.64
0.62
0.61
1.05
0.62
2.13
1.71
0.54
0.55
f1 (ppm)

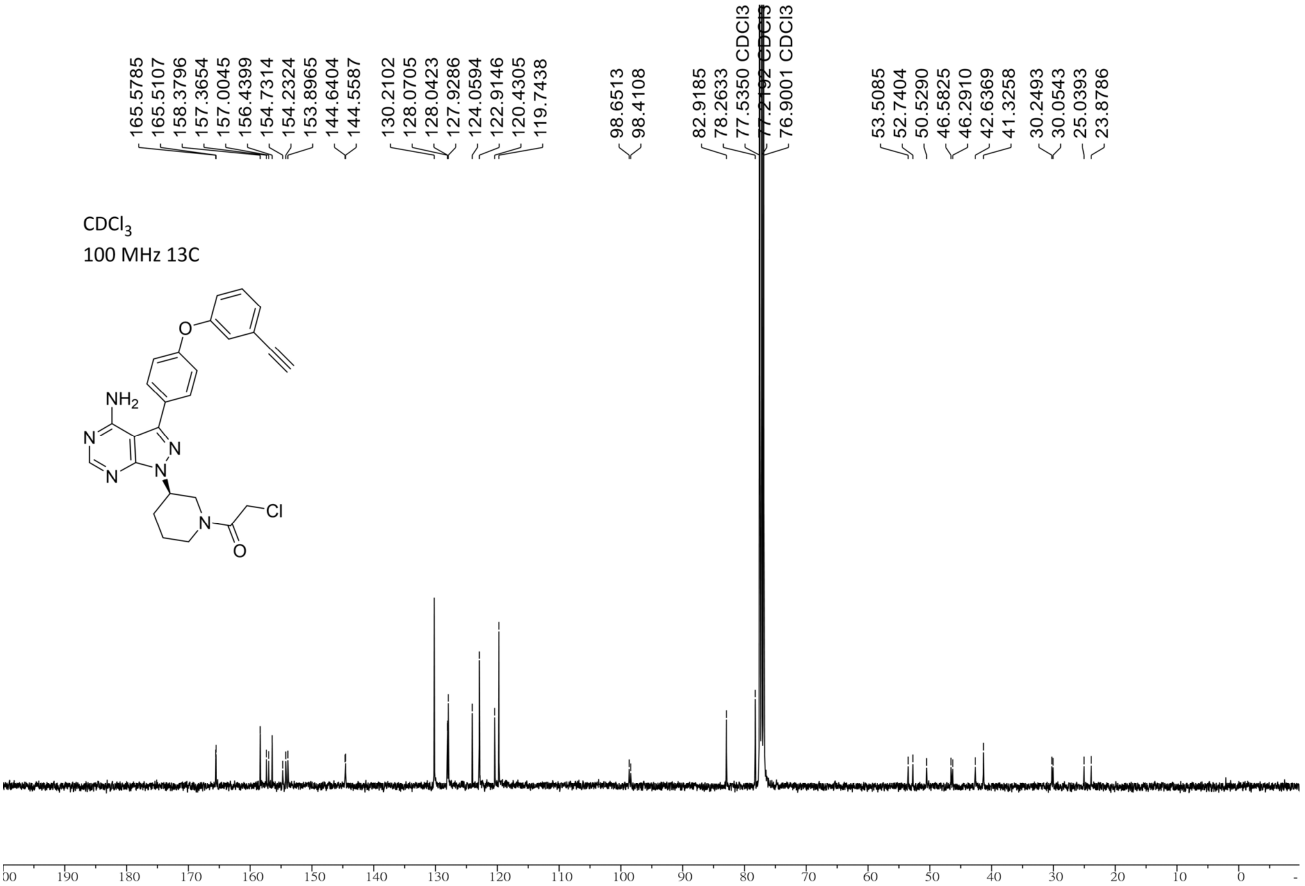
165.5785
165.5107
158.3796
157.3654
157.0045
156.4399
154.7314
154.2324
153.8965
144.6404
144.5587
130.2102
128.0705
128.0423
127.9286
124.0594
122.9146
120.4305
119.7438
98.6513
98.4108
82.9185
78.2633
77.5350 CDCl3
77.2192 CDCl3
76.9001 CDCl3
53.5085
52.7404
50.5290
46.5825
46.2910
42.6369
41.3258
30.2493
30.0543
25.0393
23.8786
$CDCl_3$
100 MHz 13C
NH_2
N
N
N
N
O
Cl
O
f1 (ppm)

IbrPPh$_3$-yne (10)

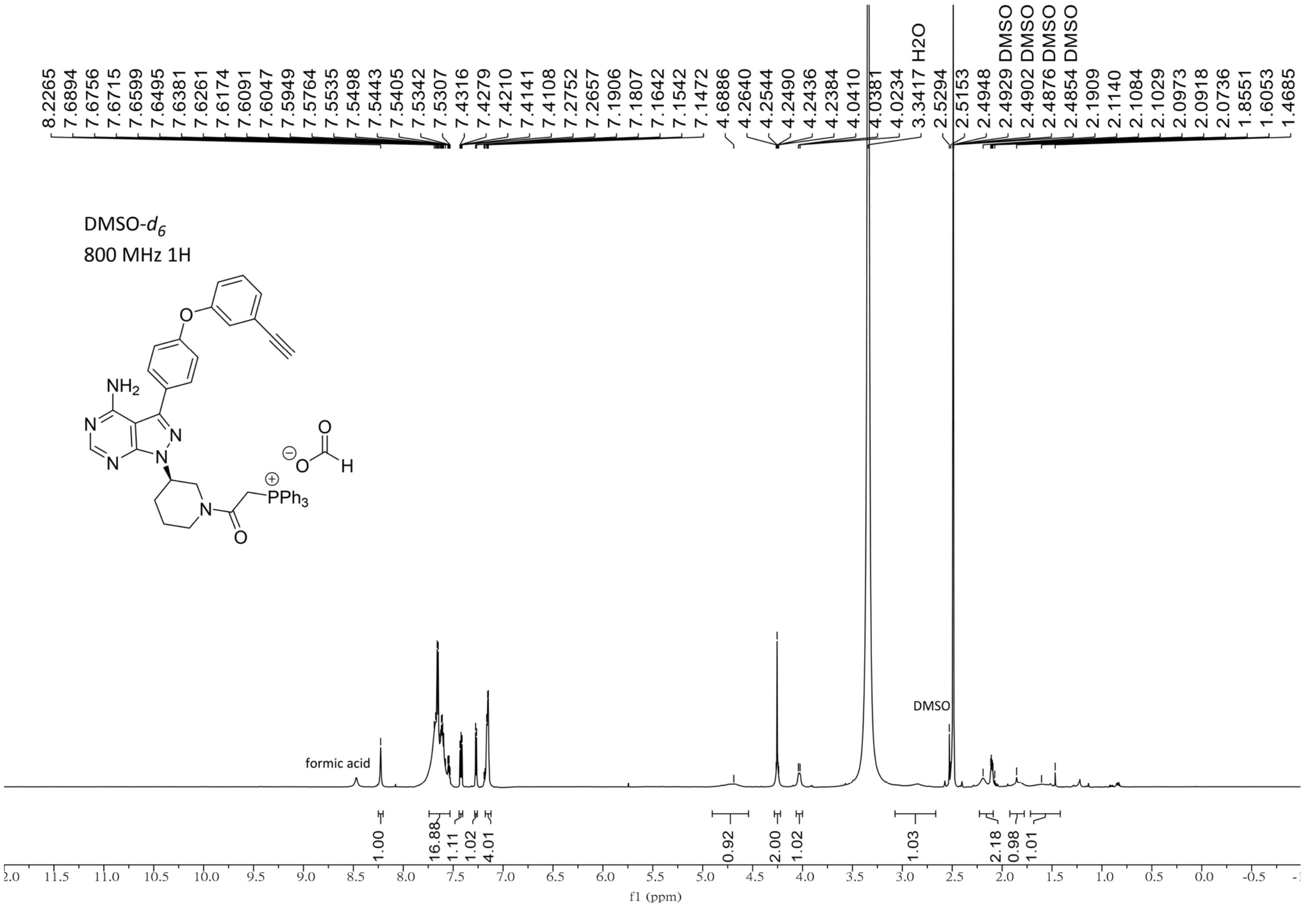

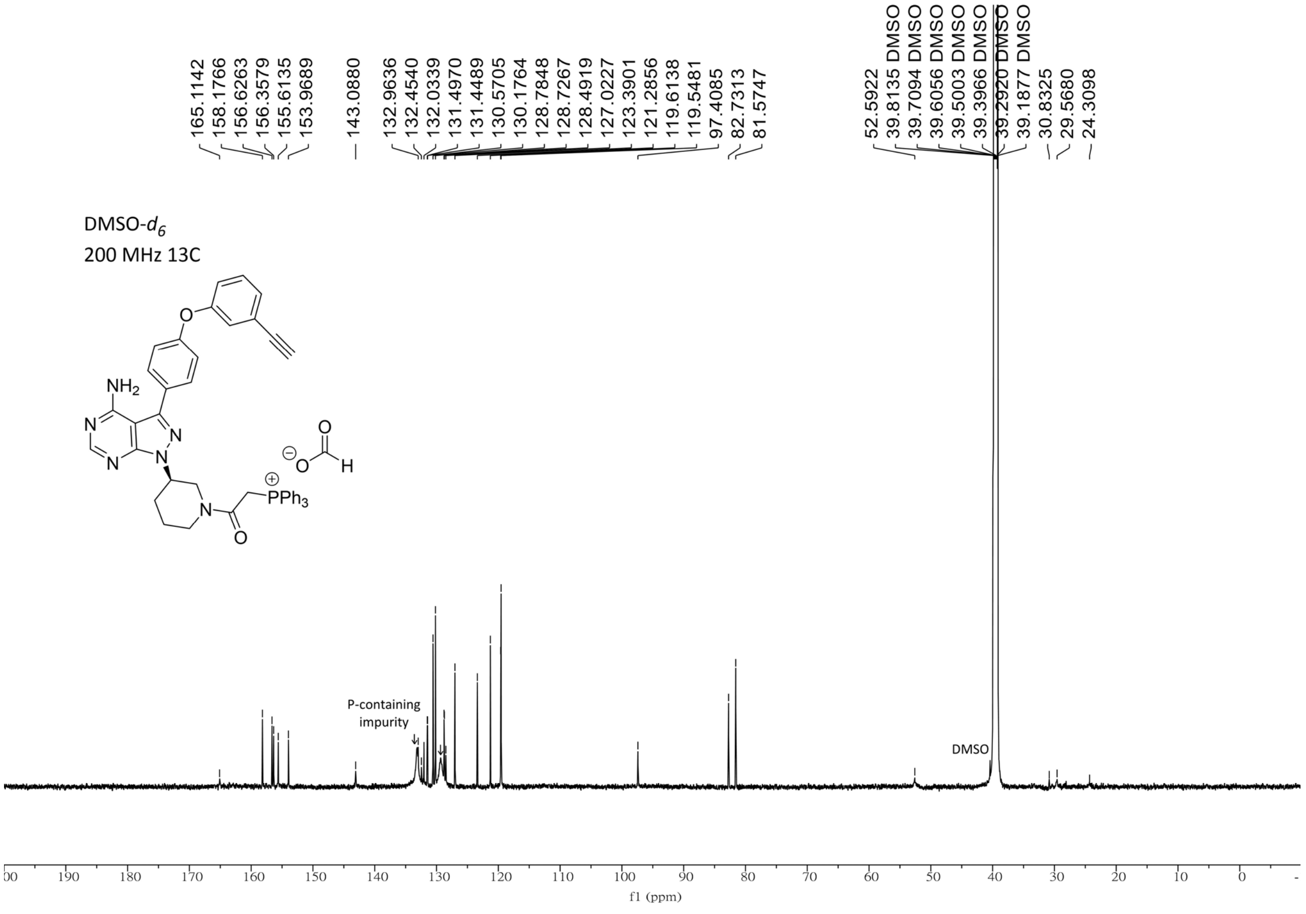

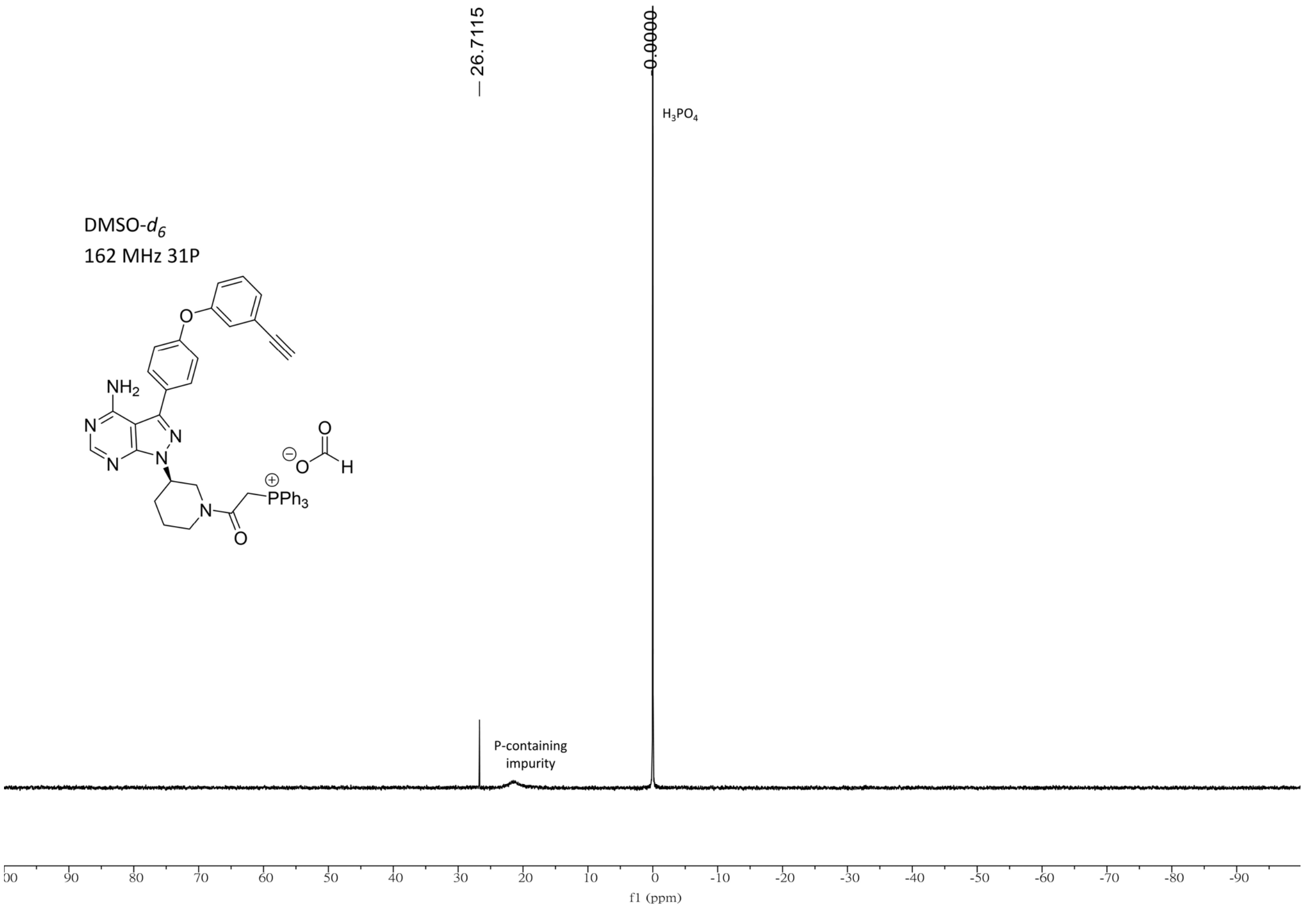

26.7115
0.0000
H3PO4
DMSO-d6
162 MHz 31P
P-containing impurity
f1 (ppm)

Compound 36

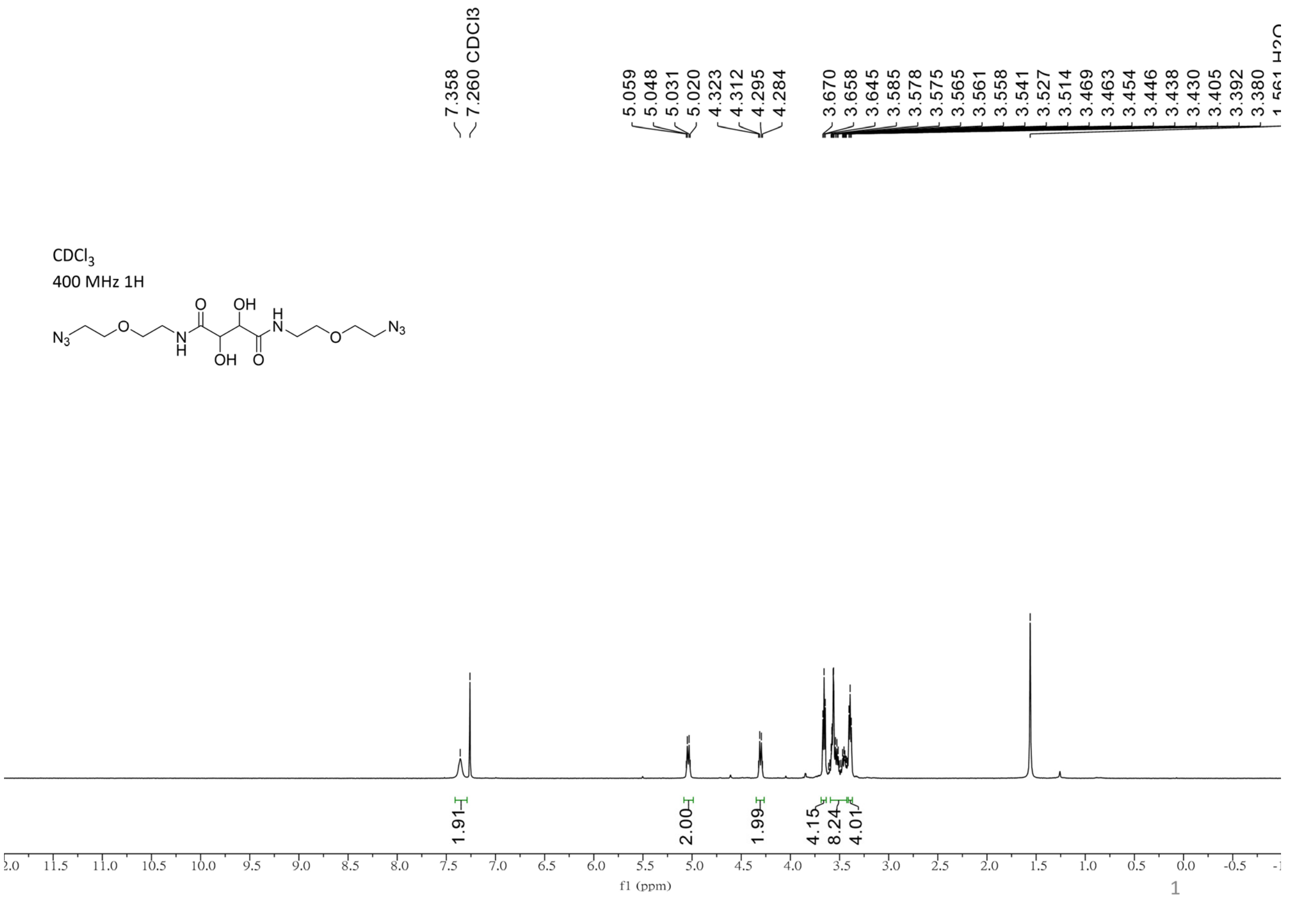

7.358
7.260 CDCl3
5.059
5.048
5.031
5.020
4.323
4.312
4.295
4.284
3.670
3.658
3.645
3.585
3.578
3.575
3.565
3.561
3.558
3.541
3.527
3.514
3.469
3.463
3.454
3.446
3.438
3.430
3.405
3.392
3.380
$CDCl_3$
400 MHz 1H
N_3
O
OH
H
N
N
H
OH
O
N_3
1.91
2.00
1.99
4.15
8.24
4.01
f1 (ppm)

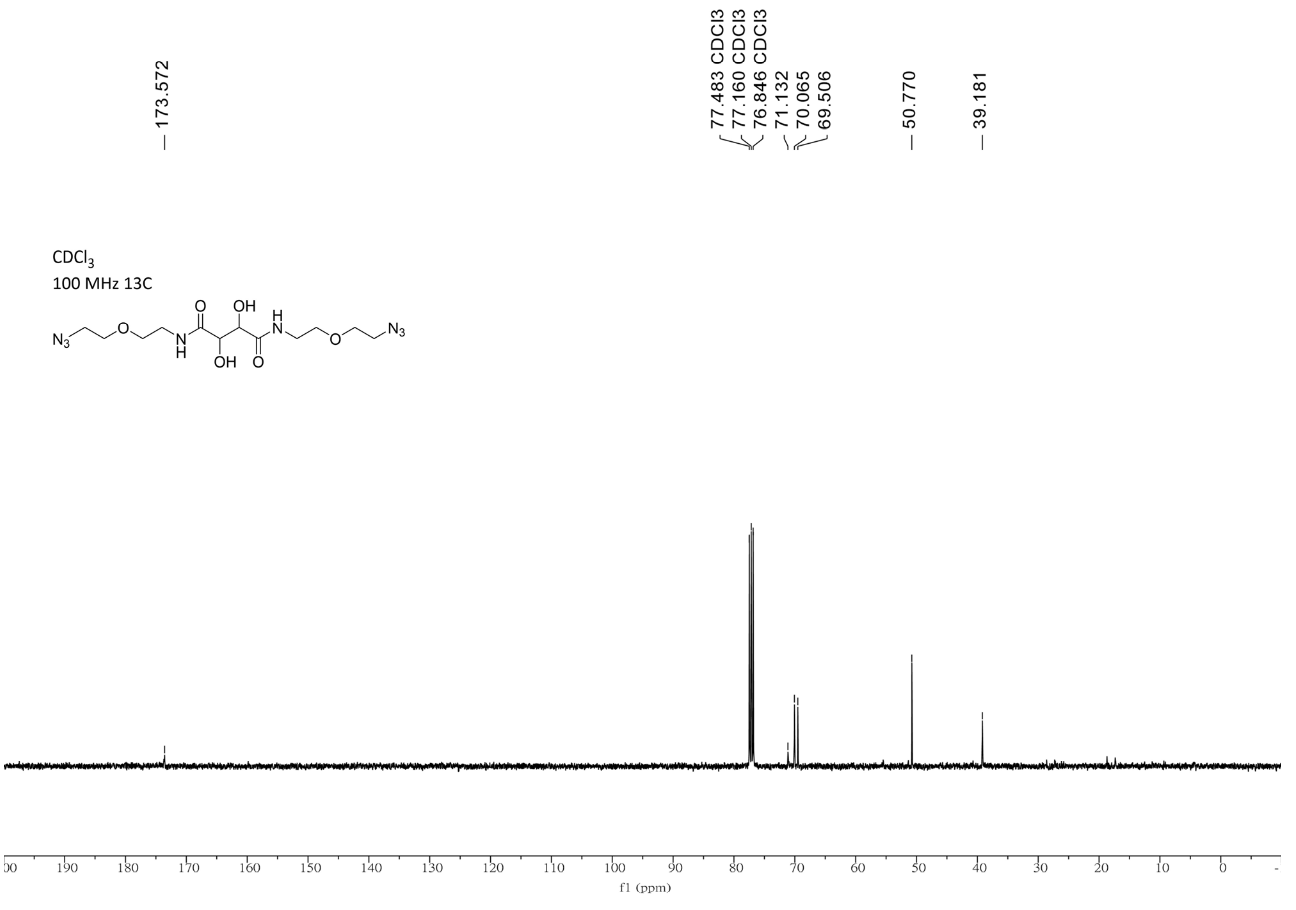

173.572
77.483 CDCl3
77.160 CDCl3
76.846 CDCl3
71.132
70.065
69.506
50.770
39.181
$CDCl_3$
100 MHz 13C
N_3
O
OH
H
N
N
H
OH
O
N_3
f1 (ppm)

AldN$_3$ (16)

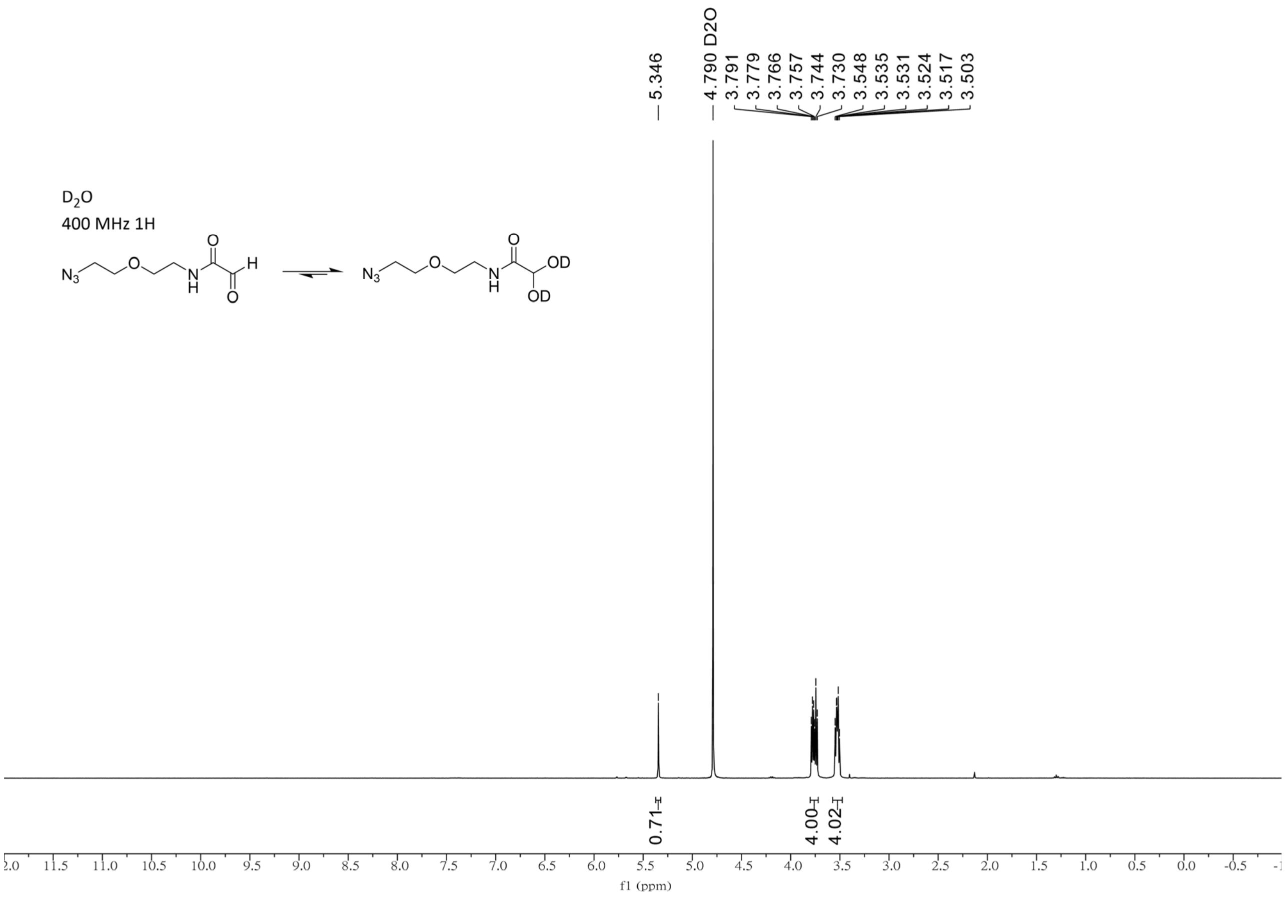
5.346
4.790 D2O
3.791
3.779
3.766
3.757
3.744
3.730
3.548
3.535
3.531
3.524
3.517
3.503
D2O
400 MHz 1H
0.71
4.00
4.02
f1 (ppm)

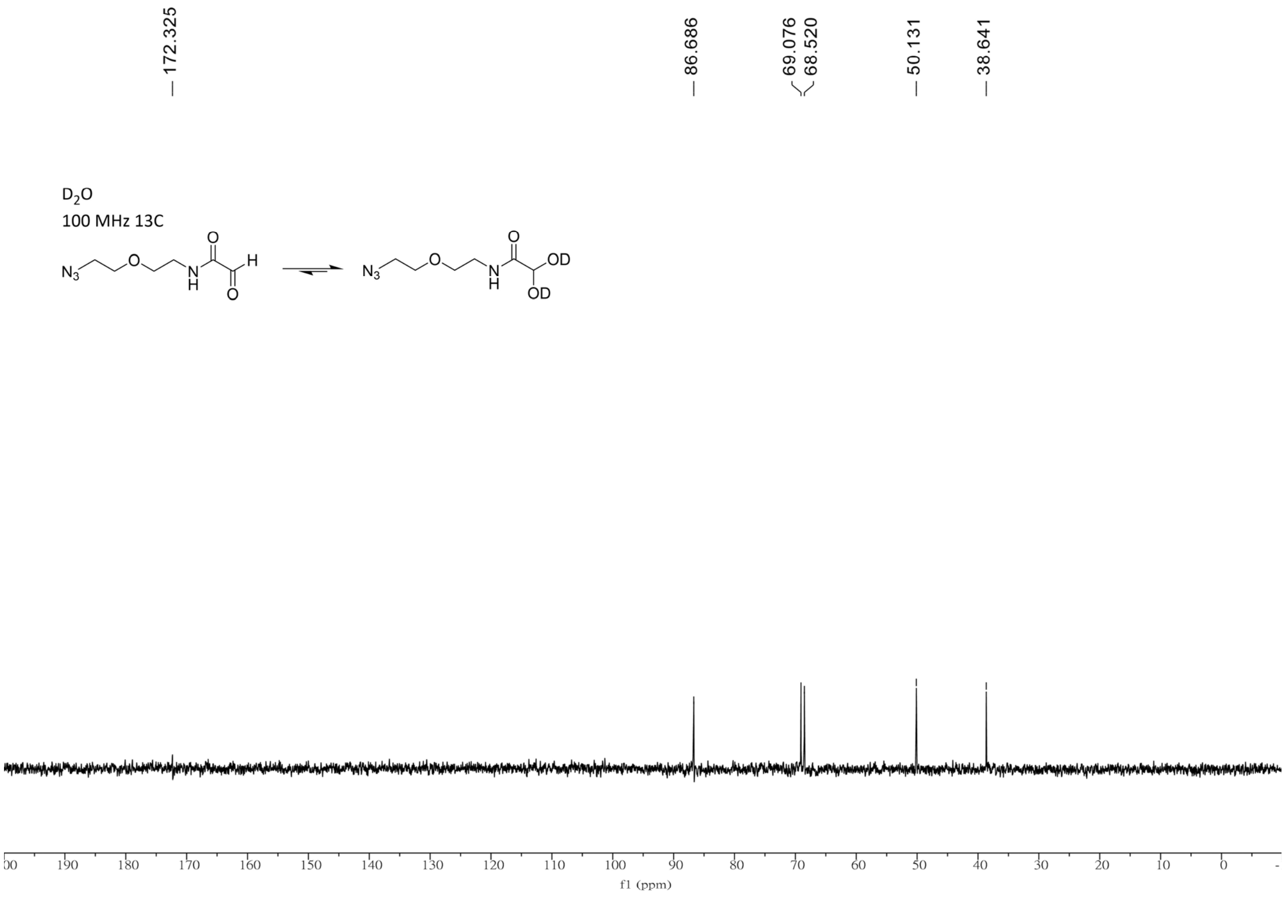
172.325
86.686
69.076
68.520
50.131
38.641
D2O
100 MHz 13C
f1 (ppm)

Compound 40

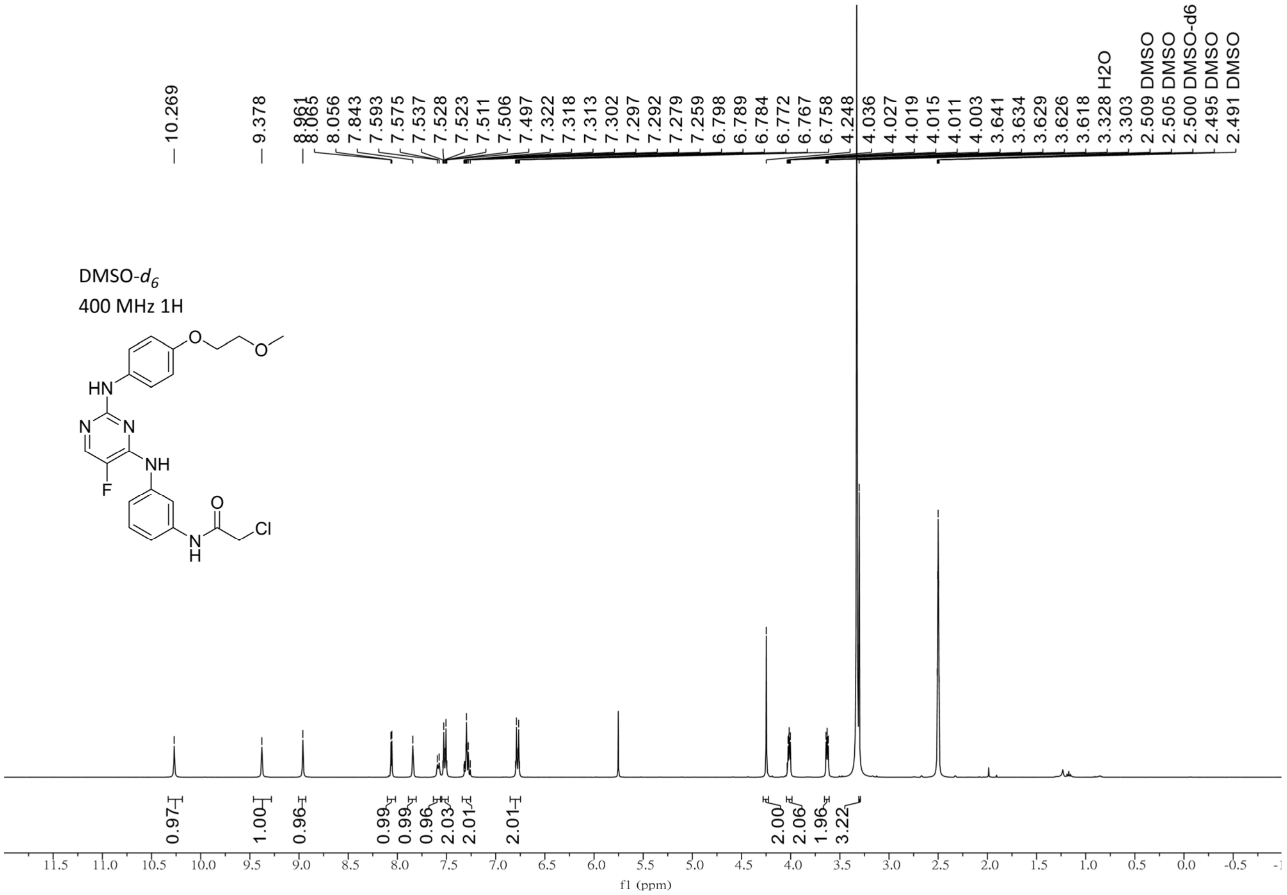
DMSO-d_6
400 MHz 1H
O
O
HN
N
N
NH
F
O
N
H
Cl
10.269
9.378
8.961
8.065
8.056
7.843
7.593
7.575
7.537
7.528
7.523
7.511
7.506
7.497
7.322
7.318
7.313
7.302
7.297
7.292
7.279
7.259
6.798
6.789
6.784
6.772
6.767
6.758
4.248
4.036
4.027
4.019
4.015
4.011
4.003
3.641
3.634
3.629
3.626
3.618
3.328 H2O
3.303
2.509 DMSO
2.505 DMSO
2.500 DMSO-d6
2.495 DMSO
2.491 DMSO
0.97
1.00
0.96
0.99
0.99
0.96
2.03
2.01
2.01
2.00
2.06
1.96
3.22
f1 (ppm)

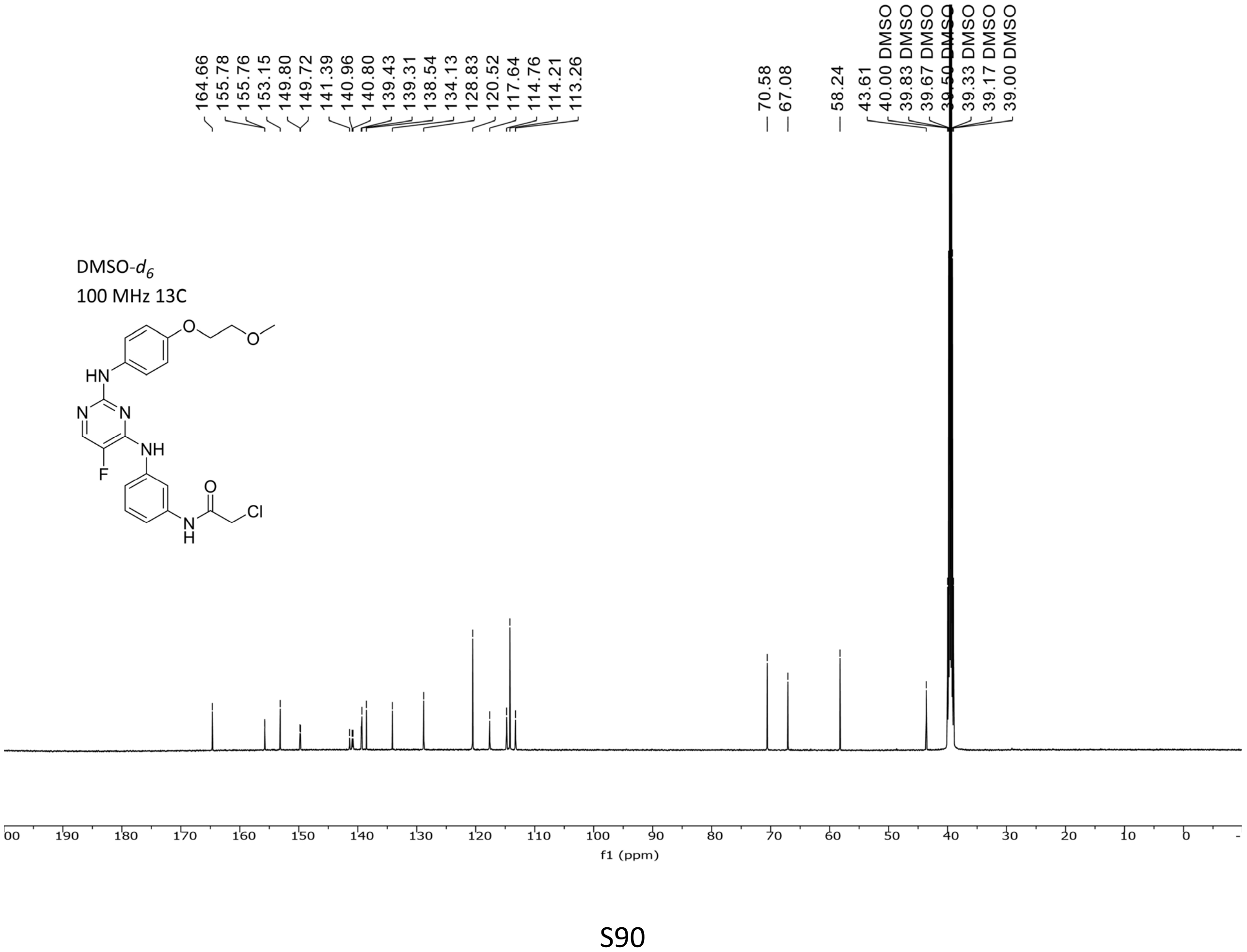
DMSO-d_6
100 MHz 13C
O
O
HN
N
N
NH
F
O
N
H
Cl
164.66
155.78
155.76
153.15
149.80
149.72
141.39
140.96
140.80
139.43
139.31
138.54
134.13
128.83
120.52
117.64
114.76
114.21
113.26
70.58
67.08
58.24
43.61
40.00 DMSO
39.83 DMSO
39.67 DMSO
39.50 DMSO
39.33 DMSO
39.17 DMSO
39.00 DMSO
f1 (ppm)

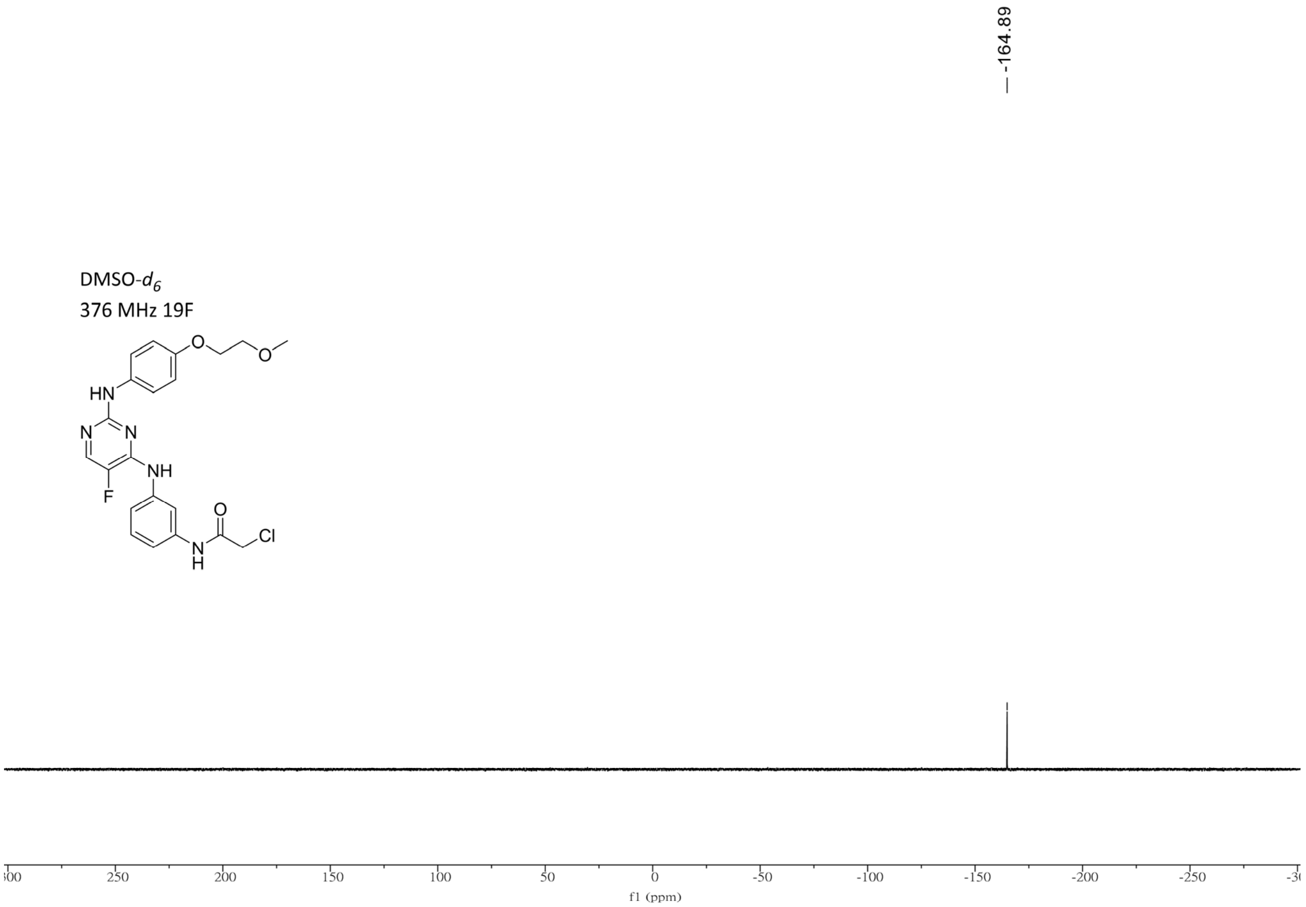
-164.89
DMSO-d_6
376 MHz 19F
HN
N
N
NH
F
O
O
O
N
H
Cl
f1 (ppm)

SpePPh$_3$ (9)

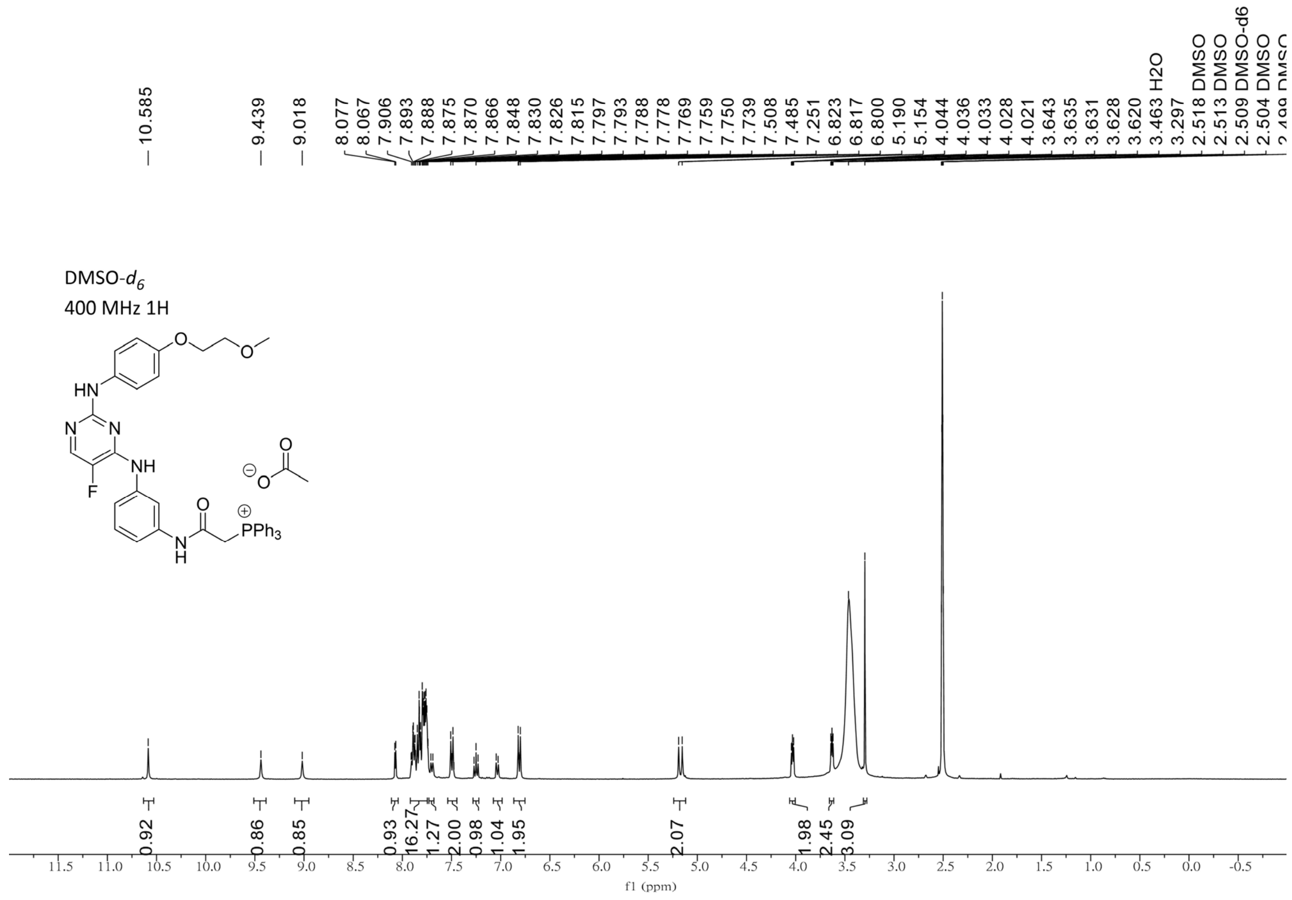

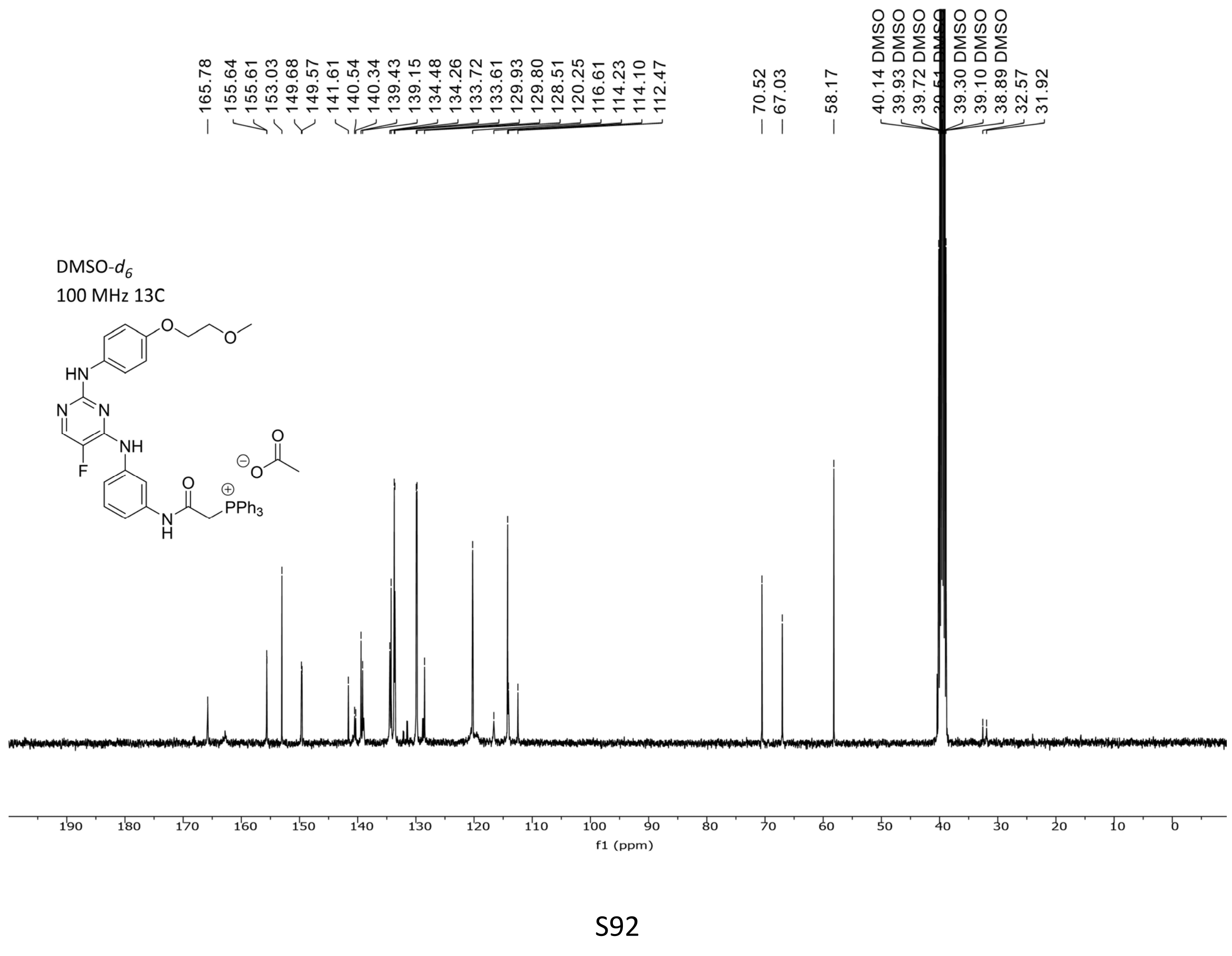

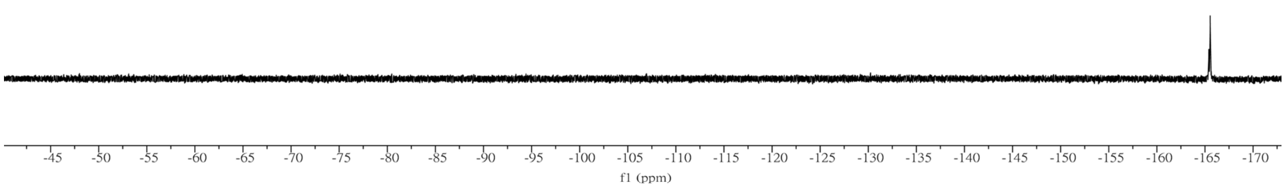

DMSO-d_6
376 MHz 19F

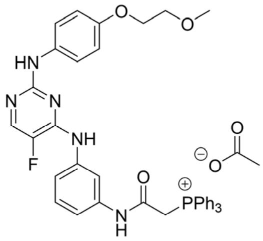

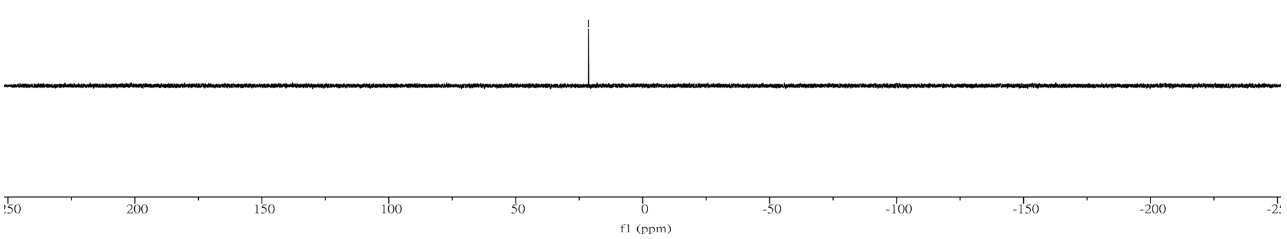

DMSO-d_6
162 MHz 31P

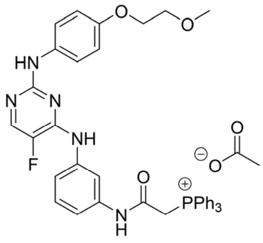

Compound 42

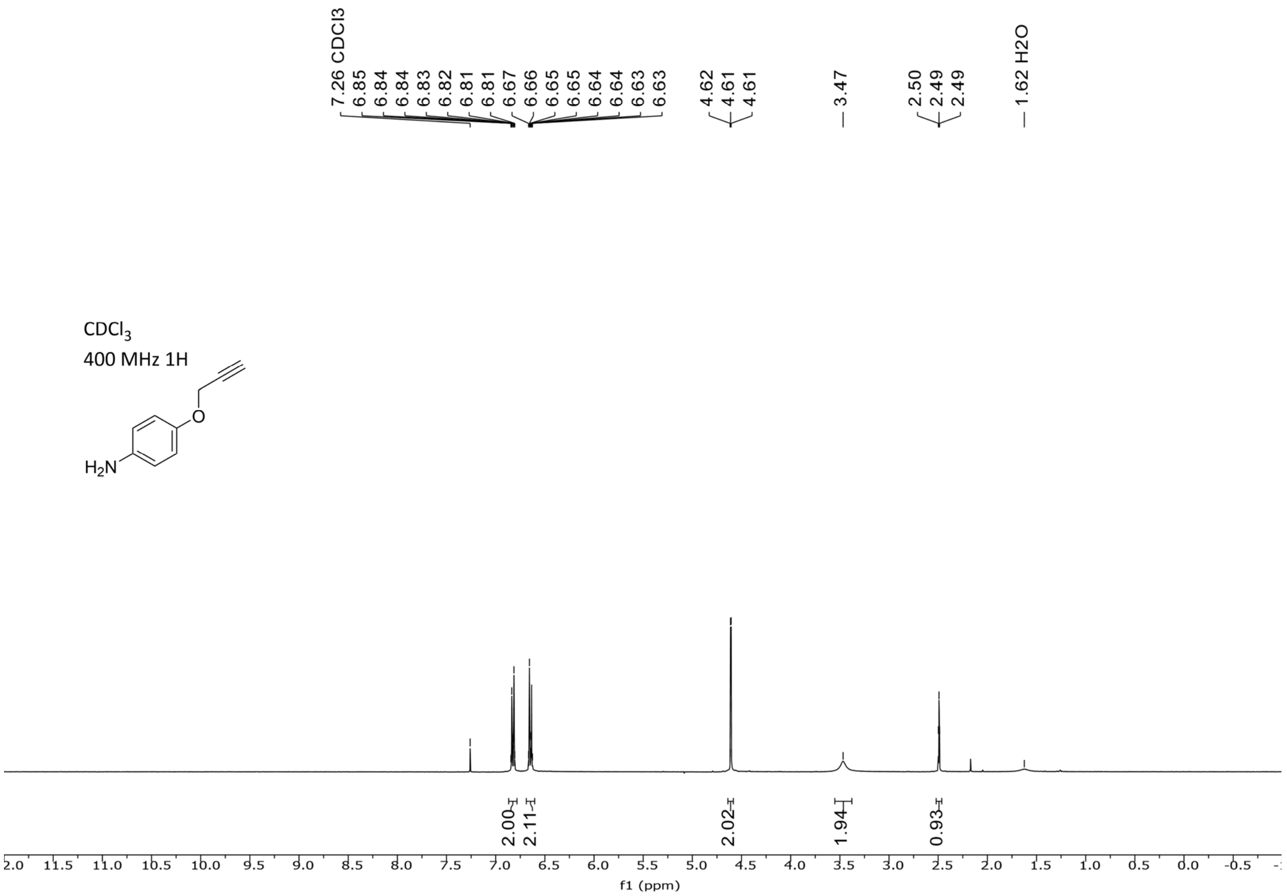
7.26 CDCl3
6.85
6.84
6.84
6.83
6.82
6.81
6.81
6.67
6.66
6.65
6.65
6.64
6.64
6.63
6.63
4.62
4.61
4.61
3.47
2.50
2.49
2.49
1.62 H2O
CDCl3
400 MHz 1H
O
H2N
2.00
2.11
2.02
1.94
0.93
f1 (ppm)

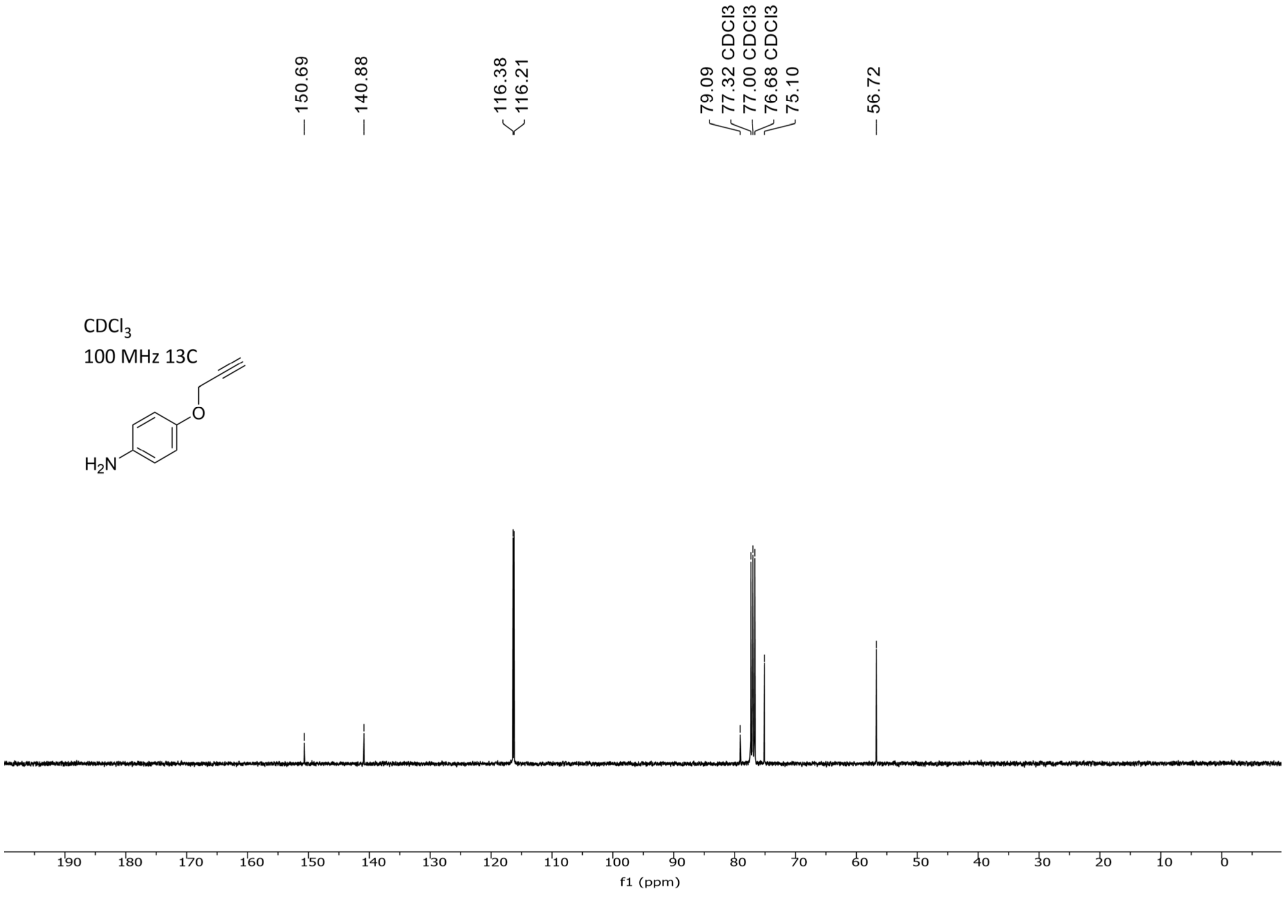
150.69
140.88
116.38
116.21
79.09
77.32 CDCl3
77.00 CDCl3
76.68 CDCl3
75.10
56.72
CDCl3
100 MHz 13C
O
H2N
f1 (ppm)

Compound 43

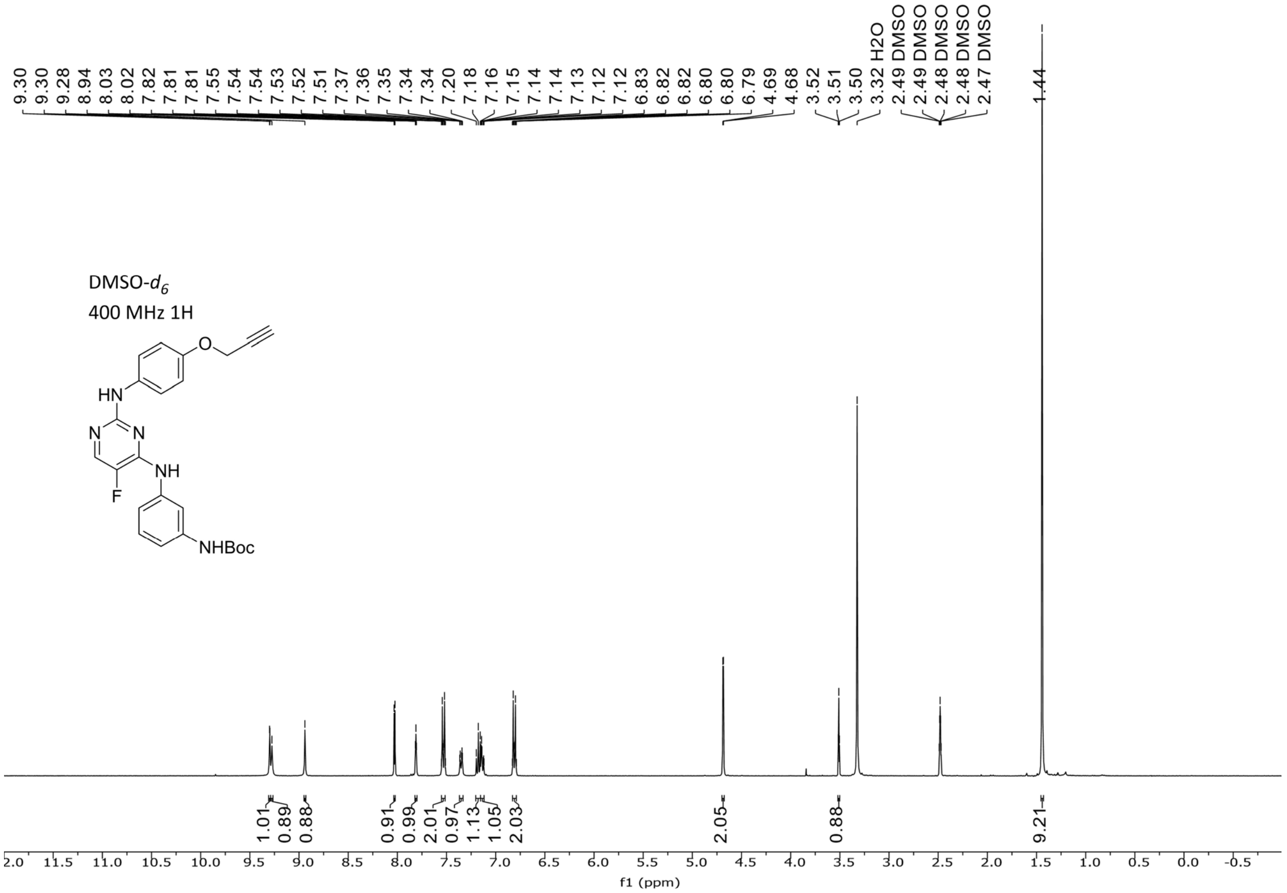
9.30
9.30
9.28
8.94
8.03
8.02
7.82
7.81
7.81
7.55
7.54
7.54
7.53
7.52
7.51
7.37
7.36
7.35
7.34
7.34
7.20
7.18
7.16
7.15
7.14
7.13
7.12
7.12
6.83
6.82
6.82
6.80
6.80
6.79
4.69
4.68
3.52
3.51
3.50
3.32 H2O
2.49 DMSO
2.49 DMSO
2.48 DMSO
2.48 DMSO
2.47 DMSO
1.44
DMSO-d_6
400 MHz 1H
HN
N
N
NH
F
NHBoc
O
1.01
0.89
0.88
0.91
0.99
2.01
0.97
1.13
1.05
2.03
2.05
0.88
9.21
f1 (ppm)

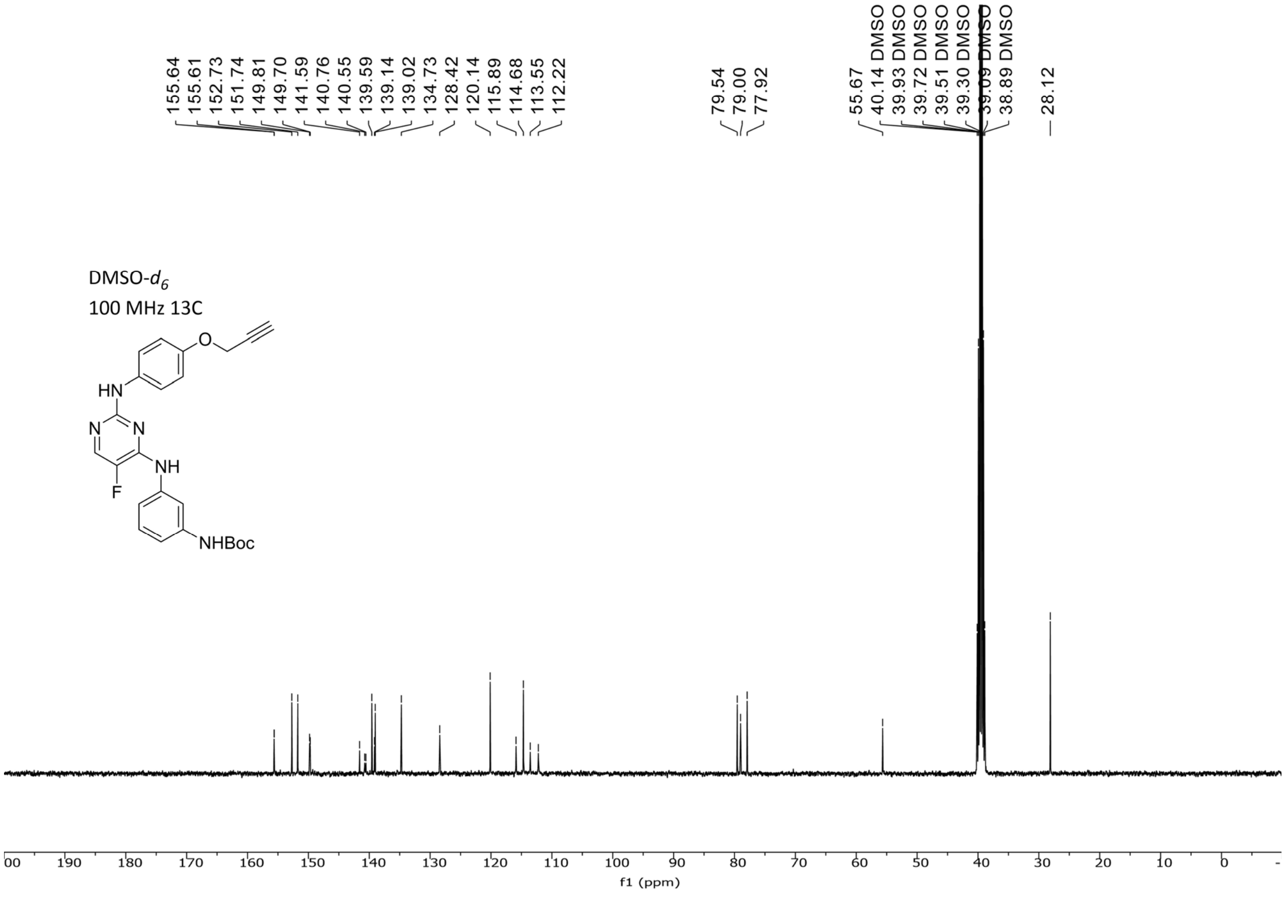
155.64
155.61
152.73
151.74
149.81
149.70
141.59
140.76
140.55
139.59
139.14
139.02
134.73
128.42
120.14
115.89
114.68
113.55
112.22
79.54
79.00
77.92
55.67
40.14 DMSO
39.93 DMSO
39.72 DMSO
39.51 DMSO
39.30 DMSO
39.09 DMSO
38.89 DMSO
28.12
DMSO-d_6
100 MHz 13C
HN
N
N
NH
F
NHBoc
O
f1 (ppm)

DMSO-d_6
376 MHz 19F

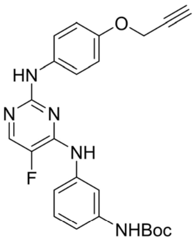

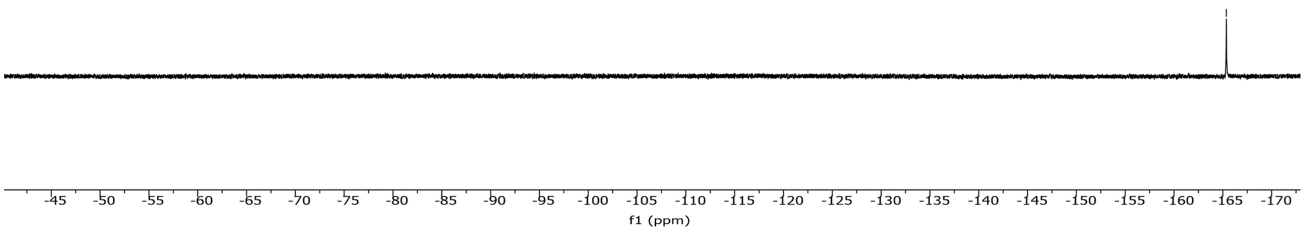

Compound 44

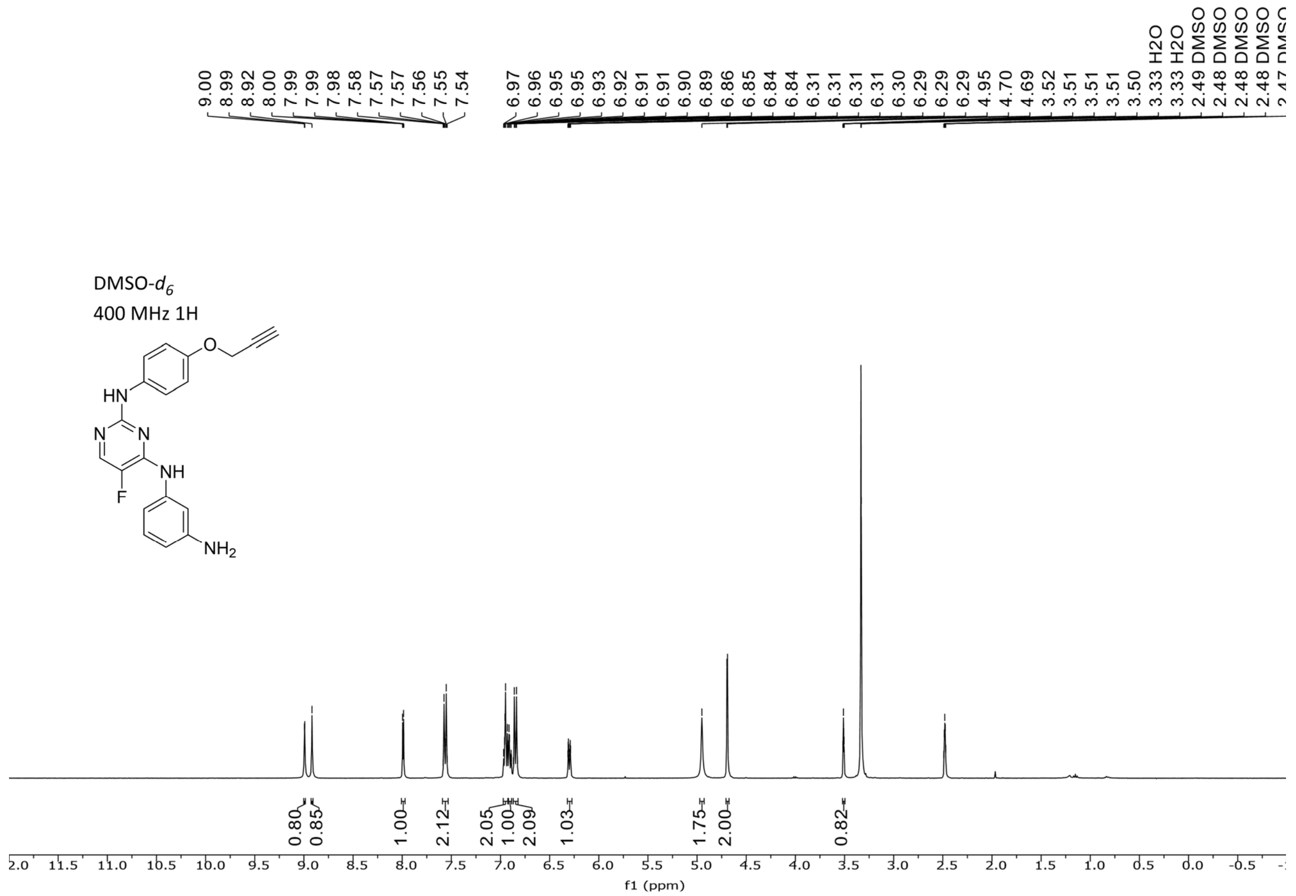

9.00 8.99 8.92 8.00 7.99 7.99 7.98 7.58 7.57 7.57 7.56 7.55 7.54
6.97 6.96 6.95 6.95 6.93 6.92 6.91 6.91 6.90 6.89 6.86 6.85 6.84 6.84 6.31 6.31 6.31 6.30 6.29 6.29 6.29 4.95 4.70 4.69 3.52 3.51 3.51 3.51 3.50
3.33 H2O
3.33 H2O
2.49 DMSO
2.48 DMSO
2.48 DMSO
2.48 DMSO
DMSO-d_6
400 MHz 1H
HN
N
N
NH
F
O
NH_2
0.80 0.85 1.00 2.12 2.05 1.00 2.09 1.03 1.75 2.00 0.82
f1 (ppm)

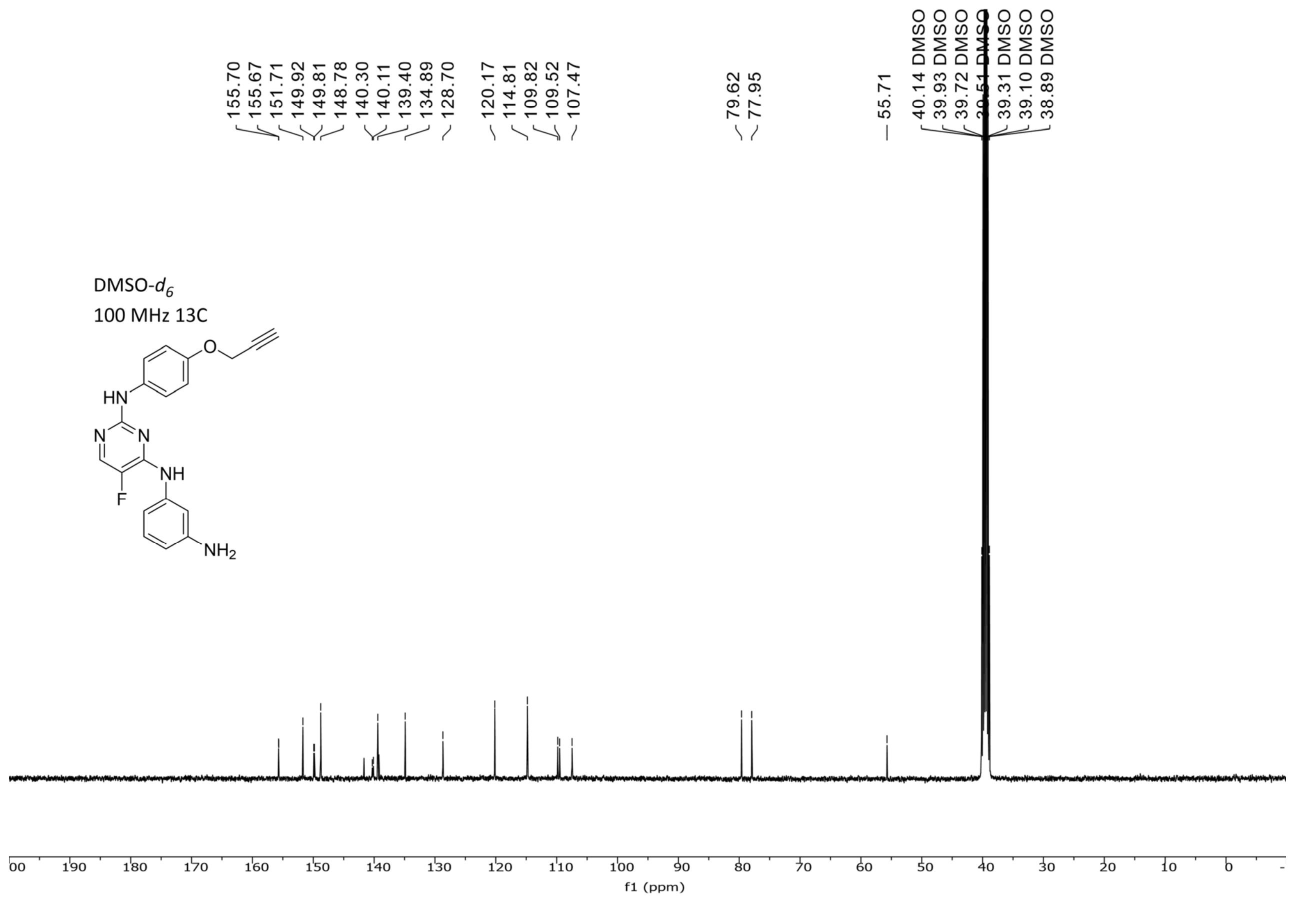

155.70 155.67 151.71 149.92 149.81 148.78 140.30 140.11 139.40 134.89 128.70
120.17 114.81 109.82 109.52 107.47
79.62 77.95
55.71
40.14 DMSO
39.93 DMSO
39.72 DMSO
39.31 DMSO
39.10 DMSO
38.89 DMSO
DMSO-d_6
100 MHz 13C
HN
N
N
NH
F
O
NH_2
f1 (ppm)

DMSO-d_6
376 MHz 19F

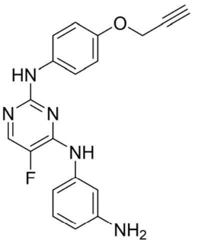

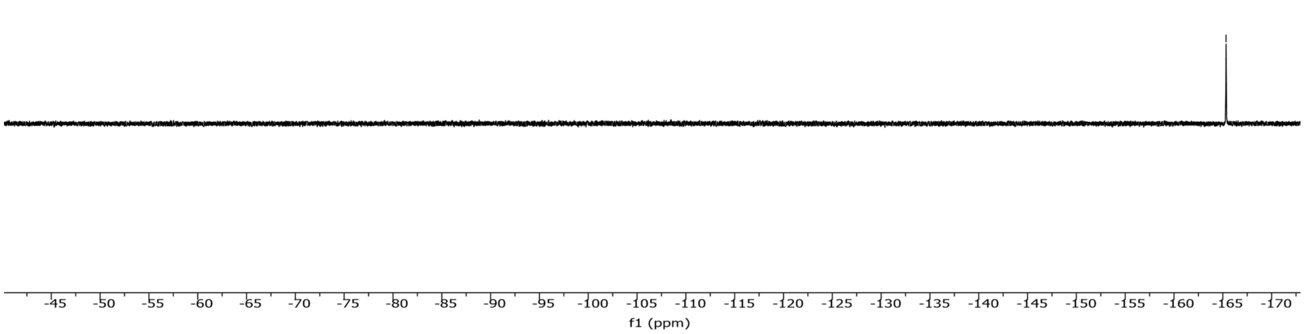

Compound 45

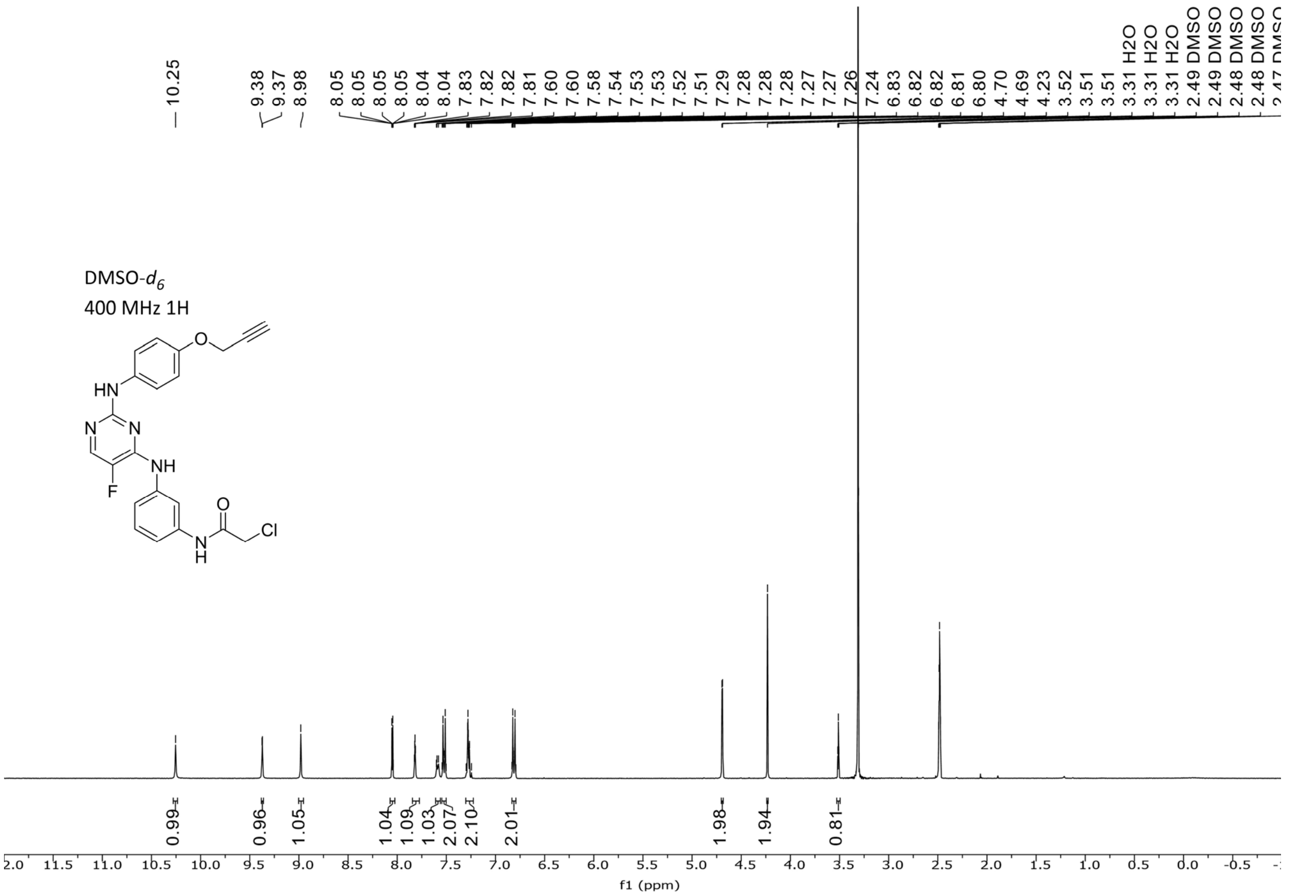
DMSO-d_6
400 MHz 1H
10.25
9.38
9.37
8.98
8.05
8.05
8.05
8.05
8.04
8.04
7.83
7.82
7.82
7.81
7.60
7.60
7.58
7.54
7.53
7.53
7.52
7.51
7.29
7.28
7.28
7.28
7.27
7.27
7.26
7.24
6.83
6.82
6.82
6.81
6.80
4.70
4.69
4.23
3.52
3.51
3.51 H2O
3.31 H2O
3.31 H2O
3.31 H2O
2.49 DMSO
2.49 DMSO
2.48 DMSO
2.48 DMSO
0.99
0.96
1.05
1.04
1.09
1.03
2.07
2.10
2.01
1.98
1.94
0.81
f1 (ppm)

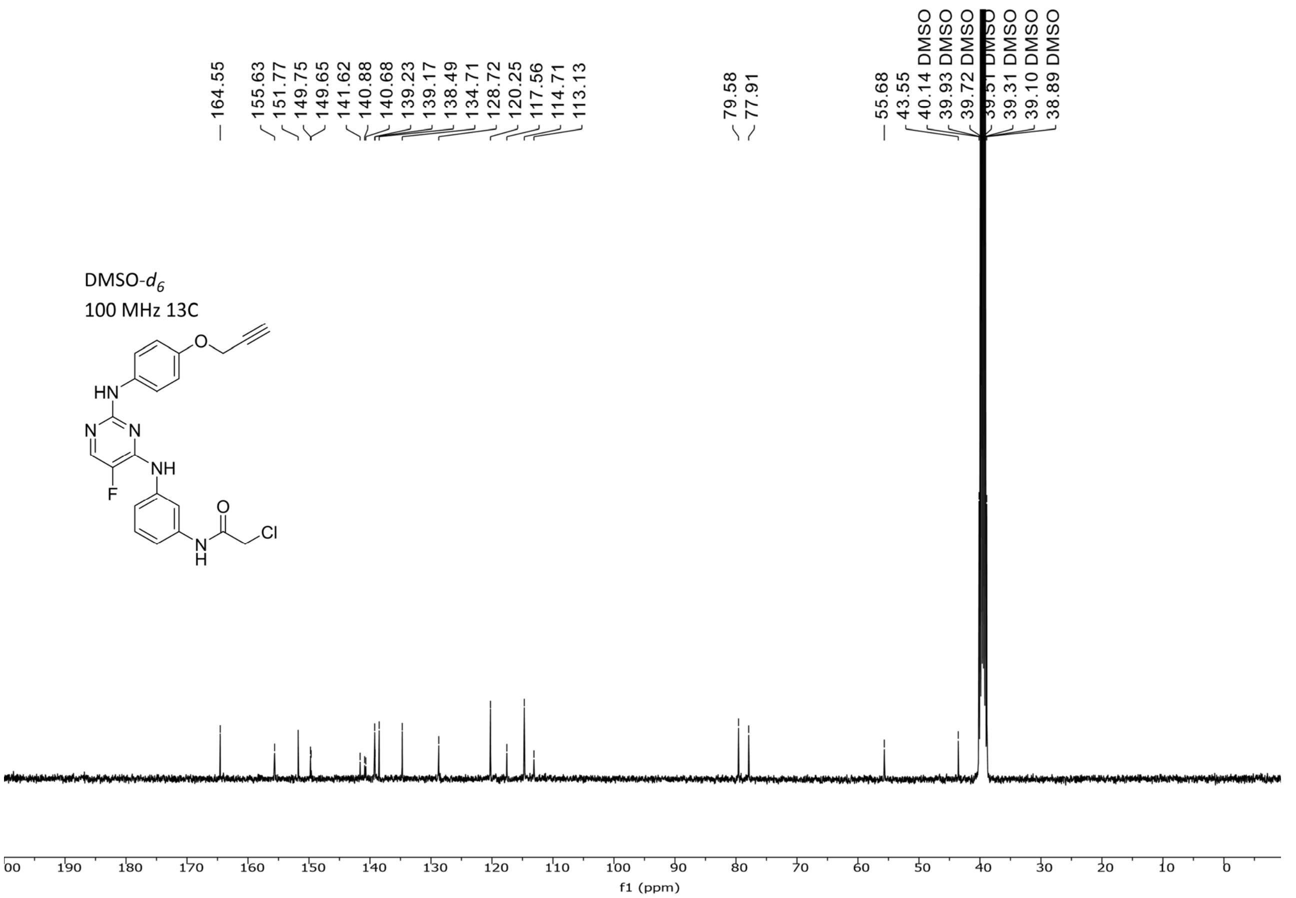
DMSO-d_6
100 MHz 13C
164.55
155.63
151.77
149.75
149.65
141.62
140.88
140.68
139.23
139.17
138.49
134.71
128.72
120.25
117.56
114.71
113.13
79.58
77.91
55.68
43.55
40.14 DMSO
39.93 DMSO
39.72 DMSO
39.51 DMSO
39.31 DMSO
39.10 DMSO
38.89 DMSO
f1 (ppm)

DMSO-d_6
376 MHz 19F

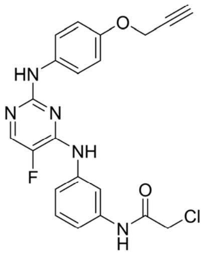

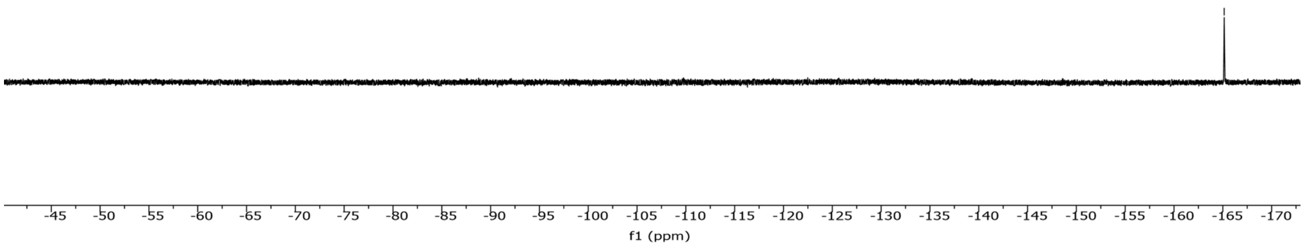

SpePPh$_3$-yne (11)

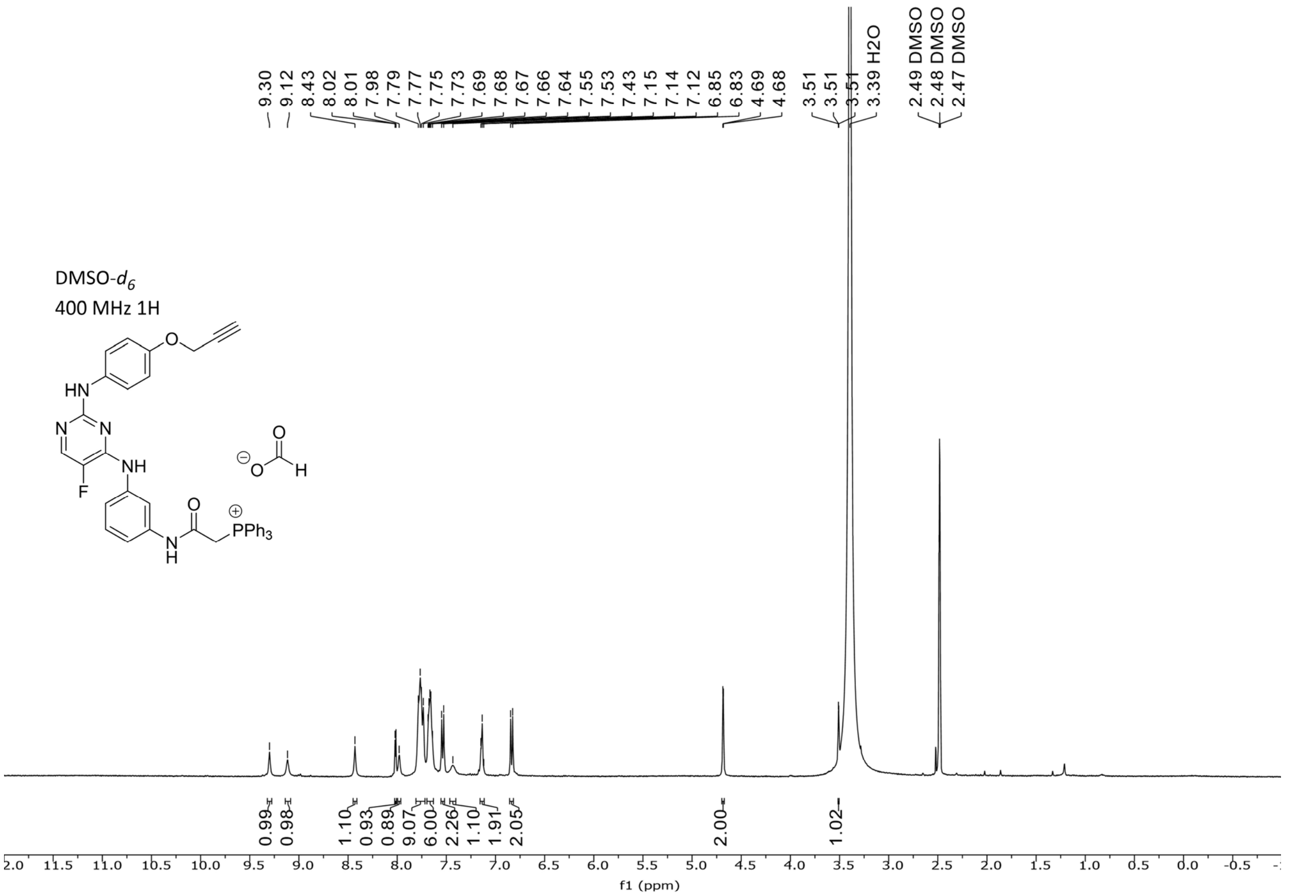

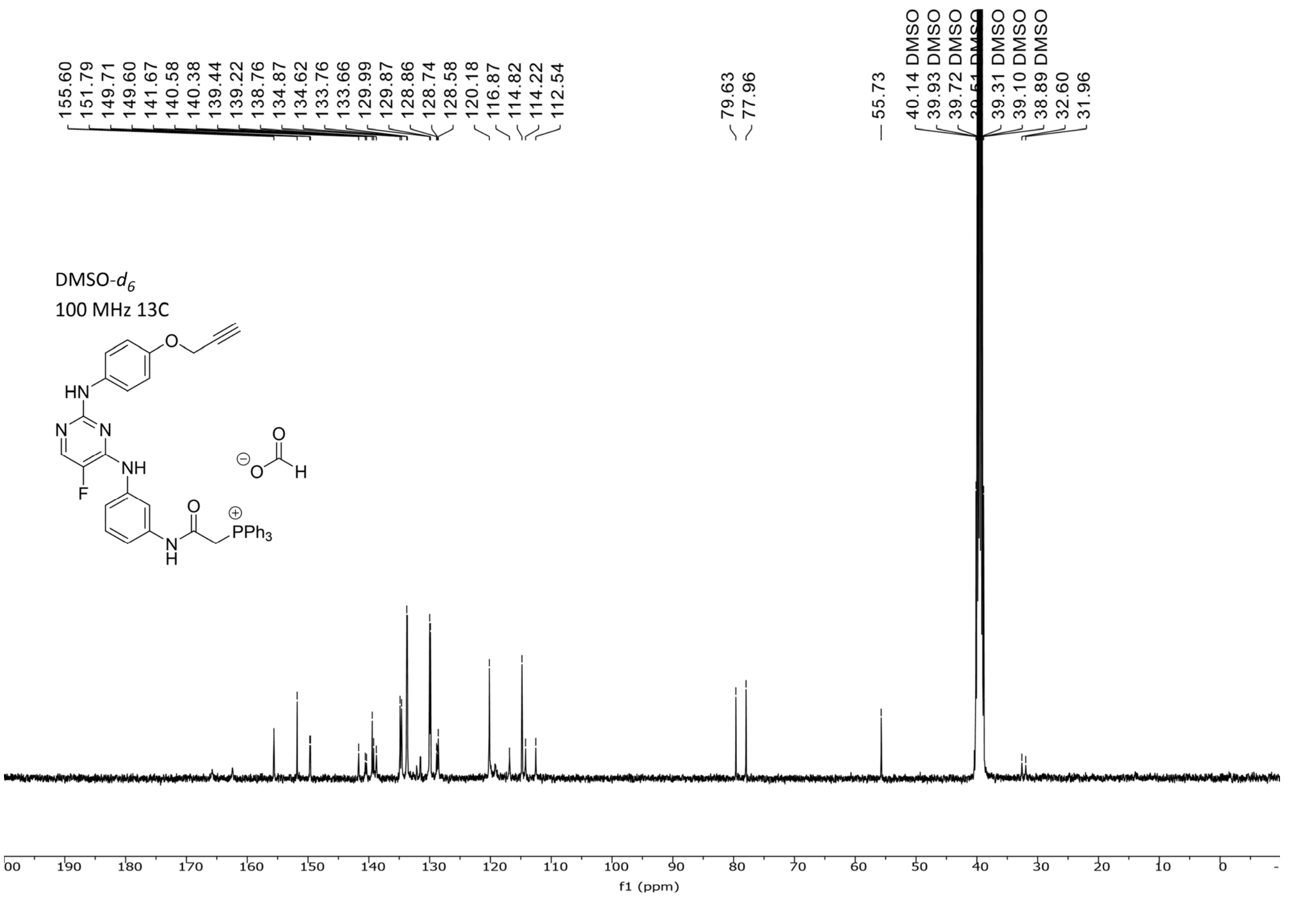

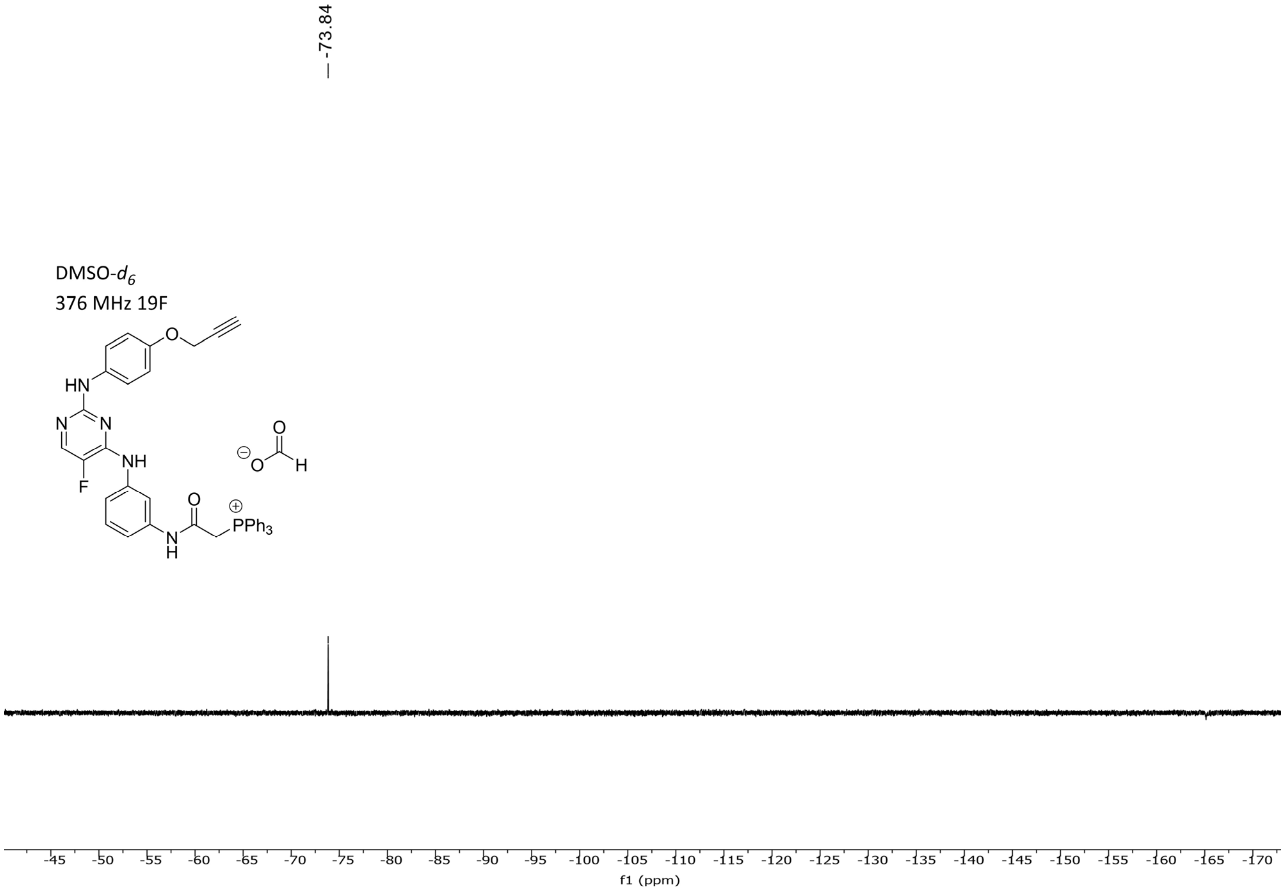
-73.84
DMSO-d_6
376 MHz 19F
f1 (ppm)

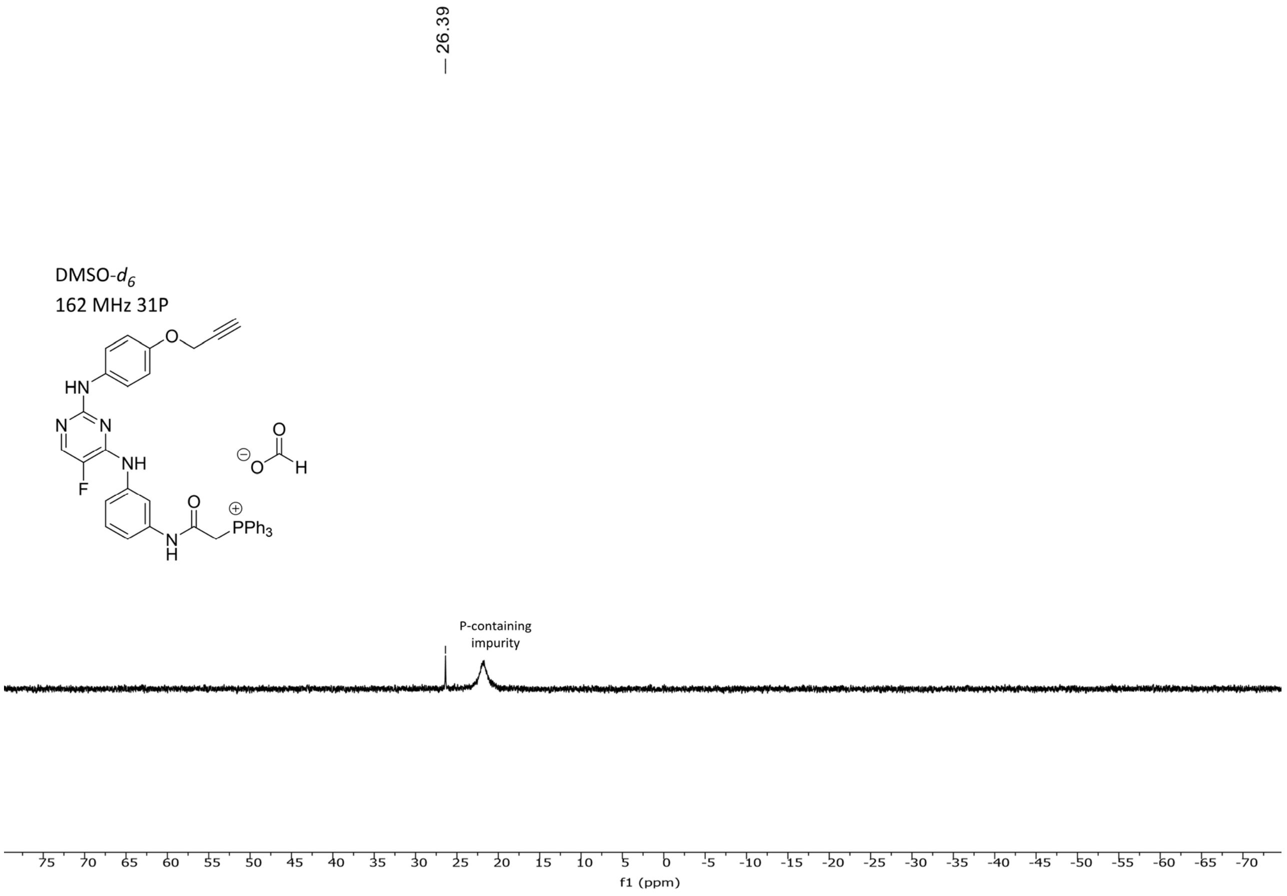
26.39
DMSO-d_6
162 MHz 31P
P-containing impurity
f1 (ppm)
